# Recent noteworthy findings of fungus gnats from Finland and northwestern Russia (Diptera: Ditomyiidae, Keroplatidae, Bolitophilidae and Mycetophilidae)

**DOI:** 10.3897/BDJ.2.e1068

**Published:** 2014-04-02

**Authors:** Jevgeni Jakovlev, Jukka Salmela, Alexei Polevoi, Jouni Penttinen, Noora-Annukka Vartija

**Affiliations:** † Finnish Environment Insitutute, Helsinki, Finland; ‡ Metsähallitus (Natural Heritage Services), Rovaniemi, Finland; § Zoological Museum, University of Turku, Turku, Finland; | Forest Research Institute KarRC RAS, Petrozavodsk, Russia; ¶ Metsähallitus (Natural Heritage Services), Jyväskylä, Finland; # Toivakka, Myllyntie, Finland

**Keywords:** Sciaroidea, Fennoscandia, faunistics

## Abstract

New faunistic data on fungus gnats (Diptera: Sciaroidea excluding Sciaridae) from Finland and NW Russia (Karelia and Murmansk Region) are presented. A total of 64 and 34 species are reported for the first time form Finland and Russian Karelia, respectively. Nine of the species are also new for the European fauna: *Mycomya shewelli* Väisänen, 1984, *Mycomya thula* Väisänen, 1984, *Acnemia trifida* Zaitzev, 1982, *Coelosia gracilis* Johannsen, 1912, *Orfelia krivosheinae* Zaitzev, 1994, *Mycetophila biformis* Maximova, 2002, *Mycetophila monstera* Maximova, 2002, *Mycetophila uschaica* Subbotina & Maximova, 2011 and *Trichonta palustris* Maximova, 2002.

## Introduction

Fungus gnats or mycetophilids (Diptera: Bolitophilidae, Diadocidiidae, Ditomyiidae, Keroplatidae, and Mycetophilidae) are a very rich assemblage of thread-horned (Nematocera) flies with more than 1,100 species that occur in Europe (Bechev 2000, Chandler 2004 and subsequent contributions by various authors). In contrast to many other insect groups, they seem to display an increasing species richness towards the north and are especially common and diverse in boreal forest environments. Research on fungus gnats in the Fennoscandian region has been greatly revitalized during the last two decades, after a period of relatively little activity. At present the north European fungus gnat fauna is the subject of several intensive taxonomical and ecological investigations (see e.g. Kjaerandsen et al. 2007, Kjaerandsen et al. 2009, Jakovlev 2011a, Søli and Rindal 2012, Halme et al. 2013, Polevoi 2013a). Currently, the Checklist of North European (Fennoscandia, NW Russia, Denmark, Iceland) fungus gnats includes 898 species (Kjaerandsen 2012). This makes up a major proportion of the entire European fungus gnat fauna.

Regional checklists have been recently compiled and updated for Sweden (Kjaerandsen et al. 2007), Iceland (Kjaerandsen et al. 2007a), and Norway (Gammelmo and Rindal 2006, Gammelmo and Søli 2006, Rindal and Gammelmo 2007, Kjaerandsen and Jordal 2007, Søli and Rindal 2012). The most recent Fennoscandian checklist is available through the Fungus Gnats Online (Kjaerandsen 2012).

A list of Finnish fungus gnats was provided by W. Hackman (Hackman 1980), including 486 species. During the past decade dozens of new species have been reported from Finland (e.g. Polevoi et al. 2006), including descriptions of new taxa (Polevoi and Hedmark 2004, Jakovlev and Penttinen 2007, Jakovlev and Polevoi 2008, Polevoi and Jakovlev 2011). The 2010 Finnish Red List assessment of fungus gnats was based on a regional species pool consisting of 734 species (Penttinen et al. 2010). The 102 red-listed fungus gnat species include 12 threatened species (Endangered [EN], Vulnerable [VU]: *Symmerus nobilis*, *Macrocera crassicornis*, *Acnemia amoena*, *Anaclileia dziedzickii*, *Sciophila salassea*, *Mycetophila cingulum*, *Mycetophila sigmoides*, *Synplasta bayardi*) and five other species (Near Threatened [NT], Data Deficient [DD]: *Bolitophila ingrica*, *Urytalpa atriceps*, *Brevicornu cognatum*, *Synplasta pseudingeniosa*, *Sceptonia flavipuncta*) that have not formally been reported from Finland. These species are treated here, accompanied with exact occurrence data.

The Russian Karelian fungus gnat fauna was thoroughly treated by Polevoi (Polevoi 2000), totaling a list of 616 species. However, dozens of species have been since either recorded or described from Russian Karelia (e.g. Polevoi and Hedmark 2004, Humala and Polevoi 2008, Polevoi and Jakovlev 2011). Species occurring in Murmansk region have not been listed, but no less than 330 fungus gnats were found from a single nature reserve close to the Finnish and Norwegian border (Polevoi 2010).

In this paper we list a total of 64 and 34 species new to the Finnish and Russian Karelian fauna, respectively; 10 of these species are also reported for the first time from Europe. We also report other noteworthy findings of fungus gnat species made by the authors in Finland and Russia (Murmansk Region and Russian Karelia). A total of 131 fungus gnat species are treated. These additions raise the total number of fungus gnat species recorded from Finland and Russian Karelia to 768 and 676 species, respectively.

## Materials and methods

**Figure 1.**
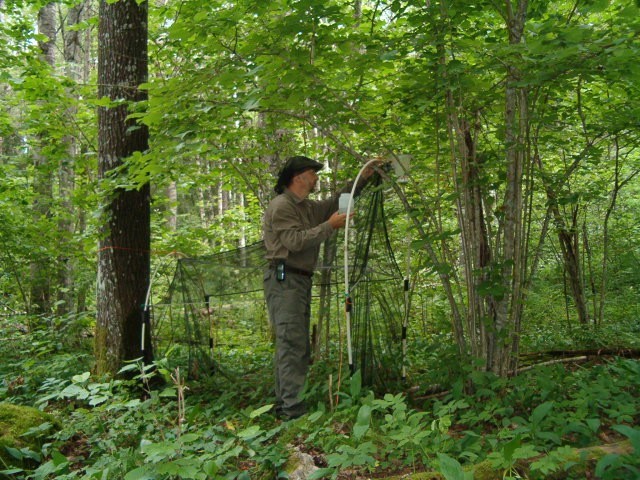
Malaise trapping of forest dwelling insects in Karkali Strict Nature Reserve (Finland, Karjalohja, hemiboreal zone). This nature reserve is one of the most famous Finnish herb-rich forests, harbouring fungus gnat species such as *Mycomya collini* Edwards, *Eudicrana nigriceps* (Lundström) and *Mycetophila sigmoides* Loew.

The majority of the material presented here was collected by using Malaise traps (Fig. 1). Malaise (length 110, height 140, width 70 cm) is a trap model made of cloth (black sides, white “roof”, or unicolorous) and is suitable for collecting low-flying insects, such as dipterans. The traps were usually installed in the beginning of the snow-free season and removed from the field in September or October. During the deployment, collecting jars were emptied in roughly four week intervals. Two types of preservatives were used in the traps: a solution of 50% ethylene glycol + a few drops of detergent, and 70% ethanol. The collected material was finally stored in 70 – 80% ethanol. In addition to Malaise traps, a minor portion of the material was collected with trunk-window traps, eclector traps and sweep netting. Most of the studied specimens are preserved in ethanol, but for some specimens, KOH macerated abdominal terminalia are preserved in separate microvials in glycerol. The following acronyms for museums and collections are used in the text: MZHF – Finnish Museum of Natural History (Zoological Museum), University of Helsinki, Helsinki, Finland; FRIP – Forest Research Institute, Petrozavodsk, Russia; JES – private collection of Jukka Salmela, Rovaniemi, Finland; JPJ – private collection of Jouni Penttinen, Jyväskylä, Finland; JJH – private collection of Jevgeni Jakovlev, Helsinki, Finland.

The arrangement of the treated species follows Bechev (2000): Ditomyiidae, Keroplatidae (Orfelini, Macrocerini), Bolitophilidae, Mycetophilidae (Mycomyinae, Sciophilinae, Gnoristinae, Leiinae, Mycetophilinae [Exechiini, Mycetophilini]).

Extended depth of field photos displaying male terminalia were taken using an Olympus SZX16 stereomicroscope attached to an Olympus E520 digital camera. Digital photos were captured and combined using the programmes Deep Focus 3.1 and Quick PHOTO CAMERA 2.3.

Asterisks after species names correspond to: * – new to Finland, ** – new to the Republic of Karelia and *** – new to Europe. Red List acronyms given here follow IUCN categories: DD = Data Deficient, EN = Endangered, VU = Vulnerable, NT = Near Threatened.

## Taxon treatments

### Symmerus annulatus

(Meigen, 1830)**

http://www.faunaeur.org/full_results.php?id=135078

#### Materials

- **Type status:** Other material. **Occurrence:** recordedBy: A. Polevoi; individualCount: 1; sex: male; **Location:** country: Russia; stateProvince: Republic Karelia; verbatimLocality: Lahdenpohja, 9 km NE of Sukopohja; decimalLatitude: 61.688; decimalLongitude: 30.159; geodeticDatum: WGS84; **Identification:** identifiedBy: A. Polevoi; **Event:** samplingProtocol: Sweep net; eventDate: 2005-7-7; **Record Level:** institutionCode: FRIP
- **Type status:** Other material. **Occurrence:** recordedBy: J.Penttinen; individualCount: 1; sex: male; **Location:** country: Finland; stateProvince: Karelia ladogensis; municipality: Parikkala; locality: Siikalahti; decimalLatitude: 61.556; decimalLongitude: 29.558; geodeticDatum: WGS84; **Identification:** identifiedBy: J.Penttinen; **Event:** samplingProtocol: Malaise trap; eventDate: 2008; habitat: old-growth forest, herb-rich type; **Record Level:** institutionCode: JPJ
- **Type status:** Other material. **Occurrence:** recordedBy: J.Jakovlev; individualCount: 1; sex: male; **Location:** country: Finland; stateProvince: Nylandia; municipality: Kirkkonummi; locality: Kuokkamaa; decimalLatitude: 60.121; decimalLongitude: 24.608; geodeticDatum: WGS84; **Identification:** identifiedBy: J.Jakovlev; **Event:** samplingProtocol: Malaise trap; eventDate: 2010-7-12/8-23; habitat: old-growth forest, herb-rich type; **Record Level:** institutionCode: JJH
- **Type status:** Other material. **Occurrence:** recordedBy: J.Jakovlev; individualCount: 1; sex: male; **Location:** country: Finland; stateProvince: Nylandia; municipality: Kirkkonummi; locality: Kuokkamaa; decimalLatitude: 60.121; decimalLongitude: 24.608; geodeticDatum: WGS84; **Identification:** identifiedBy: J.Jakovlev; **Event:** samplingProtocol: Malaise trap; eventDate: 2010-8-23/10-9; habitat: old-growth forest, herb-rich type; **Record Level:** institutionCode: JJH
- **Type status:** Other material. **Occurrence:** recordedBy: J.Jakovlev; individualCount: 2; sex: male; **Location:** country: Finland; stateProvince: Nylandia; municipality: Kirkkonummi; locality: Kuokkamaa; decimalLatitude: 60.121; decimalLongitude: 24.608; geodeticDatum: WGS84; **Identification:** identifiedBy: J.Jakovlev; **Event:** samplingProtocol: Malaise trap; eventDate: 2010-6-17/7-13; habitat: old-growth forest, herb-rich type; **Record Level:** institutionCode: JJH
- **Type status:** Other material. **Occurrence:** recordedBy: J.Jakovlev; individualCount: 1; sex: male; **Location:** country: Finland; stateProvince: Nylandia; municipality: Kirkkonummi; locality: Kuokkamaa; decimalLatitude: 60.121; decimalLongitude: 24.608; geodeticDatum: WGS84; **Identification:** identifiedBy: J.Jakovlev; **Event:** samplingProtocol: Reared from wood; eventDate: 2010-7-12/8-23; habitat: old-growth forest, herb-rich type; **Record Level:** institutionCode: JJH
- **Type status:** Other material. **Occurrence:** recordedBy: J.Jakovlev; individualCount: 2; sex: 1 male, 1 female; **Location:** country: Finland; stateProvince: Nylandia; municipality: Kirkkonummi; locality: Kuokkamaa; decimalLatitude: 60.121; decimalLongitude: 24.608; geodeticDatum: WGS84; **Identification:** identifiedBy: J.Jakovlev; **Event:** samplingProtocol: Reared from wood; eventDate: 2010-7-17/7-13; habitat: old-growth forest, herb-rich type; **Record Level:** institutionCode: JJH
- **Type status:** Other material. **Occurrence:** recordedBy: J.Jakovlev; individualCount: 1; sex: male; **Location:** country: Finland; stateProvince: Nylandia; municipality: Kirkkonummi; locality: Kuokkamaa; decimalLatitude: 60.121; decimalLongitude: 24.608; geodeticDatum: WGS84; **Identification:** identifiedBy: J.Jakovlev; **Event:** samplingProtocol: Reared from wood; eventDate: 2010-8-23/10-9; habitat: old-growth forest, herb-rich type; **Record Level:** institutionCode: JJH
- **Type status:** Other material. **Occurrence:** recordedBy: J.Jakovlev; individualCount: 1; sex: male; **Location:** country: Finland; stateProvince: Nylandia; municipality: Kirkkonummi; locality: Norra flaget; decimalLatitude: 60.113; decimalLongitude: 24.625; geodeticDatum: WGS84; **Identification:** identifiedBy: J.Jakovlev; **Event:** samplingProtocol: Malaise trap; eventDate: 2010-8-23/10-9; habitat: old-growth forest, herb-rich type; **Record Level:** institutionCode: JJH
- **Type status:** Other material. **Occurrence:** recordedBy: J.Jakovlev; individualCount: 2; sex: male; **Location:** country: Finland; stateProvince: Nylandia; municipality: Kirkkonummi; locality: Norra flaget; decimalLatitude: 60.113; decimalLongitude: 24.625; geodeticDatum: WGS84; **Identification:** identifiedBy: J.Jakovlev; **Event:** samplingProtocol: Malaise trap; eventDate: 2010-6-19/7-13; habitat: old-growth forest, herb-rich type; **Record Level:** institutionCode: JJH
- **Type status:** Other material. **Occurrence:** recordedBy: J.Jakovlev; individualCount: 1; sex: male; **Location:** country: Finland; stateProvince: Nylandia; municipality: Kirkkonummi; locality: Norra flaget; decimalLatitude: 60.113; decimalLongitude: 24.625; geodeticDatum: WGS84; **Identification:** identifiedBy: J.Jakovlev; **Event:** samplingProtocol: Reared from wood; eventDate: 2010-8-23/10-9; habitat: old-growth forest, herb-rich type; **Record Level:** institutionCode: JJH
- **Type status:** Other material. **Occurrence:** recordedBy: J.Jakovlev; individualCount: 2; sex: male; **Location:** country: Finland; stateProvince: Regio aboënsis; municipality: Tammisaari; locality: Dragsvikin kartano; decimalLatitude: 60.001; decimalLongitude: 23.491; geodeticDatum: WGS84; **Identification:** identifiedBy: J.Jakovlev; **Event:** samplingProtocol: Malaise trap; eventDate: 2010-6-18/7-14; habitat: two isolated herb-rich forests divided with sea gulf and wet meadow. Spruce-dominated, partly dominated with Alnus glutinosa. Huge willows; **Record Level:** institutionCode: JJH
- **Type status:** Other material. **Occurrence:** recordedBy: J.Jakovlev; individualCount: 1; sex: male; **Location:** country: Finland; stateProvince: Regio aboënsis; municipality: Tammisaari; locality: Dragsvikin kartano; decimalLatitude: 60.001; decimalLongitude: 23.491; geodeticDatum: WGS84; **Identification:** identifiedBy: J.Jakovlev; **Event:** samplingProtocol: Malaise trap; eventDate: 2010-8-24/9-4; habitat: two isolated herb-rich forests divided with sea gulf and wet meadow. Spruce-dominated, partly dominated with Alnus glutinosa. Huge willows; **Record Level:** institutionCode: JJH

#### Distribution

Palaearctic, besides Europe recorded from Caucasus and West Siberia (Zaitzev 1994). Widely distributed in Europe, including Fennoscandia (Chandler 2004). Recorded only from southern areas in Sweden (Kjaerandsen et al. 2007) and Norway (Gammelmo and Rindal 2006). In Finland has been found also in the southernmost parts of the country (as *Pleasiastina annulata*, Lundström 1909). New to the Republic of Karelia.

#### Ecology

Larvae develop in rotten wood, feeding on the mycelia that it contains. The species has been reared from larvae found in rotten wood of deciduous trees only: beech (*Fagus*), elm (*Ulmus*) and lime (*Tilia*) (Krivosheina and Mamaev 1967, Zaitzev 1994). Chandler (Chandler 1993) reported the rearing of *Symmerus annulatus* from a hard ascomycete fungus *Hypoxylon rubiginosum*. The Karelian specimen was collected from a young grey alder (*Alnus incana*) forest.

#### Conservation

Red-listed in Norway (VU, Anonymous 2010, Gammelmo et al. 2010).

### Symmerus nobilis

Lackschewitz, 1937*

http://www.faunaeur.org/full_results.php?id=135088

#### Materials

- **Type status:** Other material. **Occurrence:** recordedBy: A. Polevoi; individualCount: 1; sex: male; **Location:** country: Russia; stateProvince: Republic Karelia; verbatimLocality: 3 km S of Kosmozero; decimalLatitude: 62.297; decimalLongitude: 35.088; geodeticDatum: WGS84; **Identification:** identifiedBy: A. Polevoi; **Event:** samplingProtocol: Sweep net; eventDate: 2013-6-26; **Record Level:** institutionCode: FRIP
- **Type status:** Other material. **Occurrence:** recordedBy: J. Jakovlev; G. Ståhls; individualCount: 1; sex: male; **Location:** country: Finland; stateProvince: Regio aboensis; verbatimLocality: Turku, Ruissalo; decimalLatitude: 60.432; decimalLongitude: 22.165; geodeticDatum: WGS84; **Identification:** identifiedBy: J. Jakovlev; **Event:** samplingProtocol: Malaise trap; eventDate: 2005-5-11/6-20; **Record Level:** institutionCode: JJH

#### Distribution

European. *Symmerus nobilis* was described from Latvia (Lackschewitz 1937) and has been found in several countries of Central Europe (Chandler 2004, Zaitzev 1994), but is considered everywhere a rare species. From the well-studied British Isles it was recorded only from one site in Scotland (Glen Coiltie, Easterness) (Falk and Chandler 2005). In the Fennoscandian region, the species was recorded only recently from southern parts of Norway (Gammelmo and Rindal 2006, Kjaerandsen and Jordal 2007), south Sweden (Jakovlev et al. 2008), the Kivach Nature Reserve in Russian Karelia (two female specimens, Polevoi 2000). No former records from Finland.

#### Ecology

All collecting records of adults are from broadleaved forests, with the exception of Russian Karelia which lies entirely in the boreal forest zone. The Russian Karelian sites are spruce dominated forests with a high proportion of aspen (*Populus tremula*). The Finnish record is from a herb-rich spruce-dominated forest with aspen, birch, lime and oak (*Quercus robur*). Both the Finnish and the Karelian sites are old growth forests on fertile soils with a high amount of dead aspen wood, in which larvae of the species most likely develop. Larvae live in decaying wood, as indicated by rearing records from beech (Zaitzev 1994).

#### Conservation

Red-listed in Finland (VU, Penttinen et al. 2010), Norway (VU, Anonymous 2010, Gammelmo et al. 2010) and Sweden (NT, Cederberg et al. 2010).

### Isoneuromyia semirufa

Meigen, 1818

http://www.faunaeur.org/full_results.php?id=138603

#### Materials

- **Type status:** Other material. **Occurrence:** individualCount: 1; sex: male; **Taxon:** genus: Isoneuromyia; specificEpithet: semirufa; scientificNameAuthorship: Meigen, 1818; **Location:** country: Finland; stateProvince: Regio kuusamoensis; municipality: Kuusamo; locality: Kuusamo; decimalLatitude: 65.967; decimalLongitude: 29.174; geodeticDatum: WGS84; **Event:** habitat: boreal forest_Myrtillus type; **Record Level:** institutionCode: MZHF
- **Type status:** Other material. **Occurrence:** recordedBy: J.Jakovlev; individualCount: 1; sex: male; **Taxon:** genus: Isoneuromyia; specificEpithet: semirufa; scientificNameAuthorship: Meigen, 1818; **Location:** country: Finland; stateProvince: Nylandia; municipality: Kirkkonummi; locality: Kuokkamaa; decimalLatitude: 60.121; decimalLongitude: 24.608; geodeticDatum: WGS84; **Identification:** identifiedBy: J.Jakovlev; **Event:** samplingProtocol: Malaise trap; eventDate: 2010-6-20/8-23; habitat: old-growth forest_herb-rich type; **Record Level:** institutionCode: JJH
- **Type status:** Other material. **Occurrence:** recordedBy: J.Jakovlev; individualCount: 1; sex: male; **Taxon:** genus: Isoneuromyia; specificEpithet: semirufa; scientificNameAuthorship: Meigen, 1818; **Location:** country: Finland; stateProvince: Nylandia; municipality: Helsinki; locality: Itäsalmi; decimalLatitude: 60.252; decimalLongitude: 25.204; geodeticDatum: WGS84; **Identification:** identifiedBy: J.Jakovlev; **Event:** samplingProtocol: Malaise trap; eventDate: 2010-6-15/7-23; habitat: herb-rich forest dominated by aspen and spruce, young maples.; **Record Level:** institutionCode: JJH
- **Type status:** Other material. **Occurrence:** recordedBy: J.Penttinen; individualCount: 1; sex: male; **Taxon:** genus: Isoneuromyia; specificEpithet: semirufa; scientificNameAuthorship: Meigen, 1818; **Location:** country: Finland; stateProvince: Regio aboënsis; municipality: Salo; locality: Vaisakko; decimalLatitude: 60.361; decimalLongitude: 23.045; geodeticDatum: WGS84; **Identification:** identifiedBy: J.Penttinen; **Event:** eventDate: 2009-6-1/6-15; habitat: herb-rich forest_seminatural; **Record Level:** institutionCode: JPJ
- **Type status:** Other material. **Occurrence:** recordedBy: J.Jakovlev; individualCount: 1; sex: male; **Taxon:** genus: Isoneuromyia; specificEpithet: semirufa; scientificNameAuthorship: Meigen, 1818; **Location:** country: Finland; stateProvince: Regio aboënsis; municipality: Karjalohja; locality: Karkali_South; decimalLatitude: 60.238; decimalLongitude: 23.785; geodeticDatum: WGS84; **Identification:** identifiedBy: J.Jakovlev; **Event:** samplingProtocol: Malaise trap; eventDate: 2004-6-1/6-15; habitat: old-growth forest_herb-rich type; **Record Level:** institutionCode: JJH
- **Type status:** Other material. **Occurrence:** recordedBy: M.Jaschhof and C.Jaschhof; individualCount: 1; sex: male; **Taxon:** genus: Isoneuromyia; specificEpithet: semirufa; scientificNameAuthorship: Meigen, 1818; **Location:** country: Finland; stateProvince: Satakunta; municipality: Ikaalinen; locality: Multinharju; decimalLatitude: 61.907; decimalLongitude: 23.299; geodeticDatum: WGS84; **Identification:** identifiedBy: J.Jakovlev; **Event:** samplingProtocol: Malaise trap; eventDate: 2004-7-2/8-24; habitat: old-growth forest_Myrtillus type; **Record Level:** institutionCode: JJH
- **Type status:** Other material. **Occurrence:** recordedBy: R.Tuomikoski; individualCount: 1; sex: male; **Taxon:** genus: Isoneuromyia; specificEpithet: semirufa; scientificNameAuthorship: Meigen, 1818; **Location:** country: Finland; stateProvince: Regio kuusamoënsis; municipality: Kuusamo; locality: Kuusamo; decimalLatitude: 65.966; decimalLongitude: 29.174; geodeticDatum: WGS84; **Identification:** identifiedBy: W.Hackman; **Event:** samplingProtocol: Sweep netting; eventDate: 1964; **Record Level:** institutionCode: MZHF
- **Type status:** Other material. **Occurrence:** recordedBy: R.Frey; individualCount: 1; sex: male; **Taxon:** genus: Isoneuromyia; specificEpithet: semirufa; scientificNameAuthorship: Meigen, 1818; **Location:** country: Finland; stateProvince: Satakunta; municipality: Yläne; locality: Yläne; decimalLatitude: 60.870; decimalLongitude: 22.393; geodeticDatum: WGS84; **Identification:** identifiedBy: C.Lundström; **Event:** eventDate: 1900; **Record Level:** institutionCode: MZHF
- **Type status:** Other material. **Occurrence:** recordedBy: J.Sahlberg; individualCount: 1; sex: male; **Taxon:** genus: Isoneuromyia; specificEpithet: semirufa; scientificNameAuthorship: Meigen, 1818; **Location:** country: Finland; stateProvince: Regio aboënsis; municipality: Karjalohja; locality: Sammati; decimalLatitude: 60.319; decimalLongitude: 23.829; geodeticDatum: WGS84; **Identification:** identifiedBy: C.Lundström; **Event:** eventDate: 1900; **Record Level:** institutionCode: MZHF
- **Type status:** Other material. **Occurrence:** recordedBy: Krogerus; individualCount: 1; sex: female; **Taxon:** genus: Isoneuromyia; specificEpithet: semirufa; scientificNameAuthorship: Meigen, 1818; **Location:** country: Finland; stateProvince: Tavastia australis; municipality: Forssa; locality: Forssa; decimalLatitude: 60.817; decimalLongitude: 23.614; geodeticDatum: WGS84; **Identification:** identifiedBy: C.Lundström; **Event:** eventDate: 1900; **Record Level:** institutionCode: MZHF
- **Type status:** Other material. **Occurrence:** recordedBy: J. Salmela; individualCount: 1; sex: male; **Taxon:** genus: Isoneuromyia; specificEpithet: semirufa; scientificNameAuthorship: Meigen, 1818; **Location:** country: Finland; stateProvince: Ostrobothnia borealis pars borealis; verbatimLocality: Tornio, Rakanjänkkä; decimalLatitude: 65.890; decimalLongitude: 24.317; geodeticDatum: WGS84; **Event:** eventDate: 2012-7-2/8-6; habitat: rich spring fen; **Record Level:** institutionCode: JES
- **Type status:** Other material. **Occurrence:** catalogNumber: MYCE-NV-2013-0060; recordedBy: J. Salmela; individualCount: 1; sex: male; **Taxon:** genus: Isoneuromyia; specificEpithet: semirufa; scientificNameAuthorship: Meigen, 1818; **Location:** country: Finland; stateProvince: Lapponia kemensis pars occidentalis; verbatimLocality: Kittilä, Silmäsvuoma; decimalLatitude: 67.582; decimalLongitude: 25.543; geodeticDatum: WGS84; **Identification:** identifiedBy: J. Salmela; **Event:** samplingProtocol: Malaise trap; eventDate: 2007-6-26/7-27; habitat: rich fen, aapamire; **Record Level:** institutionCode: JES
- **Type status:** Other material. **Occurrence:** catalogNumber: MYCE-NV-2013-0098; recordedBy: J. Salmela; individualCount: 1; sex: male; **Taxon:** genus: Isoneuromyia; specificEpithet: semirufa; scientificNameAuthorship: Meigen, 1818; **Location:** country: Finland; stateProvince: Lapponia kemensis pars occidentalis; verbatimLocality: Kittilä, Kielisenpalo; decimalLatitude: 68.020; decimalLongitude: 25.063; geodeticDatum: WGS84; **Identification:** identifiedBy: J. Salmela; **Event:** samplingProtocol: Malaise trap; eventDate: 2007-6-26/7-27; habitat: rich spring fen; **Record Level:** institutionCode: JES
- **Type status:** Other material. **Occurrence:** catalogNumber: MYCE-JS-2012-0050; recordedBy: Jukka Salmela; individualCount: 3; sex: male; **Taxon:** genus: Isoneuromyia; specificEpithet: semirufa; scientificNameAuthorship: Meigen, 1818; **Location:** country: Finland; stateProvince: Ostrobothnia borealis pars borealis; verbatimLocality: Tornio, Ruuttulammi; decimalLatitude: 66.207; decimalLongitude: 24.898; geodeticDatum: WGS84; **Identification:** identifiedBy: J. Salmela; **Event:** samplingProtocol: Malaise trap; eventDate: 2012-7-2/8-6; habitat: rich fen; **Record Level:** institutionCode: JES
- **Type status:** Other material. **Occurrence:** recordedBy: Jukka Salmela; individualCount: 1; sex: male; **Taxon:** genus: Isoneuromyia; specificEpithet: semirufa; scientificNameAuthorship: Meigen, 1818; **Location:** country: Finland; stateProvince: Lapponia kemensis pars orientalis; verbatimLocality: Savukoski, Joutenoja; decimalLatitude: 67.821; decimalLongitude: 29.440; geodeticDatum: WGS84; **Event:** samplingProtocol: Malaise trap; eventDate: 2012-7-10/8-16; habitat: Headwater stream, old-growth boreal forest; **Record Level:** institutionCode: JES
- **Type status:** Other material. **Occurrence:** catalogNumber: MYCE-JS-2013-0036; recordedBy: Jukka Salmela; individualCount: 1; sex: male; **Taxon:** genus: Isoneuromyia; specificEpithet: semirufa; scientificNameAuthorship: Meigen, 1818; **Location:** country: Finland; stateProvince: Lapponia kemensis pars orientalis; verbatimLocality: Savukoski, Törmäoja; decimalLatitude: 67.846; decimalLongitude: 29.471; geodeticDatum: WGS84; **Identification:** identifiedBy: J. Salmela; **Event:** samplingProtocol: Malaise trap; eventDate: 2012-7-10/8-16; habitat: Headwater stream, old-growth boreal forest; **Record Level:** institutionCode: JES
- **Type status:** Other material. **Occurrence:** catalogNumber: DIPT-JS-2014-0032; recordedBy: J. Salmela; T. Hietajärvi; individualCount: 1; sex: male; **Location:** country: Finland; stateProvince: Regio kuusamoensis; verbatimLocality: Salla, Kuntasjoki, Värriö Strict Nature Reserve; verbatimElevation: 320 m; decimalLatitude: 67.749; decimalLongitude: 29.617; geodeticDatum: WGS84; **Identification:** identifiedBy: J. Salmela; **Event:** samplingProtocol: Malaise trap; eventDate: 2013; verbatimEventDate: 2013-6-29/7-29; habitat: headwater stream, old-growth boreal forest; **Record Level:** institutionCode: JES

#### Distribution

Holarctic, widely distributed in Europe (Chandler 2004). Wide range in Sweden (Kjaerandsen et al. 2007) and in the Republic of Karelia (Polevoi 2013a). In Finland mainly collected from the southern parts of the country, except for an old record from NW Lapland, Muonio (Lundström 1914).

#### Ecology

**Figure 2.**
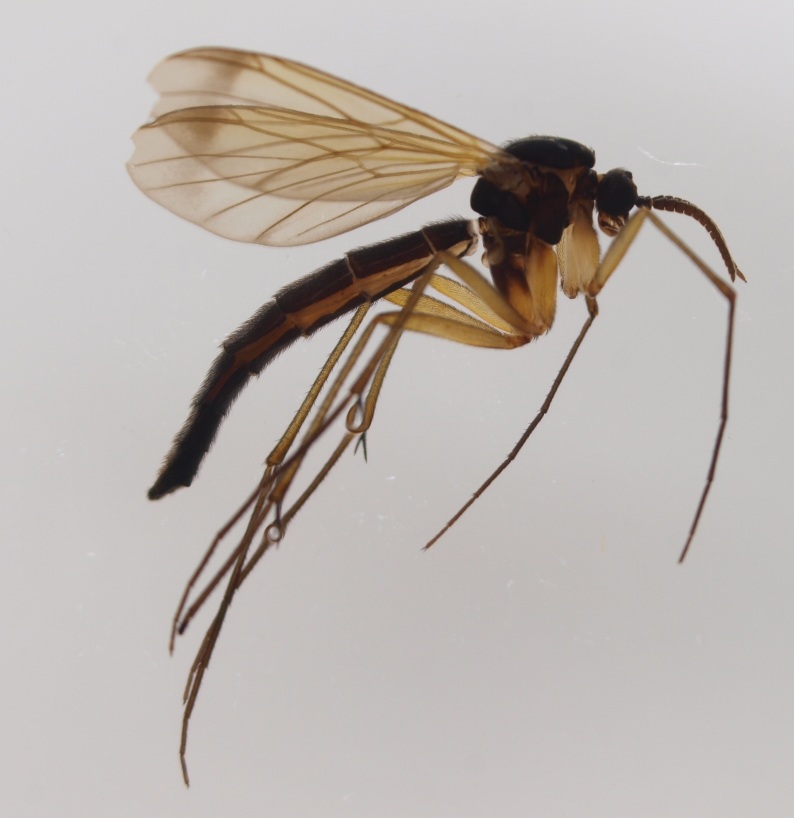
*Isoneuromyia semirufa* (Meigen), male specimen collected from Tervola, North Finland, lateral view.

New records from Lapland are mainly from calcareous spring fens and rich fens, but also from headwater streams with rich riparian vegetation. In the light of these new records *Isoneuromyia semirufa* (Fig. 2) is perhaps not confined to forest habitats, but may also thrive in peatlands. Immature stages are unknown.

#### Conservation

Red-listed in Finland (NT, Penttinen et al. 2010).

### Orfelia krivosheinae

Zaitzev, 1994***

http://www.catalogueoflife.org/col/details/species/id/8717088

#### Materials

- **Type status:** Other material. **Occurrence:** catalogNumber: MYCE-JS-2013-0269; recordedBy: Seppo Karjalainen; individualCount: 1; sex: male; **Location:** country: Finland; stateProvince: Karelia ladogensis; verbatimLocality: Parikkala, Niukkala; decimalLatitude: 61.729; decimalLongitude: 29.887; geodeticDatum: WGS84; **Identification:** identifiedBy: J. Salmela; J. Jakovlev; **Event:** samplingProtocol: Trunk window trap on *Betula* trunk; eventDate: 2012-6-20/7-19; **Record Level:** institutionCode: JES

#### Distribution

Palaearctic. *Orfelia krivosheinae* (Fig. 3) was described from Russia, Tuva (Central Asia, Zaitzev 1994). No published records are available besides the original description. New for Europe.

#### Ecology

The type material was reared from larvae found on mycelium in a decaying poplar (*Populus*) tree (Zaitzev 1994). The Finnish specimen was taken from a trunk-window trap set on a birch trunk.

### Orfelia lugubris

(Zetterstedt, 1851)*

#### Materials

- **Type status:** Other material. **Occurrence:** catalogNumber: MYCE-JS-2013-0208; recordedBy: J. Salmela; individualCount: 1; sex: male; **Location:** country: Finland; stateProvince: Ostrobothnia borealis pars borealis; verbatimLocality: Tervola, Ruuttulammi; decimalLatitude: 66.207; decimalLongitude: 24.898; geodeticDatum: WGS84; **Identification:** identifiedBy: J. Salmela; **Event:** samplingProtocol: Malaise trap; eventDate: 2012-8-6/9-26; habitat: rich spring fen; **Record Level:** institutionCode: JES
- **Type status:** Other material. **Occurrence:** catalogNumber: MYCE-JS-2012-0046; recordedBy: J. Salmela; individualCount: 1; sex: male; **Location:** country: Finland; stateProvince: Lapponia kemensis pars orientalis; verbatimLocality: Savukoski, Joutenoja; decimalLatitude: 67.821; decimalLongitude: 29.440; geodeticDatum: WGS84; **Identification:** identifiedBy: J. Salmela; **Event:** samplingProtocol: Malaise trap; eventDate: 2012-7-10/8-16; habitat: headwater stream, seminatural boreal forest; **Record Level:** institutionCode: JES
- **Type status:** Other material. **Occurrence:** catalogNumber: MYCE-JS-2013-0021; recordedBy: J. Salmela; individualCount: 1; sex: male; **Location:** country: Finland; stateProvince: Lapponia kemensis pars orientalis; verbatimLocality: Savukoski, Joutenoja; decimalLatitude: 67.821; decimalLongitude: 29.440; geodeticDatum: WGS84; **Identification:** identifiedBy: J. Salmela; **Event:** samplingProtocol: Malaise trap; eventDate: 2012-8-16/9-18; habitat: headwater stream, seminatural boreal forest; **Record Level:** institutionCode: JES
- **Type status:** Other material. **Occurrence:** catalogNumber: MYCE-JS-2013-0038; recordedBy: J. Salmela; individualCount: 1; sex: male; **Location:** country: Finland; stateProvince: Lapponia kemensis pars orientalis; verbatimLocality: Savukoski, Törmäoja; decimalLatitude: 67.846; decimalLongitude: 29.471; geodeticDatum: WGS84; **Identification:** identifiedBy: J. Salmela; **Event:** samplingProtocol: Malaise trap; eventDate: 2012-7-10/8-16; habitat: headwater stream, old-growth boreal forest; **Record Level:** institutionCode: JES
- **Type status:** Other material. **Occurrence:** recordedBy: A. Palmen; individualCount: 1; sex: male; **Location:** country: Finland; stateProvince: Lapponia enontekiensis; verbatimLocality: Enontekiö, Palojoensuu; decimalLatitude: 68.285; decimalLongitude: 23.095; geodeticDatum: WGS84; **Identification:** identifiedBy: C. Lundström; J. Jakovlev; **Event:** eventDate: 1865; **Record Level:** institutionCode: MZHF
- **Type status:** Other material. **Occurrence:** recordedBy: A. Humala; individualCount: 1; sex: male; **Location:** country: Russia; stateProvince: Republic Karelia; verbatimLocality: White Sea, is. Pechak; decimalLatitude: 64.625; decimalLongitude: 35.631; geodeticDatum: WGS84; **Identification:** identifiedBy: A. Polevoi; **Event:** samplingProtocol: sweep net; eventDate: 2001-7-24; **Record Level:** institutionCode: FRIP
- **Type status:** Other material. **Occurrence:** recordedBy: A. Humala; individualCount: 2; sex: male; **Location:** country: Russia; stateProvince: Republic Karelia; verbatimLocality: White Sea, is. Russkiy Kuzov; decimalLatitude: 64.935; decimalLongitude: 35.128; geodeticDatum: WGS84; **Identification:** identifiedBy: A. Polevoi; **Event:** samplingProtocol: sweep net; eventDate: 2001-7-18; **Record Level:** institutionCode: FRIP
- **Type status:** Other material. **Occurrence:** recordedBy: A. Humala; individualCount: 1; sex: male; **Location:** country: Russia; stateProvince: Republic Karelia; verbatimLocality: White Sea, Perhludy archipelago, is. Yuzhnyi; decimalLatitude: 64.323; decimalLongitude: 36.481; geodeticDatum: WGS84; **Identification:** identifiedBy: A. Polevoi; **Event:** samplingProtocol: sweep net; eventDate: 2002-8-16; **Record Level:** institutionCode: FRIP

#### Distribution

Palaearctic. Widely distributed in Europe. Many of the older records were made as *Orfelia tristis* Lundström, a junior synonym of *Orfelia lugubris* (Kjaerandsen et al. 2007). The species occurs in central and northern Sweden (Kjaerandsen et al. 2007), the White Sea shore in Russian Karelia (Humala and Polevoi 2008), and Norway (Kjaerandsen 2012). New for Finland.

#### Ecology

Finnish records are from rich spring fens and headwater streams surrounded by coniferous forests. Karelian specimens were collected in riparian habitats of the White Sea. Immature stages are unknown. Generally, *Orfelia* species are web-spinners chiefly associated with dead wood (Jakovlev 2012).

### Orfelia pallida

(Stæger, 1840)**

http://www.faunaeur.org/full_results.php?id=138560

#### Materials

- **Type status:** Other material. **Occurrence:** recordedBy: A. Polevoi; individualCount: 6; sex: 5 males, 1 female; **Location:** country: Russia; stateProvince: Republic Karelia; verbatimLocality: Obzha, Mayachino; decimalLatitude: 60.777; decimalLongitude: 32.818; geodeticDatum: WGS84; **Identification:** identifiedBy: A. Polevoi; **Event:** samplingProtocol: Malaise trap; eventDate: 2012-6-22/28; **Record Level:** institutionCode: FRIP
- **Type status:** Other material. **Occurrence:** recordedBy: A. Polevoi; individualCount: 2; sex: male; **Location:** country: Russia; stateProvince: Republic Karelia; verbatimLocality: Mikhailovskoe, Novikovo; decimalLatitude: 61.096; decimalLongitude: 33.755; geodeticDatum: WGS84; **Identification:** identifiedBy: A. Polevoi; **Event:** samplingProtocol: Malaise trap; eventDate: 2008-7-1/3; **Record Level:** institutionCode: FRIP
- **Type status:** Other material. **Occurrence:** recordedBy: A. Polevoi; individualCount: 3; sex: male; **Location:** country: Russia; stateProvince: Leningrad province; verbatimLocality: Podporozhje, Peldozhi; decimalLatitude: 61.027; decimalLongitude: 34.488; geodeticDatum: WGS84; **Identification:** identifiedBy: A. Polevoi; **Event:** samplingProtocol: Yellow pan trap; eventDate: 2008-7-3/4; **Record Level:** institutionCode: FRIP
- **Type status:** Other material. **Occurrence:** recordedBy: J.Jakovlev; individualCount: 1; sex: male; **Location:** country: Finland; stateProvince: Ab; municipality: Tammisaari; locality: Dragsvikin kartano; decimalLatitude: 60.000; decimalLongitude: 23.492; geodeticDatum: WGS84; **Identification:** identifiedBy: J.Jakovlev; **Event:** samplingProtocol: Malaise trap; eventDate: 2010-6-18/7-14; habitat: herb-rich forest and wet meadow, spruce-dominated, partly dominated with Alnus glutinosa. Huge willows; **Record Level:** institutionCode: JJH

#### Distribution

European, mainly nemoral, recorded from Ireland, Britain, the Netherlands, Belgium, Germany, Poland, Czech Republic and Estonia (Chandler 2004). Few records are known from the Nordic region, from Denmark (Petersen and Meier 2001), Sweden (only a single record, Kjaerandsen et al. 2007), and from Norway, without indication of the collecting locality (Rindal et al. 2008). In Finland the presence of the species was based on Hackman’s (Hackman 1980) checklist. However, the original sources of that record are unknown. In fact, the only exemplar in the MZHF collection “Diptera Fennica” identified as *Orfelia pallida* actually belongs to *Macrorrhyncha rostrata* Zetterstedt. New for the Republic of Karelia.

#### Ecology

The Finnish record is from a herb-rich, spruce dominated forest site adjacent to a sea gulf and a wet meadow with moist black alder stands. Huge willows (*Salix*) and plenty of dead wood are present as well. Karelian records are from herb-rich deciduous forests and a black alder fen. Immature stages are unknown.

#### Conservation

Due to the ambiguous occurrence data, the species was not included in the 2010 Red List of Finnish species. However, it is likely that *Orfelia pallida* is very rare in Finland and perhaps confined to hemiboreal deciduous forests.

### Monocentrota lundstromi

Edwards, 1925**

http://www.faunaeur.org/full_results.php?id=138574

#### Materials

- **Type status:** Other material. **Occurrence:** catalogNumber: MYCE-NV-2013-0058; recordedBy: J. Salmela; individualCount: 1; sex: male; **Location:** country: Finland; stateProvince: Lapponia kemensis pars occidentalis; verbatimLocality: Kittilä, Silmäsvuoma; decimalLatitude: 67.582; decimalLongitude: 25.543; geodeticDatum: WGS84; **Identification:** identifiedBy: J. Salmela; N. Vartija; **Event:** samplingProtocol: Malaise trap; eventDate: 2007-6-26/7-27; habitat: rich fen, aapamire; **Record Level:** institutionCode: JES
- **Type status:** Other material. **Occurrence:** recordedBy: A. Polevoi; individualCount: 1; sex: male; **Location:** country: Russia; stateProvince: Republic Karelia; verbatimLocality: Lahdenpohja, Niva; decimalLatitude: 61.615; decimalLongitude: 30.276; geodeticDatum: WGS84; **Identification:** identifiedBy: A. Polevoi; **Event:** samplingProtocol: Malaise trap; eventDate: 2005-7-7/8; **Record Level:** institutionCode: FRIP
- **Type status:** Other material. **Occurrence:** recordedBy: A. Polevoi; individualCount: 1; sex: male; **Location:** country: Russia; stateProvince: Republic Karelia; verbatimLocality: Zaozer’e, Pin’guba; decimalLatitude: 61.864; decimalLongitude: 34.558; geodeticDatum: WGS84; **Identification:** identifiedBy: A. Polevoi; **Event:** samplingProtocol: Yellow pan trap; eventDate: 2011-6-29/7-3; **Record Level:** institutionCode: FRIP
- **Type status:** Other material. **Occurrence:** recordedBy: A. Polevoi; individualCount: 2; sex: male; **Location:** country: Russia; stateProvince: Republic Karelia; verbatimLocality: Gomsel'ga; decimalLatitude: 62.059; decimalLongitude: 33.995; geodeticDatum: WGS84; **Identification:** identifiedBy: A. Polevoi; **Event:** samplingProtocol: Yellow pan trap; eventDate: 2012-7-4/6; **Record Level:** institutionCode: FRIP
- **Type status:** Other material. **Occurrence:** recordedBy: W.Hackman; individualCount: 1; sex: female; **Location:** country: Finland; stateProvince: Nylandia; municipality: Espoo; locality: Espoo; decimalLatitude: 60.210; decimalLongitude: 24.652; geodeticDatum: WGS84; **Identification:** identifiedBy: W.Hackman; **Event:** eventDate: 1962; habitat: herb-rich forest; **Record Level:** institutionCode: MZHF
- **Type status:** Other material. **Occurrence:** recordedBy: L.Tiensuu; individualCount: 1; sex: female; **Location:** country: Finland; stateProvince: Karelia ladogensis; municipality: Virolahti; locality: Virolahti; decimalLatitude: 60.579; decimalLongitude: 27.708; geodeticDatum: WGS84; **Identification:** identifiedBy: W.Hackman; **Event:** eventDate: 1971; **Record Level:** institutionCode: MZHF
- **Type status:** Other material. **Occurrence:** recordedBy: J.Jakovlev and G.Ståhls; individualCount: 2; sex: 1 male, 1 female; **Location:** country: Finland; stateProvince: Regio aboënsis; municipality: Turku; locality: Ruissalo; decimalLatitude: 60.432; decimalLongitude: 22.165; geodeticDatum: WGS84; **Identification:** identifiedBy: J.Jakovlev; **Event:** samplingProtocol: Malaise trap; eventDate: 2005-7-13/8-27; habitat: old-growth forest, herb-rich type; **Record Level:** institutionCode: JJH
- **Type status:** Other material. **Occurrence:** recordedBy: J.Jakovlev; individualCount: 1; sex: male; **Location:** country: Finland; stateProvince: Nylandia; municipality: Helsinki; locality: Tullisaari, Stansvikin kartano; decimalLatitude: 60.166; decimalLongitude: 25.027; geodeticDatum: WGS84; **Identification:** identifiedBy: J.Jakovlev; **Event:** samplingProtocol: Malaise trap; eventDate: 2011-6-12/8-2; habitat: City park with numerous old hollow deciduous trees, mainly lime trees, oaks and maples; **Record Level:** institutionCode: JJH
- **Type status:** Other material. **Occurrence:** recordedBy: J.Jakovlev; individualCount: 1; sex: male; **Location:** country: Finland; stateProvince: Nylandia; municipality: Helsinki; locality: Tuomarinkylä; decimalLatitude: 60.261; decimalLongitude: 24.965; geodeticDatum: WGS84; **Identification:** identifiedBy: J.Jakovlev; **Event:** samplingProtocol: Malaise trap; eventDate: 2011-7-5/7-20; habitat: Wood-storage areas in Helsinki; **Record Level:** institutionCode: JJH

#### Distribution

**Figure 4.**
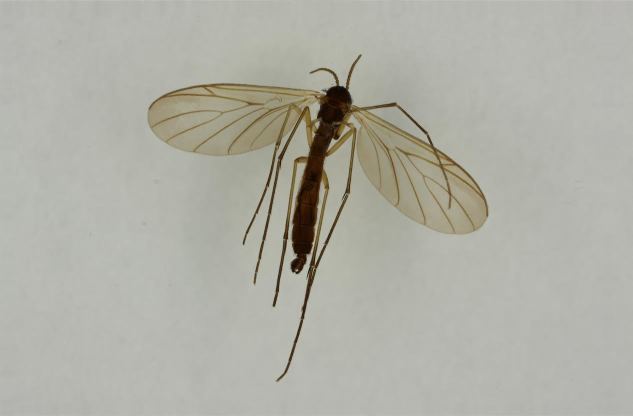
*Monocentrota lundstromi* Edwards, male specimen collected from Finland, dorsal view.

European. *Monocentrota lundstromi* (Fig. 4) is rare and poorly known in Fennoscandia. In Norway it is known from two localities in western part of the country (Kjaerandsen and Jordal 2007) and in Sweden it has been collected from a single locality in the zone of boreonemoral (or hemiboreal) forests (Kjaerandsen et al. 2007). No former records from the Republic of Karelia. Finnish records are from southern, eastern and northern parts of the country.

#### Ecology

The collecting locality in Kittilä is a large aapamire (see Fig. 5b for a definition of aapamire), dominated by rich fen vegetation. There are some small birch (*Betula pubescens*) trees growing on the mire, but the landscape is otherwise open. The specimens from the Republic Karelia have been collected from populated areas and clearcuts. Often attracted to light, active around dawn (Hutson et al. 1980). Larvae probably develop in decaying wood. The only rearing record is from southern Sweden: adults were collected with an eclector trap over a piece of oak log (Mats Jonsell leg., Jevgeni Jakovlev det. 2005).

#### Conservation

Red-listed in Finland (NT, Penttinen et al. 2010) and Norway (VU, Anonymous 2010, Gammelmo et al. 2010).

### Pyratula subcanariae

Chandler & Blasco-Zumeta, 2001*

http://www.faunaeur.org/full_results.php?id=138526

#### Materials

- **Type status:** Other material. **Occurrence:** catalogNumber: MYCE-JS-2013-0197; recordedBy: J. Salmela; individualCount: 2; sex: male; **Location:** country: Finland; stateProvince: Lapponia kemensis pars orientalis; verbatimLocality: Savukoski, Joutenoja; decimalLatitude: 67.821; decimalLongitude: 29.440; geodeticDatum: WGS84; **Identification:** identifiedBy: J. Salmela; **Event:** samplingProtocol: Malaise trap; eventDate: 2012-8-16/9-18; habitat: headwater stream, seminatural boreal forest; **Record Level:** institutionCode: JES
- **Type status:** Other material. **Occurrence:** catalogNumber: MYCE-JS-2013-0155; recordedBy: J. Salmela; individualCount: 1; sex: male; **Location:** country: Finland; stateProvince: Lapponia kemensis pars orientalis; verbatimLocality: Savukoski, Törmäoja; decimalLatitude: 67.846; decimalLongitude: 29.471; geodeticDatum: WGS84; **Identification:** identifiedBy: J. Salmela; **Event:** samplingProtocol: Malaise trap; eventDate: 2012-8-16/9-18; habitat: headwater stream, old-growth boreal forest; **Record Level:** institutionCode: JES
- **Type status:** Other material. **Occurrence:** catalogNumber: MYCE-JS-2013-0192; recordedBy: J. Salmela; individualCount: 1; sex: male; **Location:** country: Finland; stateProvince: Lapponia kemensis pars orientalis; verbatimLocality: Savukoski, Törmäoja; decimalLatitude: 67.846; decimalLongitude: 29.471; geodeticDatum: WGS84; **Identification:** identifiedBy: J. Salmela; **Event:** samplingProtocol: Malaise trap; eventDate: 2012-7-10/8-16; **Record Level:** institutionCode: JES
- **Type status:** Other material. **Occurrence:** catalogNumber: MYCE-JS-2013-0260; recordedBy: J. Salmela; individualCount: 1; sex: male; **Location:** country: Finland; stateProvince: Lapponia kemensis pars orientalis; verbatimLocality: Savukoski, Törmäoja, Ahot; decimalLatitude: 67.816; decimalLongitude: 29.426; geodeticDatum: WGS84; **Identification:** identifiedBy: J. Salmela; **Event:** samplingProtocol: Sweep net; eventDate: 2013-8-7; habitat: natural meadow; **Record Level:** institutionCode: JES
- **Type status:** Other material. **Occurrence:** catalogNumber: MYCE-JS-2013-0363; recordedBy: J. Salmela, T. Hietajärvi; individualCount: 1; sex: male; **Location:** country: Finland; stateProvince: Regio kuusamoensis; verbatimLocality: Salla, Värriö, Kuntasjoki; verbatimElevation: 330 m; decimalLatitude: 67.749; decimalLongitude: 29.616; geodeticDatum: WGS84; **Identification:** identifiedBy: J. Salmela; **Event:** samplingProtocol: Malaise trap; eventDate: 2013-6-29/7-29; habitat: headwater stream, old-growth forest; **Record Level:** institutionCode: JES

#### Distribution

European. Very poorly known species, described from Switzerland, Leuk (Chandler and Blasco-Zumeta 2001) and later reported from Bulgaria, eastern Danubian plain (Bechev 2006). The collecting site of the holotype is apparently mountainous whereas the Bulgarian site is only 150 m above sea level. Also collected from northern Sweden (Kjaerandsen 2012, J. Kjaerandsen, pers. comm.).

#### Ecology

Finnish localities are headwater streams with luxuriant riparian vegetation surrounded by coniferous forests. One of the sites (Törmäoja, Ahot) is a treeless, sloping meadow with short herbs and grasses on a moraine soil. Immature stages are unknown. The related species, *Pyratula zonata* has been collected with eclector traps on ground vegetation, moss carpets, and mineral soil under root plate of wind felled tree (Økland 1999) and on decaying trunks of aspen and goat willow, *Salix caprea* (J. Jakovlev, unpublished).

### Urytalpa atriceps

(Edwards, 1913)*

http://www.faunaeur.org/full_results.php?id=138508

#### Materials

- **Type status:** Other material. **Occurrence:** recordedBy: J.Penttinen; individualCount: 1; sex: male; **Location:** country: Finland; stateProvince: Savonia australis; municipality: Rantasalmi; locality: Linnansaari; decimalLatitude: 62.116; decimalLongitude: 28.477; geodeticDatum: WGS84; **Identification:** identifiedBy: J.Penttinen; **Event:** samplingProtocol: Malaise trap; eventDate: 2008-7-25/9-4; habitat: old-growth forest, herb-rich type; **Record Level:** institutionCode: JPJ
- **Type status:** Other material. **Occurrence:** catalogNumber: DIPT-JS-2014-0036; recordedBy: J. Salmela; T. Hietajärvi; individualCount: 1; sex: male; **Location:** country: Finland; stateProvince: Regio kuusamoensis; verbatimLocality: Salla, Kuntasjoki, Värriö Strict Nature Reserve; verbatimElevation: 320 m; decimalLatitude: 67.749; decimalLongitude: 29.617; geodeticDatum: WGS84; **Identification:** identifiedBy: J. Salmela; **Event:** samplingProtocol: Malaise trap; eventDate: 2013; verbatimEventDate: 2013-6-29/7-29; habitat: headwater stream, old-growth boreal forest; **Record Level:** institutionCode: JES

#### Distribution

European. Known from England, the Netherlands, Norway and Sweden (Kjaerandsen et al. 2009). Reported here formally as a new species for Finland.

#### Ecology

Immature stages are unknown. Linnansaari (south boreal zone) is a lush semi-dry herb-rich forest with human influence (most likely former slash-and-burn forest) where aspen is in many parts the dominant tree species with lime, birch and spruce. Collecting site in Salla (north boreal zone) is a luxuriant headwater stream with swampy margings, surrounded by pristine spruce forest.

#### Conservation

Red-listed in Finland (DD, Penttinen et al. 2010) and Sweden (NT, Cederberg et al. 2010).

### Urytalpa galdes

Hedmark & Kjaerandsen, 2009*

#### Materials

- **Type status:** Other material. **Occurrence:** recordedBy: J.Jakovlev and J.Penttinen; individualCount: 1; sex: male; **Location:** country: Finland; stateProvince: Lapponia kemensis pars occidentalis; municipality: Muonio; locality: Pallas-Yllästunturi National Park; decimalLatitude: 68.018; decimalLongitude: 24.153; geodeticDatum: WGS84; **Identification:** identifiedBy: J.Jakovlev; **Event:** samplingProtocol: Malaise trap; eventDate: 2006-7-15/8-14; habitat: old-growth forest, Myrtillus type
- **Type status:** Other material. **Occurrence:** catalogNumber: DIPT-JS-2014-0033; recordedBy: J. Salmela; T. Hietajärvi; individualCount: 1; sex: male; **Location:** country: Finland; stateProvince: Regio kuusamoensis; verbatimLocality: Salla, Kuntasjoki, Värriö Strict Nature Reserve; verbatimElevation: 320 m; decimalLatitude: 67.749; decimalLongitude: 29.617; geodeticDatum: WGS84; **Identification:** identifiedBy: J. Salmela; **Event:** samplingProtocol: Malaise trap; eventDate: 2013; verbatimEventDate: 2013-6-29/7-29; habitat: headwater stream, old-growth boreal forest; **Record Level:** institutionCode: JES

#### Distribution

Fennoscandian. The species is previously known only from the type locality in northern Sweden (Lule Lapmark, Kjaerandsen et al. 2009). The first record from Finland.

#### Ecology

Nothing is known of the life histories of *Urytalpa* spp.; they may be similar to those of *Pyratula* spp. Finnish collecting sites are old-growth boreal forests.

### Urytalpa macrocera

(Edwards, 1913)*

http://www.faunaeur.org/full_results.php?id=138509

#### Materials

- **Type status:** Other material. **Occurrence:** recordedBy: J.Salmela; individualCount: 1; sex: male; **Location:** country: Finland; stateProvince: Lapponia inarensis; municipality: Utsjoki; locality: Galddasjohka; decimalLatitude: 69.860; decimalLongitude: 27.770; geodeticDatum: WGS84; **Identification:** identifiedBy: J.Jakovlev; **Event:** samplingProtocol: Malaise trap; eventDate: 2007-7-19/8-27; habitat: subarctic stream valley; **Record Level:** institutionCode: JJH
- **Type status:** Other material. **Occurrence:** recordedBy: J.Salmela; individualCount: 1; sex: male; **Location:** country: Finland; stateProvince: Lapponia inarensis; municipality: Utsjoki; locality: Galddasjohka; decimalLatitude: 69.861; decimalLongitude: 27.790; geodeticDatum: WGS84; **Identification:** identifiedBy: J.Jakovlev; **Event:** samplingProtocol: Malaise trap; eventDate: 2007-7-19/8-27; habitat: subarctic stream valley; **Record Level:** institutionCode: JJH

#### Distribution

European, known only in northern Britain (Scotland and northern England), France, Norway, Sweden and the Netherlands (Kjaerandsen et al. 2009). No former records from Finland.

#### Ecology

Finnish localities are two close lying trapping sites in a river valley surrounded by a strip of mountain birch forest in the northernmost Lapland (Fig. 5e). From Great Britain recorded from moist deciduous forest (Falk and Chandler 2005). Immature stages are unknown.

### Macrocera crassicornis

Winnertz, 1864*

http://www.faunaeur.org/full_results.php?id=138434

#### Materials

- **Type status:** Other material. **Occurrence:** recordedBy: J.Jakovlev and J.Penttinen; individualCount: 1; sex: male; **Location:** country: Finland; stateProvince: Tavastia australis; municipality: Lammi; locality: Evo_Kotinen_Aspen part; decimalLatitude: 61.244; decimalLongitude: 25.067; geodeticDatum: WGS84; **Identification:** identifiedBy: J.Jakovlev; **Event:** samplingProtocol: Reared from soil; eventDate: 2006-8-3/8-30; habitat: old-growth forest, herb-rich type; **Record Level:** institutionCode: JJH
- **Type status:** Other material. **Occurrence:** recordedBy: J.Jakovlev and J.Penttinen; individualCount: 1; sex: male; **Location:** country: Finland; stateProvince: Tavastia borealis; municipality: Saarijärvi; locality: Pyhä-Häkki National Park; decimalLatitude: 62.836; decimalLongitude: 25.473; geodeticDatum: WGS84; **Identification:** identifiedBy: J.Jakovlev; **Event:** samplingProtocol: Reared from dead spruce log; eventDate: 2006-8-3/8-30; habitat: old-growth forest, Myrtillus type; **Record Level:** institutionCode: JJH
- **Type status:** Other material. **Occurrence:** recordedBy: J.Jakovlev and J.Penttinen; individualCount: 1; sex: male; **Location:** country: Finland; stateProvince: Tavastia borealis; municipality: Saarijärvi; locality: Pyhä-Häkki National Park; decimalLatitude: 62.836; decimalLongitude: 25.473; geodeticDatum: WGS84; **Identification:** identifiedBy: J.Jakovlev; **Event:** samplingProtocol: Reared from dead pine wood; eventDate: 2006-8-3/8-30; habitat: old-growth forest, Myrtillus type; **Record Level:** institutionCode: JJH

#### Distribution

**Figure 6.**
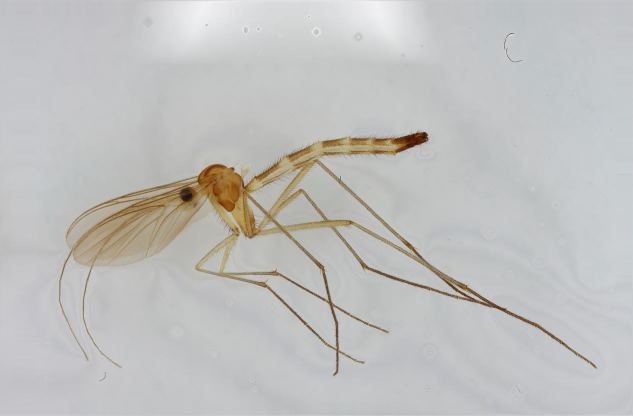
*Macrocera crassicornis* Meigen, male specimen collected from Finland, lateral view.

Palaearctic. *Macrocera crassicornis* (Fig. 6) has a rather wide range in central and southern Europe, also known from Near East and North Africa (Chandler 2004, Zaitzev 1994). Included in the Finnish Red List assessment (Penttinen et al. 2010), here formally reported as new for Finland.

#### Ecology

Reared from soil and dead coniferous wood. Finnish collecting sites are old-growth spruce dominated forests situated in the south boreal vegetation zone.

#### Conservation

Threatened fungus gnat species in Finland (VU, Penttinen et al. 2010).

### Macrocera fascipennis

Staeger, 1840

http://www.faunaeur.org/full_results.php?id=138442

#### Materials

- **Type status:** Other material. **Occurrence:** catalogNumber: MYCE-JS-2012-0010; recordedBy: J. Salmela; individualCount: 1; sex: male; **Location:** country: Finland; stateProvince: Lapponia kemensis pars orientalis; verbatimLocality: Sodankylä, Pomokaira, Kaita-aapa; decimalLatitude: 67.845; decimalLongitude: 26.553; geodeticDatum: WGS84; **Identification:** identifiedBy: J. Salmela, A. Polevoi; **Event:** samplingProtocol: Malaise trap; eventDate: 2012-7-4/8-7; habitat: intermediate rich fen, aapamire; **Record Level:** institutionCode: JES
- **Type status:** Other material. **Occurrence:** recordedBy: M.Jaschhof and C.Jaschhof; individualCount: 1; sex: male; **Location:** country: Finland; stateProvince: Karelia borealis; municipality: Ilomantsi; locality: Pirhu_1; decimalLatitude: 62.971; decimalLongitude: 31.395; geodeticDatum: WGS84; **Identification:** identifiedBy: J.Jakovlev; **Event:** samplingProtocol: Sweep netting; eventDate: 2004-7-8/7-8; habitat: young unmanaged forest; **Record Level:** institutionCode: JJH
- **Type status:** Other material. **Occurrence:** recordedBy: J.Sahlberg; individualCount: 1; sex: male; **Location:** country: Finland; stateProvince: Regio aboënsis; municipality: Karjalohja; locality: Karjalohja_unknown_locality; decimalLatitude: 60.245; decimalLongitude: 23.746; geodeticDatum: WGS84; **Identification:** identifiedBy: C.Lundström; **Event:** eventDate: 1900; **Record Level:** institutionCode: MZHF
- **Type status:** Other material. **Occurrence:** recordedBy: R.Frey; individualCount: 1; sex: female; **Location:** country: Finland; stateProvince: Tavastia australis; municipality: Tammela?; locality: Mäntyharju; decimalLatitude: 61.968; decimalLongitude: 23.697; geodeticDatum: WGS84; **Identification:** identifiedBy: C.Lundström; **Event:** samplingProtocol: Sweep netting; eventDate: 1900; **Record Level:** institutionCode: MZHF
- **Type status:** Other material. **Occurrence:** recordedBy: R.Tuomikoski; individualCount: 1; sex: male; **Location:** country: Finland; stateProvince: Tavastia australis; municipality: Lammi; locality: Evo; decimalLatitude: 61.196; decimalLongitude: 25.099; geodeticDatum: WGS84; **Identification:** identifiedBy: W.Hackman; **Event:** samplingProtocol: Sweep netting; eventDate: 1953; **Record Level:** institutionCode: MZHF
- **Type status:** Other material. **Occurrence:** recordedBy: R.Tuomikoski; individualCount: 1; sex: female; **Location:** country: Finland; stateProvince: Regio aboensis; municipality: Vihti; locality: Vihtijärvi; decimalLatitude: 60.523; decimalLongitude: 24.556; geodeticDatum: WGS84; **Identification:** identifiedBy: W.Hackman; **Event:** samplingProtocol: Sweep netting; eventDate: 1962-7-29/-7-29; **Record Level:** institutionCode: MZHF

#### Distribution

Palaearctic. In Europe recorded from the British Isles, Central and northern Europe (Chandler 2004). In Fennoscandia recorded from Finland, Russian Karelia (Lundström 1906) and Murmansk region (Lundström 1909), also known from Denmark. Hitherto collected only from a few Finnish localities in southern (Lundström 1906) and eastern Finland (Polevoi 2001a).

#### Ecology

Reared from a tussock of *Scirpus sylvaticus* in Czech Republic (Ševčík and Roháček 2008). The locality in Sodankylä, Kaita-aapa, is a large, open aapamire with intermediate rich vegetation. *Scirpus sylvaticus* was not present, but several other Cyperaceae species were abundant in the mire (*Carex* spp., *Trichophorum* spp., *Eriophorum* spp.).

### Macrocera grandis

Lundström, 1912**

http://www.faunaeur.org/full_results.php?id=138446

#### Materials

- **Type status:** Other material. **Occurrence:** recordedBy: A. Polevoi; individualCount: 1; sex: male; **Location:** country: Russia; stateProvince: Republic Karelia; verbatimLocality: Obzha, Tabanovskyi mayak; decimalLatitude: 60.757; decimalLongitude: 32.815; geodeticDatum: WGS84; **Identification:** identifiedBy: A. Polevoi; **Event:** samplingProtocol: Sweep netting; eventDate: 2012-6-25; **Record Level:** institutionCode: FRIP
- **Type status:** Other material. **Occurrence:** recordedBy: R.Tuomikoski; individualCount: 1; sex: male; **Location:** country: Finland; stateProvince: Satakunta; municipality: Kokemäki; locality: Kokemäki; decimalLatitude: 61.254; decimalLongitude: 22.317; geodeticDatum: WGS84; **Identification:** identifiedBy: W.Hackman; **Event:** samplingProtocol: Sweep netting; eventDate: 1953; **Record Level:** institutionCode: MZHF
- **Type status:** Other material. **Occurrence:** recordedBy: W.Hackman; individualCount: 1; sex: male; **Location:** country: Finland; stateProvince: Nylandia; municipality: Espoo; locality: Kolmperä; decimalLatitude: 60.244; decimalLongitude: 24.523; geodeticDatum: WGS84; **Identification:** identifiedBy: J.R.Vockeroth; **Event:** eventDate: 1964-7-21/7-21; **Record Level:** institutionCode: MZHF
- **Type status:** Other material. **Occurrence:** recordedBy: J.Salmela; individualCount: 1; sex: male; **Location:** country: Finland; stateProvince: Tavastia borealis; municipality: Äänekoski; locality: Kivipuro; decimalLatitude: 62.563; decimalLongitude: 25.511; geodeticDatum: WGS84; **Identification:** identifiedBy: J.Penttinen; **Event:** samplingProtocol: sweep net; eventDate: 2008; habitat: spruce mire along a creek, surrounded by managed forest; **Record Level:** institutionCode: JPJ

#### Distribution

Palaearctic. Described from Finland (Lundström 1912a), later recorded from Ural and Altai (Zaitzev 1994) and from Europe, chiefly from northern areas: Norway, Sweden, Finland, NW Russia, Baltic countries, Germany (Chandler 2004, Kjaerandsen et al. 2007). New to the Republic of Karelia.

#### Ecology

The species is very rare. There are only four Finnish records, one of these was made more than a hundred years ago (Lundström 1912b, SW Finland, Kaarina, Kuusisto). The only recent finding is from a headwater stream surrounded by a spruce mire (Central Finland, Äänekoski). The Karelian specimen was collected at the edge of a small settlement near a herb-rich aspen dominated forest. Immature stages are unknown, other species of the genus have been reared from soil, clumps of turf, rotting wood and cave walls and are considered predaceous (Falk and Chandler 2005, Ševčík and Roháček 2008, Jakovlev 2012).

#### Conservation

Red-listed in Finland (NT, Penttinen et al. 2010) and Norway (VU, Anonymous 2010, Gammelmo et al. 2010).

### Bolitophila (Cliopisa) ingrica

Stackelberg, 1969*

http://www.faunaeur.org/full_results.php?id=129995

#### Materials

- **Type status:** Other material. **Occurrence:** recordedBy: J. Jakovlev; J. Penttinen; individualCount: 1; sex: male; **Location:** country: Finland; stateProvince: Lapponia enontekiensis; verbatimLocality: Kilpisjärvi, Saana Mt, southern slope; decimalLatitude: 69.0456; decimalLongitude: 20.8186; geodeticDatum: WGS84; **Event:** samplingProtocol: Malaise trap; eventDate: 2006-8-1/15; **Record Level:** institutionCode: JJH
- **Type status:** Other material. **Occurrence:** recordedBy: J. Jakovlev; J. Penttinen; individualCount: 1; sex: male; **Location:** country: Finland; stateProvince: Lapponia enontekiensis; verbatimLocality: Kilpisjärvi, Saana Mt, southern slope; decimalLatitude: 69.0456; decimalLongitude: 20.8186; geodeticDatum: WGS84; **Event:** samplingProtocol: Malaise trap; eventDate: 2006-8-16/31; **Record Level:** institutionCode: JJH
- **Type status:** Other material. **Occurrence:** recordedBy: J. Jakovlev; individualCount: 2; sex: male; **Location:** country: Finland; stateProvince: Lapponia enontekiensis; verbatimLocality: Kilpisjärvi, Saana Mt, southern slope; decimalLatitude: 69.0456; decimalLongitude: 20.8186; geodeticDatum: WGS84; **Event:** samplingProtocol: sweep net; eventDate: 2006-8-20/21; **Record Level:** institutionCode: JJH
- **Type status:** Other material. **Occurrence:** recordedBy: J. Penttinen; individualCount: 1; sex: male; **Location:** country: Finland; stateProvince: Savonia australis; verbatimLocality: Rantasalmi, Linnansaari; decimalLatitude: 62.116; decimalLongitude: 28.476; geodeticDatum: WGS84; **Identification:** identifiedBy: J. Penttinen; **Event:** samplingProtocol: Malaise trap; eventDate: 2008-5-20/6-24; **Record Level:** institutionCode: JPJ

#### Distribution

European. A rare species described from Leningrad Region in northwest Russia (Ostroverkhova and Stackelberg 1988) and later found in a few places in Central Europe: Germany, Slovakia, Switzerland (Chandler 2004), and from Vologda, Kostroma and Moscow Regions of northwest and central Russia (Zaitzev 1994). In Fennoscandia found from the Republic of Karelia (Polevoi 2000), Sweden (Kjaerandsen et al. 2007) and Norway (Kjaerandsen and Jordal 2007). Included in the recent Finnish Red List, although here formally reported as a new species for the Finnish fauna.

#### Ecology

Finnish collecting localities are a herb-rich coniferous forest with a large proportion of birch and aspen in the southeastern Finland (Rantasalmi) and a sub-arctic mountain birch (Betula pubescens ssp. czerepanovii) forest with herb-rich vegetation in the slopes of Saana Mountain in northwestern Lapland. Immature stages are unknown. Generally, *Bolitophila* larvae develop inside soft fungi (Jakovlev 2012).

#### Conservation

Red-listed in Finland (NT, Penttinen et al. 2010).

### Mycomya (Coheromyia) branderi

Väisänen, 1984

http://www.faunaeur.org/full_results.php?id=139509

#### Materials

- **Type status:** Other material. **Occurrence:** catalogNumber: MYCE-JS-2013-0333; recordedBy: Jari Ilmonen; individualCount: 10; sex: male; **Location:** country: Finland; stateProvince: Nylandia; verbatimLocality: Espoo, Matalajärvi; decimalLatitude: 60.247; decimalLongitude: 24.686; geodeticDatum: WGS84; **Identification:** identifiedBy: J. Salmela; **Event:** samplingProtocol: Malaise trap; eventDate: 2012-7-21/8-23; habitat: swampy lake shore; **Record Level:** institutionCode: JES
- **Type status:** Other material. **Occurrence:** catalogNumber: MYCE-JS-2013-0280; recordedBy: Jari Ilmonen; individualCount: 12; sex: 10 males, 2 females; **Location:** country: Finland; stateProvince: Nylandia; verbatimLocality: Espoo, Matalajärvi; decimalLatitude: 60.247; decimalLongitude: 24.686; geodeticDatum: WGS84; **Identification:** identifiedBy: J. Salmela; **Event:** samplingProtocol: Malaise trap; eventDate: 2012-8-23/10-20; **Record Level:** institutionCode: JES
- **Type status:** Other material. **Occurrence:** recordedBy: M. Jaschhof; C. Jaschhof; individualCount: 1; sex: male; **Location:** country: Finland; stateProvince: Karelia ladogensis; verbatimLocality: Parikkala, Siikalahti; decimalLatitude: 61.562; decimalLongitude: 29.599; geodeticDatum: WGS84; **Identification:** identifiedBy: J. Jakovlev; **Event:** samplingProtocol: Sweep net; eventDate: 2004-8-19; habitat: swampy forest; **Record Level:** institutionCode: JJH

#### Distribution

European. Very rare and poorly known species, so far recorded from South Finland, Denmark and Great Britain (Väisänen 1984, Chandler 1992).

#### Ecology

*Mycomya branderi* is most likely associated with wetlands. The British records are from wetlands (Chandler 1992) and two recent Finnish localities are a swampy lake shore (Espoo) and a birch/alder swamp forest on a lake shore (Parikkala). Immature stages are unknown.

#### Conservation

Red-listed in Finland (VU, Penttinen et al. 2010).

### Mycomya (Mycomya) britteni

Kidd, 1955

http://www.faunaeur.org/full_results.php?id=139525

#### Materials

- **Type status:** Other material. **Occurrence:** catalogNumber: MYCE-JS-2013-0337; recordedBy: Jari Ilmonen; individualCount: 1; sex: male; **Location:** country: Finland; stateProvince: Nylandia; verbatimLocality: Espoo, Matalajärvi; decimalLatitude: 60.246; decimalLongitude: 24.687; geodeticDatum: WGS84; **Identification:** identifiedBy: J. Salmela; **Event:** samplingProtocol: Malaise trap; eventDate: 2012-7-21/8-23; habitat: swampy lake shore; **Record Level:** institutionCode: JES

#### Distribution

European. Very rare species, hitherto recorded only from Great Britain, Finland (Väisänen 1984, Karjalohja, South Finland) Norway (Gammelmo and Søli 2006), Sweden (Kjaerandsen 2012) and Czech Republic (Ševčík and Roháček 2008).

#### Ecology

Mostly likely a wetland-dwelling species. Reared from sedge (*Carex*) tussocks in Czech Republic (Ševčík and Roháček 2008). The Finnish locality is a swampy lake shore, and the only Norwegian record is from a lake shore wetland (Anonymous 2010). Immature stages are unknown.

#### Conservation

Red-listed in Norway (VU, Anonymous 2010, Gammelmo et al. 2010).

### Mycomya (Mycomya) collini

Edwards, 1941**

http://www.faunaeur.org/full_results.php?id=139535

#### Materials

- **Type status:** Other material. **Occurrence:** recordedBy: A. Polevoi; individualCount: 2; sex: male; **Location:** country: Russia; stateProvince: Republic Karelia; verbatimLocality: Onezhskoe lake, island Paleostrov; decimalLatitude: 62.569; decimalLongitude: 35.261; geodeticDatum: WGS84; **Identification:** identifiedBy: A. Polevoi; **Event:** samplingProtocol: Sweep netting; eventDate: 2004-7-3; **Record Level:** institutionCode: FRIP
- **Type status:** Other material. **Occurrence:** recordedBy: J. Jakovlev; individualCount: 1; sex: male; **Location:** country: Finland; stateProvince: Regio aboensis; verbatimLocality: Karjalohja, Karkali; decimalLatitude: 60.238; decimalLongitude: 23.785; geodeticDatum: WGS84; **Identification:** identifiedBy: J. Jakovlev; **Event:** samplingProtocol: Malaise trap; eventDate: 2004-6-16/8-23; habitat: old-growth forest, herb-rich type; **Record Level:** institutionCode: JJH
- **Type status:** Other material. **Occurrence:** recordedBy: J. Penttinen; individualCount: 1; sex: male; **Location:** country: Finland; stateProvince: Tavastia australis; verbatimLocality: Lahti, Mukkula; decimalLatitude: 61.017; decimalLongitude: 25.643; geodeticDatum: WGS84; **Identification:** identifiedBy: J. Penttinen; **Event:** samplingProtocol: Malaise trap; eventDate: 2009-6-15/8-15; habitat: old-growth forest, herb-rich type; **Record Level:** institutionCode: JPJ

#### Distribution

European. Rare species known by few records from Great Britain, Germany, Switzerland, Estonia, Norway and Finland (Edwards 1941, Väisänen 1984, Chandler 2004, Kjaerandsen and Jordal 2007), recently found also in Slovakia (Ševčík and Kurina 2011). Here reported for the first time from Russia. Only two previous collecting localities in South Finland (Väisänen 1984).

#### Ecology

New records from Russian Karelia (Paleostrov Island) and Finland (Lahti) are from herb-rich old-growth forests on fertile soil close to lake shores. Immature stages are unknown.

### Mycomya (Mycomya) fuscata

(Winnertz, 1863)

http://www.faunaeur.org/full_results.php?id=139549

#### Materials

- **Type status:** Other material. **Occurrence:** catalogNumber: MYCE-JS-2012-0069; recordedBy: J. Salmela; individualCount: 1; sex: male; **Location:** country: Finland; stateProvince: Lapponia kemensis pars orientalis; verbatimLocality: Savukoski, Joutenoja; decimalLatitude: 67.821; decimalLongitude: 29.440; geodeticDatum: WGS84; **Identification:** identifiedBy: J. Salmela; **Event:** samplingProtocol: Malaise trap; eventDate: 2012-8-16/9-18; habitat: headwater stream, seminatural boreal forest; **Record Level:** institutionCode: JES

#### Distribution

Holarctic. In Europe a boreo-montane species (Kjaerandsen et al. 2007). Fennoscandian records are from the northernmost parts of Norway (Søli and Rindal 2012), Sweden (Kjaerandsen et al. 2007) and Murmansk region (Väisänen 1984), from a total of four localities. Only one previous Finnish record from Kainuu, mid boreal vegetation zone (Polevoi et al. 2006).

#### Ecology

Poorly known species. Finnish collecting sites are an old-growth boreal forest (Kainuu, Polevoi et al. 2006) and a headwater stream with rich riparian vegetation, surrounded by a nearly pristine coniferous forest (Joutenoja). Immature stages are unknown.

### Mycomya (Mycomya) fornicata

(Lundström, 1911)**

http://www.faunaeur.org/full_results.php?id=139548

#### Materials

- **Type status:** Other material. **Occurrence:** recordedBy: A. Humala; individualCount: 1; sex: male; **Taxon:** genus: Mycomya; subgenus: Mycomya; specificEpithet: fornicata; scientificNameAuthorship: (Lundström, 1911); **Location:** country: Russia; stateProvince: Republic Karelia; verbatimLocality: White Sea, is. Kondostrov; decimalLatitude: 64.225; decimalLongitude: 36.621; geodeticDatum: WGS84; **Identification:** identifiedBy: A. Polevoi; **Event:** samplingProtocol: Sweep netting; eventDate: 08/21/2002; **Record Level:** institutionCode: FRIP

#### Distribution

Palaearctic. Rare species known by few records from the European Alps, Yakutia and Amur Province (Väisänen 1984, Zaitzev 1994, Krzemiñska and Klimont 2011). No former records from Fennoscandia; new to the Republic of Karelia.

#### Ecology

The Karelian specimen was collected in *Vaccinium myrtillus* type spruce dominated forest. Immature stages are unknown.

### Myomya (Mycomya) islandica

Väisänen, 1984**

http://www.faunaeur.org/full_results.php?id=139568

#### Materials

- **Type status:** Other material. **Occurrence:** recordedBy: A. Polevoi; individualCount: 2; sex: male; **Location:** country: Russia; stateProvince: Republic Karelia; verbatimLocality: Paanajärvi, 2 km W of Selkäjoki river mouth; decimalLatitude: 66.259; decimalLongitude: 29.968; geodeticDatum: WGS84; **Identification:** identifiedBy: A. Polevoi; **Event:** samplingProtocol: Sweep netting; eventDate: 2000-7-4; **Record Level:** institutionCode: FRIP

#### Distribution

Holarctic. Recorded from northern regions of Europe and North America (Väisänen 1984, Kjaerandsen et al. 2007). New to the Republic of Karelia.

#### Ecology

Collected in *Vaccinium myrtillus* type spruce dominated forest. Immature stages are unknown.

### Mycomya (Mycomya) lambi

Edwards, 1941

http://www.faunaeur.org/full_results.php?id=139572

#### Materials

- **Type status:** Other material. **Occurrence:** catalogNumber: MYCE-JS-2013-0066; recordedBy: J. Salmela; individualCount: 1; sex: male; **Location:** country: Finland; stateProvince: Lapponia kemensis pars orientalis; verbatimLocality: Savukoski, Joutenoja; decimalLatitude: 67.821; decimalLongitude: 29.440; geodeticDatum: WGS84; **Identification:** identifiedBy: J. Salmela; **Event:** samplingProtocol: Malaise trap; eventDate: 2012-8-16/9-18; habitat: headwater stream, seminatural boreal forest; **Record Level:** institutionCode: JES
- **Type status:** Other material. **Occurrence:** catalogNumber: MYCE-JS-2013-0152; recordedBy: J. Salmela; individualCount: 1; sex: male; **Location:** country: Finland; stateProvince: Lapponia kemensis pars orientalis; verbatimLocality: Savukoski, Törmäoja; decimalLatitude: 67.846; decimalLongitude: 29.471; geodeticDatum: WGS84; **Identification:** identifiedBy: J. Salmela; **Event:** samplingProtocol: Malaise trap; eventDate: 2012-8-16/9-18; **Record Level:** institutionCode: JES
- **Type status:** Other material. **Occurrence:** catalogNumber: MYCE-JS-2013-0261; recordedBy: J. Salmela; individualCount: 1; sex: male; **Location:** country: Finland; stateProvince: Lapponia kemensis pars orientalis; verbatimLocality: Savukoski, Törmäoja, Ahot; decimalLatitude: 67.816; decimalLongitude: 29.426; geodeticDatum: WGS84; **Identification:** identifiedBy: J. Salmela; **Event:** samplingProtocol: Sweep net; eventDate: 2013-8-7; **Record Level:** institutionCode: JES

#### Distribution

Holarctic. In Europe perhaps a boreo-montane species (Kjaerandsen et al. 2007), also recorded from the Faroes (Kjaerandsen and Jørgensen 1992) and from two coastal sites in Scotland (Falk and Chandler 2005). In Fennoscandia known from Finland, Norway and Sweden. Rather poorly known and rarely collected in Sweden (northern provinces JÄ and TO, Kjaerandsen et al. 2007). In Norway recorded from Finnmark, the northernmost part of the country (Søli and Rindal 2012). A few scattered Finnish records (Väisänen 1984), mainly from old-growth forests.

#### Ecology

New records from Savukoski are from headwater streams with rich riparian vegetation surrounded by coniferous forests. One of the sites (Törmäoja, Ahot) is a sloping meadow with short herbs and grasses on a moraine soil. Immature stages are unknown.

### Mycomya (Mycomya) safena

Väisänen, 1984*

http://www.catalogueoflife.org/col/details/species/id/8662701

#### Materials

- **Type status:** Other material. **Occurrence:** catalogNumber: MYCE-JS-2013-0383; recordedBy: J. Salmela; S. Lapinniemi; individualCount: 1; sex: male; **Location:** country: Finland; stateProvince: Lapponia enontekiensis; verbatimLocality: Pallas-Yllästunturi National Park, Röyninkuru; verbatimElevation: 380 m; decimalLatitude: 68.146; decimalLongitude: 24.071; geodeticDatum: WGS84; **Identification:** identifiedBy: J. Salmela; **Event:** samplingProtocol: Malaise trap; eventDate: 2013-6-5/7-6; habitat: headwater stream, old-growth spruce forest; **Record Level:** institutionCode: JES
- **Type status:** Other material. **Occurrence:** catalogNumber: DIPT-JS-2014-0041; recordedBy: J. Salmela; T. Hietajärvi; individualCount: 1; sex: male; **Location:** country: Finland; stateProvince: Regio kuusamoensis; verbatimLocality: Salla, Kuntasjoki, Värriö Strict Nature Reserve; verbatimElevation: 320 m; decimalLatitude: 67.749; decimalLongitude: 29.617; geodeticDatum: WGS84; **Identification:** identifiedBy: J. Salmela; **Event:** samplingProtocol: Malaise trap; verbatimEventDate: 2013-6-29/7-29; habitat: headwater stream, old-growth boreal forest; **Record Level:** institutionCode: JES

#### Distribution

Holarctic. Described from the USA and Canada (Väisänen 1984). Recently reported by Polevoi (Polevoi 2010) for the first time from the Palaearctic region (NW Russia, Murmansk region). New for Finland.

#### Ecology

The Finnish collecting sites are headwater streams surrounded by old-growth boreal, spruce dominated forests in Lapland. Immature stages are unknown.

### Mycomya (Mycomya) shewelli

Väisänen, 1984***

http://www.catalogueoflife.org/col/details/species/id/8662703

#### Materials

- **Type status:** Other material. **Occurrence:** catalogNumber: MYCE-JS-2013-0320; recordedBy: Jari Ilmonen; individualCount: 1; sex: male; **Location:** country: Finland; stateProvince: Nylandia; verbatimLocality: Espoo, Matalajärvi, SW shore; verbatimElevation: 23 m; decimalLatitude: 60.247; decimalLongitude: 24.686; geodeticDatum: WGS84; **Identification:** identifiedBy: J. Salmela; **Event:** samplingProtocol: Malaise trap; eventDate: 2012-7-21/8-23; habitat: swampy lake shore; **Record Level:** institutionCode: JES

#### Distribution

Holarctic. *Mycomya shewelli* (Fig. 7) was described from the Nearctic region (USA: Michigan, Canada: North West Territories, Manitoba, Väisänen 1984), with no previous records from the Palaearctic region. New for the European fauna.

#### Ecology

Poorly known species, immature stages are unknown. The Finnish collecting site is a diverse black alder (*Alnus glutinosa*) swamp in a lake shore (Espoo, South Finland).

### Mycomya (Mycomya) sieberti

Landrock, 1930*

http://www.faunaeur.org/full_results.php?id=139607

#### Materials

- **Type status:** Other material. **Occurrence:** recordedBy: M. Jaschhof; C. Jaschhof; individualCount: 7; sex: male; **Location:** country: Finland; stateProvince: Karelia ladogensis; verbatimLocality: Parikkala, Siikalahti; decimalLatitude: 61.562; decimalLongitude: 29.599; geodeticDatum: WGS84; **Identification:** identifiedBy: J. Jakovlev; **Event:** samplingProtocol: Sweep net; eventDate: 2004-6-8; habitat: old managed swampy forest; **Record Level:** institutionCode: JJH

#### Distribution

Palaearctic. A very rare species known so far only from Russia (Leningrad oblast and Russian Far East) and from Latvia (Väisänen 1984). No former records from other European countries (Chandler 2004). New to Finland.

#### Ecology

The only Finnish sampling site is an old, managed swampy forest in southern Finland. Immature stages are unknown.

### Mycomya (Mycomya) thula

Väisänen, 1984***

http://www.catalogueoflife.org/col/details/species/id/8662705

#### Materials

- **Type status:** Other material. **Occurrence:** catalogNumber: MYCE-JS-2012-0071; recordedBy: J. Salmela; individualCount: 3; sex: 2 males, 1 female; **Location:** country: Finland; stateProvince: Lapponia kemensis pars orientalis; verbatimLocality: Savukoski, Joutenoja; decimalLatitude: 67.821; decimalLongitude: 29.440; geodeticDatum: WGS84; **Identification:** identifiedBy: J. Salmela; A. Polevoi; **Event:** samplingProtocol: Malaise trap; eventDate: 2012-8-16/9-18; habitat: headwater stream, seminatural boreal forest; **Record Level:** institutionCode: JES
- **Type status:** Other material. **Occurrence:** catalogNumber: MYCE-JS-2012-0006; recordedBy: J. Salmela; individualCount: 1; sex: male; **Location:** country: Finland; stateProvince: Lapponia kemensis pars orientalis; verbatimLocality: Savukoski, Törmäoja; decimalLatitude: 67.846; decimalLongitude: 29.471; geodeticDatum: WGS84; **Identification:** identifiedBy: J. Salmela; **Event:** samplingProtocol: Malaise trap; eventDate: 2012-7-10/8-16; habitat: headwater stream, old-growth boreal forest; **Record Level:** institutionCode: JES

#### Distribution

Holarctic. The description of *Mycomya thula* (Fig. 8) was based on a holotype male from USA, Alaska (Väisänen 1984), with no further records. New for the Palaearctic region and the first record of this species from Finland.

#### Ecology

Extremely poorly known species. Finnish collecting sites are headwater streams with rich riparian vegetation, surrounded by coniferous forests. Immature stages are unknown.

### Mycomya (Mycomyopsis) fennica

Väisänen, 1979

http://www.faunaeur.org/full_results.php?id=139643

#### Materials

- **Type status:** Other material. **Occurrence:** recordedBy: J. Salmela; individualCount: 31; sex: male; **Location:** country: Finland; stateProvince: Regio aboensis; verbatimLocality: Turku, Pomponrahka; decimalLatitude: 60.508; decimalLongitude: 22.250; geodeticDatum: WGS84; **Identification:** identifiedBy: N. Vartija; **Event:** samplingProtocol: Malaise trap; eventDate: 2011-8-15/10-3; habitat: rich fen; **Record Level:** institutionCode: JES
- **Type status:** Other material. **Occurrence:** recordedBy: Jari Ilmonen; individualCount: 24; sex: male; **Location:** country: Finland; stateProvince: Nylandia; verbatimLocality: Espoo, Matalajärvi; verbatimElevation: 23 m; decimalLatitude: 60.247; decimalLongitude: 24.686; geodeticDatum: WGS84; **Identification:** identifiedBy: J. Salmela; **Event:** samplingProtocol: Malaise trap; eventDate: 2012-7-21/10-20; habitat: swampy lake shore
- **Type status:** Other material. **Occurrence:** catalogNumber: MYCE-JS-2012-0011; recordedBy: J. Salmela; individualCount: 1; sex: male; **Location:** country: Finland; stateProvince: Ostrobothnia borealis pars borealis; verbatimLocality: Tornio, Rakanjänkkä; decimalLatitude: 65.890; decimalLongitude: 24.317; geodeticDatum: WGS84; **Identification:** identifiedBy: J. Salmela; **Event:** samplingProtocol: Malaise trap; eventDate: 2012-8-6/9-26; habitat: rich spring fen; **Record Level:** institutionCode: JES
- **Type status:** Other material. **Occurrence:** catalogNumber: MYCE-JS-2012-0023; recordedBy: J. Salmela; individualCount: 1; sex: male; **Location:** country: Finland; stateProvince: Lapponia kemensis pars orientalis; verbatimLocality: Sodankylä, Heinäaapa; decimalLatitude: 67.596; decimalLongitude: 26.883; geodeticDatum: WGS84; **Identification:** identifiedBy: J. Salmela; **Event:** samplingProtocol: Malaise trap; eventDate: 2012-8-10/9-19; habitat: rich spring fen; **Record Level:** institutionCode: JES
- **Type status:** Other material. **Occurrence:** catalogNumber: MYCE-NV-2013-0013; recordedBy: J. Salmela; individualCount: 1; sex: male; **Location:** country: Finland; stateProvince: Lapponia kemensis pars occidentalis; verbatimLocality: Kittilä, Akaharamanvuoma; decimalLatitude: 67.593; decimalLongitude: 25.302; geodeticDatum: WGS84; **Identification:** identifiedBy: N. Vartija; **Event:** samplingProtocol: Malaise trap; eventDate: 2007-8-2/9-3; habitat: intermediate rich flark fen; **Record Level:** institutionCode: JES
- **Type status:** Other material. **Occurrence:** catalogNumber: MYCE-NV-2013-0031; recordedBy: J. Salmela; individualCount: 7; sex: male; **Location:** country: Finland; stateProvince: Lapponia kemensis pars occidentalis; verbatimLocality: Kittilä, Vasanvuoma; decimalLatitude: 67.582; decimalLongitude: 25.203; geodeticDatum: WGS84; **Identification:** identifiedBy: N. Vartija; **Event:** samplingProtocol: Malaise trap; eventDate: 2007-8-2/9-3; habitat: rich fen; **Record Level:** institutionCode: JES

#### Distribution

European. Known from northern Europe, Estonia, Austria, Germany and NW Russia (Chandler 2004). In Fennoscandia only known from Norway (Søli and Kjaerandsen 2008) and Finland (Väisänen 1984).

#### Ecology

The species is most likely associated with peatlands (Väisänen 1984, Søli and Kjaerandsen 2008). All new material presented here was collected from mires, invariably from minerotrophic rich fens. Immature stages are unknown.

### Acnemia amoena

Winnertz, 1864*

http://www.faunaeur.org/full_results.php?id=139484

#### Materials

- **Type status:** Other material. **Occurrence:** recordedBy: J.Jakovlev; individualCount: 1; sex: male; **Location:** country: Finland; stateProvince: Nylandia; municipality: Helsinki; locality: Tuomarinkylä; decimalLatitude: 60.262; decimalLongitude: 24.966; geodeticDatum: WGS84; **Identification:** identifiedBy: J.Jakovlev; **Event:** samplingProtocol: Malaise trap; eventDate: 2011-6-5/7-4; habitat: Wood-storage area in Helsinki; **Record Level:** institutionCode: JJH
- **Type status:** Other material. **Occurrence:** recordedBy: J.Penttinen; individualCount: 1; sex: male; **Location:** country: Finland; stateProvince: Savonia australis; municipality: Rantasalmi; locality: Linnansaari; decimalLatitude: 62.113; decimalLongitude: 28.479; geodeticDatum: WGS84; **Identification:** identifiedBy: J.Penttinen; **Event:** samplingProtocol: Malaise trap; eventDate: 2008-5-20/6-24; habitat: old-growth forest, herb-rich type; **Record Level:** institutionCode: JPJ
- **Type status:** Other material. **Occurrence:** recordedBy: J.Jakovlev; individualCount: 1; sex: female; **Location:** country: Finland; stateProvince: Nylandia; municipality: Vantaa; locality: Kylmäoja, Valkoisenlahdentie; decimalLatitude: 60.303; decimalLongitude: 25.015; geodeticDatum: WGS84; **Identification:** identifiedBy: J.Jakovlev; **Event:** samplingProtocol: pit-fall traps in dead wood storage area; eventDate: 2011-6-10/7-3; habitat: herb-rich forest; **Record Level:** institutionCode: JJH

#### Distribution

Palaearctic. Widely distributed in Central Europe, Mediterranean countries (Spain, Italy, Malta) and found also in Israel, the Near East (Chandler 1994, Chandler 2004) and Northern Caucasus (Zaitzev 1994). From northern Europe only known from Sweden (Kjaerandsen et al. 2007) and Denmark (Petersen and Meier 2001). Included in the recent Red List assessment of Finnish species (Penttinen et al. 2010), but here formally reported for the first time from Finland.

#### Ecology

Larvae are saproxylic. They develop on the surface of dead wood impregnated with fungal mycelium (Zaitzev 1994). Finnish specimens were collected in semi-urban habitats (Helsinki city parks) and in herb-rich forests with exceptionally fertile soils (Linnansaari, Rantasalmi) in the southern parts of the country.

#### Conservation

Threatened species in Finland (VU, Penttinen et al. 2010)

### Acnemia trifida

Zaitzev, 1982***

http://www.catalogueoflife.org/col/details/species/id/8662688

#### Materials

- **Type status:** Other material. **Occurrence:** catalogNumber: MYCE-NV-2013-0089; recordedBy: J. Salmela; individualCount: 2; sex: male; **Taxon:** genus: Acnemia; specificEpithet: trifida; scientificNameAuthorship: Zaitzev, 1982; **Location:** country: Finland; stateProvince: Lapponia kemensis pars occidentalis; verbatimLocality: Kittilä, Kielisenpalo; decimalLatitude: 68.020; decimalLongitude: 25.063; geodeticDatum: WGS84; **Identification:** identifiedBy: N. Vartija; J. Salmela; **Event:** samplingProtocol: Malaise trap; eventDate: 2007-8-2/9-3; habitat: rich spring fen; **Record Level:** institutionCode: JES
- **Type status:** Other material. **Occurrence:** catalogNumber: MYCE-NV-2013-0080; recordedBy: J. Salmela; individualCount: 14; sex: male; **Taxon:** genus: Acnemia; specificEpithet: trifida; scientificNameAuthorship: Zaitzev, 1982; **Location:** country: Finland; stateProvince: Lapponia kemensis pars occidentalis; verbatimLocality: Kittilä, Nunaravuoma; decimalLatitude: 67.699; decimalLongitude: 25.353; geodeticDatum: WGS84; **Identification:** identifiedBy: N. Vartija; J. Salmela; **Event:** samplingProtocol: Malaise trap; eventDate: 2007-8-2/9-3; habitat: poor sedge fen; **Record Level:** institutionCode: JES

#### Distribution

Holarctic. Hitherto only known from North America (USA, Zaitzev 1982a). Here reported for the first time from the Palaearctic region, new for Finland.

#### Ecology

Both collecting sites are pristine north boreal aapamires; Nunaravuoma is a poor sedge fen and Kielisenpalo a rich spring fen. Immature stages are unknown.

### Anaclileia dispar

(Winnertz, 1863)*

http://www.faunaeur.org/full_results.php?id=139472

#### Materials

- **Type status:** Other material. **Occurrence:** catalogNumber: MYCE-NV-2013-0238; recordedBy: J. Salmela; individualCount: 4; sex: male; **Location:** country: Finland; stateProvince: Lapponia kemensis pars occidentalis; verbatimLocality: Kittilä, Pomokaira, Tarpomapää; decimalLatitude: 67.820; decimalLongitude: 25.919; geodeticDatum: WGS84; **Identification:** identifiedBy: N. Vartija; **Event:** samplingProtocol: Malaise trap; eventDate: 2009-6-1/29; **Record Level:** institutionCode: JES
- **Type status:** Other material. **Occurrence:** catalogNumber: MYCE-JS-2013-0400; recordedBy: J. Salmela; T. Hietajärvi; individualCount: 2; sex: male; **Location:** country: Finland; stateProvince: Regio kuusamoensis; verbatimLocality: Salla, Kuntasjoki, Värriö Strict Nature Reserve; verbatimElevation: 320 m; decimalLatitude: 67.749; decimalLongitude: 29.617; geodeticDatum: WGS84; **Identification:** identifiedBy: J. Salmela; **Event:** samplingProtocol: Malaise trap; verbatimEventDate: 2013-6-4/29; habitat: headwater stream, old-growth boreal forest; **Record Level:** institutionCode: JES

#### Distribution

European. Rather wide range in Central Europe (Bechev 1990), records from Fennoscandia are few and scattered. In Sweden only known from Lule Lapmark (North Sweden, Kjaerandsen et al. 2007). In Norway recorded from the oceanic SW part of the country (Kjaerandsen and Jordal 2007). Also known from the Republic of Karelia, in the northern part of the White Sea shore (Humala and Polevoi 2008). New for Finland.

#### Ecology

The life history of *Anaclileia dispar* is not known. The Finnish collecting sites are small lotic waters surrounded by moist old-growth boreal forests.

### Anaclileia dziedzickii

(Landrock, 1911)*

http://www.faunaeur.org/full_results.php?id=139474

#### Materials

- **Type status:** Other material. **Occurrence:** catalogNumber: MYCE-NV-2013-0044; recordedBy: J. Salmela; individualCount: 1; sex: male; **Location:** country: Finland; stateProvince: Lapponia kemensis pars orientalis; verbatimLocality: Sodankylä, Paistipuolet; decimalLatitude: 67.836; decimalLongitude: 26.216; geodeticDatum: WGS84; **Identification:** identifiedBy: N. Vartija; J. Salmela; **Event:** samplingProtocol: Malaise trap; eventDate: 2009-6-1/29; **Record Level:** institutionCode: JES
- **Type status:** Other material. **Occurrence:** catalogNumber: MYCE-NV-2013-0133; recordedBy: J. Salmela; individualCount: 1; sex: male; **Location:** country: Finland; stateProvince: Lapponia kemensis pars orientalis; verbatimLocality: Sodankylä, Tarpomapää E; decimalLatitude: 67.831; decimalLongitude: 25.993; geodeticDatum: WGS84; **Identification:** identifiedBy: N. Vartija; J. Salmela; **Event:** samplingProtocol: Malaise trap; eventDate: 2009-6-1/29; **Record Level:** institutionCode: JES
- **Type status:** Other material. **Occurrence:** catalogNumber: MYCE-NV-2013-0239; recordedBy: J. Salmela; individualCount: 1; sex: male; **Location:** country: Finland; stateProvince: Lapponia kemensis pars occidentalis; verbatimLocality: Kittilä, Tarpomapää; decimalLatitude: 67.820; decimalLongitude: 25.919; geodeticDatum: WGS84; **Identification:** identifiedBy: N. Vartija; **Event:** samplingProtocol: Malaise trap; eventDate: 2009-6-1/29; **Record Level:** institutionCode: JES
- **Type status:** Other material. **Occurrence:** recordedBy: J.Penttinen; individualCount: 1; sex: male; **Location:** country: Finland; stateProvince: Tavastia borealis; municipality: Saarijärvi; locality: Mastomäki S 1; decimalLatitude: 62.836; decimalLongitude: 25.474; geodeticDatum: WGS84; **Identification:** identifiedBy: J.Penttinen; **Event:** samplingProtocol: Malaise trap; eventDate: 2008-5-8/6-11; habitat: old-growth forest, Myrtillus-Oxalis type; **Record Level:** institutionCode: JPJ
- **Type status:** Other material. **Occurrence:** recordedBy: J.Penttinen; individualCount: 1; sex: male; **Location:** country: Finland; stateProvince: Tavastia borealis; municipality: Saarijärvi; locality: Pyhä-Häkki National Park; decimalLatitude: 62.836; decimalLongitude: 25.475; geodeticDatum: WGS84; **Identification:** identifiedBy: J.Penttinen; **Event:** samplingProtocol: Malaise trap; eventDate: 2008; habitat: old-growth forest, Myrtillus type; **Record Level:** institutionCode: JPJ
- **Type status:** Other material. **Occurrence:** catalogNumber: DIPT-JS-2014-0002; recordedBy: J. Salmela; T. Hietajärvi; individualCount: 1; sex: male; **Location:** country: Finland; stateProvince: Regio kuusamoensis; verbatimLocality: Salla, Kuntasjoki, Värriö Strict Nature Reserve; verbatimElevation: 320 m; decimalLatitude: 67.749; decimalLongitude: 29.617; geodeticDatum: WGS84; **Identification:** identifiedBy: J. Salmela; **Event:** samplingProtocol: Malaise trap; verbatimEventDate: 2013-6-4/29; habitat: headwater stream, old-growth boreal forest; **Record Level:** institutionCode: JES

#### Distribution

European. Recorded from Central and northern Europe (Chandler 2004). Very rare and poorly known in Fennoscandia, only a few findings from Sweden (Lule Lapmark, Kjaerandsen et al. 2007) and Russian Karelia (Karelia keretina, Yakovlev et al. 2000). The species was included in the recent Finnish Red List assessment, but is here formally reported for the first time from Finland.

#### Ecology

Finnish collecting localities are aapamires and old-growth boreal forests. In Russian Karelia found only in the intact forest area in Paanajärvi National Park (Polevoi 2000, Yakovlev et al. 2000). Immature stages are unknown; related genera develop in fungi or rotten wood.

#### Conservation

Threatened species in Finland (VU, Penttinen et al. 2010).

### Eudicrana nigriceps

(Lundström, 1909)**

http://www.faunaeur.org/full_results.php?id=140890

#### Materials

- **Type status:** Other material. **Occurrence:** recordedBy: A. Polevoi; individualCount: 1; sex: male; **Location:** country: Russia; stateProvince: Republic Karelia; verbatimLocality: Obzha, Mayachino; decimalLatitude: 60.777; decimalLongitude: 32.818; geodeticDatum: WGS84; **Identification:** identifiedBy: A. Polevoi; **Event:** samplingProtocol: Sweep netting; eventDate: 2012-6-25; **Record Level:** institutionCode: FRIP
- **Type status:** Other material. **Occurrence:** catalogNumber: MYCE-JS-2013-0367; recordedBy: J. Salmela; individualCount: 1; sex: male; **Location:** country: Finland; stateProvince: Ostrobothnia borealis pars borealis; verbatimLocality: Ylitornio, Mustiaapa-Kaattasjärvi, Mustipalo N; decimalLatitude: 66.473; decimalLongitude: 25.012; geodeticDatum: WGS84; **Identification:** identifiedBy: J. Salmela; **Event:** samplingProtocol: sweep net; eventDate: 2013-06-20; habitat: spruce mire; **Record Level:** institutionCode: JES
- **Type status:** Other material. **Occurrence:** recordedBy: E.Olund; individualCount: 1; sex: male; **Location:** country: Finland; stateProvince: Alandia; municipality: Hammarland; locality: Äppelö; decimalLatitude: 60.372; decimalLongitude: 19.700; geodeticDatum: WGS84; **Identification:** identifiedBy: C.Lundström; **Event:** eventDate: 1900; **Record Level:** institutionCode: MZHF
- **Type status:** Other material. **Occurrence:** recordedBy: R.Storе; individualCount: 1; sex: male; **Location:** country: Finland; stateProvince: Ostrobothnia australis; municipality: Pietarsaari; locality: Jakobstad; decimalLatitude: 63.665; decimalLongitude: 22.672; geodeticDatum: WGS84; **Identification:** identifiedBy: W.Hackman; **Event:** eventDate: 1932-9-26/9-26; **Record Level:** institutionCode: MZHF
- **Type status:** Other material. **Occurrence:** recordedBy: R.Storå; individualCount: 1; sex: male; **Location:** country: Finland; stateProvince: Regio aboënsis; municipality: Sauvo; locality: Karuna; decimalLatitude: 60.264; decimalLongitude: 22.550; geodeticDatum: WGS84; **Identification:** identifiedBy: W.Hackman; **Event:** eventDate: 1934-4-8/4-8; **Record Level:** institutionCode: MZHF
- **Type status:** Other material. **Occurrence:** recordedBy: A.Nordman; individualCount: 1; sex: male; **Location:** country: Finland; stateProvince: Alandia; municipality: Lemland; locality: Norrhamn; decimalLatitude: 60.073; decimalLongitude: 20.001; geodeticDatum: WGS84; **Identification:** identifiedBy: W.Hackman; **Event:** eventDate: 1943-7-24/7-24; **Record Level:** institutionCode: MZHF
- **Type status:** Other material. **Occurrence:** recordedBy: W.Hackman; individualCount: 1; sex: male; **Location:** country: Finland; stateProvince: Nylandia; municipality: Espoo; locality: Westend; decimalLatitude: 60.159; decimalLongitude: 24.799; geodeticDatum: WGS84; **Identification:** identifiedBy: W.Hackman; **Event:** eventDate: 1968-7-14/7-14; **Record Level:** institutionCode: MZHF
- **Type status:** Other material. **Occurrence:** recordedBy: J.Jakovlev; individualCount: 1; sex: male; **Location:** country: Finland; stateProvince: Regio aboënsis; municipality: Karjalohja; locality: Karkali_South; decimalLatitude: 60.238; decimalLongitude: 23.784; geodeticDatum: WGS84; **Identification:** identifiedBy: J.Jakovlev; **Event:** samplingProtocol: Malaise trap; eventDate: 2004-6-1/6-14; habitat: old-growth forest, herb-rich type; **Record Level:** institutionCode: JJH

#### Distribution

A rare European species. Besides Britain the species was recorded only from Northern Europe: Finland (Lundström 1909, as *Neoempheria nigriceps*), Norway (Økland 1999), Sweden (Kjaerandsen et al. 2007), Estonia (Lackschewitz 1937) and NW Russia (Ostroverkhova and Stackelberg 1988, Chandler 2004). However, that Russian ("Northwest") record given by Ostroverkhova & Stackelberg (Ostroverkhova and Stackelberg 1988) may actually refer to the Estonian record given by Lackschewitz (Lackschewitz 1937). A few old Finnish records are mainly from southern Finland; from Åland archipelago and a few mainland sites not far from the Baltic coast (Lohja [holotype, Lundström 1909] Karjalohja, Helsinki, Espoo, Pietarsaari). The new Finnish record is from a much more northern area, SW Lapland (mid boreal vegetation zone). Reported here for the first time from the Republic of Karelia.

#### Ecology

Although the species is very rarely caught, the available records suggest that the species could be restricted to pristine forests. New findings from Russian Karelia are from herb-rich spruce dominated forest on the SE shore of Lake Ladoga. The sampling locality in SW Lapland (Ylitornio) is a spruce mire dominated by *Vaccinium vitis-idaea* on the ground layer, adult specimens were collected around a fallen spruce. Larval microhabitats are not perfectly known. Rearing records are available only from Norway where Økland (Økland 1999) collected *Eudicrana nigriceps* with eclector traps over ground vegetation in three different substrates: (1) patch of Eu-Piceetum ground vegetation, (2) wet moss carpet on steep rock in Eu-Piceetum woodland, (3) mineral soil exposed by windfelling of *Picea abies*.

#### Conservation

Red-listed in Finland (EN, Penttinen et al. 2010).

### Monoclona silvatica

Zaitzev, 1983*

http://www.faunaeur.org/full_results.php?id=139439

#### Materials

- **Type status:** Other material. **Occurrence:** recordedBy: J.Penttinen; individualCount: 1; sex: male; **Location:** country: Finland; stateProvince: Karelia ladogensis; municipality: Parikkala; locality: Kasinniemi; decimalLatitude: 61.565; decimalLongitude: 29.558; geodeticDatum: WGS84; **Identification:** identifiedBy: J.Penttinen; **Event:** samplingProtocol: Malaise trap; eventDate: 2008-7-22/9-1; habitat: old-growth forest, herb-rich type; **Record Level:** institutionCode: JPJ

#### Distribution

Palaearctic. The species is known from the Far East and European parts of Russia (Zaitzev 1994), Central Europe (Chandler 2004) and Norway (Kjaerandsen and Jordal 2007). New for Finland, Finnish sampling sites lie in the south boreal zone.

#### Ecology

Larvae are associated with wood-decaying fungi (Zaitzev 1994). Finnish collecting sites are herb-rich forests with high amounts of decaying deciduous trees and an old-growth boreal forest.

#### Conservation

Red-listed in Norway (DD, Anonymous 2010).

### Phthinia zaitzevi

Plassmann, 1990**

http://www.faunaeur.org/full_results.php?id=139409

#### Materials

- **Type status:** Other material. **Occurrence:** recordedBy: A. Polevoi; individualCount: 1; sex: male; **Location:** country: Russia; stateProvince: Republic Karelia; verbatimLocality: Kartesh, biological station; decimalLatitude: 66.337; decimalLongitude: 33.652; geodeticDatum: WGS84; **Identification:** identifiedBy: A. Polevoi; **Event:** samplingProtocol: Malaise trap; eventDate: 1996-7-30/8-1; **Record Level:** institutionCode: FRIP

#### Distribution

European. Extremely rare species (Fig. 9) which was known from the type locality in Sweden (Plassmann 1990) and later reported from Czech republic (Chandler 2004). However, the record from Czech republic is erroneous (Ševčík and Košel 2009). New to Russia and the Republic of Karelia.

#### Ecology

The Karelian specimen was collected in *Vaccinium myrtillus* type sprucedominated forest. Immature stages are unknown. Generally, *Phthinia* larvae develop in webs on the surface of fungal mycelium and moulds in rotten wood. The larvae pupate in silky cocoon (Plachter 1979).

#### Notes

Plassmann's original figure of male genitalia is sketchy, however the study of the holotype (Sweden, Abisko) confirmed identity of Karelian and Swedish speciemens.

### Sciophila bicuspidata

Zaitzev, 1982*

http://www.faunaeur.org/full_results.php?id=139336

#### Materials

- **Type status:** Other material. **Occurrence:** catalogNumber: MYCE-JS-2013-0222; recordedBy: J. Salmela; individualCount: 1; sex: male; **Location:** country: Finland; stateProvince: Ostrobothnia borealis pars borealis; verbatimLocality: Tornio, Rakanjänkkä; decimalLatitude: 65.890; decimalLongitude: 24.317; geodeticDatum: WGS84; **Identification:** identifiedBy: J. Salmela; **Event:** samplingProtocol: Malaise trap; eventDate: 2012-6-4/7-2; **Record Level:** institutionCode: JES
- **Type status:** Other material. **Occurrence:** catalogNumber: MYCE-JS-2013-0225; recordedBy: J. Salmela; individualCount: 1; sex: male; **Location:** country: Finland; stateProvince: Ostrobothnia borealis pars borealis; verbatimLocality: Tornio, Rakanjänkkä; decimalLatitude: 65.890; decimalLongitude: 24.317; geodeticDatum: WGS84; **Identification:** identifiedBy: J. Salmela; **Event:** samplingProtocol: Malaise trap; eventDate: 2012-7-2/8-6; **Record Level:** institutionCode: JES

#### Distribution

Holarctic. Described from North America, based on material collected from Canada (Quebec, British Columbia) and USA (Alaska) (Zaitzev 1982b), also recently found from Greenland (G. Varkonyi, pers. comm.). In Europe the species has been found only in Norway (Økland and Zaitzev 1997) and Russian Karelia (Polevoi 2001a). New for Finland.

#### Ecology

The collecting site is a calcareous, open spring fen, ca. 100 m from a forest edge. Immature stages are unknown.

### Sciophila caesarea

Chandler, 2001*

http://www.faunaeur.org/full_results.php?id=139338

#### Materials

- **Type status:** Other material. **Occurrence:** recordedBy: J.Salmela; individualCount: 1; sex: male; **Taxon:** genus: Sciophila; specificEpithet: caesarea; scientificNameAuthorship: Chandler, 2001; **Location:** stateProvince: Lapponia inarensis; municipality: Utsjoki; locality: Galddasjohka; decimalLatitude: 69.861; decimalLongitude: 27.809; geodeticDatum: WGS84; **Identification:** identifiedBy: J.Jakovlev; **Event:** samplingProtocol: Malaise trap; eventDate: 2007-7-19/8-27; habitat: stream valley in fell area; **Record Level:** institutionCode: JJH
- **Type status:** Other material. **Occurrence:** recordedBy: J.Jakovlev; J.Penttinen; individualCount: 1; sex: male; **Taxon:** genus: Sciophila; specificEpithet: caesarea; scientificNameAuthorship: Chandler, 2001; **Location:** stateProvince: Lapponia enontekiensis; municipality: Enontekiö; locality: Kilpisjärvi_Saana_South_2; decimalLatitude: 69.035; decimalLongitude: 20.839; geodeticDatum: WGS84; **Identification:** identifiedBy: J.Jakovlev; **Event:** samplingProtocol: Malaise trap; eventDate: 2006-8-1/8-15; habitat: subarctic mountain birch forest; **Record Level:** institutionCode: JJH
- **Type status:** Other material. **Occurrence:** recordedBy: M.Jaschhof; C.Jaschhof; individualCount: 1; sex: male; **Taxon:** genus: Sciophila; specificEpithet: caesarea; scientificNameAuthorship: Chandler, 2001; **Location:** stateProvince: Satakunta; municipality: Ikaalinen; locality: Multinharju; decimalLatitude: 61.907; decimalLongitude: 23.399; geodeticDatum: WGS84; **Identification:** identifiedBy: J.Jakovlev; **Event:** samplingProtocol: Malaise trap; eventDate: 2004-7-2/8-24; habitat: old-growth forest, Myrtillus type; **Record Level:** institutionCode: JJH

#### Distribution

European. Described from Great Britain (Chandler 2001), later found from southern Sweden (Kjaerandsen et al. 2007). New for Finland. Record from Czech republic in Fauna Europaea (Chandler 2004) is erroneous (J. Ševčík, pers.comm.).

#### Ecology

Finnish collecting sites are a luxurious old-growth coniferous forest (Ikaalinen) in central Finland, a mountain birch forest with rich vegetation (Saana) and a subarctic stream valley surrounded by a strip of mountain birch forest (Galddasjohka). Immature stages are unknown.

### Sciophila fuliginosa

Holmgren, 1883

http://www.faunaeur.org/full_results.php?id=139349

#### Materials

- **Type status:** Other material. **Occurrence:** catalogNumber: MYCE-JS-2013-0098; recordedBy: J. Salmela; individualCount: 2; sex: male; **Location:** country: Finland; stateProvince: Lapponia kemensis pars orientalis; verbatimLocality: Savukoski, Törmäoja; decimalLatitude: 67.846; decimalLongitude: 29.471; geodeticDatum: WGS84; **Identification:** identifiedBy: J. Salmela; **Event:** samplingProtocol: Malaise trap; eventDate: 2012-6-14/7-10; **Record Level:** institutionCode: JES
- **Type status:** Other material. **Occurrence:** recordedBy: J. Jakovlev; J. Penttinen; individualCount: 1; sex: male; **Location:** country: Finland; stateProvince: Lapponia enontekiensis; verbatimLocality: Kilpisjärvi, Saana; verbatimElevation: 560 m; decimalLatitude: 69.035; decimalLongitude: 20.839; geodeticDatum: WGS84; **Identification:** identifiedBy: J. Jakovlev; **Event:** samplingProtocol: Malaise trap; eventDate: 2006-8-1/15; **Record Level:** institutionCode: JJH

#### Distribution

The species appears to have an Arctic distribution. Described from Novaya Zemlya archipelago, Matotschkin Sharr (Holmgren 1883), later found in the Russian Arctic, Taimyr Peninsula (Lundström 1915), in Alaska and northern Canada (Zaitzev 1982b). In Fennoscandia only known from Finland (Hackman 1980, Kjaerandsen 2012).

#### Ecology

Finnish collecting sites are a mountain birch forest with herb-rich vegetation (Saana fell) and a luxuriant headwater stream surrounded by an old-growth coniferous forest with a mixture of deciduous trees (Törmäoja). Immature stages are unknown.

### Sciophila salassea

Matile, 1983*

http://www.faunaeur.org/full_results.php?id=139381

#### Materials

- **Type status:** Other material. **Occurrence:** catalogNumber: MYCE-JS-2013-0103; recordedBy: J. Salmela; individualCount: 1; sex: male; **Location:** country: Finland; stateProvince: Lapponia kemensis pars orientalis; verbatimLocality: Savukoski, Törmäoja; decimalLatitude: 67.846; decimalLongitude: 29.471; geodeticDatum: WGS84; **Identification:** identifiedBy: J. Salmela; **Event:** samplingProtocol: Malaise trap; eventDate: 2012-6-14/7-10; **Record Level:** institutionCode: JES
- **Type status:** Other material. **Occurrence:** recordedBy: J. Jakovlev; J. Penttinen; individualCount: 1; sex: male; **Location:** country: Finland; stateProvince: Savonia borealis; verbatimLocality: Savonranta, Kakonsalo; decimalLatitude: 62.250; decimalLongitude: 28.877; geodeticDatum: WGS84; **Identification:** identifiedBy: J. Jakovlev; **Event:** samplingProtocol: eclector trap on aspen log bearing bracket fungus *Phellinus tremulae*; eventDate: 2007-4-28/6-3; **Record Level:** institutionCode: JJH
- **Type status:** Other material. **Occurrence:** catalogNumber: DIPT-JS-2014-0029; recordedBy: J. Salmela; T. Hietajärvi; individualCount: 1; sex: male; **Location:** country: Finland; stateProvince: Regio kuusamoensis; verbatimLocality: Salla, Kuntasjoki, Värriö Strict Nature Reserve; verbatimElevation: 320 m; decimalLatitude: 67.749; decimalLongitude: 29.617; geodeticDatum: WGS84; **Identification:** identifiedBy: J. Salmela; **Event:** samplingProtocol: Malaise trap; eventDate: 2013; verbatimEventDate: 2013-6-4/29; habitat: headwater stream, old-growth boreal forest; **Record Level:** institutionCode: JES

#### Distribution

European, possibly boreal–mountainous. The species was described from the Italian Alps (Matile 1983) and later recorded from Great Britain (Chandler 2006), and from Fennoscandia: Norway (Økland and Zaitzev 1997), Sweden (Kjaerandsen et al. 2007) and Russian Karelia (Humala and Polevoi 2008, Polevoi 2000). The species was included in the Finnish Red List (Penttinen et al. 2010), but is here formally reported as a new species for Finland.

#### Ecology

Collecting site in Törmäoja is a stream valley with seepages and young deciduous forest. Slopes nearby are coniferous stands dominated by *Vaccinium vitis-idaea* and *Pinus sylvestris*. Collecting site in Värriö is a headwater stream surrounded by pristine spruce and pine forest. The male specimen from Savonranta was collected from a decaying aspen (*Populus tremula*) tree by using an eclector trap.

#### Conservation

The species was red-listed in Finland (EN, Penttinen et al. 2010) based on the single record from Savonranta. *Sciophila salassea* is also red-listed in Norway (NT), so far observed from pristine spruce forests (Anonymous 2010, Gammelmo et al. 2010)

### Speolepta leptogaster

(Winnetrz, 1863)

http://www.faunaeur.org/full_results.php?id=139327

#### Materials

- **Type status:** Other material. **Occurrence:** recordedBy: Palmen, J.; individualCount: 1; **Location:** country: Finland; stateProvince: Savonia borealis; verbatimLocality: Maaninka, Tuovilanlahti; decimalLatitude: 63.23; decimalLongitude: 27.09; geodeticDatum: WGS84; **Event:** eventDate: 1865-7-16; **Record Level:** institutionCode: MZHF
- **Type status:** Other material. **Occurrence:** recordedBy: J. Jakovlev; J. Penttinen; individualCount: 1; sex: male; **Location:** country: Finland; stateProvince: Lapponia kemensis pars occidentalis; verbatimLocality: Muonio, Pallasjärvi; decimalLatitude: 68.018; decimalLongitude: 24.153; geodeticDatum: WGS84; **Event:** samplingProtocol: Malaise trap; eventDate: 2006-6-18/7-14
- **Type status:** Other material. **Occurrence:** recordedBy: J. Penttinen; individualCount: 1; **Location:** country: Finland; stateProvince: Tavastia australis; verbatimLocality: Kangasala, Suorama; decimalLatitude: 61.463; decimalLongitude: 23.995; geodeticDatum: WGS84; **Event:** samplingProtocol: Malaise trap; eventDate: 2009-6-15/8-15; habitat: dry/semi-dry herb-rich forest, with *Tilia* and *Populus tremula*
- **Type status:** Other material. **Occurrence:** catalogNumber: MYCE-JS-2012-0031; recordedBy: J. Salmela; individualCount: 1; sex: male; **Location:** country: Finland; stateProvince: Lapponia kemensis pars orientalis; verbatimLocality: Savukoski, Törmäoja; decimalLatitude: 67.846; decimalLongitude: 29.471; geodeticDatum: WGS84; **Identification:** identifiedBy: J. Salmela; **Event:** samplingProtocol: Malaise trap; eventDate: 2012-6-14/7-10; habitat: headwater stream, old-growth boreal forest; **Record Level:** institutionCode: JES

#### Distribution

European. Widely distributed in Europe (Chandler 2004), also in the Nordic Region, but records from Finland are very scanty. Only one recent record from Murmansk Province in NW Russia (Polevoi 2010).

#### Ecology

The species lives as larvae in caves and rock crevices, on the walls, in slimy tubes, pupae are free hanging (Madwar 1937, Ševčík et al. 2012).

### Boletina atridentata

Polevoi & Hedmark, 2004*

http://www.faunaeur.org/full_results.php?id=140794

#### Materials

- **Type status:** Other material. **Occurrence:** catalogNumber: MYCE-JS-2013-0127; recordedBy: J. Salmela; individualCount: 1; sex: male; **Location:** country: Finland; stateProvince: Lapponia kemensis pars orientalis; verbatimLocality: Savukoski, Joutenoja; decimalLatitude: 67.821; decimalLongitude: 29.440; geodeticDatum: WGS84; **Identification:** identifiedBy: J. Salmela; **Event:** samplingProtocol: Malaise trap; eventDate: 2012-7-10/8-16; habitat: headwater stream, seminatural boreal forest; **Record Level:** institutionCode: JES
- **Type status:** Other material. **Occurrence:** catalogNumber: MYCE-JS-2013-0052; recordedBy: J. Salmela; individualCount: 1; sex: male; **Location:** country: Finland; stateProvince: Lapponia kemensis pars orientalis; verbatimLocality: Savukoski, Joutenoja; decimalLatitude: 67.821; decimalLongitude: 29.440; geodeticDatum: WGS84; **Identification:** identifiedBy: J. Salmela; **Event:** samplingProtocol: Malaise trap; eventDate: 2012-8-16/9-18; **Record Level:** institutionCode: JES
- **Type status:** Other material. **Occurrence:** catalogNumber: MYCE-NV-2013-0015; recordedBy: J. Salmela; individualCount: 1; sex: male; **Location:** country: Finland; stateProvince: Lapponia kemensis pars occidentalis; verbatimLocality: Kittilä, Akaharamanvuoma; decimalLatitude: 67.593; decimalLongitude: 25.302; geodeticDatum: WGS84; **Identification:** identifiedBy: N. Vartija; **Event:** samplingProtocol: Malaise trap; eventDate: 2007-8-2/9-3; habitat: intermediate rich flark fen; **Record Level:** institutionCode: JES
- **Type status:** Other material. **Occurrence:** catalogNumber: MYCE-NV-2013-0096; recordedBy: J. Salmela; individualCount: 1; sex: male; **Location:** country: Finland; stateProvince: Lapponia kemensis pars occidentalis; verbatimLocality: Kittilä, Vasanvuoma; decimalLatitude: 67.582; decimalLongitude: 25.203; geodeticDatum: WGS84; **Identification:** identifiedBy: N. Vartija; **Event:** samplingProtocol: Malaise trap; eventDate: 2007-8-2/9-3; habitat: rich fen; **Record Level:** institutionCode: JES

#### Distribution

**Figure 10.**
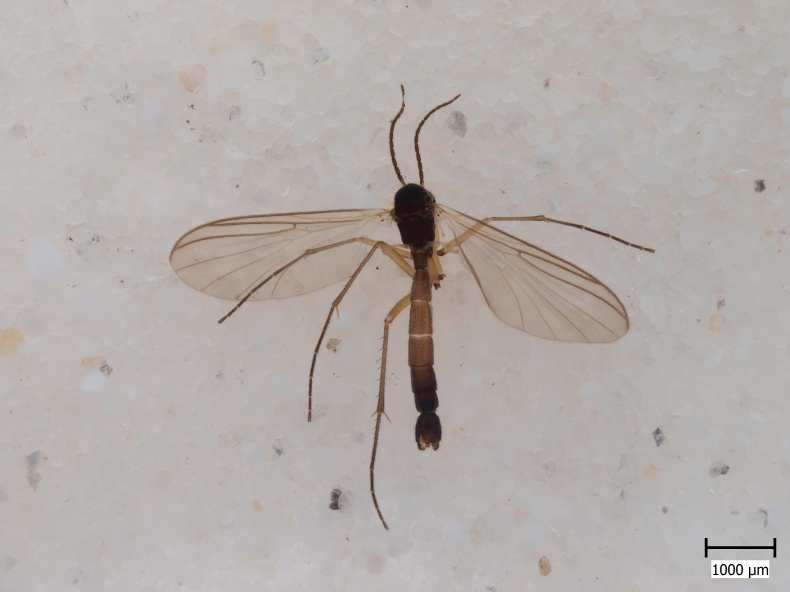
*Boletina atridentata* Polevoi & Hedmark, male specimen collected from Finland, Savukoski (northeastern Lapland).

Palaearctic. *Boletina atridentata* (Fig. 10) was described from North Sweden (Lule Lapmark) and Russian Karelia (Paanajärvi) (Polevoi and Hedmark 2004). Other Swedish records are also from Lule Lapmark (Kjaerandsen et al. 2007) and additional records reported from NW Russia (Humala and Polevoi 2008, Polevoi 2010). There is also an unpublished record from West Siberia (E.Subbotina in litt.). Finnish localities are situated in central and eastern Lapland, north boreal zone.

#### Ecology

Finnish collecting sites are a headwater stream with luxuriant riparian vegetation, surrounded by coniferous forest (Joutenoja), and aapamires (sites in Kittilä). Immature stages are unknown.

### Boletina borealis

Zetterstedt, 1852

http://www.faunaeur.org/full_results.php?id=140802

#### Materials

- **Type status:** Other material. **Occurrence:** recordedBy: J. Jakovlev; individualCount: 1; sex: male; **Location:** country: Finland; stateProvince: Lapponia kemensis pars occidentalis; verbatimLocality: Kolari; decimalLatitude: 67.210; decimalLongitude: 23.799; geodeticDatum: WGS84; **Identification:** identifiedBy: J. Jakovlev; **Event:** samplingProtocol: sweep net; eventDate: 2006-6-14
- **Type status:** Other material. **Occurrence:** recordedBy: J. Jakovlev; individualCount: 1; sex: male; **Location:** country: Finland; stateProvince: Lapponia enontekiensis; verbatimLocality: Enontekiö, Kilpisjärvi, Saana; decimalLatitude: 69.004; decimalLongitude: 20.817; geodeticDatum: WGS84; **Identification:** identifiedBy: J. Jakovlev; **Event:** samplingProtocol: sweep net; eventDate: 2006-6-21
- **Type status:** Other material. **Occurrence:** recordedBy: J. Salmela; individualCount: 5; sex: male; **Location:** country: Finland; stateProvince: Lapponia inarensis; verbatimLocality: Utsjoki, Kaldoaivi, Galddasjohka; decimalLatitude: 69.860; decimalLongitude: 27.809; geodeticDatum: WGS84; **Identification:** identifiedBy: J. Jakovlev; **Event:** samplingProtocol: Malaise trap; eventDate: 2007-7-15/8-27
- **Type status:** Other material. **Occurrence:** recordedBy: J. Salmela; individualCount: 2; sex: male; **Location:** country: Finland; stateProvince: Ostrobothnia borealis pars borealis; verbatimLocality: Tornio, Rakanjänkkä; decimalLatitude: 65.890; decimalLongitude: 24.317; geodeticDatum: WGS84; **Identification:** identifiedBy: J. Salmela; **Event:** samplingProtocol: Malaise trap; eventDate: 2012-6-2/7-2
- **Type status:** Other material. **Occurrence:** recordedBy: J. Salmela; individualCount: 7; sex: male; **Location:** country: Finland; stateProvince: Ostrobothnia borealis pars borealis; verbatimLocality: Tervola, Ruuttulammi; decimalLatitude: 66.207; decimalLongitude: 24.898; geodeticDatum: WGS84; **Identification:** identifiedBy: J. Salmela; **Event:** samplingProtocol: Malaise trap; eventDate: 2012-6-4/7-2
- **Type status:** Other material. **Occurrence:** recordedBy: J. Salmela; individualCount: 21; sex: male; **Location:** country: Finland; stateProvince: Ostrobothnia borealis pars borealis; verbatimLocality: Yli-Tornio, Tuorerommas; decimalLatitude: 66.476; decimalLongitude: 24.756; geodeticDatum: WGS84; **Identification:** identifiedBy: J. Salmela; **Event:** samplingProtocol: Malaise trap; eventDate: 2012-6-4/7-2
- **Type status:** Other material. **Occurrence:** recordedBy: J. Salmela; individualCount: 5; sex: male; **Location:** country: Finland; stateProvince: Ostrobothnia borealis pars borealis; verbatimLocality: Rovaniemi, Aitakuru; decimalLatitude: 66.938; decimalLongitude: 25.935; geodeticDatum: WGS84; **Identification:** identifiedBy: J. Salmela; **Event:** samplingProtocol: Malaise trap; eventDate: 2012-6-5/7-4
- **Type status:** Other material. **Occurrence:** catalogNumber: MYCE-JS-2013-0002; recordedBy: J. Salmela; individualCount: 3; sex: male; **Location:** country: Finland; stateProvince: Lapponia kemensis pars orientalis; verbatimLocality: Sodankylä, Pomokaira, Kaita-aapa; decimalLatitude: 67.845; decimalLongitude: 26.553; geodeticDatum: WGS84; **Identification:** identifiedBy: J. Salmela; **Event:** samplingProtocol: Malaise trap; eventDate: 2012-6-5/7-4
- **Type status:** Other material. **Occurrence:** recordedBy: J. Salmela; individualCount: 9; sex: male; **Location:** country: Finland; stateProvince: Lapponia kemensis pars orientalis; verbatimLocality: Sodankylä, Pomokaira, Tenniöaapa; decimalLatitude: 67.867; decimalLongitude: 26.449; geodeticDatum: WGS84; **Identification:** identifiedBy: J. Salmela; **Event:** samplingProtocol: Malaise trap; eventDate: 2012-6-6/7-7
- **Type status:** Other material. **Occurrence:** recordedBy: J. Salmela; individualCount: 2; sex: male; **Location:** country: Finland; stateProvince: Lapponia kemensis pars orientalis; verbatimLocality: Sodankylä, Viiankiaapa, Sakatti; decimalLatitude: 67.544; decimalLongitude: 26.760; geodeticDatum: WGS84; **Identification:** identifiedBy: J. Salmela; **Event:** samplingProtocol: Malaise trap; eventDate: 2012-6-5/7-6
- **Type status:** Other material. **Occurrence:** catalogNumber: MYCE-JS-2012-0085; recordedBy: J. Salmela; individualCount: 9; sex: male; **Location:** country: Finland; stateProvince: Lapponia kemensis pars orientalis; verbatimLocality: Sodankylä, Heinäaapa; decimalLatitude: 67.596; decimalLongitude: 26.883; geodeticDatum: WGS84; **Identification:** identifiedBy: J. Salmela; **Event:** samplingProtocol: Malaise trap; eventDate: 2012-6-6/7-6
- **Type status:** Other material. **Occurrence:** catalogNumber: MYCE-JS-2013-0186; recordedBy: J. Salmela; individualCount: 71; sex: male; **Location:** country: Finland; stateProvince: Lapponia kemensis pars orientalis; verbatimLocality: Savukoski, Joutenoja; decimalLatitude: 67.821; decimalLongitude: 29.440; geodeticDatum: WGS84; **Identification:** identifiedBy: J. Salmela; **Event:** samplingProtocol: Malaise trap; eventDate: 2012-6-14/7-10
- **Type status:** Other material. **Occurrence:** recordedBy: J. Salmela; individualCount: 65; sex: male; **Location:** country: Finland; stateProvince: Lapponia kemensis pars orientalis; verbatimLocality: Savukoski, Törmäoja; decimalLatitude: 67.846; decimalLongitude: 29.471; geodeticDatum: WGS84; **Identification:** identifiedBy: J. Salmela; **Event:** samplingProtocol: Malaise trap; eventDate: 2012-6-14/7-10
- **Type status:** Other material. **Occurrence:** catalogNumber: MYCE-NV-2013-0042; recordedBy: J. Salmela; individualCount: 4; sex: male; **Location:** country: Finland; stateProvince: Lapponia kemensis pars orientalis; verbatimLocality: Sodankylä, Paistipuolet; decimalLatitude: 67.836; decimalLongitude: 26.216; geodeticDatum: WGS84; **Identification:** identifiedBy: J. Salmela; **Event:** samplingProtocol: Malaise trap; eventDate: 2009-6-1/29
- **Type status:** Other material. **Occurrence:** recordedBy: J. Salmela; individualCount: 1; sex: male; **Location:** country: Finland; stateProvince: Lapponia kemensis pars occidentalis; verbatimLocality: Kittilä, Repsuvuoma; decimalLatitude: 67.604; decimalLongitude: 24.967; geodeticDatum: WGS84; **Identification:** identifiedBy: N. Vartija; **Event:** samplingProtocol: Malaise trap; eventDate: 2007-6-1/26
- **Type status:** Other material. **Occurrence:** recordedBy: J. Salmela; individualCount: 2; sex: male; **Location:** country: Finland; stateProvince: Lapponia kemensis pars occidentalis; verbatimLocality: Kittilä, Silmäsvuoma; decimalLatitude: 67.582; decimalLongitude: 25.543; geodeticDatum: WGS84; **Identification:** identifiedBy: N. Vartija; **Event:** samplingProtocol: Malaise trap; eventDate: 2007-5-31/6-25
- **Type status:** Other material. **Occurrence:** recordedBy: J. Salmela; individualCount: 4; sex: male; **Location:** country: Finland; stateProvince: Lapponia kemensis pars occidentalis; verbatimLocality: Kittilä, Kielisenpalo; decimalLatitude: 68.020; decimalLongitude: 25.063; geodeticDatum: WGS84; **Identification:** identifiedBy: N. Vartija; **Event:** samplingProtocol: Malaise trap; eventDate: 2007-6-26/8-2
- **Type status:** Other material. **Occurrence:** recordedBy: J. Salmela; individualCount: 12; sex: male; **Location:** country: Finland; stateProvince: Lapponia kemensis pars occidentalis; verbatimLocality: Kittilä, Vuotsonperänjänkä; decimalLatitude: 67.616; decimalLongitude: 25.449; geodeticDatum: WGS84; **Identification:** identifiedBy: N. Vartija; **Event:** samplingProtocol: Malaise trap; eventDate: 2007-5-31/6-26
- **Type status:** Other material. **Occurrence:** recordedBy: J. Salmela; individualCount: 5; sex: male; **Location:** country: Finland; stateProvince: Lapponia kemensis pars occidentalis; verbatimLocality: Kittilä, Vielmakoskenpalo; decimalLatitude: 68.008; decimalLongitude: 25.046; geodeticDatum: WGS84; **Identification:** identifiedBy: N. Vartija; **Event:** samplingProtocol: Malaise trap; eventDate: 2007-6-26/7-24
- **Type status:** Other material. **Occurrence:** recordedBy: J. Salmela; individualCount: 6; sex: male; **Location:** country: Finland; stateProvince: Lapponia kemensis pars occidentalis; verbatimLocality: Kittilä,Tarpomapää; decimalLatitude: 67.820; decimalLongitude: 25.919; geodeticDatum: WGS84; **Identification:** identifiedBy: N. Vartija; **Event:** samplingProtocol: Malaise trap; eventDate: 2009-6-1/29
- **Type status:** Other material. **Occurrence:** recordedBy: Wegelius; individualCount: 1; sex: male; **Location:** country: Finland; stateProvince: Tavastia australis; municipality: Hattula; locality: Hattula; decimalLatitude: 61.058; decimalLongitude: 24.367; geodeticDatum: WGS84; **Identification:** identifiedBy: C.Lundström; **Event:** eventDate: 1905; habitat: old-growth forest, Myrtillus type; **Record Level:** institutionCode: MZHF
- **Type status:** Other material. **Occurrence:** recordedBy: R.Frey; individualCount: 1; sex: male; **Location:** country: Finland; stateProvince: Lapponia enontekiensis; municipality: Enontekiö; locality: Palojoensuu; decimalLatitude: 68.285; decimalLongitude: 23.095; geodeticDatum: WGS84; **Identification:** identifiedBy: C.Lundström; **Event:** eventDate: 1911-7-11/7-11; habitat: subarctic; **Record Level:** institutionCode: MZHF
- **Type status:** Other material. **Occurrence:** recordedBy: J.Penttinen; individualCount: 1; sex: male; **Location:** country: Finland; stateProvince: Tavastia borealis; municipality: Rautalampi; locality: Kalajavuori; decimalLatitude: 62.578; decimalLongitude: 26.698; geodeticDatum: WGS84; **Identification:** identifiedBy: J.Penttinen; **Event:** samplingProtocol: Malaise trap; eventDate: 2004-5-3/6-6; habitat: old-growth forest, Myrtillus type; **Record Level:** institutionCode: JPJ
- **Type status:** Other material. **Occurrence:** recordedBy: J.Jakovlev and J.Penttinen; individualCount: 1; sex: male; **Location:** country: Finland; stateProvince: Lapponia enontekiensis; municipality: Enontekiö; locality: Kilpisjärvi_Saana_North_4; decimalLatitude: 69.045; decimalLongitude: 20.808; geodeticDatum: WGS84; **Identification:** identifiedBy: J.Jakovlev; **Event:** samplingProtocol: Malaise trap; eventDate: 2006-8-1/8-15; habitat: subarctic
- **Type status:** Other material. **Occurrence:** recordedBy: J.Jakovlev; individualCount: 1; sex: male; **Location:** country: Finland; stateProvince: Lapponia enontekiensis; municipality: Enontekiö; locality: Kilpisjärvi_Saana_North_5; decimalLatitude: 69.045; decimalLongitude: 20.809; geodeticDatum: WGS84; **Identification:** identifiedBy: J.Jakovlev; **Event:** samplingProtocol: Sweep netting; eventDate: 2006-6-21/6-21; habitat: Subarctic; **Record Level:** institutionCode: JJH
- **Type status:** Other material. **Occurrence:** recordedBy: J.Jakovlev and J.Penttinen; individualCount: 1; sex: male; **Location:** country: Finland; stateProvince: Lapponia enontekiensis; municipality: Enontekiö; locality: Kilpisjärvi_Saana_South_2; decimalLatitude: 69.035; decimalLongitude: 20.839; geodeticDatum: WGS84; **Identification:** identifiedBy: J.Jakovlev; **Event:** samplingProtocol: Malaise trap; eventDate: 2006-8-15/8-31; startDayOfYear: 15; endDayOfYear: 8; year: 31; month: 8; day: 2006; habitat: subarctic
- **Type status:** Other material. **Occurrence:** recordedBy: J.Jakovlev and J.Penttinen; individualCount: 1; sex: male; **Location:** country: Finland; stateProvince: Lapponia kemensis pars occidentalis; municipality: Kittilä; locality: Pallas-Yllästunturi National Park Pallas_3; decimalLatitude: 68.038; decimalLongitude: 24.136; geodeticDatum: WGS84; **Identification:** identifiedBy: J.Jakovlev; **Event:** samplingProtocol: Malaise trap; eventDate: 2006-8-15/9-15; startDayOfYear: 15; endDayOfYear: 8; year: 15; month: 9; day: 2006; habitat: old-growth forest, Myrtillus type
- **Type status:** Other material. **Occurrence:** recordedBy: J.Jakovlev; individualCount: 1; sex: male; **Location:** country: Finland; stateProvince: Lapponia kemensis pars occidentalis; municipality: Kolari; locality: Kolari_1; decimalLatitude: 67.209; decimalLongitude: 23.779; geodeticDatum: WGS84; **Identification:** identifiedBy: J.Jakovlev; **Event:** samplingProtocol: Sweep netting; eventDate: 2006-6-14/6-14; startDayOfYear: 14; endDayOfYear: 6; year: 14; month: 6; day: 2006; habitat: old-growth forest, Myrtillus type; **Record Level:** institutionCode: JJH
- **Type status:** Other material. **Occurrence:** recordedBy: J.Jakovlev and J.Penttinen; individualCount: 1; sex: male; **Location:** country: Finland; stateProvince: Lapponia kemensis pars occidentalis; municipality: Kolari; locality: Kolari_2; decimalLatitude: 67.280; decimalLongitude: 23.880; geodeticDatum: WGS84; **Identification:** identifiedBy: J.Jakovlev; **Event:** samplingProtocol: Malaise trap; eventDate: 2006-6-14/6-14; startDayOfYear: 14; endDayOfYear: 6; year: 14; month: 6; day: 2006; habitat: burnt forest
- **Type status:** Other material. **Occurrence:** recordedBy: J.Jakovlev and J.Penttinen; individualCount: 1; sex: male; **Location:** country: Finland; stateProvince: Lapponia kemensis pars occidentalis; municipality: Kolari; locality: Kolari_2; decimalLatitude: 67.280; decimalLongitude: 23.880; geodeticDatum: WGS84; **Identification:** identifiedBy: J.Jakovlev; **Event:** samplingProtocol: Malaise trap; eventDate: 2006-7-15/8-15; startDayOfYear: 15; endDayOfYear: 7; year: 15; month: 8; day: 2006; habitat: burnt forest
- **Type status:** Other material. **Occurrence:** recordedBy: J.Jakovlev and J.Penttinen; individualCount: 3; sex: male; **Location:** country: Finland; stateProvince: Lapponia kemensis pars occidentalis; municipality: Kolari; locality: Kolari_3; decimalLatitude: 67.280; decimalLongitude: 23.881; geodeticDatum: WGS84; **Identification:** identifiedBy: J.Jakovlev; **Event:** samplingProtocol: Malaise trap; eventDate: 2006-7-15/8-15; startDayOfYear: 15; endDayOfYear: 7; year: 15; month: 8; day: 2006; habitat: old-growth forest, Myrtillus type
- **Type status:** Other material. **Occurrence:** recordedBy: J.Jakovlev and J.Penttinen; individualCount: 1; sex: male; **Location:** country: Finland; stateProvince: Lapponia kemensis pars occidentalis; municipality: Muonio; locality: Pallas-Yllästunturi National Park; decimalLatitude: 67.592; decimalLongitude: 24.188; geodeticDatum: WGS84; **Identification:** identifiedBy: J.Jakovlev; **Event:** samplingProtocol: Malaise trap; eventDate: 2006-7-15/8-15; startDayOfYear: 15; endDayOfYear: 7; year: 15; month: 8; day: 2006; habitat: old-growth forest, Myrtillus type
- **Type status:** Other material. **Occurrence:** recordedBy: J.Jakovlev and J.Penttinen; individualCount: 1; sex: male; **Location:** country: Finland; stateProvince: Ostrobothnia borealis pars borealis; municipality: Rovaniemi; locality: Pisavaara; decimalLatitude: 66.279; decimalLongitude: 25.029; geodeticDatum: WGS84; **Identification:** identifiedBy: J.Jakovlev; **Event:** samplingProtocol: Sweep netting; eventDate: 2006-6-13/6-13; startDayOfYear: 13; endDayOfYear: 6; year: 13; month: 6; day: 2006; habitat: old-growth forest, herb-rich type
- **Type status:** Other material. **Occurrence:** recordedBy: J.Jakovlev and J.Penttinen; individualCount: 5; sex: male; **Location:** country: Finland; stateProvince: Regio kuusamoënsis; municipality: Kuusamo; locality: Oulanka_1; decimalLatitude: 66.365; decimalLongitude: 29.315; geodeticDatum: WGS84; **Identification:** identifiedBy: J.Jakovlev; **Event:** samplingProtocol: Malaise trap; eventDate: 2007-5-25/6-30; startDayOfYear: 25; endDayOfYear: 5; year: 30; month: 6; day: 2007; habitat: old-growth forest, Myrtillus type
- **Type status:** Other material. **Occurrence:** recordedBy: J.Salmela; individualCount: 1; sex: male; **Location:** country: Finland; stateProvince: Lapponia inarensis; municipality: Utsjoki; locality: Galddasjohka; decimalLatitude: 69.860; decimalLongitude: 27.770; geodeticDatum: WGS84; **Identification:** identifiedBy: J.Jakovlev; **Event:** samplingProtocol: Malaise trap; eventDate: 2007-6-15/7-19; startDayOfYear: 15; endDayOfYear: 6; year: 19; month: 7; day: 2007; habitat: subarctic; **Record Level:** institutionCode: JJH
- **Type status:** Other material. **Occurrence:** recordedBy: J.Salmela; individualCount: 1; sex: male; **Location:** country: Finland; stateProvince: Lapponia inarensis; municipality: Utsjoki; locality: Galddasjohka; decimalLatitude: 69.861; decimalLongitude: 27.803; geodeticDatum: WGS84; **Identification:** identifiedBy: J.Jakovlev; **Event:** samplingProtocol: Malaise trap; eventDate: 2007-6-15/7-19; startDayOfYear: 15; endDayOfYear: 6; year: 19; month: 7; day: 2007; habitat: subarctic; **Record Level:** institutionCode: JJH
- **Type status:** Other material. **Occurrence:** recordedBy: J.Salmela; individualCount: 1; sex: male; **Location:** country: Finland; stateProvince: Lapponia inarensis; municipality: Utsjoki; locality: Galddasjohka; decimalLatitude: 69.861; decimalLongitude: 27.790; geodeticDatum: WGS84; **Identification:** identifiedBy: J.Jakovlev; **Event:** samplingProtocol: Malaise trap; eventDate: 2007-7-19/8-27; startDayOfYear: 19; endDayOfYear: 7; year: 27; month: 8; day: 2007; habitat: subarctic; **Record Level:** institutionCode: JJH
- **Type status:** Other material. **Occurrence:** recordedBy: J.Salmela; individualCount: 1; sex: male; **Location:** country: Finland; stateProvince: Lapponia inarensis; municipality: Utsjoki; locality: Galddasjohka; decimalLatitude: 69.861; decimalLongitude: 27.803; geodeticDatum: WGS84; **Identification:** identifiedBy: J.Jakovlev; **Event:** samplingProtocol: Malaise trap; eventDate: 2007-7-19/8-27; startDayOfYear: 19; endDayOfYear: 7; year: 27; month: 8; day: 2007; habitat: subarctic; **Record Level:** institutionCode: JJH

#### Distribution

Palaearctic. *Boletina borealis* (Fig. 11a) is formely recorded in Europe from the northern parts of Russia (Novaya Zemlya, Karelia, Zaitzev 1994, Polevoi 2013a), northern Sweden (provinces JÄ, ÅS,LY,LU, Kjaerandsen et al. 2007), Norway (Finnmark, Søli and Rindal 2012), and also from mountainous areas of Austria, Germany, Italy and Poland (Plassmann 1984, Chandler 2004), indicating a boreal–mountainous to arctic distribution.

#### Ecology

The species is rather numerous in Malaise trap cathces collected from Finnish Lapland in June, less so during July. One of the most common fungus gnats in riparian woodlands and aapamires. Larval ecology is unknown.

#### Taxon discussion

*Boletina borealis* is very close to *Boletina intermedia* Lundström. These two taxa can reliably be distinguished if internal structures, especially parameres, of the male hypopygium are studied (see Fig. 11). In *Boletina borealis*, apices of parameres are thin and curved (Fig. 11a), whereas in *Boletina intermedia* these are stout and spear-shaped (Fig. 11b). It is likely that *Boletina intermedia* has been overlooked in faunistic surveys, but the material studied here suggests that *Boletina borealis* is rather common in North Finland, whereas *Boletina intermedia* is locally less abundant and has a smaller area of occupancy. Another species, perhaps an undescribed species close to *Boletina hymenophalloides* Sasakawa & Kimura, 1974, is superficially similar to *Boletina borealis* and *Boletina intermedia*. However, aedeagus of this species is surrounded by a weakly sclerotized membrane and additional differences are present in the sturucture of sternal submedian appendages and parameres. This species, collected from NE Lapland, will be described elsewhere.

### Boletina cincticornis

(Walker, 1848)

http://www.faunaeur.org/full_results.php?id=140805

#### Materials

- **Type status:** Other material. **Occurrence:** recordedBy: R.Frey; individualCount: 1; sex: male; **Location:** stateProvince: Lapponia kemensis pars occidentalis; municipality: Muonio; locality: Pallastunturi; decimalLatitude: 68.072; decimalLongitude: 24.068; geodeticDatum: WGS84; **Identification:** identifiedBy: C.Lundström; **Event:** eventDate: 1900-6-28/6-28; **Record Level:** institutionCode: MZHF
- **Type status:** Other material. **Occurrence:** recordedBy: Grönvik; individualCount: 1; sex: male; **Location:** stateProvince: Karelia borealis; municipality: Kontiolahti; locality: unknown_locality; **Identification:** identifiedBy: C.Lundström; **Event:** samplingProtocol: sweep netting ?; eventDate: 1911; **Record Level:** institutionCode: MZHF
- **Type status:** Other material. **Occurrence:** recordedBy: R.Frey; individualCount: 1; sex: male; **Location:** stateProvince: Lapponia kemensis pars occidentalis; municipality: Muonio; locality: Pallastunturi; decimalLatitude: 68.072; decimalLongitude: 24.068; geodeticDatum: WGS84; **Identification:** identifiedBy: C.Lundström; **Event:** eventDate: 1911-6-13/6-13; **Record Level:** institutionCode: MZHF
- **Type status:** Other material. **Occurrence:** recordedBy: M.Jaschhof and C.Jaschhof; individualCount: 1; sex: male; **Location:** stateProvince: Karelia borealis; municipality: Pielisjärvi; locality: 4km SE Koli village; decimalLatitude: 63.050; decimalLongitude: 29.496; geodeticDatum: WGS84; **Identification:** identifiedBy: J.Jakovlev; **Event:** samplingProtocol: Sweep netting; eventDate: 2004-6-10/6-10; habitat: meadow and dry hay at birch forest edge; **Record Level:** institutionCode: JJH
- **Type status:** Other material. **Occurrence:** recordedBy: J.Penttinen; individualCount: 1; sex: male; **Location:** stateProvince: Tavastia borealis; municipality: Jyväskylä; locality: Huhtala; decimalLatitude: 62.205; decimalLongitude: 25.683; geodeticDatum: WGS84; **Identification:** identifiedBy: J.Penttinen; **Event:** samplingProtocol: Malaise trap; eventDate: 2005; habitat: Clear-cut; **Record Level:** institutionCode: JPJ
- **Type status:** Other material. **Occurrence:** recordedBy: J.Jakovlev and J.Penttinen; individualCount: 1; sex: male; **Location:** stateProvince: Lapponia enontekiensis; municipality: Enontekiö; locality: Kilpisjärvi_Saana_North_4; decimalLatitude: 69.045; decimalLongitude: 20.808; geodeticDatum: WGS84; **Identification:** identifiedBy: J.Jakovlev; **Event:** samplingProtocol: Malaise trap; eventDate: 2006-8-1/8-15; habitat: subarctic
- **Type status:** Other material. **Occurrence:** recordedBy: J.Jakovlev and J.Penttinen; individualCount: 1; sex: male; **Location:** stateProvince: Lapponia kemensis pars occidentalis; municipality: Kittilä; locality: Pallas-Yllästunturi National Park Pallas_1; decimalLatitude: 68.024; decimalLongitude: 24.150; geodeticDatum: WGS84; **Identification:** identifiedBy: J.Jakovlev; **Event:** samplingProtocol: Malaise trap; eventDate: 2006-7-15/8-14; habitat: old-growth forest, Myrtillus type
- **Type status:** Other material. **Occurrence:** recordedBy: J.Salmela; individualCount: 1; sex: male; **Location:** stateProvince: Lapponia inarensis; municipality: Utsjoki; locality: Galddasjohka; decimalLatitude: 69.860; decimalLongitude: 27.770; geodeticDatum: WGS84; **Identification:** identifiedBy: J.Jakovlev; **Event:** samplingProtocol: Malaise trap; eventDate: 2007-7-19/8-27; habitat: subarctic; **Record Level:** institutionCode: JJH
- **Type status:** Other material. **Occurrence:** recordedBy: J.Salmela; individualCount: 1; sex: male; **Location:** stateProvince: Lapponia inarensis; municipality: Utsjoki; locality: Galddasjohka; decimalLatitude: 69.860; decimalLongitude: 27.808; geodeticDatum: WGS84; **Identification:** identifiedBy: J.Jakovlev; **Event:** samplingProtocol: Malaise trap; eventDate: 2007-6-15/7-19; habitat: subarctic; **Record Level:** institutionCode: JJH
- **Type status:** Other material. **Occurrence:** recordedBy: J.Salmela; individualCount: 1; sex: male; **Location:** stateProvince: Lapponia inarensis; municipality: Utsjoki; locality: Galddasjohka; decimalLatitude: 69.861; decimalLongitude: 27.790; geodeticDatum: WGS84; **Identification:** identifiedBy: J.Jakovlev; **Event:** samplingProtocol: Malaise trap; eventDate: 2007-7-19/8-27; habitat: subarctic; **Record Level:** institutionCode: JJH
- **Type status:** Other material. **Occurrence:** recordedBy: Jukka Salmela; individualCount: 1; sex: male; **Location:** country: Finland; stateProvince: Lapponia kemensis pars orientalis; verbatimLocality: Savukoski, Joutenoja; decimalLatitude: 67.821; decimalLongitude: 29.440; geodeticDatum: WGS84; **Identification:** identifiedBy: J. Salmela; **Event:** samplingProtocol: Malaise trap; eventDate: 2012-7-10/8-16; habitat: Headwater stream, boreal forest
- **Type status:** Other material. **Occurrence:** catalogNumber: MYCE-JS-2013-0131; recordedBy: Jukka Salmela; individualCount: 1; sex: male; **Location:** country: Finland; stateProvince: Lapponia kemensis pars orientalis; verbatimLocality: Savukoski, Törmäoja; decimalLatitude: 67.846; decimalLongitude: 29.471; geodeticDatum: WGS84; **Identification:** identifiedBy: J. Salmela; **Event:** samplingProtocol: Malaise trap; eventDate: 2012-7-10/8-16; habitat: Headwater stream, boreal forest; **Record Level:** institutionCode: JES
- **Type status:** Other material. **Occurrence:** catalogNumber: MYCE-NV-2013-0259; recordedBy: Jukka Salmela; individualCount: 1; sex: male; **Location:** country: Finland; stateProvince: Lapponia kemensis pars occidentalis; verbatimLocality: Kittilä, Tarpomapää; decimalLatitude: 67.820; decimalLongitude: 25.919; geodeticDatum: WGS84; **Identification:** identifiedBy: J. Salmela; **Event:** samplingProtocol: Malaise trap; eventDate: 2009-6-1/29; habitat: Spring brook, boreal forest; **Record Level:** institutionCode: JES
- **Type status:** Other material. **Occurrence:** recordedBy: J. Salmela; T. Hietajärvi; individualCount: 1; sex: male; **Location:** country: Finland; stateProvince: Regio kuusamoensis; verbatimLocality: Salla, Kuntasjoki, Värriö Strict Nature Reserve; verbatimElevation: 320 m; decimalLatitude: 67.749; decimalLongitude: 29.617; geodeticDatum: WGS84; **Identification:** identifiedBy: J. Salmela; **Event:** samplingProtocol: Malaise trap; eventDate: 2013; verbatimEventDate: 2013-6-4/29; habitat: headwater stream, old-growth boreal forest

#### Distribution

Palaearctic. Widely distributed in Europe (Chandler 2004). In the Nordic Region recorded from Norway (Gammelmo and Søli 2006), northern and central Sweden (Kjaerandsen et al. 2007), and Finland (Lundström 1912a, as *Boletina winnertzii*). Most of the Finnish records are from the northern part of the country.

#### Ecology

Recently collected Finnish material is from riparian forests, boreal forests and mountain birch forests. Immature stages are unknown.

### Boletina dubia

(Meigen, 1804)

http://www.faunaeur.org/full_results.php?id=140816

#### Materials

- **Type status:** Other material. **Occurrence:** catalogNumber: MYCE-JS-2012-0022; recordedBy: J. Salmela; individualCount: 1; sex: male; **Location:** country: Finland; stateProvince: Lapponia kemensis pars orientalis; verbatimLocality: Sodankylä, Heinäaapa; decimalLatitude: 67.596; decimalLongitude: 26.883; geodeticDatum: WGS84; **Identification:** identifiedBy: J. Salmela; **Event:** samplingProtocol: Malaise trap; eventDate: 2012-8-10/9-19; habitat: rich srping fen; **Record Level:** institutionCode: JES
- **Type status:** Other material. **Occurrence:** catalogNumber: MYCE-JS-2012-0088; recordedBy: J. Salmela; individualCount: 1; sex: male; **Location:** country: Finland; stateProvince: Lapponia kemensis pars orientalis; verbatimLocality: Sodankylä, Heinäaapa; decimalLatitude: 67.596; decimalLongitude: 26.883; geodeticDatum: WGS84; **Identification:** identifiedBy: J. Salmela; **Event:** samplingProtocol: Malaise trap; eventDate: 2012-6-6/7-6; habitat: rich srping fen; **Record Level:** institutionCode: JES
- **Type status:** Other material. **Occurrence:** catalogNumber: MYCE-JS-2012-0034; recordedBy: J. Salmela; individualCount: 1; sex: male; **Location:** country: Finland; stateProvince: Ostrobotnia borealis pars borealis; verbatimLocality: Tornio, Rakanjänkkä; decimalLatitude: 65.889; decimalLongitude: 24.317; geodeticDatum: WGS84; **Identification:** identifiedBy: J. Salmela; **Event:** samplingProtocol: Malaise trap; eventDate: 2012-8-6/9-26; habitat: rich srping fen; **Record Level:** institutionCode: JES
- **Type status:** Other material. **Occurrence:** catalogNumber: MYCE-JS-2013-0359; recordedBy: J. Salmela; individualCount: 1; sex: male; **Location:** country: Finland; stateProvince: Regio kuusamoensis; verbatimLocality: Salla, Iso Pyhätunturi; decimalLatitude: 66.776; decimalLongitude: 28.810; geodeticDatum: WGS84; **Identification:** identifiedBy: J. Salmela; **Event:** samplingProtocol: Malaise trap; eventDate: 2013-8-8/9-19; habitat: intermediate rich sloping fen; **Record Level:** institutionCode: JES
- **Type status:** Other material. **Occurrence:** catalogNumber: MYCE-NV-2013-0062; recordedBy: J. Salmela; individualCount: 1; sex: male; **Location:** country: Finland; stateProvince: Lapponia kemensis pars occidentalis; verbatimLocality: Kittilä, Silmäsvuoma; decimalLatitude: 67.582; decimalLongitude: 25.543; geodeticDatum: WGS84; **Identification:** identifiedBy: J. Salmela; **Event:** samplingProtocol: Malaise trap; eventDate: 2007-5-31/6-25; habitat: rich fen; **Record Level:** institutionCode: JES
- **Type status:** Other material. **Occurrence:** catalogNumber: MYCE-NV-2013-0085; recordedBy: J. Salmela; individualCount: 1; sex: male; **Location:** country: Finland; stateProvince: Lapponia kemensis pars occidentalis; verbatimLocality: Kittilä, Kielisenpalo; decimalLatitude: 68.020; decimalLongitude: 25.063; geodeticDatum: WGS84; **Identification:** identifiedBy: N. Vartija; **Event:** samplingProtocol: Malaise trap; eventDate: 2007-8-1/31; habitat: rich spring fen; **Record Level:** institutionCode: JES
- **Type status:** Other material. **Occurrence:** recordedBy: J. Salmela; individualCount: 1; sex: male; **Location:** country: Finland; stateProvince: Regio aboensis; verbatimLocality: Turku, Pomponrahka; decimalLatitude: 60.509; decimalLongitude: 22.249; geodeticDatum: WGS84; **Identification:** identifiedBy: N. Vartija; **Event:** samplingProtocol: Malaise trap; eventDate: 2011-5-3/6-1; habitat: rich fen
- **Type status:** Other material. **Occurrence:** recordedBy: R. Frey; individualCount: 1; sex: male; **Location:** country: Finland; stateProvince: Nylandia; verbatimLocality: Espoo; decimalLatitude: 60.19; decimalLongitude: 24.64; geodeticDatum: WGS84; **Identification:** identifiedBy: J. Jakovlev; **Event:** eventDate: 1906; **Record Level:** institutionCode: MZHF
- **Type status:** Other material. **Occurrence:** recordedBy: J. Salmela; individualCount: 1; sex: male; **Location:** country: Finland; stateProvince: Lapponia inarensis; verbatimLocality: Utsjoki, Galddasjohka; decimalLatitude: 69.860; decimalLongitude: 27.809; geodeticDatum: WGS84; **Identification:** identifiedBy: J. Jakovlev; **Event:** samplingProtocol: Malaise trap; eventDate: 2007-7-19/8-27; habitat: headwater stream; **Record Level:** institutionCode: JJH

#### Distribution

European. Widely distributed in Europe (Chandler 2004). In the Nordic Region recorded from Norway (Gammelmo and Søli 2006), Sweden (Kjaerandsen et al. 2007), Iceland (Kjaerandsen et al. 2007a) and Finland (Lundström 1912b, as *Boletina inermis*, holotype male from Nylandia, Helsinki). Wide range in Finland, including the hemiboreal, boreal and subarctic zones.

#### Ecology

*Boletina dubia* has been reared from liverworts (Cheetham 1920). New Finnish records are mainly from rich fens, and there are single records from a *Sphagnum* dominated sloping fen and from a headwater stream with rich riparian vegetation.

#### Conservation

Due to the scarcity of records until the 2010 Red List assessment, the species was considered to be rather rare in Finland. However, the species was obviously overlooked due to its mire-dwelling ecology. *Boletina dubia* is currently red-listed in Finland (NT, Penttinen et al. 2010), but the new data provided here indicate that the species is actually rather common in suitable habitats in the middle and north boreal mires.

### Boletina dispectoides

Jakovlev & Penttinen, 2007**

http://www.catalogueoflife.org/annual-checklist/2013/details/species/id/8765129

#### Materials

- **Type status:** Other material. **Occurrence:** recordedBy: A. Polevoi; individualCount: 1; sex: male; **Taxon:** genus: Boletina; specificEpithet: dispectoides; scientificNameAuthorship: Jakovlev & Penttinen, 2007; **Location:** country: Russia; stateProvince: Republic Karelia; verbatimLocality: Kivach Nature Reserve; decimalLatitude: 62.272; decimalLongitude: 33.988; geodeticDatum: WGS84; **Identification:** identifiedBy: A. Polevoi; **Event:** samplingProtocol: Malaise trap; eventDate: 1990-9-18/10-1; **Record Level:** institutionCode: FRIP

#### Distribution

European. So far was known only from the type locality in Finland (Jakovlev and Penttinen 2007) and from the Italian Alps (Kurina 2008) but might be overlooked in the Fennoscandian region. New to Russia and Karelia.

#### Ecology

The Karelian specimen was collected in herb-rich aspen dominated forest. Immature stages are unknown.

### Boletina groenlandica

Staeger, 1845

http://www.faunaeur.org/full_results.php?id=140828

#### Materials

- **Type status:** Other material. **Occurrence:** catalogNumber: MYCE-JS-2012-0082; recordedBy: J. Salmela; individualCount: 2; sex: male; **Location:** country: Finland; stateProvince: Lapponia kemensis pars orientalis; verbatimLocality: Sodankylä, Heinäaapa; decimalLatitude: 67.596; decimalLongitude: 26.883; geodeticDatum: WGS84; **Identification:** identifiedBy: J. Salmela; **Event:** samplingProtocol: Malaise trap; eventDate: 2012-6-6/7-6; habitat: rich spring fen; **Record Level:** institutionCode: JES
- **Type status:** Other material. **Occurrence:** catalogNumber: MYCE-JS-2013-0135; recordedBy: J. Salmela; individualCount: 1; sex: male; **Location:** country: Finland; stateProvince: Lapponia kemensis pars orientalis; verbatimLocality: Savukoski, Törmäoja; decimalLatitude: 67.846; decimalLongitude: 29.471; geodeticDatum: WGS84; **Identification:** identifiedBy: J. Salmela; **Event:** samplingProtocol: Malaise trap; eventDate: 2012-6-14/7-10; habitat: headwater stream; **Record Level:** institutionCode: JES
- **Type status:** Other material. **Occurrence:** catalogNumber: MYCE-JS-2013-0215; recordedBy: J. Salmela; individualCount: 1; sex: male; **Location:** country: Finland; stateProvince: Lapponia kemensis pars orientalis; verbatimLocality: Sodankylä, Kaita-aapa; decimalLatitude: 67.845; decimalLongitude: 26.553; geodeticDatum: WGS84; **Identification:** identifiedBy: J. Salmela; **Event:** samplingProtocol: Malaise trap; eventDate: 2012-6-5/7-3; habitat: aapamire, intermediate rich flark fen; **Record Level:** institutionCode: JES
- **Type status:** Other material. **Occurrence:** catalogNumber: MYCE-NV-2013-0041; recordedBy: J. Salmela; individualCount: 1; sex: male; **Location:** country: Finland; stateProvince: Lapponia kemensis pars orientalis; verbatimLocality: Sodankylä, Pomokaira, Paistipuolet; decimalLatitude: 67.836; decimalLongitude: 26.216; geodeticDatum: WGS84; **Identification:** identifiedBy: N. Vartija; J. Salmela; **Event:** samplingProtocol: Malaise trap; eventDate: 2009-6-1/29; habitat: poor sloping fen; **Record Level:** institutionCode: JES
- **Type status:** Other material. **Occurrence:** catalogNumber: MYCE-NV-2013-0104; recordedBy: J. Salmela; individualCount: 1; sex: male; **Location:** country: Finland; stateProvince: Lapponia kemensis pars orientalis; verbatimLocality: Sodankylä, Pomokaira, Aittakumpu S; decimalLatitude: 67.822; decimalLongitude: 26.027; geodeticDatum: WGS84; **Identification:** identifiedBy: N. Vartija; J. Salmela; **Event:** samplingProtocol: Malaise trap; eventDate: 2009-6-1/29; habitat: rich fen; **Record Level:** institutionCode: JES
- **Type status:** Other material. **Occurrence:** catalogNumber: MYCE-NV-2013-0139; recordedBy: J. Salmela; individualCount: 11; sex: male; **Location:** country: Finland; stateProvince: Lapponia kemensis pars orientalis; verbatimLocality: Sodankylä, Pomokaira, Tarpomapää E; decimalLatitude: 67.831; decimalLongitude: 25.993; geodeticDatum: WGS84; **Identification:** identifiedBy: N. Vartija; J. Salmela; **Event:** samplingProtocol: Malaise trap; eventDate: 2009-6-1/29; habitat: intermediate rich flark fen; **Record Level:** institutionCode: JES
- **Type status:** Other material. **Occurrence:** catalogNumber: MYCE-NV-2013-0194; recordedBy: J. Salmela; individualCount: 20; sex: male; **Location:** country: Finland; stateProvince: Lapponia kemensis pars orientalis; verbatimLocality: Sodankylä, Pomokaira, Paistipuolet NE; decimalLatitude: 67.834; decimalLongitude: 26.270; geodeticDatum: WGS84; **Identification:** identifiedBy: N. Vartija; J. Salmela; **Event:** samplingProtocol: Malaise trap; eventDate: 2009-6-1/29; habitat: intermediate rich spring fen; **Record Level:** institutionCode: JES
- **Type status:** Other material. **Occurrence:** catalogNumber: MYCE-NV-2013-0046; recordedBy: J. Salmela; individualCount: 1; sex: male; **Location:** country: Finland; stateProvince: Lapponia kemensis pars occidentalis; verbatimLocality: Kittilä, Repsuvuoma; decimalLatitude: 67.604; decimalLongitude: 24.967; geodeticDatum: WGS84; **Identification:** identifiedBy: N. Vartija; J. Salmela; **Event:** samplingProtocol: Malaise trap; eventDate: 2007-6-1/26; habitat: rich fen; **Record Level:** institutionCode: JES
- **Type status:** Other material. **Occurrence:** catalogNumber: MYCE-NV-2013-0111; recordedBy: J. Salmela; individualCount: 4; sex: male; **Location:** country: Finland; stateProvince: Lapponia kemensis pars occidentalis; verbatimLocality: Kittilä, Vuotsonperänjänkä; decimalLatitude: 67.616; decimalLongitude: 25.449; geodeticDatum: WGS84; **Identification:** identifiedBy: N. Vartija; J. Salmela; **Event:** samplingProtocol: Malaise trap; eventDate: 2007-5-31/6-25; habitat: rich fen; **Record Level:** institutionCode: JES
- **Type status:** Other material. **Occurrence:** catalogNumber: MYCE-NV-2013-0258; recordedBy: J. Salmela; individualCount: 2; sex: male; **Location:** country: Finland; stateProvince: Lapponia kemensis pars occidentalis; verbatimLocality: Kittilä, Pomokaira, Tarpomapää; decimalLatitude: 67.820; decimalLongitude: 25.919; geodeticDatum: WGS84; **Identification:** identifiedBy: N. Vartija; J. Salmela; **Event:** samplingProtocol: Malaise trap; eventDate: 2009-6-1/29; habitat: spring brook, old-growth boreal forest; **Record Level:** institutionCode: JES
- **Type status:** Other material. **Occurrence:** catalogNumber: DIPT-JS-2014-00; recordedBy: J. Salmela; T. Hietajärvi; individualCount: 1; sex: male; **Location:** country: Finland; stateProvince: Regio kuusamoensis; verbatimLocality: Salla, Kuntasjoki, Värriö Strict Nature Reserve; verbatimElevation: 320 m; decimalLatitude: 67.749; decimalLongitude: 29.617; geodeticDatum: WGS84; **Identification:** identifiedBy: J. Salmela; **Event:** samplingProtocol: Malaise trap; eventDate: 2013; verbatimEventDate: 2013-6-4/29; habitat: headwater stream, old-growth boreal forest; **Record Level:** institutionCode: JES

#### Distribution

**Figure 12.**
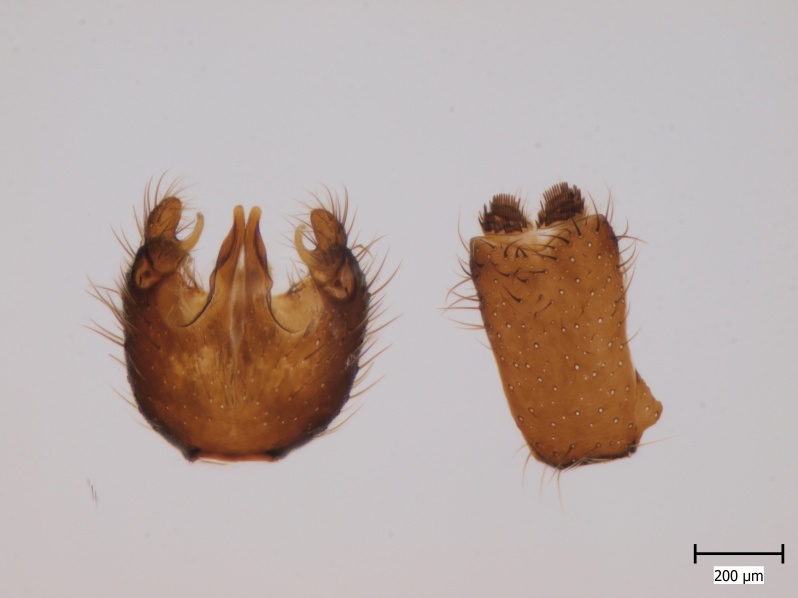
*Boletina groenlandica* Staeger, male specimen collected from Finland, Sodankylä (central Lapland). Male hypopygium (left, ventral view) and 9th tergite (right, dorsal view).

Holarctic. *Boletina groenlandica* (Fig. 12) was described from Greenland, and displays a northwestern distribution in Europe, including Great Britain, Germany, Latvia, Norway, Sweden, Finland and northwest Russia (Chandler 2004, Kjaerandsen 2012). British records are only from montane habitats in Scotland, mainly by streams above the tree line (Falk and Chandler 2005). All former Finnish records are old (1911, leg. R Frey) and originate from NW Finnish Lapland (Kittilä and Muonio, Lundström 1912b). Old records from NW Russia originate from Murmansk region (Kuzomen and Kandalaksha) and one recent record from Pasvik Nature Reserve (Polevoi 2010). New Finnish records presented here are from the north boreal zone.

#### Ecology

New Finnish records are mainly from aapamires, including both poor and rich fens. Some of the specimens were taken from the vicinity of running water. Immature stages are unknown.

### Boletina intermedia

Lundström, 1915*

http://www.faunaeur.org/full_results.php?id=140831

#### Materials

- **Type status:** Other material. **Occurrence:** catalogNumber: MYCE-JS-2012-0079; recordedBy: J. Salmela; individualCount: 1; sex: male; **Location:** country: Finland; stateProvince: Lapponia kemensis pars orientalis; verbatimLocality: Sodankylä, Heinäaapa; decimalLatitude: 67.596; decimalLongitude: 26.883; geodeticDatum: WGS84; **Identification:** identifiedBy: J. Salmela; **Event:** samplingProtocol: Malaise trap; eventDate: 2012-6-6/7-6; habitat: rich spring fen; **Record Level:** institutionCode: JES
- **Type status:** Other material. **Occurrence:** catalogNumber: MYCE-JS-2013-0217; recordedBy: J. Salmela; individualCount: 1; sex: male; **Location:** country: Finland; stateProvince: Lapponia kemensis pars orientalis; verbatimLocality: Sodankylä, Kaita-aapa; decimalLatitude: 67.845; decimalLongitude: 26.553; geodeticDatum: WGS84; **Identification:** identifiedBy: J. Salmela; **Event:** samplingProtocol: Malaise trap; eventDate: 2012-6-5/7-3; habitat: intermediate rich fen, aapamire; **Record Level:** institutionCode: JES
- **Type status:** Other material. **Occurrence:** catalogNumber: MYCE-JS-2013-0187; recordedBy: J. Salmela; individualCount: 1; sex: male; **Location:** country: Finland; stateProvince: Lapponia kemensis pars orientalis; verbatimLocality: Savukoski, Joutenoja; decimalLatitude: 67.821; decimalLongitude: 29.440; geodeticDatum: WGS84; **Identification:** identifiedBy: J. Salmela; **Event:** samplingProtocol: Malaise trap; eventDate: 2012-6-14/7-10; habitat: headwater stream, seminatural boreal forest; **Record Level:** institutionCode: JES
- **Type status:** Other material. **Occurrence:** catalogNumber: DIPT-JS-2014-0013; recordedBy: J. Salmela; T. Hietajärvi; individualCount: 1; sex: male; **Location:** country: Finland; stateProvince: Regio kuusamoensis; verbatimLocality: Salla, Kuntasjoki, Värriö Strict Nature Reserve; verbatimElevation: 320 m; decimalLatitude: 67.749; decimalLongitude: 29.617; geodeticDatum: WGS84; **Identification:** identifiedBy: J. Salmela; **Event:** samplingProtocol: Malaise trap; eventDate: 2013; verbatimEventDate: 2013-6-4/29; habitat: headwater stream, old-growth boreal forest; **Record Level:** institutionCode: JES

#### Distribution

Palaearctic. *Boletina intermedia* (Fig. 11b) is a poorly-known fungus gnat, described from Russia, New Siberian Islands (Lundström 1915). In Europe there is a previous record from Germany (Plassmann and Joost 1986) here reported for the first time from Fennoscandia. Finnish records are from the north boreal zone, central (Sodankylä) and eastern (Salla, Savukoski) Lapland.

#### Ecology

Collected from fens (an intermediate rich flark fen and a rich spring fen) and in the vicinity of a headwater stream. Immature stages are unknown.

#### Taxon discussion

See *Boletina borealis* Zetterstedt.

### Boletina kivachiana

Polevoi & Hedmark, 2004

http://www.faunaeur.org/full_results.php?id=140834

#### Materials

- **Type status:** Other material. **Occurrence:** catalogNumber: MYCE-JS-2013-0058; recordedBy: J. Salmela; individualCount: 1; sex: male; **Location:** country: Finland; stateProvince: Lapponia kemensis pars orientalis; verbatimLocality: Savukoski, Joutenoja; decimalLatitude: 67.821; decimalLongitude: 29.440; geodeticDatum: WGS84; **Identification:** identifiedBy: J. Salmela; **Event:** samplingProtocol: Malaise trap; eventDate: 2012-8-16/9-18; habitat: headwater stream, seminatural boreal forest; **Record Level:** institutionCode: JES
- **Type status:** Other material. **Occurrence:** catalogNumber: MYCE-JS-2013-0107; recordedBy: J. Salmela; individualCount: 1; sex: male; **Location:** country: Finland; stateProvince: Lapponia kemensis pars orientalis; verbatimLocality: Savukoski, Törmäoja; decimalLatitude: 67.846; decimalLongitude: 29.471; geodeticDatum: WGS84; **Identification:** identifiedBy: J. Salmela; **Event:** samplingProtocol: Malaise trap; eventDate: 2012-6-14/7-10; habitat: headwater stream, old-growth boreal forest; **Record Level:** institutionCode: JES
- **Type status:** Other material. **Occurrence:** catalogNumber: MYCE-JS-2013-0353; recordedBy: J. Salmela; individualCount: 1; sex: male; **Location:** country: Finland; stateProvince: Lapponia kemensis pars orientalis; verbatimLocality: Savukoski, Törmäoja, Ahot; decimalLatitude: 67.827; decimalLongitude: 29.435; geodeticDatum: WGS84; **Identification:** identifiedBy: J. Salmela; **Event:** samplingProtocol: Malaise trap; eventDate: 2013-8-7/9-19; habitat: swampy meadow with Carex tussocks; **Record Level:** institutionCode: JES
- **Type status:** Other material. **Occurrence:** recordedBy: A. Humala; individualCount: 3; sex: male; **Location:** country: Russia; stateProvince: Republic Karelia; verbatimLocality: White Sea, island Kondostrov; decimalLatitude: 64.224; decimalLongitude: 36.622; geodeticDatum: WGS84; **Identification:** identifiedBy: A. Polevoi; **Event:** samplingProtocol: Sweep netting; eventDate: 2002-8-21; **Record Level:** institutionCode: FRIP
- **Type status:** Other material. **Occurrence:** recordedBy: A. Polevoi; individualCount: 21; sex: male; **Location:** country: Russia; stateProvince: Republic Karelia; verbatimLocality: Shun'ga, Turastamozero; decimalLatitude: 62.56; decimalLongitude: 34.706; geodeticDatum: WGS84; **Identification:** identifiedBy: A. Polevoi; **Event:** samplingProtocol: Malaise trap; eventDate: 2012-7-24/8-24; **Record Level:** institutionCode: FRIP
- **Type status:** Other material. **Occurrence:** recordedBy: J.Jakovlev; individualCount: 1; sex: male; **Location:** country: Finland; stateProvince: Tavastia australis; municipality: Lammi; locality: Evo_Kotinen_Aspen part; decimalLatitude: 61.244; decimalLongitude: 25.067; geodeticDatum: WGS84; **Identification:** identifiedBy: J.Jakovlev; **Event:** samplingProtocol: Malaise trap; eventDate: 2004-7-27/8-27; habitat: old-growth forest, herb-rich type
- **Type status:** Other material. **Occurrence:** recordedBy: J.Penttinen; individualCount: 1; sex: male; **Location:** country: Finland; stateProvince: Savonia australis; municipality: Rantasalmi; locality: Linnansaari; decimalLatitude: 62.114; decimalLongitude: 28.479; geodeticDatum: WGS84; **Identification:** identifiedBy: J.Penttinen; **Event:** samplingProtocol: Malaise trap; eventDate: 2008-5-20/6-24; habitat: old-growth forest, herb-rich type; **Record Level:** institutionCode: JPJ
- **Type status:** Other material. **Occurrence:** recordedBy: J.Jakovlev; individualCount: 1; sex: male; **Location:** country: Finland; stateProvince: Nylandia; municipality: Sipoo; locality: Sipoonkorpi_1; decimalLatitude: 60.322; decimalLongitude: 25.157; geodeticDatum: WGS84; **Identification:** identifiedBy: J.Jakovlev; **Event:** samplingProtocol: Malaise trap; eventDate: 2008-7-21/-8-6; habitat: old managed forest, herb-rich type
- **Type status:** Other material. **Occurrence:** recordedBy: J.Penttinen; individualCount: 1; sex: male; **Location:** country: Finland; stateProvince: Tavastia australis; municipality: Lammi; locality: Reväsvuori 2/1; decimalLatitude: 61.070; decimalLongitude: 25.070; geodeticDatum: WGS84; **Identification:** identifiedBy: J.Penttinen; **Event:** samplingProtocol: Malaise trap; eventDate: 2009-9-30; habitat: old managed forest, herb-rich type; **Record Level:** institutionCode: JPJ
- **Type status:** Other material. **Occurrence:** catalogNumber: DIPT-JS-2014-0035; recordedBy: J. Salmela; T. Hietajärvi; individualCount: 1; sex: male; **Location:** country: Finland; stateProvince: Regio kuusamoensis; verbatimLocality: Salla, Kuntasjoki, Värriö Strict Nature Reserve; verbatimElevation: 320 m; decimalLatitude: 67.749; decimalLongitude: 29.617; geodeticDatum: WGS84; **Identification:** identifiedBy: J. Salmela; **Event:** samplingProtocol: Malaise trap; eventDate: 2013; verbatimEventDate: 2013-6-29/7-29; habitat: headwater stream, old-growth boreal forest; **Record Level:** institutionCode: JES

#### Distribution

European. Described from Russian Karelia and northern Finland (Polevoi and Hedmark 2004), also found in Scotland (Edwards 1925, as *Boletina nigrofusca*), the Italian Alps (Kurina 2008), northern Sweden (Kjaerandsen et al. 2007), and northern Norway (Søli and Rindal 2012).

#### Ecology

In Fennoscandia it is a characteristic species of old-growth boreal forests. In Scotland recorded from wet native woodland (Falk and Chandler 2005) and from a broad-leaved forest (P. Chandler, pers.comm.). Immature stages are unknown.

#### Conservation

Red-listed in Finland (VU, Penttinen et al. 2010).

### Boletina jamalensis

Zaitzev, 1994

http://www.faunaeur.org/full_results.php?id=140832

#### Materials

- **Type status:** Holotype. **Occurrence:** recordedBy: L.Tiensuu; individualCount: 1; sex: male; **Taxon:** scientificName: Boletina jamalensis; originalNameUsage: Boletina struthioides; nameAccordingTo: Polevoi, A.V. 2013. On the systematics and distribution of some poorly known species of Boletina (Diptera: Mycetophilidae) in northern Europe, with the description of a new species. Zoosystematica Rossica 22: 114-122.; **Location:** country: Finland; stateProvince: Lapponia enontekiensis; municipality: Enontekiö; locality: Kilpisjärvi; decimalLatitude: 69.044; decimalLongitude: 20.800; geodeticDatum: WGS84; **Identification:** identifiedBy: A.Polevoi; **Event:** samplingProtocol: Unknown method; eventDate: 1970-8-16/8-16; habitat: subarctic; **Record Level:** institutionCode: MZHF

#### Distribution

Palaearctic. Described from the Jamal Peninsula (Zaitzev 1994), later recorded as *Boletina struthioides* from northern areas of Russian Karelia, Finland (Kilpisjärvi) and Sweden (Vuollerim) (Polevoi and Hedmark 2004). The species *Boletina struthioides* Polevoi & Hedmark, 2004 was considered a junior synonym of *Boletina jamalensis* Zaitzev, 1994 by Polevoi (Polevoi 2013b).

#### Ecology

Karelian specimens were collected in mixed forests and adjacent meadows (Polevoi 2013b). Immature stages are unknown.

### Boletina kurilensis

Zaitzev, 1994

http://www.faunaeur.org/full_results.php?id=140836

#### Materials

- **Type status:** Other material. **Occurrence:** recordedBy: J.Salmela; individualCount: 1; sex: male; **Location:** country: Finland; stateProvince: Lapponia inarensis; municipality: Utsjoki; locality: Galddasjohka; decimalLatitude: 69.861; decimalLongitude: 27.790; geodeticDatum: WGS84; **Identification:** identifiedBy: J.Jakovlev; **Event:** samplingProtocol: Malaise trap; eventDate: 2007-7-19/8-27; habitat: subarctic stream valley; **Record Level:** institutionCode: JJH
- **Type status:** Other material. **Occurrence:** catalogNumber: MYCE-JS-2013-0150; recordedBy: J. Salmela; individualCount: 1; sex: male; **Location:** country: Finland; stateProvince: Lapponia kemensis pars orientalis; verbatimLocality: Savukoski, Törmäoja; decimalLatitude: 67.835; decimalLongitude: 29.454; geodeticDatum: WGS84; **Identification:** identifiedBy: J. Salmela; **Event:** samplingProtocol: Malaise trap; eventDate: 2012-8-16/9-18; habitat: headwater stream, old-growth boreal forest; **Record Level:** institutionCode: JES
- **Type status:** Other material. **Occurrence:** catalogNumber: MYCE-JS-2013-0117; recordedBy: J. Salmela; individualCount: 1; sex: male; **Location:** country: Finland; stateProvince: Lapponia kemensis pars orientalis; verbatimLocality: Savukoski, Joutenoja; decimalLatitude: 67.821; decimalLongitude: 29.440; geodeticDatum: WGS84; **Identification:** identifiedBy: J. Salmela; **Event:** samplingProtocol: Malaise trap; eventDate: 2012-8-16/9-18; habitat: headwater stream, seminatural boreal forest; **Record Level:** institutionCode: JES

#### Distribution

Palaearctic, described from Kuril Islands (Zaitzev 1994). In Europe, according to Polevoi (Polevoi 2013b) found only in the northernmost areas of northwest Russia (Murmansk Region), Finland (subarctic zone, Kilpisjärvi) and Norway (Finnmark). Former records from Russian Karelia (Jakovlev and Polevoi 1991, Jakovlev and Polevoi 1997, Polevoi 2000, Polevoi 2010), Finland (Jakovlev et al. 2006) and Sweden (Kjaerandsen et al. 2007), in fact belong to the recently described *Boletina palmata* Polevoi, 2013. Here reported from the boreal forest zone, Savukoski (eastern Lapland).

#### Ecology

Immature stages are unknown. Adults have been collected around lotic waters in a subarctic fell area (Utsjoki) and the coniferous zone (Savukoski), and also from mountain birch forests in Finland and Murmansk region (Polevoi 2013b).

### Boletina landrocki

Edwards, 1924**

http://www.faunaeur.org/full_results.php?id=140837

#### Materials

- **Type status:** Other material. **Occurrence:** recordedBy: A. Polevoi; individualCount: 2; sex: male; **Location:** country: Russia; stateProvince: Leningrad province; verbatimLocality: Voznesenje, 1 km SE of Gimreka; decimalLatitude: 61.151; decimalLongitude: 35.642; geodeticDatum: WGS84; **Identification:** identifiedBy: A. Polevoi; **Event:** samplingProtocol: Malaise trap; eventDate: 2008-8-27/10-1; **Record Level:** institutionCode: FRIP
- **Type status:** Other material. **Occurrence:** recordedBy: J.Jakovlev; individualCount: 1; sex: male; **Location:** country: Finland; stateProvince: Tavastia australis; municipality: Lammi; locality: Evo_Hattukivenmaa; decimalLatitude: 61.207; decimalLongitude: 25.153; geodeticDatum: WGS84; **Identification:** identifiedBy: J.Jakovlev; **Event:** samplingProtocol: Malaise trap; eventDate: 2004-7-27/8-27; habitat: old managed forest, Myrtillus type
- **Type status:** Other material. **Occurrence:** recordedBy: J.Jakovlev; individualCount: 2; sex: male; **Location:** country: Finland; stateProvince: Nylandia; municipality: Sipoo; locality: Käsis-Solbacka; decimalLatitude: 60.445; decimalLongitude: 25.193; geodeticDatum: WGS84; **Identification:** identifiedBy: J.Jakovlev; **Event:** samplingProtocol: Malaise trap; eventDate: 2005-5-13/6-13; habitat: young unmanaged forest; **Record Level:** institutionCode: JJH
- **Type status:** Other material. **Occurrence:** catalogNumber: MYCE-JS-2012-0068; recordedBy: J. Salmela; individualCount: 1; sex: male; **Location:** country: Finland; stateProvince: Lapponia kemensis pars orientalis; verbatimLocality: Savukoski, Joutenoja; decimalLatitude: 67.821; decimalLongitude: 29.440; geodeticDatum: WGS84; **Identification:** identifiedBy: J. Salmela; **Event:** samplingProtocol: Malaise trap; eventDate: 2012-8-16/9-18; habitat: headwater stream, seminatural boreal forest; **Record Level:** institutionCode: JES
- **Type status:** Other material. **Occurrence:** catalogNumber: MYCE-JS-2012-0025; recordedBy: J. Salmela; individualCount: 1; sex: male; **Location:** country: Finland; stateProvince: Lapponia kemensis pars orientalis; verbatimLocality: Savukoski, Törmäoja; decimalLatitude: 67.846; decimalLongitude: 29.471; geodeticDatum: WGS84; **Identification:** identifiedBy: J. Salmela; **Event:** samplingProtocol: Malaise trap; eventDate: 2012-8-16/9-18; habitat: headwater stream, seminatural boreal forest; **Record Level:** institutionCode: JES
- **Type status:** Other material. **Occurrence:** catalogNumber: MYCE-NV-2013-0117; recordedBy: J. Salmela; individualCount: 1; sex: male; **Location:** country: Finland; stateProvince: Lapponia kemensis pars occidentalis; verbatimLocality: Kittilä, Vuotsonperänjänkä; decimalLatitude: 67.616; decimalLongitude: 25.449; geodeticDatum: WGS84; **Identification:** identifiedBy: N. Vartija; J. Salmela; **Event:** samplingProtocol: Malaise trap; eventDate: 2007-8-2/9-3; habitat: rich fen; **Record Level:** institutionCode: JES
- **Type status:** Other material. **Occurrence:** catalogNumber: DIPT-JS-2014-0069; recordedBy: J. Salmela; T. Hietajärvi; individualCount: 1; sex: male; **Location:** country: Finland; stateProvince: Regio kuusamoensis; verbatimLocality: Salla, Kuntasjoki, Värriö Strict Nature Reserve; verbatimElevation: 320 m; decimalLatitude: 67.749; decimalLongitude: 29.617; geodeticDatum: WGS84; **Identification:** identifiedBy: J. Salmela; **Event:** samplingProtocol: Malaise trap; verbatimEventDate: 2013-7-29/9-19; habitat: headwater stream, old-growth boreal forest; **Record Level:** institutionCode: JES

#### Distribution

European. A rare species recorded from France (Chandler 2004) and Northern Europe: St. Petersburg (Zaitzev 1994), Finland, Estonia, Latvia (Chandler 2004), Scotland (Chandler 2006), Norway (Gammelmo and Søli 2006) and Sweden (Kjaerandsen et al. 2007). New to the Republic of Karelia.

#### Ecology

Finnish collecting sites in Lapland are a rich fen (Kittilä) and riparian forests (Savukoski). Karelian specimens were collected in secondary *Vaccinium myrtillus* type pinedominated forest. Immature stages are unknown.

### Boletina lapponica

Polevoi & Hedmark, 2004*

http://www.faunaeur.org/full_results.php?id=140838

#### Materials

- **Type status:** Other material. **Occurrence:** catalogNumber: MYCE-JS-2013-0141; recordedBy: J. Salmela; individualCount: 1; sex: male; **Location:** country: Finland; stateProvince: Lapponia kemensis pars orientalis; verbatimLocality: Savukoski, Joutenoja; decimalLatitude: 67.821; decimalLongitude: 29.440; geodeticDatum: WGS84; **Identification:** identifiedBy: J. Salmela; **Event:** samplingProtocol: Malaise trap; eventDate: 2012-7-10/8-16; habitat: headwater stream, seminatural boreal forest; **Record Level:** institutionCode: JES
- **Type status:** Other material. **Occurrence:** catalogNumber: MYCE-JS-2013-0112; recordedBy: J. Salmela; individualCount: 1; sex: male; **Location:** country: Finland; stateProvince: Lapponia kemensis pars orientalis; verbatimLocality: Savukoski, Joutenoja; decimalLatitude: 67.821; decimalLongitude: 29.440; geodeticDatum: WGS84; **Identification:** identifiedBy: J. Salmela; **Event:** samplingProtocol: Malaise trap; eventDate: 2012-8-16/9-18; habitat: headwater stream, seminatural boreal forest; **Record Level:** institutionCode: JES
- **Type status:** Other material. **Occurrence:** catalogNumber: MYCE-JS-2013-0357; recordedBy: J. Salmela; individualCount: 2; sex: male; **Location:** country: Finland; stateProvince: Lapponia kemensis pars orientalis; verbatimLocality: Savukoski, Törmäoja, Ahot; decimalLatitude: 67.827; decimalLongitude: 29.435; geodeticDatum: WGS84; **Identification:** identifiedBy: J. Salmela; **Event:** samplingProtocol: Malaise trap; eventDate: 2013-8-7/9-19; habitat: headwater stream, seminatural boreal forest; **Record Level:** institutionCode: JES

#### Distribution

**Figure 13.**
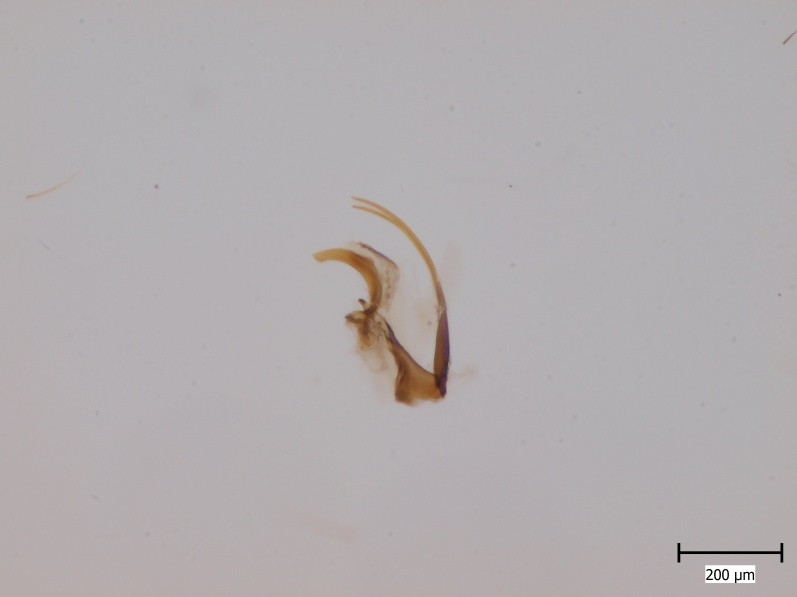
*Boletina lapponica* Polevoi & Hedmark, male specimen collected from Finland, Savukoski (eastern Lapland). Aedeagus and parameres, lateral view.

Fennoscandian. *Boletina lapponica* (Fig. 13) is known from northern Sweden (Lule Lappmark, Polevoi and Hedmark 2004, Kjaerandsen et al. 2007) and Russia (Republic Karelia, Kivach Nature Reserve, Polevoi and Hedmark 2004, Polevoi 2013a). New for Finland. Finnish records are from the north boreal zone, eastern Lapland (Savukoski).

#### Ecology

Finnish sampling localities are a headwater stream surrounded by boreal forest and a swampy meadow with *Carex* tussocks. Immature stages are unknown.

### Boletina maculata

Holmgren, 1870**

http://www.faunaeur.org/full_results.php?id=140841

#### Materials

- **Type status:** Other material. **Occurrence:** recordedBy: A. Polevoi; individualCount: 1; sex: male; **Location:** country: Russia; stateProvince: Republic Karelia; verbatimLocality: Paanajarvi, Leppälä; decimalLatitude: 66.272; decimalLongitude: 30.091; geodeticDatum: WGS84; **Identification:** identifiedBy: A. Polevoi; **Event:** samplingProtocol: Sweep netting; eventDate: 2000-7-3; **Record Level:** institutionCode: FRIP
- **Type status:** Other material. **Occurrence:** recordedBy: R. Frey; individualCount: 1; sex: male; **Location:** country: Finland; stateProvince: Lapponia kemensis pars orientalis; verbatimLocality: Pelkosenniemi; decimalLatitude: 67.15; decimalLongitude: 27.49; geodeticDatum: WGS84; **Identification:** identifiedBy: J. Jakovlev; **Event:** eventDate: 1911; eventRemarks: exact sampling locality unknown; **Record Level:** institutionCode: MZHF
- **Type status:** Other material. **Occurrence:** catalogNumber: MYCE-JS-2013-0396; recordedBy: J. Salmela; T. Hietajärvi; individualCount: 1; sex: male; **Location:** country: Finland; stateProvince: Regio kuusamoensis; verbatimLocality: Salla, Kuntasjoki, Värriö Strict Nature Reserve; verbatimElevation: 320 m; decimalLatitude: 67.749; decimalLongitude: 29.617; geodeticDatum: WGS84; **Identification:** identifiedBy: J. Salmela; **Event:** samplingProtocol: Malaise trap; eventDate: 2013; verbatimEventDate: 2013-6-4/29; habitat: headwater stream, old-growth boreal forest; **Record Level:** institutionCode: JES

#### Distribution

European. The species was described from Spitsbergen (Holmgren 1869) and has since been recorded in Europe from the Kola Peninsula in Russia (Lundström 1914, as *Boletina longicauda*, Zaitzev 1994), Sweden (Kjaerandsen et al. 2007) and Norway (Søli and Rindal 2012), and also from Latvia, Germany and Austria (Chandler 2004) indicating an arctic to boreal–mountainous distribution. Only three records exist so far from Finland, from Muonio (Lundström 1912b, as *Boletina longicauda*, leg. R Frey 1911), Pelkosenniemi and Salla, all from the north boreal zone. New to the Republic of Karelia.

#### Ecology

The Karelian specimen was collected in *Vaccinium myrtillus* type sprucedominated forest. The Finnish specimen from Salla was collected from a stream valley surrounded by old-growth boreal forest. Immature stages are unknown.

### Boletina palmata

Polevoi, 2013

#### Materials

- **Type status:** Other material. **Occurrence:** recordedBy: R.Frey; individualCount: 1; sex: male; **Location:** country: Finland; stateProvince: Lapponia inarensis; municipality: Utsjoki; locality: Utsjoki; decimalLatitude: 69.909; decimalLongitude: 27.021; geodeticDatum: WGS84; **Identification:** identifiedBy: A. Polevoi; **Event:** samplingProtocol: Unknown method; eventDate: 1913; habitat: subarctic; **Record Level:** institutionCode: MZHF
- **Type status:** Other material. **Occurrence:** recordedBy: J.Jakovlev; individualCount: 1; sex: male; **Location:** country: Finland; stateProvince: Tavastia australis; municipality: Lammi; locality: Evo_Lapinjärvi; decimalLatitude: 61.238; decimalLongitude: 25.087; geodeticDatum: WGS84; **Identification:** identifiedBy: J.Jakovlev; **Event:** samplingProtocol: Malaise trap; eventDate: 2004-8-28/10-4; habitat: burnt clear-cut; **Record Level:** institutionCode: JJH
- **Type status:** Other material. **Occurrence:** recordedBy: J.Penttinen; individualCount: 1; sex: male; **Location:** country: Finland; stateProvince: Satakunta; municipality: Rantasalmi; locality: Linnansaari; decimalLatitude: 62.116; decimalLongitude: 28.476; geodeticDatum: WGS84; **Identification:** identifiedBy: J.Penttinen; **Event:** samplingProtocol: Malaise trap; eventDate: 2008-5-20/6-24; habitat: old-growth forest, herb-rich type; **Record Level:** institutionCode: JPJ

#### Distribution

Fennoscandian. The type material originates from Northwest Russia: Murmansk Region (holotype) and Karelia (paratype), recorded as *Boletina kurilensis* Zaitzev from Russian Karelia, Finland, Norway and Sweden (Polevoi 2013b).

#### Ecology

Immature stages are unknown.

### Boletina pinusia

Maximova, 2001

http://www.catalogueoflife.org/col/details/species/id/8761104

#### Materials

- **Type status:** Other material. **Occurrence:** catalogNumber: MYCE-JS-2012-0092; recordedBy: J. Salmela; individualCount: 1; sex: male; **Location:** country: Finland; stateProvince: Lapponia kemensis pars orientalis; verbatimLocality: Sodankylä, Heinäaapa; decimalLatitude: 67.596; decimalLongitude: 26.883; geodeticDatum: WGS84; **Identification:** identifiedBy: J. Salmela; **Event:** samplingProtocol: Malaise trap; eventDate: 2012-6-6/7-6; habitat: rich spring fen; **Record Level:** institutionCode: JES
- **Type status:** Other material. **Occurrence:** catalogNumber: MYCE-JS-2012-0047; recordedBy: J. Salmela; individualCount: 1; sex: male; **Location:** country: Finland; stateProvince: Lapponia kemensis pars orientalis; verbatimLocality: Savuskoski, Joutenoja; decimalLatitude: 67.821; decimalLongitude: 29.440; geodeticDatum: WGS84; **Identification:** identifiedBy: J. Salmela; **Event:** samplingProtocol: Malaise trap; eventDate: 2012-7-10/8-16; habitat: headwater stream, seminatural boreal forest; **Record Level:** institutionCode: JES
- **Type status:** Other material. **Occurrence:** recordedBy: J. Salmela; individualCount: 2; sex: male; **Location:** country: Finland; stateProvince: Regio kuusamoensis; verbatimLocality: Kuusamo, Oulanka National Park; decimalLatitude: 66.372; decimalLongitude: 29.309; geodeticDatum: WGS84; **Identification:** identifiedBy: J. Salmela; **Event:** samplingProtocol: Malaise trap; eventDate: 2012-7-1/30; habitat: headwater stream, seminatural boreal forest
- **Type status:** Other material. **Occurrence:** recordedBy: L.Tiensuu; individualCount: 1; sex: male; **Location:** country: Finland; stateProvince: Karelia australis; municipality: Savonlinna; locality: Vehkalahti; decimalLatitude: 61.762; decimalLongitude: 28.834; geodeticDatum: WGS84; **Identification:** identifiedBy: J.Jakovlev; **Event:** samplingProtocol: sweep-netting; eventDate: 1971-6-5/6-5; **Record Level:** institutionCode: MZHF
- **Type status:** Other material. **Occurrence:** recordedBy: L.Tiensuu; individualCount: 1; sex: male; **Location:** country: Finland; stateProvince: Karelia australis; municipality: Savonlinna; locality: Vehkalahti; decimalLatitude: 61.762; decimalLongitude: 28.834; geodeticDatum: WGS84; **Identification:** identifiedBy: A.Polevoi and J.Jakovlev; **Event:** samplingProtocol: sweep-netting; eventDate: 1971-10-9/10-9; **Record Level:** institutionCode: MZHF
- **Type status:** Other material. **Occurrence:** recordedBy: J.Jakovlev; individualCount: 3; sex: male; **Location:** country: Finland; stateProvince: Tavastia australis; municipality: Lammi; locality: Evo_Kotinen_Spruce part; decimalLatitude: 61.246; decimalLongitude: 25.069; geodeticDatum: WGS84; **Identification:** identifiedBy: J.Jakovlev; **Event:** samplingProtocol: Malaise trap; eventDate: 2003-9-1/9-10; habitat: old-growth forest, Myrtillus type; **Record Level:** institutionCode: JJH
- **Type status:** Other material. **Occurrence:** recordedBy: J.Jakovlev; individualCount: 95; sex: male; **Location:** country: Finland; stateProvince: Tavastia australis; municipality: Lammi; locality: Evo_Kotinen_Spruce part; decimalLatitude: 61.246; decimalLongitude: 25.069; geodeticDatum: WGS84; **Identification:** identifiedBy: A.Polevoi; **Event:** samplingProtocol: Malaise trap; eventDate: 2003-9-3/10-3; habitat: old-growth forest, Myrtillus type
- **Type status:** Other material. **Occurrence:** recordedBy: J.Jakovlev; individualCount: 82; sex: male; **Location:** country: Finland; stateProvince: Tavastia australis; municipality: Lammi; locality: Evo_Kotinen_Spruce part; decimalLatitude: 61.246; decimalLongitude: 25.069; geodeticDatum: WGS84; **Identification:** identifiedBy: J.Jakovlev; **Event:** samplingProtocol: Malaise trap; eventDate: 2003-9-15/-10-15; habitat: old-growth forest, Myrtillus type
- **Type status:** Other material. **Occurrence:** recordedBy: J.Jakovlev; individualCount: 12; sex: male; **Location:** country: Finland; stateProvince: Tavastia australis; municipality: Lammi; locality: Evo_Kotinen_Spruce part; decimalLatitude: 61.246; decimalLongitude: 25.069; geodeticDatum: WGS84; **Identification:** identifiedBy: J.Jakovlev; **Event:** samplingProtocol: Malaise trap; eventDate: 2003-10-3/10-15; habitat: old-growth forest, Myrtillus type
- **Type status:** Other material. **Occurrence:** recordedBy: J.Jakovlev; individualCount: 35; sex: male; **Location:** country: Finland; stateProvince: Tavastia australis; municipality: Lammi; locality: Evo_Pukkivuori (metsikkö_514 K); decimalLatitude: 61.219; decimalLongitude: 25.157; geodeticDatum: WGS84; **Identification:** identifiedBy: J.Jakovlev; **Event:** samplingProtocol: Malaise trap; eventDate: 2003-9-1/9-10; habitat: old managed forest, Myrtillus type; **Record Level:** institutionCode: JJH
- **Type status:** Other material. **Occurrence:** recordedBy: J.Jakovlev; individualCount: 104; sex: male; **Location:** country: Finland; stateProvince: Tavastia australis; municipality: Lammi; locality: Evo_Pukkivuori (metsikkö_514 K); decimalLatitude: 61.219; decimalLongitude: 25.157; geodeticDatum: WGS84; **Identification:** identifiedBy: J.Jakovlev; **Event:** samplingProtocol: Malaise trap; eventDate: 2003-9-15/10-3; habitat: old managed forest, Myrtillus type
- **Type status:** Other material. **Occurrence:** recordedBy: J.Jakovlev; individualCount: 30; sex: male; **Location:** country: Finland; stateProvince: Tavastia australis; municipality: Lammi; locality: Evo_Pukkivuori (metsikkö_514 K); decimalLatitude: 61.219; decimalLongitude: 25.157; geodeticDatum: WGS84; **Identification:** identifiedBy: J.Jakovlev; **Event:** samplingProtocol: Malaise trap; eventDate: 2003-10-3/10-15; habitat: old managed forest, Myrtillus type
- **Type status:** Other material. **Occurrence:** recordedBy: J.Jakovlev; individualCount: 1; sex: male; **Location:** country: Finland; stateProvince: Regio aboënsis; municipality: Karjalohja; locality: Karkali_South; decimalLatitude: 60.238; decimalLongitude: 23.785; geodeticDatum: WGS84; **Identification:** identifiedBy: J.Jakovlev; **Event:** samplingProtocol: Malaise trap; eventDate: 2004-6-16/8-23; habitat: old-growth forest, herb-rich type
- **Type status:** Other material. **Occurrence:** recordedBy: J.Jakovlev; individualCount: 1; sex: male; **Location:** country: Finland; stateProvince: Tavastia australis; municipality: Lammi; locality: Evo_Hankajärvi; decimalLatitude: 61.204; decimalLongitude: 25.160; geodeticDatum: WGS84; **Identification:** identifiedBy: J.Jakovlev; **Event:** samplingProtocol: Malaise trap; eventDate: 2004-8-28/10-4; habitat: burnt clear-cut
- **Type status:** Other material. **Occurrence:** recordedBy: J.Jakovlev; individualCount: 25; sex: male; **Location:** country: Finland; stateProvince: Tavastia australis; municipality: Lammi; locality: Evo_Hattukivenmaa; decimalLatitude: 61.207; decimalLongitude: 25.153; geodeticDatum: WGS84; **Identification:** identifiedBy: J.Jakovlev; **Event:** samplingProtocol: Malaise trap; eventDate: 2004-8-28/10-4; habitat: burnt clear-cut
- **Type status:** Other material. **Occurrence:** recordedBy: J.Jakovlev; individualCount: 3; sex: male; **Location:** country: Finland; stateProvince: Tavastia australis; municipality: Lammi; locality: Evo_Hattukivenmaa; decimalLatitude: 61.207; decimalLongitude: 25.153; geodeticDatum: WGS84; **Identification:** identifiedBy: J.Jakovlev; **Event:** samplingProtocol: Malaise trap; eventDate: 2004-5-28/6-28; habitat: old managed forest, Myrtillus type
- **Type status:** Other material. **Occurrence:** recordedBy: J.Jakovlev; individualCount: 5; sex: male; **Location:** country: Finland; stateProvince: Tavastia australis; municipality: Lammi; locality: Evo_Kotinen_Aspen part; decimalLatitude: 61.244; decimalLongitude: 25.067; geodeticDatum: WGS84; **Identification:** identifiedBy: J.Jakovlev; **Event:** samplingProtocol: Malaise trap; eventDate: 2004-7-27/8-27; habitat: old-growth forest, herb-rich type
- **Type status:** Other material. **Occurrence:** recordedBy: J.Jakovlev; individualCount: 2; sex: male; **Location:** country: Finland; stateProvince: Tavastia australis; municipality: Lammi; locality: Evo_Kotinen_Aspen part; decimalLatitude: 61.244; decimalLongitude: 25.067; geodeticDatum: WGS84; **Identification:** identifiedBy: J.Jakovlev; **Event:** samplingProtocol: Malaise trap; eventDate: 2004-8-28/10-4; habitat: old-growth forest, herb-rich type
- **Type status:** Other material. **Occurrence:** recordedBy: J.Jakovlev; individualCount: 1; sex: male; **Location:** country: Finland; stateProvince: Tavastia australis; municipality: Lammi; locality: Evo_Kotinen_Aspen part; decimalLatitude: 61.244; decimalLongitude: 25.067; geodeticDatum: WGS84; **Identification:** identifiedBy: J.Jakovlev; **Event:** samplingProtocol: Malaise trap; eventDate: 2004-4-28/5-27; habitat: old-growth forest, herb-rich type
- **Type status:** Other material. **Occurrence:** recordedBy: J.Jakovlev; individualCount: 1; sex: male; **Location:** country: Finland; stateProvince: Tavastia australis; municipality: Lammi; locality: Evo_Lapinjärvi; decimalLatitude: 61.238; decimalLongitude: 25.087; geodeticDatum: WGS84; **Identification:** identifiedBy: J.Jakovlev; **Event:** samplingProtocol: Malaise trap; eventDate: 2004-7-27/8-27; habitat: burnt clear-cut
- **Type status:** Other material. **Occurrence:** recordedBy: J.Jakovlev; individualCount: 3; sex: male; **Location:** country: Finland; stateProvince: Tavastia australis; municipality: Lammi; locality: Evo_Lapinjärvi; decimalLatitude: 61.238; decimalLongitude: 25.087; geodeticDatum: WGS84; **Identification:** identifiedBy: J.Jakovlev; **Event:** samplingProtocol: Malaise trap; eventDate: 2004-8-28/10-4; habitat: burnt clear-cut
- **Type status:** Other material. **Occurrence:** recordedBy: J.Jakovlev; individualCount: 1; sex: male; **Location:** country: Finland; stateProvince: Tavastia australis; municipality: Lammi; locality: Evo_Leipäsuonaho; decimalLatitude: 61.203; decimalLongitude: 25.065; geodeticDatum: WGS84; **Identification:** identifiedBy: J.Jakovlev; **Event:** samplingProtocol: Malaise trap; eventDate: 2004-7-27/8-27; habitat: clear-cut
- **Type status:** Other material. **Occurrence:** recordedBy: J.Jakovlev; individualCount: 3; sex: male; **Location:** country: Finland; stateProvince: Tavastia australis; municipality: Lammi; locality: Evo_Leipäsuonaho; decimalLatitude: 61.203; decimalLongitude: 25.062; geodeticDatum: WGS84; **Identification:** identifiedBy: J.Jakovlev; **Event:** samplingProtocol: Malaise trap; eventDate: 2004-8-28/10-4; habitat: clear-cut
- **Type status:** Other material. **Occurrence:** recordedBy: J.Jakovlev; individualCount: 6; sex: male; **Location:** country: Finland; stateProvince: Tavastia australis; municipality: Lammi; locality: Evo_Leipäsuonaho; decimalLatitude: 61.203; decimalLongitude: 25.064; geodeticDatum: WGS84; **Identification:** identifiedBy: J.Jakovlev; **Event:** samplingProtocol: Malaise trap; eventDate: 2004-8-28/10-4; habitat: clear-cut
- **Type status:** Other material. **Occurrence:** recordedBy: J.Jakovlev; individualCount: 3; sex: male; **Location:** country: Finland; stateProvince: Tavastia australis; municipality: Lammi; locality: Evo_Leipäsuonaho; decimalLatitude: 61.204; decimalLongitude: 25.059; geodeticDatum: WGS84; **Identification:** identifiedBy: J.Jakovlev; **Event:** samplingProtocol: Malaise trap; eventDate: 2004-8-28/10-4; habitat: burnt clear-cut
- **Type status:** Other material. **Occurrence:** recordedBy: J.Jakovlev; individualCount: 1; sex: male; **Location:** country: Finland; stateProvince: Tavastia australis; municipality: Lammi; locality: Evo_Leipäsuonaho; decimalLatitude: 61.205; decimalLongitude: 25.064; geodeticDatum: WGS84; **Identification:** identifiedBy: J.Jakovlev; **Event:** samplingProtocol: Malaise trap; eventDate: 2004-8-28/10-4; habitat: old managed forest, Myrtillus type
- **Type status:** Other material. **Occurrence:** recordedBy: J.Jakovlev; individualCount: 2; sex: male; **Location:** country: Finland; stateProvince: Tavastia australis; municipality: Lammi; locality: Evo_Leipäsuonaho; decimalLatitude: 61.205; decimalLongitude: 25.064; geodeticDatum: WGS84; **Identification:** identifiedBy: J.Jakovlev; **Event:** samplingProtocol: Malaise trap; eventDate: 2004-5-28/6-28; habitat: old managed forest, Myrtillus type
- **Type status:** Other material. **Occurrence:** recordedBy: J.Jakovlev; individualCount: 5; sex: male; **Location:** country: Finland; stateProvince: Tavastia australis; municipality: Lammi; locality: Evo_Niemisjärvi; decimalLatitude: 61.216; decimalLongitude: 25.028; geodeticDatum: WGS84; **Identification:** identifiedBy: J.Jakovlev; **Event:** samplingProtocol: Malaise trap; eventDate: 2004-5-28/6-28; habitat: old managed forest, Myrtillus type
- **Type status:** Other material. **Occurrence:** recordedBy: J.Jakovlev; individualCount: 1; sex: male; **Location:** country: Finland; stateProvince: Tavastia australis; municipality: Lammi; locality: Evo_Palohonka; decimalLatitude: 61.222; decimalLongitude: 25.037; geodeticDatum: WGS84; **Identification:** identifiedBy: J.Jakovlev; **Event:** samplingProtocol: Malaise trap; eventDate: 2004-7-27/10-4; habitat: old-growth forest, Myrtillus type
- **Type status:** Other material. **Occurrence:** recordedBy: J.Jakovlev; individualCount: 2; sex: male; **Location:** country: Finland; stateProvince: Tavastia australis; municipality: Lammi; locality: Evo_Puukkohonka; decimalLatitude: 61.222; decimalLongitude: 25.052; geodeticDatum: WGS84; **Identification:** identifiedBy: J.Jakovlev; **Event:** samplingProtocol: Malaise trap; eventDate: 2004-7-27/8-27; habitat: old-growth forest, Myrtillus type
- **Type status:** Other material. **Occurrence:** recordedBy: J.Jakovlev; individualCount: 1; sex: male; **Location:** country: Finland; stateProvince: Tavastia australis; municipality: Lammi; locality: Evo_Puukkohonka; decimalLatitude: 61.222; decimalLongitude: 25.052; geodeticDatum: WGS84; **Identification:** identifiedBy: J.Jakovlev; **Event:** samplingProtocol: Malaise trap; eventDate: 2004-8-28/10-4; habitat: old-growth forest, Myrtillus type
- **Type status:** Other material. **Occurrence:** recordedBy: J.Jakovlev; individualCount: 23; sex: male; **Location:** country: Finland; stateProvince: Tavastia australis; municipality: Lammi; locality: Evo_Puukkohonka; decimalLatitude: 61.222; decimalLongitude: 25.052; geodeticDatum: WGS84; **Identification:** identifiedBy: J.Jakovlev; **Event:** samplingProtocol: Malaise trap; eventDate: 2004-5-28/6-28; habitat: old-growth forest, Myrtillus type
- **Type status:** Other material. **Occurrence:** recordedBy: J.Jakovlev; individualCount: 1; sex: male; **Location:** country: Finland; stateProvince: Tavastia australis; municipality: Lammi; locality: Evo_Saarijärventie_2; decimalLatitude: 61.229; decimalLongitude: 25.060; geodeticDatum: WGS84; **Identification:** identifiedBy: J.Jakovlev; **Event:** samplingProtocol: Malaise trap; eventDate: 2004-7-27/8-27; habitat: clear-cut
- **Type status:** Other material. **Occurrence:** recordedBy: J.Jakovlev; individualCount: 1; sex: male; **Location:** country: Finland; stateProvince: Tavastia australis; municipality: Lammi; locality: Evo_Saarijärventie_2; decimalLatitude: 61.229; decimalLongitude: 25.060; geodeticDatum: WGS84; **Identification:** identifiedBy: J.Jakovlev; **Event:** samplingProtocol: Malaise trap; eventDate: 2004-8-28/10-4; habitat: clear-cut
- **Type status:** Other material. **Occurrence:** recordedBy: J.Jakovlev; individualCount: 3; sex: male; **Location:** country: Finland; stateProvince: Tavastia australis; municipality: Lammi; locality: Evo_Saarijärvi; decimalLatitude: 61.230; decimalLongitude: 25.061; geodeticDatum: WGS84; **Identification:** identifiedBy: J.Jakovlev; **Event:** samplingProtocol: Malaise trap; eventDate: 2004-5-28/6-28; habitat: old managed forest, Myrtillus type
- **Type status:** Other material. **Occurrence:** recordedBy: J.Jakovlev; individualCount: 2; sex: male; **Location:** country: Finland; stateProvince: Tavastia australis; municipality: Lammi; locality: Siperiantie_1; decimalLatitude: 61.268; decimalLongitude: 25.158; geodeticDatum: WGS84; **Identification:** identifiedBy: J.Jakovlev; **Event:** samplingProtocol: Malaise trap; eventDate: 2004-8-28/10-4; habitat: old managed forest, Myrtillus type
- **Type status:** Other material. **Occurrence:** recordedBy: J.Jakovlev; individualCount: 3; sex: male; **Location:** country: Finland; stateProvince: Tavastia australis; municipality: Lammi; locality: Siperiantie_1; decimalLatitude: 61.268; decimalLongitude: 25.158; geodeticDatum: WGS84; **Identification:** identifiedBy: J.Jakovlev; **Event:** samplingProtocol: Malaise trap; eventDate: 2004-6-29/7-26; habitat: old managed forest, Myrtillus type
- **Type status:** Other material. **Occurrence:** recordedBy: J.Jakovlev; individualCount: 3; sex: male; **Location:** country: Finland; stateProvince: Tavastia australis; municipality: Lammi; locality: Siperiantie_2; decimalLatitude: 61.268; decimalLongitude: 25.160; geodeticDatum: WGS84; **Identification:** identifiedBy: J.Jakovlev; **Event:** samplingProtocol: Malaise trap; eventDate: 2004-7-27/8-27; habitat: clear-cut
- **Type status:** Other material. **Occurrence:** recordedBy: J.Jakovlev; individualCount: 14; sex: male; **Location:** country: Finland; stateProvince: Tavastia australis; municipality: Lammi; locality: Siperiantie_2; decimalLatitude: 61.268; decimalLongitude: 25.160; geodeticDatum: WGS84; **Identification:** identifiedBy: J.Jakovlev; **Event:** samplingProtocol: Malaise trap; eventDate: 2004-8-28/10-4; habitat: clear-cut
- **Type status:** Other material. **Occurrence:** recordedBy: J.Jakovlev; individualCount: 2; sex: male; **Location:** country: Finland; stateProvince: Tavastia australis; municipality: Padasjoki; locality: Vesijako Strict Nature Reserve; decimalLatitude: 61.349; decimalLongitude: 25.105; geodeticDatum: WGS84; **Identification:** identifiedBy: J.Jakovlev; **Event:** samplingProtocol: Malaise trap; eventDate: 2004-7-26/8-27; habitat: old-growth forest, Myrtillus type
- **Type status:** Other material. **Occurrence:** recordedBy: J.Jakovlev; individualCount: 1; sex: male; **Location:** country: Finland; stateProvince: Tavastia australis; municipality: Padasjoki; locality: Vesijako Strict Nature Reserve; decimalLatitude: 61.349; decimalLongitude: 25.105; geodeticDatum: WGS84; **Identification:** identifiedBy: J.Jakovlev; **Event:** samplingProtocol: Malaise trap; eventDate: 2004-8-28/10-4; habitat: old-growth forest, Myrtillus type
- **Type status:** Other material. **Occurrence:** recordedBy: J.Jakovlev; individualCount: 6; sex: male; **Location:** country: Finland; stateProvince: Tavastia australis; municipality: Padasjoki; locality: Vesijako Strict Nature Reserve; decimalLatitude: 61.349; decimalLongitude: 25.105; geodeticDatum: WGS84; **Identification:** identifiedBy: J.Jakovlev; **Event:** samplingProtocol: Malaise trap; eventDate: 2004-8-28/10-4; habitat: old-growth forest, Myrtillus type
- **Type status:** Other material. **Occurrence:** recordedBy: J.Penttinen; individualCount: 1; sex: male; **Location:** country: Finland; stateProvince: Tavastia borealis; municipality: Rautalampi; locality: Kalajavuori; decimalLatitude: 62.578; decimalLongitude: 26.698; geodeticDatum: WGS84; **Identification:** identifiedBy: J.Penttinen; **Event:** samplingProtocol: Malaise trap; eventDate: 2004-5-3/6-6; habitat: old-growth forest, Myrtillus type; **Record Level:** institutionCode: JPJ
- **Type status:** Other material. **Occurrence:** recordedBy: J.Jakovlev and J.Penttinen; individualCount: 1; sex: male; **Location:** country: Finland; stateProvince: Lapponia kittilensis; municipality: Kolari; locality: Kolari_4; decimalLatitude: 67.234; decimalLongitude: 23.693; geodeticDatum: WGS84; **Identification:** identifiedBy: J.Jakovlev; **Event:** samplingProtocol: Malaise trap; eventDate: 2006-7-15/8-15; habitat: old-growth forest, Myrtillus type
- **Type status:** Other material. **Occurrence:** recordedBy: J.Jakovlev and J.Penttinen; individualCount: 1; sex: male; **Location:** country: Finland; stateProvince: Lapponia kemensis pars occidentalis; municipality: Muonio; locality: Pallas-Yllästunturi National Park_Yllas_3; decimalLatitude: 67.592; decimalLongitude: 24.188; geodeticDatum: WGS84; **Identification:** identifiedBy: J.Jakovlev; **Event:** samplingProtocol: Malaise trap; eventDate: 2006-7-15/8-15; habitat: old-growth forest, Myrtillus type
- **Type status:** Other material. **Occurrence:** recordedBy: J.Penttinen; individualCount: 1; sex: male; **Location:** country: Finland; stateProvince: Tavastia borealis; municipality: Kannonkoski; locality: Raakkipuro_1; decimalLatitude: 62.958; decimalLongitude: 25.444; geodeticDatum: WGS84; **Identification:** identifiedBy: J.Penttinen; **Event:** samplingProtocol: Malaise trap; eventDate: 2008-5-8/6-29; habitat: old-growth forest, Myrtillus type; **Record Level:** institutionCode: JPJ
- **Type status:** Other material. **Occurrence:** recordedBy: J.Penttinen; individualCount: 1; sex: male; **Location:** country: Finland; stateProvince: Tavastia borealis; municipality: Kannonkoski; locality: Raakkipuro_2; decimalLatitude: 62.957; decimalLongitude: 25.445; geodeticDatum: WGS84; **Identification:** identifiedBy: J.Penttinen; **Event:** samplingProtocol: Malaise trap; eventDate: 2008-5-8/6-29; habitat: old-growth forest, Myrtillus type; **Record Level:** institutionCode: JPJ
- **Type status:** Other material. **Occurrence:** recordedBy: Noora Vartija; individualCount: 1; sex: male; **Location:** country: Finland; stateProvince: Tavastia australis; municipality: Muurame; locality: Kuusimäki Forest Reserve; decimalLatitude: 62.215; decimalLongitude: 25.496; geodeticDatum: WGS84; **Identification:** identifiedBy: J.Penttinen; **Event:** samplingProtocol: Reared from wood; eventDate: 2008-8-21/10-5; habitat: old-growth forest, Myrtillus type; **Record Level:** institutionCode: JPJ
- **Type status:** Other material. **Occurrence:** recordedBy: J.Jakovlev; individualCount: 1; sex: male; **Location:** country: Finland; stateProvince: Tavastia australis; municipality: Padasjoki; locality: Vesijako Strict Nature Reserve; decimalLatitude: 61.355; decimalLongitude: 25.106; geodeticDatum: WGS84; **Identification:** identifiedBy: J.Jakovlev; **Event:** samplingProtocol: Sweep netting; eventDate: 2008-8-18/8-18; habitat: old-growth forest, Myrtillus type
- **Type status:** Other material. **Occurrence:** recordedBy: J.Penttinen; individualCount: 1; sex: male; **Location:** country: Finland; stateProvince: Tavastia borealis; municipality: Rautalampi; locality: Kalajavuori; decimalLatitude: 62.573; decimalLongitude: 26.685; geodeticDatum: WGS84; **Identification:** identifiedBy: J.Penttinen; **Event:** samplingProtocol: Sweep-netting; eventDate: 2008-5-3/6-6; habitat: old-growth forest, Myrtillus type; **Record Level:** institutionCode: JPJ
- **Type status:** Other material. **Occurrence:** recordedBy: J.Penttinen; individualCount: 1; sex: male; **Location:** country: Finland; stateProvince: Tavastia borealis; municipality: Saarijärvi; locality: Pyhä-Häkki National Park; decimalLatitude: 62.842; decimalLongitude: 25.474; geodeticDatum: WGS84; **Identification:** identifiedBy: J.Penttinen; **Event:** samplingProtocol: Malaise trap; eventDate: 2008; habitat: old-growth forest, Myrtillus type; **Record Level:** institutionCode: JPJ
- **Type status:** Other material. **Occurrence:** recordedBy: J.Jakovlev; individualCount: 1; sex: male; **Location:** country: Finland; stateProvince: Nylandia; municipality: Sipoo; locality: Sipoonkorpi_1; decimalLatitude: 60.322; decimalLongitude: 25.157; geodeticDatum: WGS84; **Identification:** identifiedBy: J.Jakovlev; **Event:** samplingProtocol: Malaise trap; eventDate: 2008-8-6/8-22; habitat: old managed forest, herb-rich type

#### Distribution

Palaearctic, known from Russia (West Siberia, NW Russia), Finland, Norway, Sweden and Italy (Kurina 2008, as *Boletina jamalensis*, Søli and Rindal 2012, Polevoi 2013b). *Boletina pinusia* was recently redescribed and discussed by Polevoi 2013b. This species has been misintepreted by European authors as *Boletina jamalensis* sensu auct.; true *Boletina jamalensis* Zaitzev, also occurring in Fennoscandia, was redescribed and discussed by Polevoi 2013b.

#### Ecology

In Finland, the species has been collected in tens of sites, mostly in old-growth coniferous forests, also in ordinary clear-cuts and clear-cuts with retention trees treated with prescribed burning. Collected from a decaying aspen tree by using an eclector trap in Finland (as *Boletina jamalensis*, Halme et al. 2013)

### Boletina polaris

Lundström, 1915*

http://www.faunaeur.org/full_results.php?id=140861

#### Materials

- **Type status:** Other material. **Occurrence:** catalogNumber: MYCE-JS-2012-0061; recordedBy: J. Salmela; individualCount: 2; sex: male; **Location:** country: Finland; stateProvince: Ostrobothnia borealis pars borealis; verbatimLocality: Ylitornio, Tuorerommas; decimalLatitude: 66.476; decimalLongitude: 24.756; geodeticDatum: WGS84; **Identification:** identifiedBy: J. Salmela; **Event:** samplingProtocol: Malaise trap; eventDate: 2012-8-6/9-26; habitat: spring brook, old-growth forest; **Record Level:** institutionCode: JES
- **Type status:** Other material. **Occurrence:** catalogNumber: MYCE-JS-2012-0067; recordedBy: J. Salmela; individualCount: 3; sex: male; otherCatalogNumbers: MYCE-JS-2013-0062; **Location:** country: Finland; stateProvince: Lapponia kemensis pars orientalis; verbatimLocality: Savukoski, Joutenoja; decimalLatitude: 67.821; decimalLongitude: 29.440; geodeticDatum: WGS84; **Identification:** identifiedBy: J. Salmela; **Event:** samplingProtocol: Malaise trap; eventDate: 2012-8-16/9-18; habitat: headwater stream, seminatural boreal forest; **Record Level:** institutionCode: JES
- **Type status:** Other material. **Occurrence:** catalogNumber: DIPT-JS-2014-0054; recordedBy: J. Salmela; T. Hietajärvi; individualCount: 1; sex: male; **Location:** country: Finland; stateProvince: Regio kuusamoensis; verbatimLocality: Salla, Kuntasjoki, Värriö Strict Nature Reserve; verbatimElevation: 320 m; decimalLatitude: 67.749; decimalLongitude: 29.617; geodeticDatum: WGS84; **Identification:** identifiedBy: J. Salmela; **Event:** samplingProtocol: Malaise trap; verbatimEventDate: 2013-7-29/9-19; habitat: headwater stream, old-growth boreal forest; **Record Level:** institutionCode: JES

#### Distribution

Palaearctic. *Boletina polaris* (Fig. 14) was described from arctic Siberia (Lundström 1915) and has been later found from the Kola Peninsula, northernmost parts of Russia (Zaitzev 1994), north Sweden (Lule Lappmark, Kjaerandsen et al. 2007), Norway (Søli and Rindal 2012), Germany (Chandler 2004) and the Italian Alps (Kurina 2008). New for Finland. Finnish records are from SW Lapland (mid boreal zone) and eastern Lapland (north boreal zone).

#### Ecology

Finnish collecting localities are characterized by small lotic waters surrounded by pristine or seminatural boreal forests. Immature stages are unknown.

### Boletina pseudonitida

Zaitzev, 1994*

http://www.faunaeur.org/full_results.php?id=140863

#### Materials

- **Type status:** Other material. **Occurrence:** catalogNumber: MYCE-JS-2013-0020; recordedBy: J. Salmela; individualCount: 5; sex: male; otherCatalogNumbers: MYCE-JS-2013-0061; **Location:** country: Finland; stateProvince: Lapponia kemensis pars orientalis; verbatimLocality: Savukoski, Joutenoja; decimalLatitude: 67.821; decimalLongitude: 29.440; geodeticDatum: WGS84; **Identification:** identifiedBy: J. Salmela; **Event:** samplingProtocol: Malaise trap; habitat: headwater stream; **Record Level:** institutionCode: JES
- **Type status:** Other material. **Occurrence:** catalogNumber: MYCE-JS-2013-0147; recordedBy: J. Salmela; individualCount: 1; sex: male; **Location:** country: Finland; stateProvince: Lapponia kemensis pars orientalis; verbatimLocality: Savukoski, Törmäoja; decimalLatitude: 67.846; decimalLongitude: 29.471; geodeticDatum: WGS84; **Identification:** identifiedBy: J. Salmela; **Event:** samplingProtocol: Malaise trap; eventDate: 2012-8-16/9-18; habitat: headwater stream, old-growth boreal forest; **Record Level:** institutionCode: JES
- **Type status:** Other material. **Occurrence:** catalogNumber: MYCE-JS-2013-0262; recordedBy: J. Salmela, Jari Aaltio; individualCount: 1; sex: male; **Location:** country: Finland; stateProvince: Lapponia kemensis pars orientalis; verbatimLocality: Sodankylä, Pomokaira, Syväkuru; decimalLatitude: 67.871; decimalLongitude: 26.210; geodeticDatum: WGS84; **Identification:** identifiedBy: J. Salmela; **Event:** samplingProtocol: sugar bait, hand net; eventDate: 2012-8-21; habitat: old-growth spruce forest; **Record Level:** institutionCode: JES
- **Type status:** Other material. **Occurrence:** catalogNumber: MYCE-NV-2013-0086; recordedBy: J. Salmela; individualCount: 1; sex: male; **Location:** country: Finland; stateProvince: Lapponia kemensis pars occidentalis; verbatimLocality: Kittilä, Kielisenpalo; decimalLatitude: 68.020; decimalLongitude: 25.063; geodeticDatum: WGS84; **Identification:** identifiedBy: N. Vartija; **Event:** samplingProtocol: Malaise trap; eventDate: 2007-7-28/8-31; habitat: rich spring fen; **Record Level:** institutionCode: JES
- **Type status:** Other material. **Occurrence:** catalogNumber: MYCE-NV-2013-0157; recordedBy: J. Salmela; individualCount: 1; sex: male; **Location:** country: Finland; stateProvince: Lapponia kemensis pars orientalis; verbatimLocality: Sodankylä, Ylä-Postojoki; decimalLatitude: 67.851; decimalLongitude: 26.481; geodeticDatum: WGS84; **Identification:** identifiedBy: N. Vartija; **Event:** samplingProtocol: Malaise trap; eventDate: 2009-6-29/8-3; habitat: headwater stream; **Record Level:** institutionCode: JES
- **Type status:** Other material. **Occurrence:** catalogNumber: MYCE-NV-2013-0243; recordedBy: J. Salmela; individualCount: 28; sex: male; **Location:** country: Finland; stateProvince: Lapponia kemensis pars occidentalis; verbatimLocality: Kittilä, Pomokaira, Tarpomapää; decimalLatitude: 67.820; decimalLongitude: 25.919; geodeticDatum: WGS84; **Identification:** identifiedBy: N. Vartija; **Event:** samplingProtocol: Malaise trap; eventDate: 2009-6-1/29; habitat: spring brook, spruce mire; **Record Level:** institutionCode: JES
- **Type status:** Other material. **Occurrence:** recordedBy: J. Jakovlev; J. Penttinen; individualCount: 2; sex: male; **Location:** country: Finland; stateProvince: Lapponia enontekiensis; verbatimLocality: Enontekiö, Kilpisjärvi, Saana; decimalLatitude: 69.0456; decimalLongitude: 20.8186; geodeticDatum: WGS84; **Identification:** identifiedBy: J. Jakovlev; **Event:** samplingProtocol: Malaise trap; eventDate: 2006-7-15/8-1

#### Distribution

**Figure 15.**
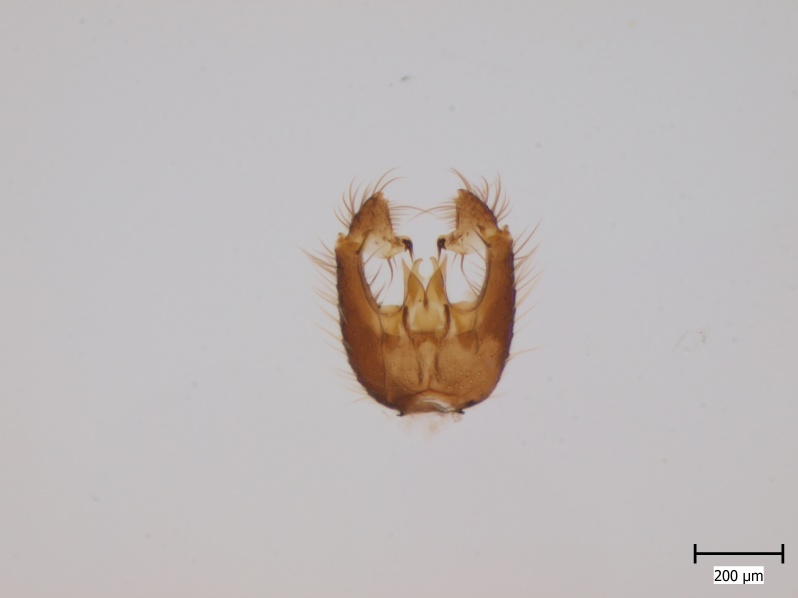
*Boletina pseudonitida* Zaitzev, male specimen collected from Finland, Savukoski (northeastern Lapland). Male hypopygium, ventral view.

Palaearctic. *Boletina pseudonitida* (Fig. 15) was described from the Altai Mountains (Zaitzev 1994) and has been since only recorded from north Sweden (Kjaerandsen et al. 2007) and northernmost Norway (Søli and Rindal 2012). New for Finland.

#### Ecology

Finnish collecting sites are mainly coniferous forests around lotic waters, also caught from a subarctic mountain birch forest and from a rich fen. Immature stages are unknown.

### Boletina takagii

Sasakawa & Kimura, 1974

http://www.faunaeur.org/full_results.php?id=140872

#### Materials

- **Type status:** Other material. **Occurrence:** recordedBy: A. Polevoi; individualCount: 1; sex: male; **Location:** country: Russia; stateProvince: Republic Karelia; verbatimLocality: Gizhino; decimalLatitude: 60.993; decimalLongitude: 33.79; geodeticDatum: WGS84; **Identification:** identifiedBy: A. Polevoi; **Event:** samplingProtocol: Malaise trap; eventDate: 2008-7-4/6; **Record Level:** institutionCode: FRIP
- **Type status:** Other material. **Occurrence:** recordedBy: A. Polevoi; individualCount: 1; sex: male; **Location:** country: Russia; stateProvince: Republic Karelia; verbatimLocality: Shunga, Turastamozero; decimalLatitude: 62.559; decimalLongitude: 34.709; geodeticDatum: WGS84; **Identification:** identifiedBy: A. Polevoi; **Event:** samplingProtocol: Malaise trap; eventDate: 2012-7-21/8-24; **Record Level:** institutionCode: FRIP
- **Type status:** Other material. **Occurrence:** recordedBy: A. Polevoi; individualCount: 1; sex: male; **Location:** country: Russia; stateProvince: Leningrad province; verbatimLocality: Gimreka; decimalLatitude: 61.150; decimalLongitude: 35.641; geodeticDatum: WGS84; **Identification:** identifiedBy: A. Polevoi; **Event:** samplingProtocol: Malaise trap; eventDate: 2008-6-26/7-25; **Record Level:** institutionCode: FRIP
- **Type status:** Other material. **Occurrence:** catalogNumber: MYCE-NV-2013-0162; recordedBy: J. Salmela; individualCount: 1; sex: male; **Location:** country: Finland; stateProvince: Lapponia kemensis pars orientalis; verbatimLocality: Sodankylä, Ylä-Postojoki; decimalLatitude: 67.851; decimalLongitude: 26.481; geodeticDatum: WGS84; **Identification:** identifiedBy: N. Vartija; J. Salmela; **Event:** samplingProtocol: Malaise trap; eventDate: 2009-6-29/8-3; habitat: headwater stream; **Record Level:** institutionCode: JES
- **Type status:** Other material. **Occurrence:** individualCount: 4; sex: male; **Location:** country: Finland; stateProvince: Lapponia inarensis; verbatimLocality: Utsjoki; decimalLatitude: 69.88; decimalLongitude: 27.04; geodeticDatum: WGS84; georeferenceRemarks: no detailed information on the sampling locality; **Identification:** identifiedBy: J. Jakovlev; **Event:** eventDate: undated; **Record Level:** institutionCode: MZHF

#### Distribution

Palaearctic. The species was described from Japan (Sasakawa and Kimura 1974) and has been later found on the Kuril Islands (Zaitzev 1994). In Europe it has recently been reported only from the Fennoscandian region: Russian Karelia (Polevoi 2000), Murmansk Province (Polevoi 2010), Finland (Jakovlev et al. 2006), Sweden (Kjaerandsen et al. 2007) and Norway (Gammelmo and Søli 2006, Søli and Rindal 2012). All Finnish findings are from the north boreal (Sodankylä) and subarctic (Utsjoki) areas, however in Eastern Fennoscandia there are also records from the south boreal zone.

#### Ecology

The trapping site in Sodankylä is a headwater stream surrounded by coniferous forest. In NW Russia collected mainly in secondary deciduous and mixed forests but also in mountain scrub. Immature stages are unknown.

### Boletina tiroliensis

Plassmann, 1980**

http://www.faunaeur.org/full_results.php?id=140873

#### Materials

- **Type status:** Other material. **Occurrence:** recordedBy: M. Tietäväinen et al.; individualCount: 1; sex: male; **Location:** country: Russia; stateProvince: Republic Karelia; verbatimLocality: 5 km N of Tolvojarvi; decimalLatitude: 62.318; decimalLongitude: 31.436; geodeticDatum: WGS84; **Identification:** identifiedBy: A. Polevoi; **Event:** samplingProtocol: Malaise trap; eventDate: 1999-9-13/29; **Record Level:** institutionCode: FRIP
- **Type status:** Other material. **Occurrence:** catalogNumber: MYCE-JS-2013-0053; recordedBy: J. Salmela; individualCount: 2; sex: male; otherCatalogNumbers: MYCE-JS-2013-0111; **Location:** country: Finland; stateProvince: Lapponia kemensis pars orientalis; verbatimLocality: Savukoski, Joutenoja; decimalLatitude: 67.821; decimalLongitude: 29.440; geodeticDatum: WGS84; **Identification:** identifiedBy: J. Salmela; **Event:** samplingProtocol: Malaise trap; eventDate: 2012-8-16/9-18; habitat: headwater stream, seminatural boreal forest; **Record Level:** institutionCode: JES
- **Type status:** Other material. **Occurrence:** recordedBy: J.Jakovlev and J.Penttinen; individualCount: 4; sex: male; **Location:** country: Finland; stateProvince: Lapponia kemensis pars occidentalis; municipality: Kittilä; locality: Pallas-Yllästunturi National Park; decimalLatitude: 69.018; decimalLongitude: 24.153; geodeticDatum: WGS84; **Identification:** identifiedBy: J.Jakovlev; **Event:** samplingProtocol: Malaise trap; eventDate: 2006-7-15/8-14; habitat: old-growth forest, Myrtillus type
- **Type status:** Other material. **Occurrence:** recordedBy: J.Jakovlev and J.Penttinen; individualCount: 2; sex: male; **Location:** country: Finland; stateProvince: Lapponia kemensis pars occidentalis; municipality: Kittilä; locality: Pallas-Yllästunturi National Park; decimalLatitude: 68.038; decimalLongitude: 24.136; geodeticDatum: WGS84; **Identification:** identifiedBy: J.Jakovlev; **Event:** samplingProtocol: Malaise trap; eventDate: 2006-7-15/8-14; habitat: old-growth forest, Myrtillus type
- **Type status:** Other material. **Occurrence:** recordedBy: J.Jakovlev and J.Penttinen; individualCount: 2; sex: male; **Location:** country: Finland; stateProvince: Lapponia kemensis pars occidentalis; municipality: Kittilä; locality: Pallas-Yllästunturi National Park; decimalLatitude: 68.038; decimalLongitude: 24.136; geodeticDatum: WGS84; **Identification:** identifiedBy: J.Jakovlev; **Event:** samplingProtocol: Malaise trap; eventDate: 2006-8-15/9-15; habitat: old-growth forest, Myrtillus type

#### Distribution

Palaearctic. Known from Austria (Plassmann 1980), Sweden (Kjaerandsen et al. 2007), Norway (Gammelmo and Søli 2006, Søli and Rindal 2012), Russia (Dikson island, Yamal Peninsula, Kola Peninsula, Yakutia Zaitzev 1994) and Finland (Polevoi et al. 2006). Finnish records are from the mid boreal (Kainuu Province, Ostrobothnia kajanense Polevoi et al. 2006) and north boreal zones (material presented here). New to the Republic of Karelia.

#### Ecology

Finnish collecting sites are old-growth boreal forests and a headwater stream surrounded by seminatural boreal forest. The Karelian specimen was collected in *Vaccinium myrtillus* type sprucedominated forest. Immature stages are unknown.

#### Conservation

Red-listed in Norway (NT, Anonymous 2010, Gammelmo et al. 2010)

### Boletina verticillata

Stackelberg, 1943

http://www.faunaeur.org/full_results.php?id=140877

#### Materials

- **Type status:** Other material. **Occurrence:** catalogNumber: MYCE-JS-2012-0009; recordedBy: J. Salmela; individualCount: 1; sex: male; **Location:** country: Finland; stateProvince: Lapponia kemensis pars orientalis; verbatimLocality: Savukoski, Törmäoja; decimalLatitude: 67.846; decimalLongitude: 29.471; geodeticDatum: WGS84; **Identification:** identifiedBy: J. Salmela; **Event:** samplingProtocol: Malaise trap; eventDate: 2012-7-10/8-16; habitat: headwater stream, old-growth boreal forest; **Record Level:** institutionCode: JES
- **Type status:** Other material. **Occurrence:** catalogNumber: MYCE-JS-2013-0017; recordedBy: J. Salmela; individualCount: 7; sex: male; otherCatalogNumbers: MYCE-JS-2013-0060; **Location:** country: Finland; stateProvince: Lapponia kemensis pars orientalis; verbatimLocality: Savukoski, Joutenoja; decimalLatitude: 67.821; decimalLongitude: 29.440; geodeticDatum: WGS84; **Identification:** identifiedBy: J. Salmela; **Event:** samplingProtocol: Malaise trap; eventDate: 2012-8-16/9-18; habitat: headwater stream, seminatural boreal forest; **Record Level:** institutionCode: JES
- **Type status:** Other material. **Occurrence:** catalogNumber: MYCE-NV-2013-0116; recordedBy: J. Salmela; individualCount: 1; sex: male; otherCatalogNumbers: MYCE-JS-2013-0060; **Location:** country: Finland; stateProvince: Lapponia kemensis pars occidentalis; verbatimLocality: Kittilä, Vuotsonperänjänkä; decimalLatitude: 67.616; decimalLongitude: 25.449; geodeticDatum: WGS84; **Identification:** identifiedBy: N. Vartija; **Event:** samplingProtocol: Malaise trap; eventDate: 2012-8-16/9-18; habitat: rich fen; **Record Level:** collectionID: 2007-8-1/9-3; institutionCode: JES
- **Type status:** Other material. **Occurrence:** recordedBy: J. Salmela; individualCount: 4; sex: male; **Location:** country: Finland; stateProvince: Lapponia inarensis; verbatimLocality: Utsjoki, Kaldoaivi, Galddasjohka; decimalLatitude: 69.860; decimalLongitude: 27.809; geodeticDatum: WGS84; **Identification:** identifiedBy: J. Jakovlev; **Event:** samplingProtocol: Malaise trap; eventDate: 2007-7-19/8-27; habitat: headwater stream, fell area
- **Type status:** Other material. **Occurrence:** recordedBy: R.Frey; individualCount: 1; sex: male; **Location:** country: Finland; stateProvince: Lapponia kemensis pars occidentalis; municipality: Kittilä; locality: Kittilä; decimalLatitude: 67.654; decimalLongitude: 24.898; geodeticDatum: WGS84; **Identification:** identifiedBy: J. Jakovlev; **Event:** eventDate: 1900; habitat: subarctic; **Record Level:** institutionCode: MZHF
- **Type status:** Other material. **Occurrence:** recordedBy: J.Salmela; individualCount: 1; sex: male; **Location:** country: Finland; stateProvince: Lapponia inarensis; municipality: Utsjoki; locality: Galddasjohka; decimalLatitude: 69.861; decimalLongitude: 27.790; geodeticDatum: WGS84; **Identification:** identifiedBy: J.Jakovlev; **Event:** samplingProtocol: Malaise trap; eventDate: 2007-7-19/8-27; habitat: subarctic; **Record Level:** institutionCode: JJH
- **Type status:** Other material. **Occurrence:** recordedBy: J.Salmela; individualCount: 1; sex: male; **Location:** country: Finland; stateProvince: Lapponia inarensis; municipality: Utsjoki; locality: Galddasjohka; decimalLatitude: 69.860; decimalLongitude: 27.770; geodeticDatum: WGS84; **Identification:** identifiedBy: J.Jakovlev; **Event:** samplingProtocol: Malaise trap; eventDate: 2007-7-19/8-27; habitat: subarctic; **Record Level:** institutionCode: JJH

#### Distribution

**Figure 16.**
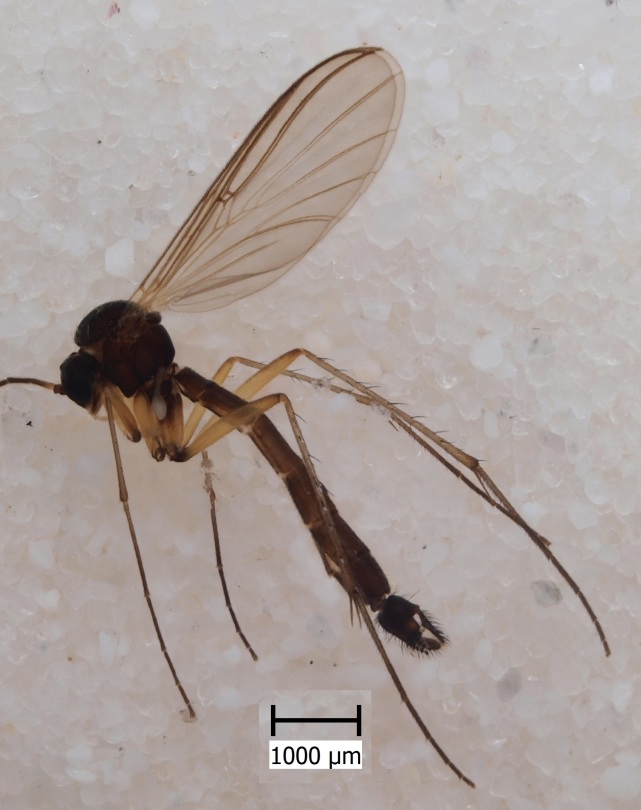
*Boletina verticillata* Stackelberg, male specimen collected from Finland, Savukoski (eastern Lapland). Habitus, lateral view.

Palaearctic. *Boletina verticillata* (Fig. 16) was described from NW Siberia (Stackelberg 1943) and has been later found from Russian Far East, Mongolia (Zaitzev 1994), Norway (Gammelmo and Søli 2006, Gammelmo et al. 2010) and Sweden (Kjaerandsen et al. 2007). Finnish records are from the north boreal zone (Kittilä, Savukoski) and from the subarctic zone (Utsjoki).

#### Ecology

Finnish sampling localities are riparian forests and a rich fen. Immature stages are unknown.

#### Conservation

Red-listed in Norway (VU, Anonymous 2010, Gammelmo et al. 2010).

### Coelosia gracilis

Johannsen, 1912***

http://www.catalogueoflife.org/col/details/species/id/8668681

#### Materials

- **Type status:** Other material. **Occurrence:** catalogNumber: DIPT-JS-2014-0057; recordedBy: J. Salmela; T. Hietajärvi; individualCount: 1; sex: male; **Location:** country: Finland; stateProvince: Regio kuusamoensis; verbatimLocality: Salla, Kuntasjoki, Värriö Strict Nature Reserve; verbatimElevation: 320 m; decimalLatitude: 67.749; decimalLongitude: 29.617; geodeticDatum: WGS84; **Identification:** identifiedBy: J. Salmela; **Event:** samplingProtocol: Malaise trap; verbatimEventDate: 2013-7-29/9-19; habitat: headwater stream, old-growth boreal forest; **Record Level:** institutionCode: JES
- **Type status:** Other material. **Occurrence:** catalogNumber: MYCE-JS-2013-0007; recordedBy: J. Salmela; individualCount: 5; sex: male; otherCatalogNumbers: MYCE-JS-2013-0059; **Location:** country: Finland; stateProvince: Lapponia kemensis pars orientalis; verbatimLocality: Savukoski, Joutenoja; decimalLatitude: 67.821; decimalLongitude: 29.440; geodeticDatum: WGS84; **Identification:** identifiedBy: J. Salmela; A. Polevoi; **Event:** samplingProtocol: Malaise trap; eventDate: 2012-8-16/9-18; habitat: headwater stream, seminatural boreal forest; **Record Level:** institutionCode: JES
- **Type status:** Other material. **Occurrence:** recordedBy: J. Salmela; individualCount: 4; sex: male; otherCatalogNumbers: MYCE-JS-2013-0059; **Location:** country: Finland; stateProvince: Lapponia kemensis pars orientalis; verbatimLocality: Savukoski, Törmäoja; decimalLatitude: 67.846; decimalLongitude: 29.471; geodeticDatum: WGS84; **Identification:** identifiedBy: J. Salmela; A. Polevoi; **Event:** samplingProtocol: Malaise trap; eventDate: 2012-8-16/9-18; habitat: headwater stream, old-growth boreal forest; **Record Level:** institutionCode: JES
- **Type status:** Other material. **Occurrence:** recordedBy: J. Jakovlev; J. Penttinen; individualCount: 1; sex: male; **Location:** country: Finland; stateProvince: Lapponia enontekiensis; verbatimLocality: Enontekiö, Kilpisjärvi, Saana; decimalLatitude: 69.044; decimalLongitude: 20.816; geodeticDatum: WGS84; **Identification:** identifiedBy: J. Jakovlev; **Event:** samplingProtocol: Malaise trap; eventDate: 2006-6-19/7-14; habitat: mountain birch forest, herb rich vegetation; **Record Level:** institutionCode: JJH
- **Type status:** Other material. **Occurrence:** catalogNumber: MYCE-NV-2013-0189; recordedBy: J. Salmela; individualCount: 8; sex: male; **Location:** country: Finland; stateProvince: Lapponia kemensis pars occidentalis; verbatimLocality: Kittilä, Vielmakoskenpalo NW; decimalLatitude: 68.009; decimalLongitude: 25.044; geodeticDatum: WGS84; **Identification:** identifiedBy: N. Vartija; J. Salmela; **Event:** samplingProtocol: Malaise trap; eventDate: 2007-8-2/31; habitat: rich spruce mire; **Record Level:** institutionCode: JES

#### Distribution

**Figure 17.**
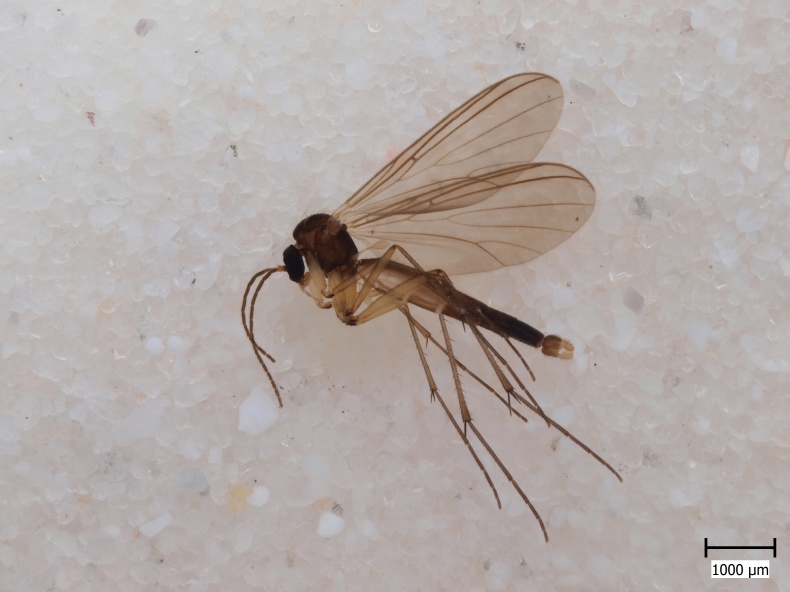
*Coelosia gracilis* (Johannsen), male specimen collected from Finland, Savukoski (northeastern Lapland). Habitus, lateral view.

Holarctic. *Coelosia gracilis* (Fig. 17) is here reported for the first time from the Palaearctic region. The species was described from the USA, California and Colorado (Johannsen 1911) and according to Søli (Søli 1997) the species has a wide range in the western part of the Nearctic region.

#### Ecology

Finnish sampling sites are headwater streams surrounded by boreal forests, a mountain birch forest and a rich spruce mire. Immature stages are unknown.

#### Taxon discussion

*Coelosia gracilis* is very close to the European species *Coelosia truncata* Lundström, 1909, and perhaps overlooked in the Palaearctic region.

### Coelosia flava

(Stæger, 1840)**

http://www.faunaeur.org/full_results.php?id=140782

#### Materials

- **Type status:** Other material. **Occurrence:** recordedBy: A. Polevoi; individualCount: 1; sex: male; **Location:** country: Russia; stateProvince: Republic Karelia; verbatimLocality: Obzha, Mayachino; decimalLatitude: 60.777; decimalLongitude: 32.818; geodeticDatum: WGS84; **Identification:** identifiedBy: A. Polevoi; **Event:** samplingProtocol: Malaise trap; eventDate: 2012-6-22/8; **Record Level:** institutionCode: FRIP

#### Distribution

European. Widely distributed (Chandler 2004). New to the Republic of Karelia.

#### Ecology

The Karelian specimen was collected in a black alder fen. Immature stages are unknown. *Coelosia* larvae are generally associated with fungal fruiting bodies (Jakovlev 1994), but some species have been collected with eclector traps over dead wood, or on soil (Jakovlev et al. 1994, Økland 1999).

### Coelosia limpida

Plassmann, 1986*

http://www.faunaeur.org/full_results.php?id=140786

#### Materials

- **Type status:** Other material. **Occurrence:** catalogNumber: MYCE-JS-2012-0073; recordedBy: J. Salmela; individualCount: 26; sex: male; otherCatalogNumbers: MYCE-JS-2013-0005; **Location:** country: Finland; stateProvince: Lapponia kemensis pars orientalis; verbatimLocality: Savukoski, Joutenoja; decimalLatitude: 67.821; decimalLongitude: 29.440; geodeticDatum: WGS84; **Identification:** identifiedBy: J. Salmela; **Event:** samplingProtocol: Malaise trap; eventDate: 2012-8-16/9-18; habitat: headwater stream, seminatural boreal forest; **Record Level:** institutionCode: JES
- **Type status:** Other material. **Occurrence:** recordedBy: J. Salmela; individualCount: 26; sex: male; **Location:** country: Finland; stateProvince: Lapponia kemensis pars orientalis; verbatimLocality: Savukoski, Törmäoja; decimalLatitude: 67.846; decimalLongitude: 29.471; geodeticDatum: WGS84; **Identification:** identifiedBy: J. Salmela; **Event:** samplingProtocol: Malaise trap; eventDate: 2012-8-16/9-18; habitat: headwater stream, old-growth boreal forest; **Record Level:** institutionCode: JES
- **Type status:** Other material. **Occurrence:** catalogNumber: MYCE-NV-2013-0048; recordedBy: J. Salmela; individualCount: 26; sex: male; **Location:** country: Finland; stateProvince: Lapponia kemensis pars occidentalis; verbatimLocality: Kittilä, Repsuvuoma; decimalLatitude: 67.604; decimalLongitude: 24.967; geodeticDatum: WGS84; **Identification:** identifiedBy: N. Vartija; **Event:** samplingProtocol: Malaise trap; eventDate: 2007-8-1/9-3; habitat: rich fen; **Record Level:** institutionCode: JES
- **Type status:** Other material. **Occurrence:** recordedBy: J. Salmela; individualCount: 1; sex: male; **Location:** country: Finland; stateProvince: Lapponia kemensis pars occidentalis; verbatimLocality: Kittilä, Repsuvuoma; decimalLatitude: 67.582; decimalLongitude: 25.543; geodeticDatum: WGS84; **Identification:** identifiedBy: N. Vartija; **Event:** samplingProtocol: Malaise trap; eventDate: 2007-8-1/9-3; habitat: rich fen
- **Type status:** Other material. **Occurrence:** catalogNumber: MYCE-NV-2013-0069; recordedBy: J. Salmela; individualCount: 10; sex: male; **Location:** country: Finland; stateProvince: Lapponia kemensis pars occidentalis; verbatimLocality: Kittilä, Taljavaaranvuoma; decimalLatitude: 67.578; decimalLongitude: 25.358; geodeticDatum: WGS84; **Identification:** identifiedBy: N. Vartija; **Event:** samplingProtocol: Malaise trap; eventDate: 2007-8-1/9-2; habitat: rich fen; **Record Level:** institutionCode: JES
- **Type status:** Other material. **Occurrence:** catalogNumber: MYCE-NV-2013-0081; recordedBy: J. Salmela; individualCount: 2; sex: male; **Location:** country: Finland; stateProvince: Lapponia kemensis pars occidentalis; verbatimLocality: Kittilä, Kielisenpalo; decimalLatitude: 68.020; decimalLongitude: 25.063; geodeticDatum: WGS84; **Identification:** identifiedBy: N. Vartija; **Event:** samplingProtocol: Malaise trap; eventDate: 2007-8-1/31; habitat: rich spring fen; **Record Level:** institutionCode: JES
- **Type status:** Other material. **Occurrence:** recordedBy: J. Salmela; individualCount: 5; sex: male; **Location:** country: Finland; stateProvince: Lapponia kemensis pars occidentalis; verbatimLocality: Kittilä, Vuotsonperänjänkä; decimalLatitude: 67.616; decimalLongitude: 25.449; geodeticDatum: WGS84; **Identification:** identifiedBy: N. Vartija; **Event:** samplingProtocol: Malaise trap; eventDate: 2007-8-1/9-3; habitat: rich fen
- **Type status:** Other material. **Occurrence:** recordedBy: J. Salmela; individualCount: 52; sex: male; **Location:** country: Finland; stateProvince: Lapponia kemensis pars occidentalis; verbatimLocality: Kittilä, Vielmakoskenpalo; decimalLatitude: 68.008; decimalLongitude: 25.046; geodeticDatum: WGS84; **Identification:** identifiedBy: N. Vartija; **Event:** samplingProtocol: Malaise trap; eventDate: 2007-8-2/31; habitat: rich fen
- **Type status:** Other material. **Occurrence:** catalogNumber: MYCE-JS-2013-0274; recordedBy: J. Salmela; individualCount: 368; sex: male; otherCatalogNumbers: MYCE-JS-2013-0298; **Location:** country: Finland; stateProvince: Nylandia; verbatimLocality: Espoo, Matalajärvi; decimalLatitude: 60.247; decimalLongitude: 24.687; geodeticDatum: WGS84; **Identification:** identifiedBy: J. Salmela; **Event:** samplingProtocol: Malaise trap; samplingEffort: 8 traps; eventDate: 2012-7-21/10-20; habitat: swampy lake shore; **Record Level:** institutionCode: JES
- **Type status:** Other material. **Occurrence:** recordedBy: M.Jaschhof and C.Jaschhof; individualCount: 1; sex: male; **Location:** country: Finland; stateProvince: Karelia ladogensis; municipality: Parikkala; locality: Lake Siikalahti W Kaukola; decimalLatitude: 61.561; decimalLongitude: 29.593; geodeticDatum: WGS84; **Identification:** identifiedBy: J.Jakovlev; **Event:** samplingProtocol: Malaise trap; eventDate: 2004-6-24/8-19; habitat: old managed forest, swampy; **Record Level:** institutionCode: JJH
- **Type status:** Other material. **Occurrence:** recordedBy: M.Jaschhof and C.Jaschhof; individualCount: 2; sex: male; **Location:** country: Finland; stateProvince: Karelia ladogensis; municipality: Parikkala; locality: Lake Siikalahti W Kaukola, <100m; decimalLatitude: 61.561; decimalLongitude: 29.593; geodeticDatum: WGS84; **Identification:** identifiedBy: J.Jakovlev; **Event:** samplingProtocol: Sweep netting; eventDate: 2004-8-19/8-19; habitat: old managed forest, swampy; **Record Level:** institutionCode: JJH
- **Type status:** Other material. **Occurrence:** recordedBy: J.Salmela; individualCount: 1; sex: male; **Location:** country: Finland; stateProvince: Lapponia inarensis; municipality: Utsjoki; locality: Galddasjohka; decimalLatitude: 69.861; decimalLongitude: 27.790; geodeticDatum: WGS84; **Identification:** identifiedBy: J.Jakovlev; **Event:** samplingProtocol: Malaise trap; eventDate: 2007-7-19/8-27; habitat: stream valley with a narrow belt of mountain birch forest; **Record Level:** institutionCode: JJH

#### Distribution

Fennoscandian species, only known from Sweden (Plassmann 1987, Kjaerandsen et al. 2007), Norway (Gammelmo et al. 2010, Søli and Rindal 2012), Russian Karelia (Humala and Polevoi 2009) and Murmansk Province (Polevoi 2010). New for Finland. Wide range in Finland, known from the hemiboreal, north boreal and subarctic zones.

#### Ecology

All Finnish collecting sites are wetlands, in a wide sense. The species is apparently quite common on rich fens in central Lapland (Kittilä) and it was very numerous on the shores of Lake Matalajärvi, Espoo (hemiboreal zone). *Coelosia limpida* may prefer calcareous habitats. Immature stages are unknown.

#### Conservation

Red-listed in Norway (DD, Anonymous 2010, Gammelmo et al. 2010).

### Ectrepesthoneura nigra

Zaitzev, 1984* **

http://www.faunaeur.org/full_results.php?id=140766

#### Materials

- **Type status:** Other material. **Occurrence:** recordedBy: M. Tietäväinen et al.; individualCount: 1; sex: male; **Location:** country: Russia; stateProvince: Republic Karelia; verbatimLocality: 5 km N of Tolvojarvi; decimalLatitude: 62.318; decimalLongitude: 31.436; geodeticDatum: WGS84; **Identification:** identifiedBy: A. Polevoi; **Event:** samplingProtocol: Malaise trap; eventDate: 1999-6-11/22; **Record Level:** institutionCode: FRIP
- **Type status:** Other material. **Occurrence:** catalogNumber: DIPT-JS-2014-0006; recordedBy: J. Salmela; T. Hietajärvi; individualCount: 1; sex: male; **Location:** country: Finland; stateProvince: Regio kuusamoensis; verbatimLocality: Salla, Kuntasjoki, Värriö Strict Nature Reserve; verbatimElevation: 320 m; decimalLatitude: 67.749; decimalLongitude: 29.617; geodeticDatum: WGS84; **Identification:** identifiedBy: J. Salmela; **Event:** samplingProtocol: Malaise trap; eventDate: 2013; verbatimEventDate: 2013-6-4/29; habitat: headwater stream, old-growth boreal forest; **Record Level:** institutionCode: JES

#### Distribution

European. The species was known from the type locality in Central Russia (Zaitzev 1994) and Norway (Gammelmo and Søli 2006). New to the Republic of Karelia and Finland.

#### Ecology

The Karelian specimen was collected in *Vaccinium myrtillus* type spruce dominated forest. The Finnish sampling site is a north boreal headwater stream with swampy margins, surrounded by old-growth spruce forest. Larvae feed on fungal mycelium in decaying wood (Zaitzev 1994).

#### Conservation

Red-listed in Norway (NT, Anonymous 2010, Gammelmo et al. 2010).

### Gnoriste bilineata

Zetterstedt, 1852

http://www.faunaeur.org/full_results.php?id=140749

#### Materials

- **Type status:** Other material. **Occurrence:** catalogNumber: MYCE-JS-2012-0001; recordedBy: J. Salmela; individualCount: 1; sex: male; **Location:** country: Finland; stateProvince: Lapponia kemensis pars orientalis; verbatimLocality: Savukoski, Törmäoja; decimalLatitude: 67.846; decimalLongitude: 29.471; geodeticDatum: WGS84; **Identification:** identifiedBy: J. Salmela; **Event:** samplingProtocol: Malaise trap; eventDate: 2012-7-10/8-16; habitat: headwater stream, old-growth boreal forest; **Record Level:** institutionCode: JES
- **Type status:** Other material. **Occurrence:** catalogNumber: MYCE-JS-2012-0037; recordedBy: J. Salmela; individualCount: 6; sex: male; **Location:** country: Finland; stateProvince: Lapponia kemensis pars orientalis; verbatimLocality: Savukoski, Joutenoja; decimalLatitude: 67.821; decimalLongitude: 29.440; geodeticDatum: WGS84; **Identification:** identifiedBy: J. Salmela; **Event:** samplingProtocol: Malaise trap; eventDate: 2012-7-10/8-16; habitat: headwater stream, seminatural boreal forest; **Record Level:** institutionCode: JES
- **Type status:** Other material. **Occurrence:** catalogNumber: MYCE-NV-2013-0155; recordedBy: J. Salmela; individualCount: 6; sex: male; **Location:** country: Finland; stateProvince: Lapponia kemensis pars orientalis; verbatimLocality: Sodankylä, Ylä-Postojoki; decimalLatitude: 67.851; decimalLongitude: 26.481; geodeticDatum: WGS84; **Identification:** identifiedBy: N. Vartija; **Event:** samplingProtocol: Malaise trap; eventDate: 2009-6-29/8-3; habitat: headwater stream, seminatural boreal forest; **Record Level:** institutionCode: JES
- **Type status:** Other material. **Occurrence:** catalogNumber: MYCE-NV-2013-0181; recordedBy: J. Salmela; individualCount: 1; sex: male; **Location:** country: Finland; stateProvince: Lapponia kemensis pars occidentalis; verbatimLocality: Kittilä, Vielmakoskenpalo NW; decimalLatitude: 68.009; decimalLongitude: 25.044; geodeticDatum: WGS84; **Identification:** identifiedBy: N. Vartija; **Event:** samplingProtocol: Malaise trap; eventDate: 2007-6-26/8-1; habitat: rich spruce mire; **Record Level:** institutionCode: JES
- **Type status:** Other material. **Occurrence:** recordedBy: W.Hellen; individualCount: 1; sex: male; **Location:** country: Finland; stateProvince: Tavastia borealis; municipality: Tuovilanlaks; locality: Vammelsjoki; decimalLatitude: 63.228; decimalLongitude: 27.115; geodeticDatum: WGS84; **Identification:** identifiedBy: C.Lundström; **Event:** eventDate: 1905; **Record Level:** institutionCode: MZHF
- **Type status:** Other material. **Occurrence:** recordedBy: R.Frey; individualCount: 1; sex: male; **Location:** country: Finland; stateProvince: Regio aboënsis; municipality: Vihti; locality: Vihtijärvi; decimalLatitude: 60.523; decimalLongitude: 24.556; geodeticDatum: WGS84; **Identification:** identifiedBy: C.Lundström; **Event:** eventDate: 1905; habitat: herb-rich forest; **Record Level:** institutionCode: MZHF
- **Type status:** Other material. **Occurrence:** recordedBy: R.Frey; individualCount: 1; sex: male; **Location:** country: Finland; stateProvince: Regio aboensis; municipality: Vihti; locality: Vihtijärvi; decimalLatitude: 60.523; decimalLongitude: 24.556; geodeticDatum: WGS84; **Identification:** identifiedBy: C.Lundström; **Event:** eventDate: 1905; **Record Level:** institutionCode: MZHF
- **Type status:** Other material. **Occurrence:** recordedBy: R.Frey; individualCount: 1; sex: male; **Location:** country: Finland; stateProvince: Alandia; locality: Jomala; decimalLatitude: 60.151; decimalLongitude: 19.948; geodeticDatum: WGS84; **Identification:** identifiedBy: C.Lundström; **Event:** eventDate: 1905; habitat: herb-rich forest; **Record Level:** institutionCode: MZHF
- **Type status:** Other material. **Occurrence:** recordedBy: W.Hackman; individualCount: 1; sex: male; **Location:** country: Finland; stateProvince: Karelia borealis; municipality: Kitee; locality: Närsäkkälä; decimalLatitude: 61.918; decimalLongitude: 30.138; geodeticDatum: WGS84; **Identification:** identifiedBy: W.Hackman; **Event:** eventDate: 1962-5; **Record Level:** institutionCode: MZHF
- **Type status:** Other material. **Occurrence:** recordedBy: P.Vilkamaa; individualCount: 1; sex: male; **Location:** country: Finland; stateProvince: Nylandia; municipality: Sipoo; locality: Hindsby, Gillerberget; decimalLatitude: 60.344; decimalLongitude: 25.191; geodeticDatum: WGS84; **Identification:** identifiedBy: J.Jakovlev; **Event:** samplingProtocol: Malaise trap; eventDate: 2004-7-6/7-16; habitat: old-growth forest, herb-rich type; **Record Level:** institutionCode: JJH
- **Type status:** Other material. **Occurrence:** recordedBy: J.Jakovlev; individualCount: 2; sex: 1 male, 1 female; **Location:** country: Finland; stateProvince: Nylandia; municipality: Sipoo; locality: Hindsby, Gladersеker; decimalLatitude: 60.339; decimalLongitude: 25.218; geodeticDatum: WGS84; **Identification:** identifiedBy: J.Jakovlev; **Event:** samplingProtocol: Malaise trap; eventDate: 2005-7-21/7-25; habitat: old-growth forest, herb-rich type; **Record Level:** institutionCode: JJH
- **Type status:** Other material. **Occurrence:** recordedBy: J.Jakovlev and G.Ståhls; individualCount: 1; sex: male; **Location:** country: Finland; stateProvince: Regio aboënsis; municipality: Turku; locality: Ruissalo-1; decimalLatitude: 60.432; decimalLongitude: 22.165; geodeticDatum: WGS84; **Identification:** identifiedBy: J.Jakovlev; **Event:** samplingProtocol: Malaise trap; eventDate: 2005-7-13/8-27; habitat: old-growth forest, herb-rich type; **Record Level:** institutionCode: JJH
- **Type status:** Other material. **Occurrence:** recordedBy: J.Jakovlev and G.Ståhls; individualCount: 1; sex: male; **Location:** country: Finland; stateProvince: Regio aboënsis; municipality: Turku; locality: Ruissalo-2; decimalLatitude: 60.432; decimalLongitude: 22.158; geodeticDatum: WGS84; **Identification:** identifiedBy: J.Jakovlev; **Event:** samplingProtocol: Malaise trap; eventDate: 2005-5-11/6-20; habitat: old-growth forest, herb-rich type; **Record Level:** institutionCode: JJH
- **Type status:** Other material. **Occurrence:** recordedBy: J.Jakovlev; individualCount: 1; sex: female; **Location:** country: Finland; stateProvince: Regio aboënsis; municipality: Karjalohja; locality: Karkali_North; decimalLatitude: 60.243; decimalLongitude: 23.797; geodeticDatum: WGS84; **Identification:** identifiedBy: J.Jakovlev; **Event:** samplingProtocol: Reared from wood; eventDate: 2007-5-16/6-25; habitat: old-growth forest, herb-rich type; **Record Level:** institutionCode: JJH
- **Type status:** Other material. **Occurrence:** recordedBy: J.Jakovlev; individualCount: 2; sex: male; **Location:** country: Finland; stateProvince: Nylandia; municipality: Kirkkonummi; locality: Kuokkamaa; decimalLatitude: 60.121; decimalLongitude: 24.608; geodeticDatum: WGS84; **Identification:** identifiedBy: J.Jakovlev; **Event:** samplingProtocol: Malaise trap; eventDate: 2010-6-17/7-13; habitat: old-growth forest, herb-rich type; **Record Level:** institutionCode: JJH
- **Type status:** Other material. **Occurrence:** recordedBy: J.Jakovlev; individualCount: 2; sex: male; **Location:** country: Finland; stateProvince: Nylandia; municipality: Kirkkonummi; locality: Norra flaget; decimalLatitude: 60.113; decimalLongitude: 24.624; geodeticDatum: WGS84; **Identification:** identifiedBy: J.Jakovlev; **Event:** samplingProtocol: Malaise trap; eventDate: 2010-7-13/8-23; habitat: old-growth forest, herb-rich type; **Record Level:** institutionCode: JJH
- **Type status:** Other material. **Occurrence:** recordedBy: J.Jakovlev; individualCount: 1; sex: female; **Location:** country: Finland; stateProvince: Regio aboënsis; municipality: Tammisaari; locality: Dragsvikin kartano; decimalLatitude: 60.001; decimalLongitude: 23.492; geodeticDatum: WGS84; **Identification:** identifiedBy: J.Jakovlev; **Event:** samplingProtocol: Malaise trap; eventDate: 2010-6-18/7-14; habitat: herb-rich forest, spruce dominated,huge willows.; **Record Level:** institutionCode: JJH
- **Type status:** Other material. **Occurrence:** recordedBy: J.Jakovlev; individualCount: 1; sex: male; **Location:** country: Finland; stateProvince: Regio aboënsis; municipality: Tammisaari; locality: Dragsvikin kartano; decimalLatitude: 60.001; decimalLongitude: 23.492; geodeticDatum: WGS84; **Identification:** identifiedBy: J.Jakovlev; **Event:** samplingProtocol: Malaise trap; eventDate: 2010-6-18/7-14; habitat: herb-rich forest, spruce dominated,huge willows; **Record Level:** institutionCode: JJH

#### Distribution

European, wide range in Europe (Chandler 2004). Rather common in herb-rich forests in South Finland, no former records from Finnish Lapland.

#### Ecology

Immature stages are unknown, but adults have been reared from moss patches and exposed forest soil in Norway (Økland 1999) and from an aspen log bearing polypores (J. Jakovlev, unpublished). However, due to the methods used (eclector trap *in situ*), it can not be ruled out that adult specimens were already present in the substrate when the trap was set (Økland 1999). Congeneric species *Gnoriste apicalis* Meigen, 1818 has been reared from moss cushions around a seepage in Germany (Lenz 1927).

### Syntemna penicilla

Hutson, 1979**

http://www.faunaeur.org/full_results.php?id=140711

#### Materials

- **Type status:** Other material. **Occurrence:** catalogNumber: MYCE-JS-2012-0032; recordedBy: J. Salmela; individualCount: 1; sex: male; **Location:** country: Finland; stateProvince: Lapponia kemensis pars orientalis; verbatimLocality: Savukoski, Törmäoja; decimalLatitude: 67.835; decimalLongitude: 29.454; geodeticDatum: WGS84; **Identification:** identifiedBy: J. Salmela; **Event:** samplingProtocol: Malaise trap; eventDate: 2012-6-14/7-10; habitat: headwater stream, old-growth boreal forest; **Record Level:** institutionCode: JES
- **Type status:** Other material. **Occurrence:** recordedBy: A. Polevoi; individualCount: 1; sex: male; **Location:** country: Russia; stateProvince: Republic Karelia; verbatimLocality: Paanajarvi, 4,5 km N of lake Pihlajarvi; decimalLatitude: 66.375; decimalLongitude: 30.367; geodeticDatum: WGS84; **Identification:** identifiedBy: A. Polevoi; **Event:** samplingProtocol: Malaise trap; eventDate: 2001-6-11/14; **Record Level:** institutionCode: FRIP
- **Type status:** Other material. **Occurrence:** recordedBy: G.Varkonyi; individualCount: 2; sex: male; **Location:** country: Finland; stateProvince: Ostrobothnia kajanensis; municipality: Puolanka; locality: Paljakka Strict Nature Reserve; decimalLatitude: 64.744; decimalLongitude: 28.050; geodeticDatum: WGS84; **Identification:** identifiedBy: J.Jakovlev; **Event:** samplingProtocol: reared from pine log, Eclector_trap; startDayOfYear: 2004-8-4/8-14; habitat: old-growth forest, Myrtillus type; **Record Level:** institutionCode: JJH
- **Type status:** Other material. **Occurrence:** recordedBy: G.Varkonyi; individualCount: 1; sex: male; **Location:** country: Finland; stateProvince: Ostrobothnia kajanensis; municipality: Puolanka; locality: Paljakka Strict Nature Reserve; decimalLatitude: 64.744; decimalLongitude: 28.050; geodeticDatum: WGS84; **Identification:** identifiedBy: J.Jakovlev; **Event:** samplingProtocol: Reared from wood; startDayOfYear: 2004-8-4/8-14; **Record Level:** institutionCode: JJH
- **Type status:** Other material. **Occurrence:** catalogNumber: MYCE-JS-2013-0397; recordedBy: J. Salmela; T. Hietajärvi; individualCount: 2; sex: male; **Location:** country: Finland; stateProvince: Regio kuusamoensis; verbatimLocality: Salla, Kuntasjoki, Värriö Strict Nature Reserve; verbatimElevation: 320 m; decimalLatitude: 67.749; decimalLongitude: 29.617; geodeticDatum: WGS84; **Identification:** identifiedBy: J. Salmela; **Event:** samplingProtocol: Malaise trap; eventDate: 2013; verbatimEventDate: 2013-6-4/29; habitat: headwater stream, old-growth boreal forest; **Record Level:** institutionCode: JES

#### Distribution

Fennoscandian. Only known from Finland (Hutson 1979, Polevoi 2003), Sweden (Kjaerandsen et al. 2007) and Norway (Søli and Rindal 2012). New to the Republic of Karelia and Russia.

#### Ecology

The Karelian specimen was collected in moist sprucedominated forest. Saproxylic, reared from a rotting pine log (Jakovlev 2011a).

#### Conservation

Red-listed in Finland (VU, Penttinen et al. 2010), associated with old-growth boreal forests.

### Docosia expectata

Laštovka & Ševčík, 2006*

http://www.catalogueoflife.org/col/details/species/id/8763254

#### Materials

- **Type status:** Other material. **Occurrence:** recordedBy: M.Jaschhof and C.Jaschhof; individualCount: 1; sex: male; **Location:** country: Finland; stateProvince: Regio aboënsis; municipality: Turku; locality: Ruissalo; decimalLatitude: 60.434; decimalLongitude: 22.272; geodeticDatum: WGS84; **Identification:** identifiedBy: J.Jakovlev; **Event:** samplingProtocol: Sweep netting; eventDate: 2004-6-27/6-27; habitat: old-growth forest, herb-rich type; **Record Level:** institutionCode: JJH

#### Distribution

European. Known from Czech Republic, Slovakia (Laštovka and Ševčík 2006), Great Britain (Hutson et al. 1980, as *Docosia* sp. indet), Sweden and Germany (Kurina et al. 2004, Kjaerandsen et al. 2007). New for Finland.

#### Ecology

The only Finnish record is from a herb-rich old-growth forest in the hemiboreal zone. Immature stages are unknown. Generally, *Docosia* larvae develope in a variety of microhabitats, including fungi, fungus infested wood, other vegetable matter and the nests of birds and mammals (Hutson et al. 1980).

### Docosia flavicoxa

Strobl, 1900

http://www.faunaeur.org/full_results.php?id=140664

#### Materials

- **Type status:** Other material. **Occurrence:** catalogNumber: MYCE-JS-2013-0118; recordedBy: J. Salmela; individualCount: 1; sex: male; **Location:** country: Finland; stateProvince: Lapponia kemensis pars orientalis; verbatimLocality: Savukoski, Joutenoja; decimalLatitude: 67.821; decimalLongitude: 29.440; geodeticDatum: WGS84; **Identification:** identifiedBy: J. Salmela; **Event:** samplingProtocol: Malaise trap; eventDate: 2012-8-16/9-18; habitat: headwater stream, seminatural boreal forest; **Record Level:** institutionCode: JES
- **Type status:** Other material. **Occurrence:** recordedBy: R.Frey; individualCount: 1; sex: male; **Location:** country: Finland; stateProvince: Alandia; locality: Finström; decimalLatitude: 60.234; decimalLongitude: 19.984; geodeticDatum: WGS84; **Identification:** identifiedBy: J.Jakovlev; **Event:** eventDate: 1900; **Record Level:** institutionCode: MZHF

#### Distribution

European species, known from Central Europe, British Isles (Laštovka and Ševčík 2006) and Fennoscandia (Kjaerandsen 2012). The only Finnish record is Hackman’s (Hackman 1980) checklist, where the species was included with a question mark. Here we confirm two records from Finland (the localities are in the hemiboreal and north boreal zones).

#### Ecology

The collecting site in Savukoski (Fig. 5c), eastern Lapland, is a headwater stream surrounded by seminatural coniferous forest. Immature stages are unknown.

### Docosia landrocki

Laštovka & Ševčík, 2006*

http://www.catalogueoflife.org/col/details/species/id/8763255

#### Materials

- **Type status:** Other material. **Occurrence:** recordedBy: R.Frey; individualCount: 1; sex: male; **Location:** country: Finland; stateProvince: Nylandia; municipality: Helsinki; locality: Munkkiniemi; decimalLatitude: 60.205; decimalLongitude: 24.868; geodeticDatum: WGS84; **Identification:** identifiedBy: J.Jakovlev; **Event:** eventDate: 1900; **Record Level:** institutionCode: MZHF
- **Type status:** Other material. **Occurrence:** recordedBy: J.Jakovlev; individualCount: 3; sex: male; **Location:** country: Finland; stateProvince: Nylandia; municipality: Helsinki; locality: Itäsalmi; decimalLatitude: 60.252; decimalLongitude: 25.204; geodeticDatum: WGS84; **Identification:** identifiedBy: J.Jakovlev; **Event:** samplingProtocol: Malaise trap; eventDate: 2010-6-15/7-23; habitat: herb-rich forest, dominated by aspen and spruce, plenty of young maple trees; **Record Level:** institutionCode: JJH

#### Distribution

European, known from central and western Europe (Laštovka and Ševčík 2006). In Fennoscandia hitherto only known from Sweden (preliminary record, Kjaerandsen 2012), new for Finland. Finnish records are from southernmost Finland, hemiboreal zone.

#### Ecology

Collected from a herb-rich forest. Immature stages are unknown.

### Docosia muelleri

Plassmann, 1986*

http://www.faunaeur.org/full_results.php?id=140675

#### Materials

- **Type status:** Other material. **Occurrence:** catalogNumber: MYCE-JS-2013-0195; recordedBy: J. Salmela; individualCount: 1; sex: male; **Location:** country: Finland; stateProvince: Lapponia kemensis pars orientalis; verbatimLocality: Savukoski, Törmäoja; decimalLatitude: 67.835; decimalLongitude: 29.454; geodeticDatum: WGS84; **Identification:** identifiedBy: J. Salmela; **Event:** samplingProtocol: Malaise trap; eventDate: 2012-7-10/8-16; habitat: headwater stream, old-growth boreal forest; **Record Level:** institutionCode: JES
- **Type status:** Other material. **Occurrence:** recordedBy: J.Jakovlev and J.Penttinen; individualCount: 1; sex: male; **Location:** stateProvince: Lapponia enontekiensis; municipality: Enontekiö; locality: Kilpisjärvi_Saana_North_3; decimalLatitude: 69.045; decimalLongitude: 20.807; geodeticDatum: WGS84; **Identification:** identifiedBy: J.Jakovlev; **Event:** samplingProtocol: Malaise trap; eventDate: 2006-8-1/8-15; habitat: mountain birch forest
- **Type status:** Other material. **Occurrence:** recordedBy: J.Jakovlev and J.Penttinen; individualCount: 1; sex: male; **Location:** stateProvince: Lapponia enontekiensis; municipality: Enontekiö; locality: Kilpisjärvi_Saana_North_3; decimalLatitude: 69.045; decimalLongitude: 20.807; geodeticDatum: WGS84; **Identification:** identifiedBy: J.Jakovlev; **Event:** samplingProtocol: Malaise trap; eventDate: 2006-8-15/8-31; habitat: mountain birch forest
- **Type status:** Other material. **Occurrence:** recordedBy: J.Jakovlev and J.Penttinen; individualCount: 1; sex: male; **Location:** stateProvince: Lapponia enontekiensis; municipality: Enontekiö; locality: Kilpisjärvi_Saana_South_1; decimalLatitude: 69.033; decimalLongitude: 20.838; geodeticDatum: WGS84; **Identification:** identifiedBy: J.Penttinen; **Event:** samplingProtocol: Malaise trap; eventDate: 2006-6-19/7-14; habitat: mountain birch forest; **Record Level:** institutionCode: JPJ
- **Type status:** Other material. **Occurrence:** recordedBy: J.Jakovlev and J.Penttinen; individualCount: 1; sex: male; **Location:** stateProvince: Lapponia kemensis pars occidentalis; municipality: Muonio; locality: Pallas-Yllästunturi National Park; decimalLatitude: 67.587; decimalLongitude: 24.214; geodeticDatum: WGS84; **Identification:** identifiedBy: J.Jakovlev; **Event:** samplingProtocol: Malaise trap; eventDate: 2006-7-15/8-15; habitat: old-growth forest, Myrtillus type

#### Distribution

Palaearctic. *Docosia muelleri* (Fig. 18) is in Fennoscandia so far known only by the type material from northern Sweden (Plassmann 1986). No former records from Finland.

#### Ecology

Current knowledge based on a few findings suggests a northern distribution. Finnish records are from subarctic mountain birch forest on the slopes of Saana mountain, from old-growth coniferous stands close to the timberline (Pallas-Yllästunturi National Park) and from riparian forests (Savukoski). Immature stages are unknown.

#### Taxon discussion

It was recently noted that *Docosia moravica* Landrock sensu Zaitzev (Zaitzev 1994, p. 254, Fig. 81: 9) most likely represents *Docosia muelleri* (Kurina and Ševčík 2012). We studied the holotype of *Docosia muelleri* (Sweden, Abisko) and specimens identified as *Docosia moravica* by A.Zaitzev (West Siberia: Nizhnyaya Tunguska and Norilsk) and can confirm that the species are identical. Male genitalia of real *Docosia moravica* were figured by Laštovka and Ševčík (Laštovka and Ševčík 2006) who studied Landrock's specimens and designated lectotype.

### Docosia tibialis

Laštovka & Ševčík, 2006*

http://www.catalogueoflife.org/col/details/species/id/8763261

#### Materials

- **Type status:** Other material. **Occurrence:** recordedBy: R.Tuomikoski; individualCount: 1; sex: male; **Location:** country: Finland; stateProvince: Satakunta; municipality: Kokemäki; decimalLatitude: 61.254; decimalLongitude: 22.317; geodeticDatum: WGS84; **Identification:** identifiedBy: J.Jakovlev; **Event:** samplingProtocol: Sweep netting; eventDate: 1953; **Record Level:** institutionCode: MZHF

#### Distribution

European. Described recently from Czech Republic and Italy (Laštovka and Ševčík 2006), and later recorded from Sweden (Kjaerandsen 2012) and northwest Russia (Polevoi 2010). New for Finland.

#### Ecology

Immature stages are unknown.

### Greenomyia baikalica

Zaitzev, 1994

http://www.faunaeur.org/full_results.php?id=140656

#### Materials

- **Type status:** Other material. **Occurrence:** catalogNumber: MYCE-JS-2013-0195; recordedBy: J. Salmela; individualCount: 1; sex: male; **Location:** country: Finland; stateProvince: Lapponia kemensis pars orientalis; verbatimLocality: Savukoski, Törmäoja; decimalLatitude: 67.835; decimalLongitude: 29.454; geodeticDatum: WGS84; **Identification:** identifiedBy: J. Salmela; **Event:** samplingProtocol: Malaise trap; eventDate: 2012-7-10/8-16; habitat: headwater stream, old-growth boreal forest; **Record Level:** institutionCode: JES
- **Type status:** Other material. **Occurrence:** catalogNumber: MYCE-JS-2013-0185; recordedBy: J. Salmela; individualCount: 2; sex: 1 male, 1 female; **Location:** country: Finland; stateProvince: Lapponia kemensis pars orientalis; verbatimLocality: Savukoski, Joutenoja; decimalLatitude: 67.821; decimalLongitude: 29.440; geodeticDatum: WGS84; **Identification:** identifiedBy: J. Salmela; **Event:** samplingProtocol: Malaise trap; eventDate: 2012-7-10/8-16; habitat: headwater stream, seminatural boreal forest; **Record Level:** institutionCode: JES
- **Type status:** Other material. **Occurrence:** catalogNumber: MYCE-JS-2012-0057; recordedBy: J. Salmela; individualCount: 1; sex: male; **Location:** country: Finland; stateProvince: Ostrobothnia borealis pars borealis; verbatimLocality: Ylitornio, Tuorerommas; decimalLatitude: 66.478; decimalLongitude: 24.753; geodeticDatum: WGS84; **Identification:** identifiedBy: J. Salmela; **Event:** samplingProtocol: Malaise trap; eventDate: 2012-8-6/9-26; habitat: spring brook, old-growth boreal forest; **Record Level:** institutionCode: JES
- **Type status:** Other material. **Occurrence:** recordedBy: M.Jaschhof and C.Jaschhof; individualCount: 1; sex: male; **Location:** country: Finland; stateProvince: Regio kuusamoënsis; municipality: Kuusamo; locality: Kuohusuo-Kalliovaara; decimalLatitude: 65.674; decimalLongitude: 28.888; geodeticDatum: WGS84; **Identification:** identifiedBy: J.Jakovlev; **Event:** samplingProtocol: Sweep netting; eventDate: 2004-7-21/7-25; habitat: old-growth forest, Myrtillus type; **Record Level:** institutionCode: JJH
- **Type status:** Other material. **Occurrence:** recordedBy: J.Jakovlev; individualCount: 1; sex: male; **Location:** country: Finland; stateProvince: Tavastia australis; municipality: Lammi; locality: Evo_Kotinen_Aspen part; decimalLatitude: 61.244; decimalLongitude: 25.067; geodeticDatum: WGS84; **Identification:** identifiedBy: J.Jakovlev; **Event:** samplingProtocol: Malaise trap; eventDate: 2004-8-28/10-4; habitat: old-growth forest, herb-rich type; **Record Level:** institutionCode: JJH
- **Type status:** Other material. **Occurrence:** recordedBy: J.Jakovlev and J.Penttinen; individualCount: 1; sex: female; **Location:** country: Finland; stateProvince: Tavastia australis; municipality: Lammi; locality: Evo_Kotinen_Aspen part; decimalLatitude: 61.244; decimalLongitude: 25.067; geodeticDatum: WGS84; **Identification:** identifiedBy: J.Jakovlev; **Event:** samplingProtocol: Rearing from wood; eventDate: 2006-6-28/8-2; habitat: old-growth forest, herb-rich type; **Record Level:** institutionCode: JJH
- **Type status:** Other material. **Occurrence:** recordedBy: Noora Vartija; individualCount: 1; sex: male; **Location:** country: Finland; stateProvince: Tavastia australis; municipality: Muurame; locality: Kuusimäki Forest Reserve; decimalLatitude: 62.2125; decimalLongitude: 25.497; geodeticDatum: WGS84; **Identification:** identifiedBy: J.Penttinen; **Event:** samplingProtocol: Reared from wood; eventDate: 2007-7-27/8-27; habitat: old-growth forest, Myrtillus type; **Record Level:** institutionCode: JPJ
- **Type status:** Other material. **Occurrence:** recordedBy: J.Jakovlev; individualCount: 1; sex: male; **Location:** country: Finland; stateProvince: Tavastia australis; municipality: Padasjoki; locality: Vesijako Strict Nature Reserve; decimalLatitude: 61.355; decimalLongitude: 25.106; geodeticDatum: WGS84; **Identification:** identifiedBy: J.Jakovlev; **Event:** samplingProtocol: Sweep netting; eventDate: 2008-8-18/8-18; habitat: old-growth forest, Myrtillus type; **Record Level:** institutionCode: JJH
- **Type status:** Other material. **Occurrence:** catalogNumber: DIPT-JS-2014-0055; recordedBy: J. Salmela; T. Hietajärvi; individualCount: 1; sex: male; **Location:** country: Finland; stateProvince: Regio kuusamoensis; verbatimLocality: Salla, Kuntasjoki, Värriö Strict Nature Reserve; verbatimElevation: 320 m; decimalLatitude: 67.749; decimalLongitude: 29.617; geodeticDatum: WGS84; **Identification:** identifiedBy: J. Salmela; **Event:** samplingProtocol: Malaise trap; verbatimEventDate: 2013-7-29/9-19; habitat: headwater stream, old-growth boreal forest; **Record Level:** institutionCode: JES

#### Distribution

Palaearctic. The species was described from Siberia, Buryatia (Zaitzev 1994) and has been subsequently found only from Fennoscandia (Polevoi 2000, Kjaerandsen et al. 2007, Kurina et al. 2011).

#### Ecology

Collected from seminatural or old-growth boreal forests, larvae are associated with saproxylic fungi (Zaitzev 1994). In Finland has been collected with an eclector trap over aspen log bearing polypores *Phellinus tremulae* and *Trametes ochraceae* (J. Jakovlev, unpublished).

#### Conservation

Red-listed in Finland (VU, Penttinen et al. 2010) and Norway (VU, Anonymous 2010, Gammelmo et al. 2010).

### Greenomyia mongolica

Lastovka & Matile, 1974*

http://www.catalogueoflife.org/col/details/species/id/8734367

#### Materials

- **Type status:** Other material. **Occurrence:** recordedBy: Elina Peuhu; individualID: 1; individualCount: 1; sex: female; **Location:** country: Finland; stateProvince: Nylandia; municipality: Helsinki; locality: Herttoniemen kartanopuisto; decimalLatitude: 60.190; decimalLongitude: 25.042; geodeticDatum: WGS84; **Identification:** identifiedBy: J.Jakovlev; **Event:** samplingProtocol: pit-fall trap inside a hollow lime tree (Tilia cordata); eventDate: 2006-7-6/7-19; habitat: old managed forest, herb-rich type; **Record Level:** institutionCode: JJH
- **Type status:** Other material. **Occurrence:** recordedBy: Elina Peuhu; individualCount: 1; sex: male; **Location:** country: Finland; stateProvince: Nylandia; municipality: Helsinki; locality: Tuomarinkylä; decimalLatitude: 60.262; decimalLongitude: 24.966; geodeticDatum: WGS84; **Identification:** identifiedBy: J.Jakovlev; **Event:** samplingProtocol: bowl window trap; eventDate: 2006-7-4/7-18; habitat: Wood-storage areas in Helsinki; **Record Level:** institutionCode: JJH
- **Type status:** Other material. **Occurrence:** recordedBy: J.Jakovlev; individualCount: 11; sex: 7 males, 4 females; **Location:** country: Finland; stateProvince: Nylandia; municipality: Helsinki; locality: Tuomarinkylä; decimalLatitude: 60.262; decimalLongitude: 24.966; geodeticDatum: WGS84; **Identification:** identifiedBy: J.Jakovlev; **Event:** samplingProtocol: Malaise trap; eventDate: 2011-6-5/7-4; habitat: Wood-storage areas in Helsinki; **Record Level:** institutionCode: JJH
- **Type status:** Other material. **Occurrence:** recordedBy: J.Jakovlev; individualCount: 33; sex: 21 males, 12 females; **Location:** country: Finland; stateProvince: Nylandia; municipality: Helsinki; locality: Tuomarinkylä; decimalLatitude: 60.262; decimalLongitude: 24.966; geodeticDatum: WGS84; **Identification:** identifiedBy: J.Jakovlev; **Event:** samplingProtocol: Malaise trap; eventDate: 2011-7-5/7-20; habitat: Wood-storage areas in Helsinki; **Record Level:** institutionCode: JJH
- **Type status:** Other material. **Occurrence:** recordedBy: J.Jakovlev; individualCount: 14; sex: 10 males, 4 females; **Location:** country: Finland; stateProvince: Nylandia; municipality: Helsinki; locality: Tuomarinkylä; decimalLatitude: 60.262; decimalLongitude: 24.966; geodeticDatum: WGS84; **Identification:** identifiedBy: J.Jakovlev; **Event:** samplingProtocol: Malaise trap; eventDate: 2011-7-5/7-20; habitat: Wood-storage areas in Helsinki; **Record Level:** institutionCode: JJH
- **Type status:** Other material. **Occurrence:** recordedBy: J.Jakovlev; individualCount: 17; sex: 8 males, 9 females; **Location:** country: Finland; stateProvince: Nylandia; municipality: Helsinki; locality: Tuomarinkylä; decimalLatitude: 60.262; decimalLongitude: 24.966; geodeticDatum: WGS84; **Identification:** identifiedBy: J.Jakovlev; **Event:** samplingProtocol: Malaise trap; eventDate: 2011-7-5/7-20; habitat: Wood-storage areas in Helsinki; **Record Level:** institutionCode: JJH
- **Type status:** Other material. **Occurrence:** recordedBy: J.Jakovlev; individualCount: 8; sex: 4 males, 4 females; **Location:** country: Finland; stateProvince: Nylandia; municipality: Helsinki; locality: Tuomarinkylä; decimalLatitude: 60.262; decimalLongitude: 24.966; geodeticDatum: WGS84; **Identification:** identifiedBy: J.Jakovlev; **Event:** samplingProtocol: Malaise trap; eventDate: 2011-7-5/7-20; habitat: Wood-storage areas in Helsinki; **Record Level:** institutionCode: JJH

#### Distribution

**Figure 19.**
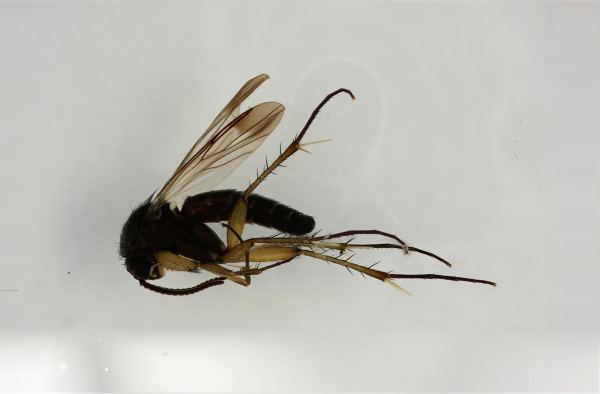
*Greenomyia mongolica* Lastovka & Matile, male specimen collected from Finland, lateral view.

Palaearctic. *Greenomyia mongolica* (Fig. 19) is known from Mongolia (Laštovka and Matile 1974), Russia (Zaitzev 1994), Estonia, southern and central Europe (Chandler 2004, as *Greenomyia theresae* Matile, 2002, Kurina et al. 2011), Spain (Chandler and Camaño Portela 2011) and Britain (Chandler 2008). In Nordic countries collected from the southern parts of Sweden and Norway (Soli et al. 2009, Anonymous 2010, Kurina et al. 2011). No previous findings from Finland.

#### Ecology

Larvae are saproxylic, apparently feeding on mycelia in decaying wood (Zaitzev 1994). Finnish specimens were collected in wood-storage areas in the city parks of Helsinki. Interestingly, a related species, *Greenomyia stackelbergi* Zaitzev was also found only in semi-urban habitats in Norway and Sweden; in Sweden the larvae had probably developed in a garden compost in which fungal fruiting bodies were regularly discarded by the mycologist M. Karström (Søli and Kjaerandsen 2008).

### Leia longiseta

Barendrecht, 1938*

http://www.faunaeur.org/full_results.php?id=140637

#### Materials

- **Type status:** Other material. **Occurrence:** catalogNumber: MYCE-JS-2013-0270; recordedBy: J. Ilmonen; individualCount: 73; sex: 53 males, 20 females; otherCatalogNumbers: MYCE-JS-2013-0284; **Location:** country: Finland; stateProvince: Nylandia; municipality: Espoo; locality: Matalajärvi; decimalLatitude: 60.247; decimalLongitude: 24.687; geodeticDatum: WGS84; **Identification:** identifiedBy: J. Salmela; **Event:** samplingProtocol: Malaise trap; eventDate: 2012-5-9/10-20; habitat: swampy lake shore; **Record Level:** institutionCode: JES

#### Distribution

European. A rare and poorly known species, so far only known from the Netherlands (Barendrecht 1938), Great Britain (Chandler 1992), Norway (Anonymous 2010) and Germany (Plassmann 1988). The first record from the Baltic sea catchment area, new for Finland.

#### Ecology

Finnish sampling locality is a swampy lake shore with luxuriant vegetation. Collecting sites reported by Chandler (Chandler 1992) are wetlands and the only known Norwegian locality is a lake shore wetland (Anonymous 2010). Relatively long flying period, specimens were caught between May and October. However, 60% of the caught specimens were trapped between 23rd of August - 20th of October. Larval habitats are unknown. Generally, *Leia* larvae spin a slimy web on the under surface of fungi and dead wood (Jakovlev 2011a). In addition, some species have been reared from the nests of birds and mammals (Falk and Chandler 2005) and from tussocks of grasses and sedges (Ševčík and Roháček 2008).

#### Conservation

Red-listed in Norway (VU, Anonymous 2010, Gammelmo et al. 2010).

### Allodia (Brachycampta) bohemica

Ševčík, 2004**

http://www.faunaeur.org/full_results.php?id=304273

#### Materials

- **Type status:** Other material. **Occurrence:** recordedBy: A. Polevoi; individualCount: 1; sex: male; **Location:** country: Russia; stateProvince: Republic Karelia; verbatimLocality: Pin'guba; decimalLatitude: 61.865; decimalLongitude: 34.556; geodeticDatum: WGS84; **Identification:** identifiedBy: A. Polevoi; **Event:** samplingProtocol: Sweep netting; eventDate: 2007-6-17; **Record Level:** institutionCode: FRIP

#### Distribution

European. This species was known only from type locality in Czech Republic (Ševčík 2004). New to the Republic of Karelia, Russia, and Fennoscandia.

#### Ecology

Immature stages of this rare species are unknown. Generally, *Allodia* species for which rearing records exist are associated with fruiting bodies of soft macrofungi, chiefly agarics. Some *Allodia* species within the subgenus *Brachycampta* colonize ascomycete fungi of the order Pezizales (Jakovlev 1994, Jakovlev 2012).

### Allodia (Brachycampta) huggerti

Kjaerandsen, 2007*

http://www.catalogueoflife.org/col/details/species/id/8762838

#### Materials

- **Type status:** Other material. **Occurrence:** catalogNumber: MYCE-JS-2013-0296; recordedBy: J. Ilmonen; individualCount: 1; sex: male; **Location:** country: Finland; stateProvince: Nylandia; municipality: Espoo; locality: Matalajärvi; decimalLatitude: 60.247; decimalLongitude: 24.687; geodeticDatum: WGS84; **Identification:** identifiedBy: J. Salmela; **Event:** samplingProtocol: Malaise trap; eventDate: 2012-8-23/10-20; habitat: swampy lake shore; **Record Level:** institutionCode: JES

#### Distribution

**Figure 20.**
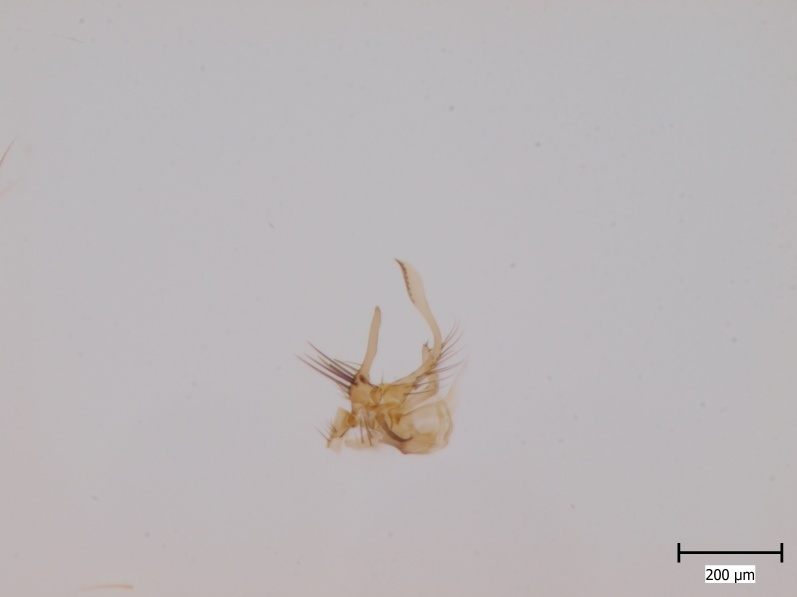
Allodia (Brachycampta) huggerti Kjaerandsen, male specimen collected from South Finland, Espoo. Male gonostylus, lateral view.

Fennoscandian. *Allodia huggerti* (Fig. 20) is a recently described species, hitherto only known from the type locality in South Sweden (Kjaerandsen 2007). New for Finland.

#### Ecology

Immature stages are unknown.

### Allodia (Brachycampta) penicillata

(Lundström, 1912)

http://www.faunaeur.org/full_results.php?id=140064

#### Materials

- **Type status:** Other material. **Occurrence:** recordedBy: M. Tietäväinen; individualCount: 2; sex: male; **Location:** country: Finland; stateProvince: Karelia borealis; verbatimLocality: Ilomantsi, Kotavaara; decimalLatitude: 63.029; decimalLongitude: 31.377; geodeticDatum: WGS84; **Identification:** identifiedBy: A. Polevoi; **Event:** samplingProtocol: Malaise trap; eventDate: 1998-6-11/15; **Record Level:** institutionCode: FRIP
- **Type status:** Other material. **Occurrence:** catalogNumber: MYCE-JS-2013-0189; recordedBy: J. Salmela; individualCount: 1; sex: male; **Location:** country: Finland; stateProvince: Lapponia kemensis pars orientalis; verbatimLocality: Savukoski, Joutenoja; decimalLatitude: 67.821; decimalLongitude: 29.440; geodeticDatum: WGS84; **Identification:** identifiedBy: J. Salmela; J. Jakovlev; **Event:** samplingProtocol: Malaise trap; eventDate: 2012-6-14/7-10; habitat: headwater stream, seminatural boreal forest; **Record Level:** institutionCode: JES
- **Type status:** Other material. **Occurrence:** catalogNumber: DIPT-JS-2014-0010; recordedBy: J. Salmela; T. Hietajärvi; individualCount: 1; sex: male; **Location:** country: Finland; stateProvince: Regio kuusamoensis; verbatimLocality: Salla, Kuntasjoki, Värriö Strict Nature Reserve; verbatimElevation: 320 m; decimalLatitude: 67.749; decimalLongitude: 29.617; geodeticDatum: WGS84; **Identification:** identifiedBy: J. Salmela; **Event:** samplingProtocol: Malaise trap; eventDate: 2013; verbatimEventDate: 2013-6-4/29; habitat: headwater stream, old-growth boreal forest; **Record Level:** institutionCode: JES

#### Distribution

European, known from Finland (as *Brachycampta penicillata*, Lundström 1912b), Russian Karelia (Polevoi 2000) and Latvia (Lackschewitz 1937). Very rare species, in Finland hitherto only recorded from the type locality in NW Lapland, Muonio (R. Frey leg. 1911, Lundström 1912b).

#### Ecology

Collected in *Vaccinium myrtillus* type spruce dominated forests and from riparian forests. Immature stages are unknown.

### Allodia (Brachycampta) subpistillata

Ševčík, 1999

http://www.faunaeur.org/full_results.php?id=140073

#### Materials

- **Type status:** Other material. **Occurrence:** catalogNumber: MYCE-JS-2013-0123; recordedBy: J. Salmela; individualCount: 1; sex: male; **Location:** country: Finland; stateProvince: Lapponia kemensis pars orientalis; verbatimLocality: Savukoski, Joutenoja; decimalLatitude: 67.821; decimalLongitude: 29.440; geodeticDatum: WGS84; **Identification:** identifiedBy: J. Salmela; **Event:** samplingProtocol: Malaise trap; eventDate: 2012-8-16/9-18; habitat: headwater stream, seminatural boreal forest; **Record Level:** institutionCode: JES
- **Type status:** Other material. **Occurrence:** catalogNumber: MYCE-JS-2013-0294; recordedBy: J. Ilmonen; individualCount: 1; sex: male; **Location:** country: Finland; stateProvince: Nylandia; municipality: Espoo; locality: Matalajärvi; decimalLatitude: 60.247; decimalLongitude: 24.687; geodeticDatum: WGS84; **Identification:** identifiedBy: J. Salmela; **Event:** samplingProtocol: Malaise trap; eventDate: 2012-8-23/10-20; habitat: swampy lake shore; **Record Level:** institutionCode: JES
- **Type status:** Other material. **Occurrence:** catalogNumber: MYCE-JS-2013-0311; recordedBy: J. Ilmonen; individualCount: 1; sex: male; **Location:** country: Finland; stateProvince: Nylandia; municipality: Espoo; locality: Matalajärvi; decimalLatitude: 60.247; decimalLongitude: 24.687; geodeticDatum: WGS84; **Identification:** identifiedBy: J. Salmela; **Event:** samplingProtocol: Malaise trap; eventDate: 2012-6-16/7-21; habitat: swampy lake shore; **Record Level:** institutionCode: JES

#### Distribution

European. The species was described from Czech Republic (Ševčík 1999), and has been recorded from Sweden (north, south, Kjaerandsen et al. 2007), Russian Karelia (Polevoi 2000) and Finland (south). Here reported from North Finland.

#### Ecology

Sampling sites are a headwater stream and a swampy lake shore. Immature stages are unknown. Related species Allodia (Brachycampta) pistillata Lundström was reared by Jakovlev (Jakovlev 1994) from the ascomycete fungus *Peziza* sp.

### Allodiopsis korolevi

Zaitzev, 1982*

http://www.faunaeur.org/full_results.php?id=140029

#### Materials

- **Type status:** Other material. **Occurrence:** recordedBy: R.Frey; individualCount: 1; sex: male; **Location:** country: Finland; stateProvince: Alandia; municipality: Sund; decimalLatitude: 60.250; decimalLongitude: 20.108; geodeticDatum: WGS84; **Identification:** identifiedBy: J.Jakovlev; **Event:** eventDate: 1900; **Record Level:** institutionCode: MZHF

#### Distribution

Palaearctic. Known from European and Asian parts of Russia (Zaitzev 2003), Great Britain, Switzerland and Romania (Chandler 2004). New for Finland.

#### Ecology

Immature stages are unknown. The larval microhabitats of *Allodiopsis* species are quite similar to those of *Allodia*. Some species colonise also Lycoperdales (Ševčík 2010, Jakovlev 2011a).

### Anatella bremia

Chandler, 1994

http://www.faunaeur.org/full_results.php?id=139996

#### Materials

- **Type status:** Other material. **Occurrence:** recordedBy: A. Polevoi; individualCount: 1; sex: male; **Location:** country: Russia; stateProvince: Leningrad province; verbatimLocality: Voznesenje, 1 km E of Gimreka; decimalLatitude: 61.151; decimalLongitude: 35.64; geodeticDatum: WGS84; **Identification:** identifiedBy: A. Polevoi; **Event:** samplingProtocol: Malaise trap; eventDate: 2008-4-23/5-25; **Record Level:** institutionCode: FRIP
- **Type status:** Other material. **Occurrence:** catalogNumber: MYCE-JS-2013-0332; recordedBy: J. Ilmonen; individualCount: 1; sex: male; **Location:** country: Finland; stateProvince: Nylandia; municipality: Espoo; locality: Matalajärvi; decimalLatitude: 60.247; decimalLongitude: 24.687; geodeticDatum: WGS84; **Identification:** identifiedBy: J. Salmela; **Event:** samplingProtocol: Malaise trap; eventDate: 2012-7-21/8-23; habitat: swampy lake shore; **Record Level:** institutionCode: JES

#### Distribution

European. Described from Great Britain (Chandler 1994) and later found from Norway (Anonymous 2010), Sweden (Kjaerandsen et al. 2007), Germany (Chandler 2004), Russia (Polevoi 2000, Zaitzev 2003) and Finland. In Finland recorded only once before, from the eastern part of the country (Karelia borealis, Polevoi 2001).

#### Ecology

In Britain the species is associated with wet meadows and peatlands (Falk and Chandler 2005). Finnish sampling sites are an abandoned field (Polevoi 2001) and a swampy lake shore (Matalajärvi). Karelian records are from *Cladonia* type pine forest and secondary *Vaccinium myrtillus* type pine dominated forest. Immature stages are unknown. The larval biology of *Anatella* is mostly unknown, the few known associations are with ascomycetes or other small wood-decay fungi (Alexander 2002, Ševčík 2010).

### Brevicornu arcticum

(Lundström, 1913)*

http://www.faunaeur.org/full_results.php?id=139931

#### Materials

- **Type status:** Other material. **Occurrence:** catalogNumber: MYCE-JS-2013-0078; recordedBy: J. Salmela; individualCount: 1; sex: male; **Location:** country: Finland; stateProvince: Lapponia kemensis pars orientalis; verbatimLocality: Savukoski, Joutenoja; decimalLatitude: 67.821; decimalLongitude: 29.440; geodeticDatum: WGS84; **Identification:** identifiedBy: J. Salmela; **Event:** samplingProtocol: Malaise trap; eventDate: 2012-8-16/9-18; habitat: headwater stream, seminatural boreal forest; **Record Level:** institutionCode: JES
- **Type status:** Other material. **Occurrence:** catalogNumber: MYCE-JS-2013-0041; recordedBy: J. Salmela; individualCount: 1; sex: male; **Location:** country: Finland; stateProvince: Lapponia kemensis pars orientalis; verbatimLocality: Savukoski, Törmäoja; decimalLatitude: 67.835; decimalLongitude: 29.454; geodeticDatum: WGS84; **Identification:** identifiedBy: J. Salmela; **Event:** samplingProtocol: Malaise trap; eventDate: 2012-7-10/8-16; habitat: headwater stream, old-growth boreal forest; **Record Level:** institutionCode: JES
- **Type status:** Other material. **Occurrence:** catalogNumber: MYCE-JS-2013-0156; recordedBy: J. Salmela; individualCount: 1; sex: male; **Location:** country: Finland; stateProvince: Lapponia kemensis pars orientalis; verbatimLocality: Savukoski, Törmäoja; decimalLatitude: 67.835; decimalLongitude: 29.454; geodeticDatum: WGS84; **Identification:** identifiedBy: J. Salmela; **Event:** samplingProtocol: Malaise trap; eventDate: 2012-8-16/9-18; habitat: headwater stream, old-growth boreal forest; **Record Level:** institutionCode: JES
- **Type status:** Other material. **Occurrence:** catalogNumber: DIPT-JS-2014-0028; recordedBy: J. Salmela; T. Hietajärvi; individualCount: 1; sex: male; **Location:** country: Finland; stateProvince: Regio kuusamoensis; verbatimLocality: Salla, Kuntasjoki, Värriö Strict Nature Reserve; verbatimElevation: 320 m; decimalLatitude: 67.749; decimalLongitude: 29.617; geodeticDatum: WGS84; **Identification:** identifiedBy: J. Salmela; **Event:** samplingProtocol: Malaise trap; eventDate: 2013; verbatimEventDate: 2013-6-4/29; habitat: headwater stream, old-growth boreal forest; **Record Level:** institutionCode: JES

#### Distribution

Holarctic, known from arctic Russia (Kanin Peninsula, as *Brachycampta arctica*, Lundström and Frey 1913), Russian Karelia and Murmansk region (Polevoi 2000, Polevoi 2010), Ireland (Chandler 1977), Central Europe (Chandler 2004) and USA (Zaitzev 1988). In the Nordic countries recorded from Sweden and Norway (Kjaerandsen 2012). Perhaps a boreo-montane species (Kjaerandsen et al. 2007). New for Finland.

#### Ecology

Finnish sampling localities are headwater streams surrounded by pristine or seminatural boreal forests. Immature stages are unknown. In their larval habitats, *Brevicornu* do not resemble closely related species of *Allodia*. At least some *Brevicornu* species develop in dead wood and in soil litter, feeding probably on microfungi (Jakovlev 2011a).

### Brevicornu auriculatum

(Edwards, 1925)*

http://www.faunaeur.org/full_results.php?id=139932

#### Materials

- **Type status:** Other material. **Occurrence:** catalogNumber: MYCE-JS-2013-0158; recordedBy: J. Salmela; individualCount: 1; sex: male; **Location:** country: Finland; stateProvince: Lapponia kemensis pars orientalis; verbatimLocality: Savukoski, Törmäoja; decimalLatitude: 67.835; decimalLongitude: 29.454; geodeticDatum: WGS84; **Identification:** identifiedBy: J. Salmela, A. Polevoi; **Event:** samplingProtocol: Malaise trap; eventDate: 2012-8-16/9-18; habitat: headwater stream, old-growth boreal forest; **Record Level:** institutionCode: JES
- **Type status:** Other material. **Occurrence:** recordedBy: J.Jakovlev and J.Penttinen; individualCount: 2; sex: male; **Location:** country: Finland; stateProvince: Lapponia enontekiensis; municipality: Enontekiö; locality: Kilpisjärvi_Saana_South_2; decimalLatitude: 69.035; decimalLongitude: 20.839; geodeticDatum: WGS84; **Identification:** identifiedBy: J.Jakovlev; **Event:** samplingProtocol: Malaise trap; eventDate: 2006-8-1/8-15; habitat: subarctic
- **Type status:** Other material. **Occurrence:** recordedBy: J.Jakovlev and J.Penttinen; individualCount: 1; sex: male; **Location:** country: Finland; stateProvince: Lapponia enontekiensis; municipality: Enontekiö; locality: Kilpisjärvi_Saana_North_4; decimalLatitude: 69.045; decimalLongitude: 20.808; geodeticDatum: WGS84; **Identification:** identifiedBy: J.Jakovlev; **Event:** samplingProtocol: Malaise trap; eventDate: 2006-7-15/8-1; habitat: subarctic

#### Distribution

Palaearctic. Rather widespread in Europe (Chandler 2004), also recorded from the Russian Far East (Kuril Islands, Zaitzev 2003), but so far not recorded from other parts of Russia. In the Nordic countries recorded from Sweden, Norway, Iceland and Denmark (Kjaerandsen 2012, Søli and Rindal 2012). Here reported for the first time from Finland.

#### Ecology

Immature stages are unknown. Finnish collecting sites are located in Lapland, Savukoski (north boreal zone, riparian forest) and Kilpisjärvi (subarctic zone, mountain birch forest).

### Brevicornu cognatum

Ostroverkhova, 1979*

http://www.catalogueoflife.org/col/details/species/id/8698225

#### Materials

- **Type status:** Other material. **Occurrence:** catalogNumber: MYCE-JS-2013-0290; recordedBy: J. Ilmonen; individualCount: 2; sex: male; otherCatalogNumbers: MYCE-JS-2013-0308; **Location:** country: Finland; stateProvince: Nylandia; municipality: Espoo; locality: Matalajärvi; decimalLatitude: 60.247; decimalLongitude: 24.687; geodeticDatum: WGS84; **Identification:** identifiedBy: J. Salmela; **Event:** samplingProtocol: Malaise trap; eventDate: 2012-8-23/10-20; habitat: swampy lake shore; **Record Level:** institutionCode: JES
- **Type status:** Other material. **Occurrence:** catalogNumber: MYCE-JS-2013-0358; recordedBy: J. Salmela; individualCount: 2; sex: male; **Location:** country: Finland; stateProvince: Lapponia kemensis pars orientalis; municipality: Savukoski; locality: Törmäoja, Ahot; decimalLatitude: 67.827; decimalLongitude: 29.437; geodeticDatum: WGS84; **Identification:** identifiedBy: J. Salmela; **Event:** samplingProtocol: Malaise trap; eventDate: 2013-8-7/9-19; habitat: Carex swamp; **Record Level:** institutionCode: JES
- **Type status:** Other material. **Occurrence:** recordedBy: J.Jakovlev and J.Penttinen; individualCount: 1; sex: male; **Location:** country: Finland; stateProvince: Lapponia enontekiensis; municipality: Enontekiö; locality: Kilpisjärvi_Saana_South_1; decimalLatitude: 69.033; decimalLongitude: 20.838; geodeticDatum: WGS84; **Identification:** identifiedBy: J.Penttinen; **Event:** samplingProtocol: Malaise trap; eventDate: 2006-6-19/7-15; habitat: Subarctic; **Record Level:** institutionCode: JPJ
- **Type status:** Other material. **Occurrence:** recordedBy: J.Penttinen; individualCount: 1; sex: male; **Location:** country: Finland; stateProvince: Tavastia australis; municipality: Hämeenlinna; locality: Tenhola; decimalLatitude: 60.980; decimalLongitude: 23.718; geodeticDatum: WGS84; **Identification:** identifiedBy: J.Penttinen; **Event:** samplingProtocol: Malaise trap; eventDate: 2009-6-15/8-15; habitat: old managed forest, herb-rich type; **Record Level:** institutionCode: JPJ
- **Type status:** Other material. **Occurrence:** recordedBy: J.Penttinen; individualCount: 1; sex: male; **Location:** country: Finland; stateProvince: Regio aboënsis; municipality: Salo; locality: Märy; decimalLatitude: 60.223; decimalLongitude: 22.905; geodeticDatum: WGS84; **Identification:** identifiedBy: J.Penttinen; **Event:** samplingProtocol: Malaise trap; eventDate: 2009-6-1/6-15; habitat: old managed forest, herb-rich type; **Record Level:** institutionCode: JPJ
- **Type status:** Other material. **Occurrence:** recordedBy: J.Penttinen; individualCount: 1; sex: male; **Location:** country: Finland; stateProvince: Regio aboënsis; municipality: Salo; locality: Märy; decimalLatitude: 60.223; decimalLongitude: 22.905; geodeticDatum: WGS84; **Identification:** identifiedBy: J.Penttinen; **Event:** samplingProtocol: Malaise trap; eventDate: 2009-8-15/9-15; habitat: old managed forest, herb-rich type; **Record Level:** institutionCode: JPJ

#### Distribution

Palaearctic. Described from Western Siberia (Ostroverkhova 1979) and later recorded only from southern Sweden (Kurina et al. 2004, Kjaerandsen et al. 2007). A record from Germany (Plassmann and Schacht 2002) is incorrect (Kjaerandsen et al. 2007). Here reported formally for the first time from Finland; the records presented here are from the hemiboreal, south boreal, north boreal and subarctic zones.

#### Ecology

In Finland collected from swamps, herb-rich forests and from a subarctic mountain birch forest. Immature stages are unknown.

#### Conservation

Included in the Finnish Red List (DD, Penttinen et al. 2010), but reported here formally as a new species for Finland.

### Brevicornu glandis

Lastovka & Matile, 1974*

http://www.faunaeur.org/full_results.php?id=139946

#### Materials

- **Type status:** Other material. **Occurrence:** catalogNumber: MYCE-JS-2013-0328; recordedBy: J. Ilmonen; individualCount: 1; sex: male; **Location:** country: Finland; stateProvince: Nylandia; municipality: Espoo; locality: Matalajärvi; decimalLatitude: 60.247; decimalLongitude: 24.687; geodeticDatum: WGS84; **Identification:** identifiedBy: J. Salmela; **Event:** samplingProtocol: Malaise trap; eventDate: 2012-7-21/8-23; habitat: swampy lake shore; **Record Level:** institutionCode: JES

#### Distribution

Palaearctic. Described from Mongolia (Laštovka and Matile 1974) and later found from Europe, including Germany (Caspers 1987), British Isles (Chandler 2001), Czech Republic, France (Chandler 2004) and Sweden (Kjaerandsen 2012). New for Finland.

#### Ecology

Immature stages are unknown. The Finnish collecting site is a swampy shore of a shallow, eutrophic lake.

### Brevicornu rosmellitum

Chandler, 2001*

http://www.faunaeur.org/full_results.php?id=139967

#### Materials

- **Type status:** Other material. **Occurrence:** catalogNumber: DIPT-JS-2014-0038; recordedBy: J. Salmela; T. Hietajärvi; individualCount: 1; sex: male; **Location:** country: Finland; stateProvince: Regio kuusamoensis; verbatimLocality: Salla, Kuntasjoki, Värriö Strict Nature Reserve; verbatimElevation: 320 m; decimalLatitude: 67.749; decimalLongitude: 29.617; geodeticDatum: WGS84; **Identification:** identifiedBy: J. Salmela; **Event:** samplingProtocol: Malaise trap; verbatimEventDate: 2013-6-29/7-29; habitat: headwater stream, old-growth boreal forest; **Record Level:** institutionCode: JES

#### Distribution

Holarctic. The species was described from England and Nearctic non-type material studied by Zaitzev (Zaitzev 1988, as *Brevicornu nigrofuscum*, from USA and Canada) actually represents *Brevicornu rosmellitum* (Chandler 2001). New for Finland.

#### Ecology

Immature stages are unknown. The British type specimens, five males, were taken from honey dew (Chandler 2001). The Finnish collecting site is a headwater stream surrounded by old-growth boreal forest.

### Brevicornu setulosum

Zaitzev, 1988

http://www.faunaeur.org/full_results.php?id=139977

#### Materials

- **Type status:** Other material. **Occurrence:** catalogNumber: MYCE-JS-2013-0027; recordedBy: J. Salmela; individualCount: 2; sex: male; **Location:** country: Finland; stateProvince: Lapponia kemensis pars orientalis; verbatimLocality: Savukoski, Joutenoja; decimalLatitude: 67.821; decimalLongitude: 29.440; geodeticDatum: WGS84; **Identification:** identifiedBy: J. Salmela; **Event:** samplingProtocol: Malaise trap; eventDate: 2012-8-16/9-18; habitat: headwater stream, seminatural boreal forest; **Record Level:** institutionCode: JES
- **Type status:** Other material. **Occurrence:** catalogNumber: MYCE-JS-2013-0087; recordedBy: J. Salmela; individualCount: 3; sex: male; **Location:** country: Finland; stateProvince: Lapponia kemensis pars orientalis; verbatimLocality: Savukoski, Joutenoja; decimalLatitude: 67.821; decimalLongitude: 29.440; geodeticDatum: WGS84; **Identification:** identifiedBy: J. Salmela; **Event:** samplingProtocol: Malaise trap; eventDate: 2012-7-10/8-16; habitat: headwater stream, seminatural boreal forest; **Record Level:** institutionCode: JES
- **Type status:** Other material. **Occurrence:** catalogNumber: MYCE-NV-2013-0033; recordedBy: J. Salmela; individualCount: 1; sex: male; **Location:** country: Finland; stateProvince: Lapponia kemensis pars occidentalis; verbatimLocality: Kittilä, Taljavaaranvuoma; decimalLatitude: 67.578; decimalLongitude: 25.358; geodeticDatum: WGS84; **Identification:** identifiedBy: N. Vartija; **Event:** samplingProtocol: Malaise trap; eventDate: 2007-6-25/7-24; habitat: Rich fen; **Record Level:** institutionCode: JES
- **Type status:** Other material. **Occurrence:** recordedBy: J. Salmela; individualCount: 1; sex: male; **Location:** country: Finland; stateProvince: Lapponia kemensis pars occidentalis; verbatimLocality: Kittilä, Silmäsvuoma; decimalLatitude: 67.582; decimalLongitude: 25.543; geodeticDatum: WGS84; **Identification:** identifiedBy: N. Vartija; **Event:** samplingProtocol: Malaise trap; eventDate: 2007-5-31/6-25; habitat: Rich fen
- **Type status:** Other material. **Occurrence:** catalogNumber: MYCE-NV-2013-0082; recordedBy: J. Salmela; individualCount: 1; sex: male; **Location:** country: Finland; stateProvince: Lapponia kemensis pars occidentalis; verbatimLocality: Kittilä, Kielisenpalo; decimalLatitude: 68.020; decimalLongitude: 25.063; geodeticDatum: WGS84; **Identification:** identifiedBy: N. Vartija; **Event:** samplingProtocol: Malaise trap; eventDate: 2007-8-1/31; habitat: Rich spring fen; **Record Level:** institutionCode: JES
- **Type status:** Other material. **Occurrence:** recordedBy: J. Salmela; individualCount: 1; sex: male; **Location:** country: Finland; stateProvince: Lapponia kemensis pars orientalis; verbatimLocality: Sodankylä, Ylä-Postojoki; decimalLatitude: 67.851; decimalLongitude: 26.481; geodeticDatum: WGS84; **Identification:** identifiedBy: N. Vartija; **Event:** samplingProtocol: Malaise trap; eventDate: 2009-6-1/29; habitat: riparian forest
- **Type status:** Other material. **Occurrence:** recordedBy: J. Salmela; individualCount: 1; sex: male; **Location:** country: Finland; stateProvince: Lapponia kemensis pars occidentalis; verbatimLocality: Kittilä, Vielmakoskenpalo; decimalLatitude: 68.008; decimalLongitude: 25.046; geodeticDatum: WGS84; **Identification:** identifiedBy: N. Vartija; **Event:** samplingProtocol: Malaise trap; eventDate: 2007-8-1/31; habitat: Rich fen

#### Distribution

Holarctic. Known from eastern Siberia (Lena River, holotype), USA (Coeur d'Alene, paratype) (Zaitzev 1988), NW Russia (Polevoi 2000, Polevoi 2010), Finland (Polevoi 2001b) and Sweden (north, Kjaerandsen et al. 2007). In Finland hitherto collected only from a locality in the eastern part of the country (Ilomantsi). Here reported from Finnish Lapland, north boreal zone.

#### Ecology

Finnish collecting sites are an abandoned field (Polevoi 2001b), riparian forests and rich fens. Immature stages are unknown.

### Brevicornu spathulatum

(Lundström, 1911)*

http://www.faunaeur.org/full_results.php?id=139978

#### Materials

- **Type status:** Other material. **Occurrence:** recordedBy: J.Penttinen; individualCount: 1; sex: male; **Location:** country: Finland; stateProvince: Regio aboënsis; municipality: Salo; locality: Roomunmäki; decimalLatitude: 60.324; decimalLongitude: 23.665; geodeticDatum: WGS84; **Identification:** identifiedBy: J.Penttinen; **Event:** samplingProtocol: Malaise; eventDate: 2009-8-15/9-15; habitat: seminatural herb-rich forest; **Record Level:** institutionCode: JPJ

#### Distribution

Palaearctic. Described from Hungary (as *Brachycampta spathulata*, Lundström 1911), later recorded from Bulgaria, Romania, Czech Republic (Chandler 2004) and from Altai Mountains, Western Siberia (Zaitzev 2003). In the Nordic Region recorded from Sweden (Kjaerandsen 2012). No former records from Finland.

#### Ecology

The Finnish sampling locality is a herb-rich forest. Immature stages are unknown.

### Brevicornu verralli

(Edwards, 1925)*

http://www.faunaeur.org/full_results.php?id=139980

#### Materials

- **Type status:** Other material. **Occurrence:** recordedBy: J.Jakovlev; individualCount: 1; sex: male; **Location:** stateProvince: Lapponia enontekiensis; municipality: Enontekiö; locality: Kilpisjärvi_Saana_North_4; decimalLatitude: 69.045; decimalLongitude: 20.808; geodeticDatum: WGS84; **Identification:** identifiedBy: J.Jakovlev; **Event:** samplingProtocol: Sweep netting; eventDate: 2006-6-21/6-21; habitat: subarctic mountain birch forest; **Record Level:** institutionCode: JJH
- **Type status:** Other material. **Occurrence:** recordedBy: J.Jakovlev; J.Penttinen; individualCount: 1; sex: male; **Location:** stateProvince: Lapponia kemensis pars occidentalis; municipality: Kittilä; locality: Pallas-Yllästunturi National Park; decimalLatitude: 68.024; decimalLongitude: 24.150; geodeticDatum: WGS84; **Identification:** identifiedBy: J.Jakovlev; **Event:** samplingProtocol: Malaise trap; eventDate: 2006-7-15/8-14; habitat: old-growth forest, Myrtillus type
- **Type status:** Other material. **Occurrence:** recordedBy: J.Penttinen; individualCount: 1; sex: male; **Location:** stateProvince: Savonia australis; municipality: Rantasalmi; locality: Linnansaari; decimalLatitude: 62.116; decimalLongitude: 28.477; geodeticDatum: WGS84; **Identification:** identifiedBy: J.Penttinen; **Event:** samplingProtocol: Malaise trap; eventDate: 2008-6-24/7-25; habitat: old-growth forest, herb-rich type; **Record Level:** institutionCode: JPJ

#### Distribution

Palaearctic. Widely distributed in Europe (Chandler 2004), also in North Europe: Iceland, Norway, Sweden, Russian Karelia and Murmansk region (Kjaerandsen 2012). No former records from Finland.

#### Ecology

Immature stages are unknown. Finnish collecting sites are a mountain birch forest, an old-growth boreal forest and a herb-rich forest.

### Exechia borealis

Lundström, 1912

#### Materials

- **Type status:** Other material. **Occurrence:** recordedBy: R.Frey; individualCount: 1; sex: male; **Location:** stateProvince: Lapponia enontekiensis; municipality: Enontekiö; locality: Palojoensuu; decimalLatitude: 68.285; decimalLongitude: 23.095; geodeticDatum: WGS84; **Identification:** identifiedBy: C.Lundström; **Event:** eventDate: 1911-7-20/7-20; habitat: subarctic; **Record Level:** institutionCode: MZHF
- **Type status:** Other material. **Occurrence:** recordedBy: R.Frey; individualCount: 2; sex: 1 male, 1 female; **Location:** stateProvince: Lapponia kemensis pars occidentalis; municipality: Muonio; locality: Olostunturi; decimalLatitude: 67.923; decimalLongitude: 23.801; geodeticDatum: WGS84; **Identification:** identifiedBy: C.Lundström; **Event:** eventDate: 1911-8-1; **Record Level:** institutionCode: MZHF
- **Type status:** Other material. **Occurrence:** recordedBy: R.Frey; individualCount: 2; sex: male; **Location:** country: Finland; stateProvince: Lapponia kemensis pars occidentalis; municipality: Muonio; locality: Pallastunturi; decimalLatitude: 68.072; decimalLongitude: 24.068; geodeticDatum: WGS84; **Identification:** identifiedBy: C.Lundström; **Event:** eventDate: 1911-6-22; **Record Level:** institutionCode: MZHF
- **Type status:** Other material. **Occurrence:** recordedBy: J.Jakovlev; J.Penttinen; individualCount: 1; sex: male; **Location:** stateProvince: Lapponia enontekiensis; municipality: Enontekiö; locality: Kilpisjärvi_Saana_North_4; decimalLatitude: 69.045; decimalLongitude: 20.808; geodeticDatum: WGS84; **Identification:** identifiedBy: J.Jakovlev; **Event:** samplingProtocol: Malaise trap; eventDate: 2006-7-15/8-1; habitat: subarctic; **Record Level:** institutionCode: JJH

#### Distribution

European. This species was recently reinstated as separate from *Exechia spinuligera* Lundström, 1912 (lectotype from Enontekiö, Palojoki, Kjaerandsen et al. 2007a); before that it has been overlooked and confused with *Exechia frigida* (Boheman) in Europe (Chandler and Perry 2010). In the Nordic Region known from Iceland, Norway, Sweden and Finland (Kjaerandsen et al. 2007a).

#### Ecology

Immature stages are unknown.

### Exechia micans

Lastovka & Matile, 1974*

http://www.faunaeur.org/full_results.php?id=139867

#### Materials

- **Type status:** Other material. **Occurrence:** recordedBy: K.Mikkola; individualCount: 1; sex: male; **Location:** country: Finland; stateProvince: Lapponia enontekiensis; municipality: Enontekiö; locality: Siilastupa; decimalLatitude: 69.047; decimalLongitude: 20.900; geodeticDatum: WGS84; **Identification:** identifiedBy: J.Kjaerandsen; **Event:** samplingProtocol: Sweep netting; eventDate: 1963-6-23/6-23; habitat: subarctic; **Record Level:** institutionCode: MZHF

#### Distribution

Palaearctic. Described from Mongolia (Laštovka and Matile 1974), later recorded only from Germany (Chandler 2004), Russian Karelia (Zaitzev 2003), Murmansk region (Polevoi 2010), Iceland (Kjaerandsen et al. 2007a), Norway (Søli and Kjaerandsen 2008) and Sweden (Kjaerandsen et al. 2007). No former records from Finland.

#### Ecology

Immature stages are unknown. Mainly collected from northern areas. This species was recorded as widespread and common in Iceland (Kjaerandsen et al. 2007a), and occurs also in Greenland (G. Varkonyi, pers.comm.).

### Exechia styriaca

Strobl, 1898**

http://www.faunaeur.org/full_results.php?id=139893

#### Materials

- **Type status:** Other material. **Occurrence:** recordedBy: A. Polevoi; individualCount: 1; sex: male; **Location:** country: Russia; stateProvince: Leningrad province; verbatimLocality: Voznesenje, Gimreka; decimalLatitude: 61.153; decimalLongitude: 35.618; geodeticDatum: WGS84; **Identification:** identifiedBy: A. Polevoi; **Event:** samplingProtocol: Sweep netting; eventDate: 2007-6-2; **Record Level:** institutionCode: FRIP

#### Distribution

Palaearctic. Scattered records from Europe and Altai (Chandler 2004, Zaitzev 2003). In Fennoscandia known from Finland (Hackman 1980), Norway (Søli and Kjaerandsen 2008) and Sweden (Kjaerandsen et al. 2007). New to the Republic of Karelia.

#### Ecology

Immature stages are unknown. The Karelian specimen was collected in a herb-richbirch dominated forest.

### Exechiopsis (Exechiopsis) distendens

(Lackschewitz, 1937)

http://www.faunaeur.org/full_results.php?id=139780

#### Materials

- **Type status:** Other material. **Occurrence:** recordedBy: A. Polevoi; individualCount: 1; sex: male; **Location:** country: Russia; stateProvince: Leningrad province; verbatimLocality: Voznesenje, 1 km E of Gimreka; decimalLatitude: 61.151; decimalLongitude: 35.64; geodeticDatum: WGS84; **Identification:** identifiedBy: A. Polevoi; **Event:** samplingProtocol: Malaise trap; eventDate: 2008-8-27/10-1; **Record Level:** institutionCode: FRIP
- **Type status:** Other material. **Occurrence:** catalogNumber: MYCE-JS-2013-0323; recordedBy: J. Ilmonen; individualCount: 1; sex: male; **Location:** country: Finland; stateProvince: Nylandia; municipality: Espoo; locality: Matalajärvi; decimalLatitude: 60.247; decimalLongitude: 24.687; geodeticDatum: WGS84; **Identification:** identifiedBy: J. Salmela; **Event:** samplingProtocol: Malaise trap; eventDate: 2012-7-21/8-23; habitat: swampy lake shore; **Record Level:** institutionCode: JES
- **Type status:** Other material. **Occurrence:** recordedBy: W.Hackman; individualCount: 1; sex: male; **Location:** country: Finland; stateProvince: Nylandia; municipality: Espoo; locality: Westend; decimalLatitude: 60.159; decimalLongitude: 24.799; geodeticDatum: WGS84; **Identification:** identifiedBy: J.Kjaerandsen; **Event:** eventDate: 1962; **Record Level:** institutionCode: MZHF
- **Type status:** Other material. **Occurrence:** recordedBy: R.Tuomikoski; individualCount: 1; sex: male; **Location:** country: Finland; stateProvince: Nylandia; municipality: Helsinki; locality: Vuosaari; decimalLatitude: 60.218; decimalLongitude: 25.157; geodeticDatum: WGS84; **Identification:** identifiedBy: J.Kjaerandsen; **Event:** samplingProtocol: Sweep netting; eventDate: 1962; habitat: old-growth forest, Myrtillus type; **Record Level:** institutionCode: MZHF
- **Type status:** Other material. **Occurrence:** recordedBy: R.Tuomikoski; individualCount: 1; sex: male; **Location:** country: Finland; stateProvince: Regio aboënsis; municipality: Vihti; locality: Vihtijärvi; decimalLatitude: 60.523; decimalLongitude: 24.556; geodeticDatum: WGS84; **Identification:** identifiedBy: J.Kjaerandsen; **Event:** samplingProtocol: Sweep netting; eventDate: 1962-5-19/5-19; **Record Level:** institutionCode: MZHF
- **Type status:** Other material. **Occurrence:** recordedBy: R.Tuomikoski; individualCount: 1; sex: male; **Location:** country: Finland; stateProvince: Karelia australis; municipality: Punkasalmi; locality: Punkasalmi; decimalLatitude: 61.763; decimalLongitude: 29.402; geodeticDatum: WGS84; **Identification:** identifiedBy: J.Kjaerandsen; **Event:** samplingProtocol: Sweep netting; eventDate: 1963; **Record Level:** institutionCode: MZHF
- **Type status:** Other material. **Occurrence:** recordedBy: R.Tuomikoski; individualCount: 1; sex: male; **Location:** country: Finland; stateProvince: Regio kuusamoënsis; municipality: Kuusamo; locality: Iivaara; decimalLatitude: 65.797; decimalLongitude: 29.684; geodeticDatum: WGS84; **Identification:** identifiedBy: J.Kjaerandsen; **Event:** samplingProtocol: Sweep netting; eventDate: 1964-6-23/6-23; habitat: old-growth forest, Myrtillus type; **Record Level:** institutionCode: MZHF
- **Type status:** Other material. **Occurrence:** recordedBy: R.Tuomikoski and K.Mikkola; individualCount: 1; sex: male; **Location:** country: Finland; stateProvince: Regio kuusamoënsis; municipality: Kuusamo; locality: Jäkälävuoma; decimalLatitude: 66.258; decimalLongitude: 29.444; geodeticDatum: WGS84; **Identification:** identifiedBy: J.Kjaerandsen; **Event:** samplingProtocol: Sweep netting; eventDate: 1964-6-24/6-24; habitat: old-growth forest, Myrtillus type; **Record Level:** institutionCode: MZHF
- **Type status:** Other material. **Occurrence:** recordedBy: R.Tuomikoski; individualCount: 1; sex: male; **Location:** country: Finland; stateProvince: Karelia borealis; municipality: Pielisjärvi; locality: Koli_old; decimalLatitude: 63.120; decimalLongitude: 29.791; geodeticDatum: WGS84; **Identification:** identifiedBy: J.Kjaerandsen; **Event:** samplingProtocol: Sweep netting; eventDate: 1964; habitat: old-growth forest, Myrtillus type; **Record Level:** institutionCode: MZHF
- **Type status:** Other material. **Occurrence:** recordedBy: R.Tuomikoski; individualCount: 1; sex: male; **Location:** country: Finland; stateProvince: Karelia borealis; municipality: Tohmajärvi; locality: Hiidenvaara; decimalLatitude: 62.203; decimalLongitude: 30.629; geodeticDatum: WGS84; **Identification:** identifiedBy: J.Kjaerandsen; **Event:** samplingProtocol: Sweep netting; eventDate: 1965; habitat: old-growth forest, Myrtillus type; **Record Level:** institutionCode: MZHF
- **Type status:** Other material. **Occurrence:** recordedBy: T.Brander; individualCount: 1; sex: male; **Location:** country: Finland; stateProvince: Tavastia australis; municipality: Urjala; locality: Urjala; decimalLatitude: 60.180; decimalLongitude: 23.662; geodeticDatum: WGS84; **Identification:** identifiedBy: J.Kjaerandsen; **Event:** habitat: old-growth forest, Myrtillus type; **Record Level:** institutionCode: MZHF

#### Distribution

Palaearctic. Widely distributed (Chandler 2004, Zaitzev 2003). In Fennoscandia recorded from Finland (Hackman 1980), Norway (Gammelmo and Søli 2006) and Sweden (Kjaerandsen et al. 2007). In the Republic of Karelia it was only known from the Kivach Nature Reserve (Zaitzev 2003).

#### Ecology

Most of the specimens were collected in coniferous forests. Immature stages are unknown. The host range of *Exechiopsis* includes fruiting bodies of soft terrestrial fungi, and also some wood-encrusting fungi (Jakovlev 2011a).

#### Conservation

 Red-listed in Finland (NT, Penttinen et al. 2010).

### Exechiopsis (Exechiopsis) hammi

(Edwards, 1925)

http://www.faunaeur.org/full_results.php?id=139789

#### Materials

- **Type status:** Other material. **Occurrence:** catalogNumber: MYCE-JS-2013-0295; recordedBy: J. Ilmonen; individualCount: 1; sex: male; **Location:** country: Finland; stateProvince: Nylandia; municipality: Espoo; locality: Matalajärvi; decimalLatitude: 60.247; decimalLongitude: 24.687; geodeticDatum: WGS84; **Identification:** identifiedBy: J. Salmela; **Event:** samplingProtocol: Malaise trap; eventDate: 2012-8-23/10-20; habitat: swampy lake shore; **Record Level:** institutionCode: JES

#### Distribution

Palaearctic, rather widely distributed in Europe (Chandler 2004). In Fennoscandia recorded from Sweden, Norway, Finland and Russian Karelia (Hackman 1980, Polevoi 2000, Zaitzev 2003, Kjaerandsen et al. 2007). In Finland only known from the southern parts of the country.

#### Ecology

Immature stages are unknown, hibernating adults have been observed in caves in Norway (Kjaerandsen 1993). In Finland collected from old-growth forests, burnt forests, herb-rich forests and from a swampy lake shore (Matalajärvi).

#### Conservation

Red-listed (NT) in Finland (Penttinen et al. 2010).

### Exechiopsis (Xenexechia) davatchii

Matile, 1969**

http://www.faunaeur.org/full_results.php?id=139817

#### Materials

- **Type status:** Other material. **Occurrence:** recordedBy: A. Polevoi; individualCount: 1; sex: male; **Location:** country: Russia; stateProvince: Republic Karelia; verbatimLocality: Shun'ga, Turastamozero; decimalLatitude: 62.56; decimalLongitude: 34.706; geodeticDatum: WGS84; **Identification:** identifiedBy: A. Polevoi; **Event:** samplingProtocol: Malaise trap; eventDate: 2012-7-21/8-24; **Record Level:** institutionCode: FRIP

#### Distribution

Palaearctic. Widespread in western Europe (Chandler 2004) and has recently been recorded from Britain (Chandler and Perry 2010). Scattered records from the Near East and East Russia (Zaitzev 2003, Chandler 2004, Kurina and Ševčík 2006). In Fennoscandia recorded from South Finland (Jakovlev et al. 2006) and Sweden (Kjaerandsen et al. 2007). New to the Republic of Karelia.

#### Ecology

The Karelian specimen was collected in *Vaccinium myrtillus* type pine dominated forest.

### Pseudexechia parallela

(Edwards, 1925)*

http://www.faunaeur.org/full_results.php?id=139762

#### Materials

- **Type status:** Other material. **Occurrence:** catalogNumber: MYCE-JS-2013-0315; recordedBy: J. Ilmonen; individualCount: 1; sex: male; **Location:** country: Finland; stateProvince: Nylandia; municipality: Espoo; locality: Matalajärvi; decimalLatitude: 60.247; decimalLongitude: 24.687; geodeticDatum: WGS84; **Identification:** identifiedBy: J. Salmela; **Event:** samplingProtocol: Malaise trap; eventDate: 2012-8-23/10-20; habitat: swampy lake shore; **Record Level:** institutionCode: JES

#### Distribution

Holarctic. Widely distributed in Europe, in Fennoscandia known from Sweden (Kjaerandsen 2009). The specimen recorded from Russian Karelia (Polevoi 2000) in fact belongs to *Pseudexechia pectinacea* Ostroverkhova. Most likely the species is quite rare in Northern Europe, its range seems to be confined to nemoral - hemiboreal vegetation zones (cf. Kjaerandsen 2009). New for Finland, here reported from the hemiboreal vegetation zone.

#### Ecology

Immature stages are unknown, but several British collecting localities are wetlands (Kjaerandsen 2009). In Finland the species was trapped from a swampy lake shore (Matalajärvi).

### Rymosia pinnata

Ostroverkhova, 1979**

http://www.faunaeur.org/full_results.php?id=139727

#### Materials

- **Type status:** Other material. **Occurrence:** recordedBy: A. Polevoi; individualCount: 1; sex: male; **Location:** country: Russia; stateProvince: Republic Karelia; verbatimLocality: Ladvozero, Haapavaara; decimalLatitude: 64.853; decimalLongitude: 29.897; geodeticDatum: WGS84; **Identification:** identifiedBy: A. Polevoi; **Event:** samplingProtocol: Sweep netting; eventDate: 2012-9-13; **Record Level:** institutionCode: FRIP
- **Type status:** Other material. **Occurrence:** recordedBy: A. Polevoi; individualCount: 1; sex: male; **Location:** country: Russia; stateProvince: Murmansk province; verbatimLocality: Kutsa, near lake Pyhäjarvi; decimalLatitude: 66.714; decimalLongitude: 29.968; geodeticDatum: WGS84; **Identification:** identifiedBy: A. Polevoi; **Event:** samplingProtocol: Sweep netting; eventDate: 2010-6-2; **Record Level:** institutionCode: FRIP

#### Distribution

Palaearctic. Described from West Siberia and Russian Far East (Ostroverkhova 1979). In Europe was known only from Finland (Polevoi et al. 2006) and Sweden (Kjaerandsen et al. 2007). New to the Republic of Karelia and Murmansk Province.

#### Ecology

Immature stages are unknown. All existing rearing records of *Rymosia* species are from fruiting bodies of soft macrofungi (Jakovlev 1994, Ševčík 2010). Specimens from Karelia and Murmansk Province were collected in *Vaccinium myrtillus* type coniferous forests.

### Synplasta bayardi

Matile, 1971*

http://www.faunaeur.org/full_results.php?id=139672

#### Materials

- **Type status:** Other material. **Occurrence:** recordedBy: J.Jakovlev; individualCount: 1; sex: male; **Location:** country: Finland; stateProvince: Tavastia australis; municipality: Luopioinen; locality: Kuohijoen kalkkilehto; decimalLatitude: 61.307; decimalLongitude: 24.874; geodeticDatum: WGS84; **Identification:** identifiedBy: J.Jakovlev; **Event:** samplingProtocol: Malaise trap; eventDate: 2007-4-29/6-10; habitat: old-growth forest, herb-rich type; **Record Level:** institutionCode: JJH

#### Distribution

European. Very rare species, described from France (as *Allodiopsis bayardi*, Matile 1971) and found later from Central Europe (Chandler 2004), Russian Karelia (Polevoi 2000) and Sweden (Kjaerandsen et al. 2007). Included in the Red List of Finnish species, here reported formally for the first time from Finland.

#### Ecology

Immature stages are unknown. Based on the ecology of related genera, the larvae of *Synplasta* probably develop in soft macrofungi. The Finnish collecting site is a calcareous herb-rich forest.

#### Conservation

Red-listed in Finland (VU, Penttinen et al. 2010).

### Synplasta pseudingeniosa

Zaitzev, 1993*

http://www.faunaeur.org/full_results.php?id=139685

#### Materials

- **Type status:** Other material. **Occurrence:** recordedBy: M.Jaschhof and C.Jaschhof; individualCount: 1; sex: male; **Location:** country: Finland; stateProvince: Regio kuusamoënsis; municipality: Kuusamo; locality: Kuohusuo-Kalliovaara; decimalLatitude: 65.674; decimalLongitude: 28.888; geodeticDatum: WGS84; **Identification:** identifiedBy: J.Jakovlev; **Event:** samplingProtocol: Sweep netting; eventDate: 2004-7-30/7-30; habitat: old managed forest, Myrtillus type; **Record Level:** institutionCode: JJH
- **Type status:** Other material. **Occurrence:** recordedBy: A. Polevoi; individualCount: 1; sex: male; **Location:** country: Russia; stateProvince: Republic Karelia; verbatimLocality: Tambitsy, Tolstyi Navolok; decimalLatitude: 62.291; decimalLongitude: 35.57; geodeticDatum: WGS84; **Identification:** identifiedBy: A. Polevoi; **Event:** samplingProtocol: Sweep netting; eventDate: 2013-8-26; **Record Level:** institutionCode: FRIP

#### Distribution

European. Recorded so far only from NW Russia (Zaitzev 1993, Polevoi 2000), Norway (Søli and Kjaerandsen 2008), Sweden (Kjaerandsen et al. 2007), Estonia (Chandler 2004) and Slovakia (Ševčík and Kurina 2011).

#### Ecology

Immature stages are unknown.

### Dynatosoma cochleare

Strobl, 1895**

http://www.faunaeur.org/full_results.php?id=140556

#### Materials

- **Type status:** Other material. **Occurrence:** recordedBy: A. Polevoi; individualCount: 1; sex: male; **Location:** country: Russia; stateProvince: Republic Karelia; verbatimLocality: Valaam, Divnaya Bukhta; decimalLatitude: 61.35; decimalLongitude: 30.99; geodeticDatum: WGS84; **Identification:** identifiedBy: A. Polevoi; **Event:** samplingProtocol: Malaise trap; eventDate: 2009-7-27/8-2; **Record Level:** institutionCode: FRIP

#### Distribution

Palaearctic. Widely distributed in Europe (Chandler 2004, Ševčík and Kurina 2011) and also recorded from the Russian Far East, Kuril Islands (Zaitzev 2003). In Fennoscandia reported from Finland (Hackman 1980), Norway (Gammelmo and Søli 2006) and Sweden (Kjaerandsen et al. 2007). New to the Republic of Karelia.

#### Ecology

The Karelian specimen was collected in herb-rich spruce dominated forest. The larvae of *Dynatosoma* usually live within fruiting bodies of soft polypores (Ševčík 2010, Jakovlev 2012). Their presence may often be detected by white frass that the larvae extrude from their burrows onto the surface of the fungus (Edwards 1925).

### Dynatosoma majus

Landrock, 1912

http://www.faunaeur.org/full_results.php?id=140563

#### Materials

- **Type status:** Other material. **Occurrence:** recordedBy: J. Jakovlev; individualCount: 1; sex: male; **Location:** country: Finland; stateProvince: Tavastia australis; verbatimLocality: Padasjoki, Vesijako Strict Nature Reserve; decimalLatitude: 61.350; decimalLongitude: 25.105; geodeticDatum: WGS84; **Identification:** identifiedBy: J. Jakovlev; **Event:** samplingProtocol: Malaise trap; eventDate: 2004-4-28/5-27; habitat: old-growth forest, Myrtillus type; **Record Level:** institutionCode: JJH
- **Type status:** Other material. **Occurrence:** recordedBy: J. Jakovlev; individualCount: 1; sex: male; **Location:** country: Finland; stateProvince: Tavastia australis; verbatimLocality: Lammi, Evo, Puukkohonka; decimalLatitude: 61.222; decimalLongitude: 25.052; geodeticDatum: WGS84; **Identification:** identifiedBy: J. Jakovlev; **Event:** samplingProtocol: Malaise trap; eventDate: 2004-6-29/7-27; habitat: old-growth forest, Myrtillus type; **Record Level:** institutionCode: JJH
- **Type status:** Other material. **Occurrence:** catalogNumber: MYCE-JS-2013-0395; recordedBy: J. Salmela; S. Lapinniemi; individualCount: 1; sex: male; **Location:** country: Finland; stateProvince: Lapponia enontekiensis; verbatimLocality: Pallas-Yllästunturi National Park, Röyninkuru; verbatimElevation: 360 m; decimalLatitude: 68.; decimalLongitude: 24.; geodeticDatum: WGS84; **Identification:** identifiedBy: J. Salmela; **Event:** samplingProtocol: Malaise trap; eventDate: 2013-7-5/8-7; habitat: headwater stream, old-growth spruce forest; **Record Level:** institutionCode: JES
- **Type status:** Other material. **Occurrence:** recordedBy: M. Kuussaari; individualCount: 1; sex: male; **Location:** country: Finland; stateProvince: Tavastia australis; verbatimLocality: Kuru, Petäjäjärvi; decimalLatitude: 62.077; decimalLongitude: 23.051; geodeticDatum: WGS84; **Identification:** identifiedBy: A. Polevoi; J. Jakovlev; **Event:** samplingProtocol: Bait trap; eventDate: 1998-8-24/9-17; habitat: old-growth forest, Myrtillus type; **Record Level:** institutionCode: FRIP
- **Type status:** Other material. **Occurrence:** recordedBy: M. Kuussaari; individualCount: 1; sex: male; **Location:** country: Finland; stateProvince: Tavastia australis; verbatimLocality: Ruovesi, Susimäki; decimalLatitude: 61.857; decimalLongitude: 24.237; geodeticDatum: WGS84; **Identification:** identifiedBy: A. Polevoi; J. Jakovlev; **Event:** samplingProtocol: Window trap; eventDate: 1998-7-17/8-12; habitat: old-growth forest, Myrtillus type
- **Type status:** Other material. **Occurrence:** recordedBy: M. Kuussaari; individualCount: 1; sex: male; **Location:** country: Finland; stateProvince: Satakunta; verbatimLocality: Parkano, Nälkähittenkangas; decimalLatitude: 62.083; decimalLongitude: 23.090; geodeticDatum: WGS84; **Identification:** identifiedBy: A. Polevoi; J. Jakovlev; **Event:** samplingProtocol: Bait trap; eventDate: 1998-6-15/29; habitat: old-growth forest, Myrtillus type

#### Distribution

Palaearctic. The species is known from Central and eastern Europe (Chandler 2004), Sibera and Russian Far East (Zaitzev 2003). In Fennoscandia only recorded from Sweden (hemiboreal zone, Kjaerandsen et al. 2007) and Finland. The majority of the Finnish records, five out of six, are from the south boreal zone. One of the records is from NW Lapland, north boreal zone.

#### Ecology

Finnish records are invariably from *Vaccinium myrtillus* type old-growth boreal forests. Immature stages are unknown, but are likely associated with polyporous fungi (see *Dynatosoma cochleare*).

#### Conservation

Red-listed in Finland (NT, Penttinen et al. 2010).

### Dynatosoma rufescens

(Zetterstedt, 1838)

http://www.faunaeur.org/full_results.php?id=140571

#### Materials

- **Type status:** Other material. **Occurrence:** recordedBy: A. Polevoi; individualCount: 3; sex: male; **Location:** country: Russia; stateProvince: Republic Karelia; verbatimLocality: Valday, lake Ladozero; decimalLatitude: 63.588; decimalLongitude: 35.844; geodeticDatum: WGS84; **Identification:** identifiedBy: A. Polevoi; **Event:** samplingProtocol: Malaise trap; eventDate: 2010-6-27/8-13; **Record Level:** institutionCode: FRIP

#### Distribution

European. Known from central and northern Europe (Chandler 2004). In Fennoscandia reported from Finland (Hackman 1980), Norway (Gammelmo and Søli 2006) and Sweden (Kjaerandsen et al. 2007). Earlier records from the Republic of Karelia (Polevoi 2000) are erroneous and refer to *Dynatosoma silesiacum* Ševčík.

#### Ecology

Karelian specimens were collected in *Vaccinium myrtillus* type spruce dominated forest. This species was reared from *Laetiphorus sulphureus* in Germany (Kallweit 1990).

### Dynatosoma silesiacum

Ševčík, 2001**

http://www.faunaeur.org/full_results.php?id=140575

#### Materials

- **Type status:** Other material. **Occurrence:** recordedBy: A. Polevoi; individualCount: 2; sex: male; **Location:** country: Russia; stateProvince: Republic Karelia; verbatimLocality: Kartesh; decimalLatitude: 66.337; decimalLongitude: 33.649; geodeticDatum: WGS84; **Identification:** identifiedBy: A. Polevoi; **Event:** samplingProtocol: Malaise trap; eventDate: 1996-7-22/24; **Record Level:** institutionCode: FRIP
- **Type status:** Other material. **Occurrence:** recordedBy: M. Tietäväinen et al.; individualCount: 2; sex: male; **Location:** country: Russia; stateProvince: Republic Karelia; verbatimLocality: 6 km N of Tolvojarvi; decimalLatitude: 62.317; decimalLongitude: 31.435; geodeticDatum: WGS84; **Identification:** identifiedBy: A. Polevoi; **Event:** samplingProtocol: Malaise trap; eventDate: 1998-7-25/8-14; **Record Level:** institutionCode: FRIP
- **Type status:** Other material. **Occurrence:** recordedBy: M. Tietäväinen et al.; individualCount: 8; sex: male; **Location:** country: Russia; stateProvince: Republic Karelia; verbatimLocality: 5 km N of Tolvojarvi; decimalLatitude: 62.317; decimalLongitude: 31.435; geodeticDatum: WGS84; **Identification:** identifiedBy: A. Polevoi; **Event:** samplingProtocol: Malaise trap; eventDate: 1999-6-2/9-7; **Record Level:** institutionCode: FRIP

#### Distribution

Described from Czech Republic (Ševčík 2001a), later reported from Finland (Jakovlev et al. 2006) and Sweden (Kjaerandsen et al. 2007). Specimens from Russian Karelia were previously misidentified as *Dynatosoma rufescens* (Polevoi 2000).

#### Ecology

Karelian specimens were collected in *Vaccinium myrtillus* type forests of different age and tree composition. Immature stages are unknown.

### Epicypta limnophila

Chandler, 1981*

http://www.faunaeur.org/full_results.php?id=140549

#### Materials

- **Type status:** Other material. **Occurrence:** recordedBy: J.Jakovlev; J.Penttinen; individualCount: 1; sex: male; **Taxon:** genus: Epicypta; specificEpithet: limnophila; scientificNameAuthorship: Chandler, 1981; **Location:** country: Finland; stateProvince: Nylandia; municipality: Espoo; locality: Nuuksio, Siikajärvi; decimalLatitude: 60.288; decimalLongitude: 24.541; geodeticDatum: WGS84; **Identification:** identifiedBy: J.Jakovlev; **Event:** samplingProtocol: Malaise trap; eventDate: 2005-5-13/6-13; habitat: old-growth forest, herb-rich type; **Record Level:** institutionCode: JJH
- **Type status:** Other material. **Occurrence:** catalogNumber: MYCE-JS-2013-0339; recordedBy: J. Ilmonen; individualCount: 2; sex: male; otherCatalogNumbers: MYCE-JS-2013-0343; **Taxon:** genus: Epicypta; specificEpithet: limnophila; scientificNameAuthorship: Chandler, 1981; **Location:** country: Finland; stateProvince: Nylandia; municipality: Espoo; locality: Matalajärvi; decimalLatitude: 60.247; decimalLongitude: 24.687; geodeticDatum: WGS84; **Identification:** identifiedBy: J. Salmela; **Event:** samplingProtocol: Malaise trap; eventDate: 2012-7-21/8-23; habitat: swampy lake shore; **Record Level:** institutionCode: JES

#### Distribution

Holarctic. *Epicypta limnophila* is known from USA, British Isles (Chandler 1981), Central Europe and Fennoscandia (Chandler 2004, Kjaerandsen 2012). In Fennoscandia recorded from Russian Karelia (Polevoi 2000, citing Zaitzev 1987), Norway (Anonymous 2010) and Sweden (Kjaerandsen 2012). New for Finland.

#### Ecology

In the British Isles the species is associated with wet woodlands and bogs, suggesting that it may develop on decaying herbaceous vegetation, rather than dead wood, unlike the related species, *Epicypta aterrima* (Zetterstedt) and *Epicypta scatophora* (Perris) (Chandler 1981). Finnish sampling sites are a herb-rich forest and a swampy lake shore.

#### Conservation

Red-listed in Norway (VU, Anonymous 2010, Gammelmo et al. 2010).

### Macrobrachius kowarzii

Dziedzicki, 1889**

http://www.faunaeur.org/full_results.php?id=140540

#### Materials

- **Type status:** Other material. **Occurrence:** recordedBy: A. Polevoi; individualCount: 1; sex: male; **Location:** country: Russia; stateProvince: Leningrad province; verbatimLocality: Voznesenje, 1 km SE of Gimreka; decimalLatitude: 61.151; decimalLongitude: 35.642; geodeticDatum: WGS84; **Identification:** identifiedBy: A. Polevoi; **Event:** samplingProtocol: Malaise trap; eventDate: 2008-4-23/5-25; **Record Level:** institutionCode: FRIP

#### Distribution

European. Rare species distributed in Central Europe (Chandler 2004, Ševčík and Kurina 2011). New to Russia and Fennoscandia.

#### Ecology

Collected in *Vaccinium myrtillus* type pine-dominated forest. Immature stages are unknown.

### Mycetophila biformis

Maximova, 2002***

#### Materials

- **Type status:** Other material. **Occurrence:** recordedBy: M. Tietäväinen et al.; individualCount: 1; sex: male; **Location:** country: Russia; stateProvince: Republic Karelia; verbatimLocality: 5 km N of Tolvojarvi; decimalLatitude: 62.318; decimalLongitude: 31.436; geodeticDatum: WGS84; **Identification:** identifiedBy: A. Polevoi; **Event:** samplingProtocol: Malaise trap; eventDate: 1999-6-22/30; **Record Level:** institutionCode: FRIP
- **Type status:** Other material. **Occurrence:** catalogNumber: MYCE-JS-2013-0138; recordedBy: J. Salmela; individualCount: 1; sex: male; **Location:** country: Finland; stateProvince: Lapponia kemensis pars orientalis; verbatimLocality: Savukoski, Törmäoja; decimalLatitude: 67.835; decimalLongitude: 29.454; geodeticDatum: WGS84; **Identification:** identifiedBy: A. Polevoi; **Event:** samplingProtocol: Malaise trap; eventDate: 2012-7-10/8-16; habitat: headwater stream, old-growth boreal forest; **Record Level:** institutionCode: JES

#### Distribution

Palaearctic. Described from West Siberia (Maximova 2002), no other previous records were known. New to Europe, Finland and the Republic of Karelia.

#### Ecology

The Karelian specimen was collected in *Vaccinium myrtillus* type sprucedominated forest. The Finnish sampling site is a headwater stream surrounded by old-growth boreal forest. Immature stages are unknown.

#### Taxon discussion

*Mycetophila biformis* Maximova, 2002 is a junior primary homonym of *Mycetophila biformis* Duret, 1992. A replacement name will be given by the author in the near future (Yu. Maximova, pers. comm.).

### Mycetophila boreocruciator

Ševčík, 2003

http://www.faunaeur.org/full_results.php?id=140347

#### Materials

- **Type status:** Other material. **Occurrence:** catalogNumber: DIPT-JS-2014-0009; recordedBy: J. Salmela; T. Hietajärvi; individualCount: 2; sex: male; **Location:** country: Finland; stateProvince: Regio kuusamoensis; verbatimLocality: Salla, Kuntasjoki, Värriö Strict Nature Reserve; verbatimElevation: 320 m; decimalLatitude: 67.749; decimalLongitude: 29.617; geodeticDatum: WGS84; **Identification:** identifiedBy: J. Salmela; **Event:** samplingProtocol: Malaise trap; eventDate: 2013; verbatimEventDate: 2013-6-4/29; habitat: headwater stream, old-growth boreal forest; **Record Level:** institutionCode: JES

#### Distribution

European. Description of the species was based on material collected from Sweden, Estonia and Slovakia (Ševčík 2003). Recently found from Russia, Murmansk region (Polevoi 2010) and northern Norway (Søli and Rindal 2012). The Swedish records range from the hemiboreal to the boreal zone (Kjaerandsen et al. 2007). New for Finland. Records of *Mycetophila paracruciator* Lastovka & Matile, 1974 from Switzerland, Italy and France may actually represent *Mycetophila boreocruciator* (Chandler 2004).

#### Ecology

Immature stages are unknown. The Finnish sampling site is a headwater stream valley surrounded by old-growth boreal forest.

### Mycetophila cingulum

Meigen, 1830*

http://www.faunaeur.org/full_results.php?id=140354

#### Materials

- **Type status:** Other material. **Occurrence:** recordedBy: J.Penttinen; individualCount: 1; sex: male; **Location:** country: Finland; stateProvince: Karelia ladogensis; municipality: Parikkala; locality: Siikalahti; decimalLatitude: 61.556; decimalLongitude: 29.558; geodeticDatum: WGS84; **Identification:** identifiedBy: J.Jakovlev; **Event:** samplingProtocol: Malaise trap; eventDate: 2008-7-22/9-1; habitat: old-growth forest, herb-rich type; **Record Level:** institutionCode: JPJ
- **Type status:** Other material. **Occurrence:** recordedBy: J.Jakovlev; individualCount: 1; sex: male; **Location:** country: Finland; stateProvince: Nylandia; municipality: Helsinki; locality: Tullisaari, Stansvikin kartano; decimalLatitude: 60.169; decimalLongitude: 25.024; geodeticDatum: WGS84; **Identification:** identifiedBy: J.Jakovlev; **Event:** samplingProtocol: Malaise trap; eventDate: 2011-8-2/8-13; habitat: Wood-storage areas in Helsinki; **Record Level:** institutionCode: JJH

#### Distribution

Holarctic. In Europe recorded mainly from western, northern and central Europe (Chandler 2004, Kjaerandsen 2012). In Fennoscandia there are few and scattered records from southern and northern parts of the region (Kjaerandsen et al. 2007, Søli and Kjaerandsen 2008). Here reported formally for the first time from Finland.

#### Ecology

Larvae are associated with fruiting bodies of a saproxylic bracket fungi: *Polyporus squamosus* (Jakovlev 1994, Zaitzev 2003, Ševčík 2010) and *Grifola frondosa* (Chandler 1993).

#### Conservation

Red-listed in Finland (VU, Penttinen et al. 2010). This species is common in the British Isles and western Europe, but in Finland was found only in two localities, a herb rich forest at Parikkala and a city park at Helsinki, both situated in southernmost part of the country. Its host fungus *Polyporus squamosus* is distributed chiefly in southern Finland, e.g. colonize elm, ash, maple trees in parks, but could also be found at moist places on willows in central Finland.

### Mycetophila confusa

Dziedzicki, 1884

http://www.faunaeur.org/full_results.php?id=140360

#### Materials

- **Type status:** Other material. **Occurrence:** recordedBy: J.Penttinen; individualCount: 1; sex: male; **Location:** country: Finland; stateProvince: Tavastia australis; municipality: Nastola; locality: Kurasto; decimalLatitude: 61.109; decimalLongitude: 24.262; geodeticDatum: WGS84; **Identification:** identifiedBy: J.Penttinen; **Event:** samplingProtocol: Malaise trap; eventDate: 2009-6-1/6-15; habitat: Semi-natural forest with lime; **Record Level:** institutionCode: JPJ

#### Distribution

Palaearctic, rather wide range in Europe (Chandler 2004). Listed from Finland as *Mycetophila affluctata* Edwards, 1941 (Hackman 1980) without locality data. Most likely a very rare species in Fennoscandia (cf. Kjaerandsen et al. 2007, Anonymous 2010).

#### Ecology

The Finnish collecting site is a herb-rich forest in the south boreal zone. Immature stages are unknown. Generally, *Mycetophila* species are associated as larvae with fruiting bodies of macrofungi, both terrestrial and wood-growing; a few species feed on slime moulds (Jakovlev 2011a).

#### Conservation

Red-listed in Norway (VU, Anonymous 2010, Gammelmo et al. 2010).

### Mycetophila devioides

Bechev, 1988*

http://www.faunaeur.org/full_results.php?id=140370

#### Materials

- **Type status:** Other material. **Occurrence:** recordedBy: M. Tietäväinen et al.; individualCount: 1; sex: male; **Location:** country: Finland; stateProvince: Karelia borealis; verbatimLocality: Ilomantsi, Kotavaara; decimalLatitude: 63.029; decimalLongitude: 31.377; geodeticDatum: WGS84; **Identification:** identifiedBy: A. Polevoi; **Event:** samplingProtocol: Malaise trap; eventDate: 1997-9-15/29; **Record Level:** institutionCode: FRIP

#### Distribution

European. The species was described from Bulgaria (Bechev 1988) and has been later recorded only from Slovakia (Chandler 2004) and Ukraine (Zaitzev 2003). No previous findings from the Nordic region, new for Finland.

#### Ecology

Collected in *Vaccinium myrtillus* type spruce dominated forest. Immature stages are unknown.

### Mycetophila distigma

Meigen, 1830*

http://www.faunaeur.org/full_results.php?id=140372

#### Materials

- **Type status:** Other material. **Occurrence:** recordedBy: J.Jakovlev; individualCount: 5; sex: 2 males, 3 females; **Location:** country: Finland; stateProvince: Nylandia; municipality: Helsinki; locality: Tuomarinkylä; decimalLatitude: 60.261; decimalLongitude: 24.965; geodeticDatum: WGS84; **Identification:** identifiedBy: J.Jakovlev; **Event:** samplingProtocol: Malaise trap; eventDate: 2011-6-5/7-4; habitat: Wood-storage areas in Helsinki; **Record Level:** institutionCode: JJH
- **Type status:** Other material. **Occurrence:** recordedBy: J.Jakovlev; individualCount: 1; sex: male; **Location:** country: Finland; stateProvince: Nylandia; municipality: Helsinki; locality: Tuomarinkylä; decimalLatitude: 60.261; decimalLongitude: 24.965; geodeticDatum: WGS84; **Identification:** identifiedBy: J.Jakovlev; **Event:** samplingProtocol: Malaise trap; eventDate: 2011-7-5/7-20; habitat: Wood-storage areas in Helsinki; **Record Level:** institutionCode: JJH

#### Distribution

**Figure 21.**
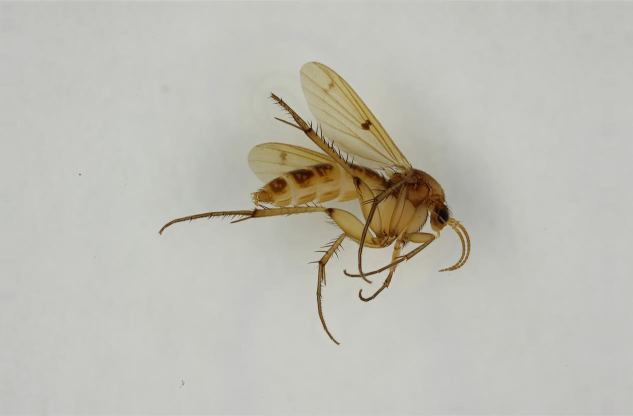
*Mycetophila distigma* Meigen, male specimen collected from Finland, lateral view.

European. *Mycetophila distigma* (Fig. 21) is recorded from central and northern Europe (Chandler 2004). In Fennoscandia known from Sweden (Lule Lapmark, Kjaerandsen et al. 2007) and Norway (Akershus, Soli et al. 2009). New for the Finnish fauna.

#### Ecology

Finnish specimens were collected in wood-storage areas in the city parks of Helsinki. Probably a saproxylic species, reared by Ševčík (Ševčík 2010) from the polypore fungus *Bjerkandera adusta*.

### Mycetophila forcipata

Lundström, 1913**

http://www.faunaeur.org/full_results.php?id=140386

#### Materials

- **Type status:** Other material. **Occurrence:** recordedBy: A. Polevoi; individualCount: 2; sex: male; **Location:** country: Russia; stateProvince: Leningrad province; verbatimLocality: Voznesenje, 1 km SE of Gimreka; decimalLatitude: 61.151; decimalLongitude: 35.642; geodeticDatum: WGS84; **Identification:** identifiedBy: A. Polevoi; **Event:** samplingProtocol: Malaise trap; eventDate: 2008-8-27/10-1; **Record Level:** institutionCode: FRIP
- **Type status:** Other material. **Occurrence:** recordedBy: M. Tietäväinen; individualCount: 1; sex: male; **Location:** country: Russia; stateProvince: Republic Karelia; verbatimLocality: 5 km N of Tolvojarvi; decimalLatitude: 62.318; decimalLongitude: 31.436; geodeticDatum: WGS84; **Identification:** identifiedBy: A. Polevoi; **Event:** samplingProtocol: Malaise trap; eventDate: 1999-6-11/22; **Record Level:** institutionCode: FRIP
- **Type status:** Other material. **Occurrence:** recordedBy: M. Tietäväinen; individualCount: 1; sex: male; **Location:** country: Russia; stateProvince: Republic Karelia; verbatimLocality: 5 km N of Tolvojarvi; decimalLatitude: 62.318; decimalLongitude: 31.436; geodeticDatum: WGS84; **Identification:** identifiedBy: A. Polevoi; **Event:** samplingProtocol: Malaise trap; eventDate: 1999-8-17/26; **Record Level:** institutionCode: FRIP

#### Distribution

Palaearctic. Widely distributed in Europe and East Palaearctic (Chandler 2004, Zaitzev 2003). In Fennoscandia known from Sweden (Kjaerandsen et al. 2007), Finland and Murmansk Province (Jakovlev and Penttinen 2007, Polevoi 2010). New to the Republic of Karelia.

#### Ecology

Karelian specimens were collected in *Vaccinium myrtillus* type coniferous forests, the Finnish sampling sites are chiefly old-growth coniferous forest, but also burnt clear cuts with some retained trees. Larvae live in bracket fungi, most rearing records are from *Piptoporus betulinus* (Jakovlev 1994, Ševčík 2010).

### Mycetophila lobulata

Zaitzev, 1999*

http://www.faunaeur.org/full_results.php?id=140422

#### Materials

- **Type status:** Other material. **Occurrence:** recordedBy: J.Penttinen; individualCount: 1; sex: male; **Location:** country: Finland; stateProvince: Regio aboënsis; municipality: Perniö; locality: Matilda; decimalLatitude: 60.329; decimalLongitude: 23.670; geodeticDatum: WGS84; **Identification:** identifiedBy: J.Penttinen; **Event:** samplingProtocol: Malaise trap; eventDate: 2009-8-15/9-15; habitat: Semi-natural forest with lime; **Record Level:** institutionCode: JPJ

#### Distribution

Palaearctic. The species was described from Russia (European part and Far East, Zaitzev 1999) and has been later found from southern Sweden (Kurina et al. 2004). No previous records from Finland.

#### Ecology

In Sweden collected from a mixed forest (Kurina et al. 2004), the Finnish sampling site is a herb-rich forest in the hemiboreal zone. Saproxylic, larvae were found in a bracket fungus (*Inonotus* sp; Zaitzev 1999, Zaitzev 2003).

### Mycetophila mohilevensis

Dziedzicki, 1884**

http://www.faunaeur.org/full_results.php?id=140433

#### Materials

- **Type status:** Other material. **Occurrence:** recordedBy: A. Polevoi; individualCount: 1; sex: male; **Location:** country: Russia; stateProvince: Republic Karelia; verbatimLocality: Obzha, 1 km NE of Ustje Obzhanki; decimalLatitude: 60.828; decimalLongitude: 32.83; geodeticDatum: WGS84; **Identification:** identifiedBy: A. Polevoi; **Event:** samplingProtocol: Sweep netting; eventDate: 2012-6-23; **Record Level:** institutionCode: FRIP
- **Type status:** Other material. **Occurrence:** recordedBy: A. Polevoi; individualCount: 1; sex: male; **Location:** country: Russia; stateProvince: Republic Karelia; verbatimLocality: 2 km NW of Besovets; decimalLatitude: 61.869; decimalLongitude: 34.116; geodeticDatum: WGS84; **Identification:** identifiedBy: A. Polevoi; **Event:** samplingProtocol: Pitfall trap; eventDate: 2009-10-12/16; **Record Level:** institutionCode: FRIP

#### Distribution

Palaearctic. Scattered records in Europe and East Russia (Chandler 2004, Zaitzev 2003). In Fennoscandia known from Finland (Hackman 1980), Norway (Gammelmo and Søli 2006) and Sweden (Kjaerandsen et al. 2007). New to the Republic of Karelia.

#### Ecology

Collected in *Vaccinium myrtillus* type coniferous forests in different succession stages. Larvae live in lignicolous fungi, rearing records exist from *Piptoporus betulinus* (Zaitzev 2003) and *Tyromyces chioneus* (Ševčík 2010).

### Mycetophila monstera

Maximova, 2002***

http://www.catalogueoflife.org/col/details/species/id/8760852

#### Materials

- **Type status:** Other material. **Occurrence:** catalogNumber: MYCE-JS-2013-0391; recordedBy: J. Salmela; S. Lapinniemi; individualCount: 1; sex: male; **Location:** country: Finland; stateProvince: Lapponia enontekiensis; verbatimLocality: Pallas-Yllästunturi National Park, Röyninkuru; verbatimElevation: 380 m; decimalLatitude: 68.146; decimalLongitude: 24.071; geodeticDatum: WGS84; **Identification:** identifiedBy: J. Salmela; **Event:** samplingProtocol: Malaise trap; eventDate: 2013-6-5/7-6; habitat: headwater stream, old-growth spruce forest; **Record Level:** institutionCode: JES

#### Distribution

Palaearctic. The species was recently described from West Siberia (Maximova 2002), here reported for the first time from Europe and Finland.

#### Ecology

Apparently a forest-dwelling, boreal species. The Finnish sampling site is a headwater stream surrounded by an old-growth spruce forest. Immature stages are unknown.

### Mycetophila ostentanea

Zaitzev, 1998**

http://www.faunaeur.org/full_results.php?id=140454

#### Materials

- **Type status:** Other material. **Occurrence:** recordedBy: M. Tietäväinen et al.; individualCount: 2; sex: male; **Location:** country: Russia; stateProvince: Republic Karelia; verbatimLocality: 5 km N of Tolvojarvi; decimalLatitude: 62.318; decimalLongitude: 31.436; geodeticDatum: WGS84; **Identification:** identifiedBy: A. Polevoi; **Event:** samplingProtocol: Malaise trap; eventDate: 1998-8-29/9-11; **Record Level:** institutionCode: FRIP

#### Distribution

European. Described from Vologda Province in Russia (Zaitzev 1998). Also recorded from Czech Republic (Ševčík 2001b) and Finland (Polevoi et al. 2006). New to the Republic of Karelia.

#### Ecology

Collected in *Vaccinium myrtillus* type spruce dominated forest. Reared by Ševčík (Ševčík 2010) from the polyporous fungus *Postia undosa*.

### Mycetophila pyrenaica

Matile, 1967*

http://www.faunaeur.org/full_results.php?id=140471

#### Materials

- **Type status:** Other material. **Occurrence:** recordedBy: J.Penttinen; individualCount: 1; sex: male; **Location:** country: Finland; stateProvince: Tavastia australis; municipality: Tammela; locality: Pehkijärvi; decimalLatitude: 59.963; decimalLongitude: 23.585; geodeticDatum: WGS84; **Identification:** identifiedBy: J.Penttinen; **Event:** samplingProtocol: Malaise trap; eventDate: 2099-6-1/6-15; habitat: aspen dominated HRT in Häme, close to Forss; **Record Level:** institutionCode: JPJ

#### Distribution

Palaearctic. Described from France (Matile 1967) and has been later recorded from Germany (Chandler 2004), Poland (Kurina and Ševčík 2006), Russia (Zaitzev 2003), Norway (Anonymous 2010, Gammelmo and Søli 2006), Sweden (Kurina et al. 2004) and the Italian Alps (Kurina 2008). New for Finland.

#### Ecology

Immature stages are unknown. The Finnish collecting site is a herb-rich forest dominated by aspen and spruce.

#### Conservation

Red-listed in Norway (VU, Anonymous 2010, Gammelmo et al. 2010).

### Mycetophila sigmoides

Loew, 1869*

http://www.faunaeur.org/full_results.php?id=140484

#### Materials

- **Type status:** Other material. **Occurrence:** recordedBy: J.Jakovlev; individualCount: 1; sex: male; **Location:** country: Finland; stateProvince: Nylandia; municipality: Helsinki; locality: Tullisaari, Stansvikin kartano; decimalLatitude: 60.166; decimalLongitude: 25.027; geodeticDatum: WGS84; **Identification:** identifiedBy: J.Jakovlev; **Event:** samplingProtocol: Malaise trap; eventDate: 2011-6-12/8-2; habitat: City parks_old protected mansion park in the city of Helsinki with numerous old hollow deciduous trees, mainly lime trees, oaks and maples; **Record Level:** institutionCode: JJH
- **Type status:** Other material. **Occurrence:** recordedBy: J.Jakovlev; individualCount: 1; sex: male; **Location:** country: Finland; stateProvince: Regio aboënsis; municipality: Karjalohja; locality: Karkali_South; decimalLatitude: 60.238; decimalLongitude: 23.785; geodeticDatum: WGS84; **Identification:** identifiedBy: J.Jakovlev; **Event:** samplingProtocol: Malaise trap; eventDate: 2004-8-23/10-4; habitat: old-growth forest, herb-ric type; **Record Level:** institutionCode: JJH

#### Distribution

Holarctic. The species was described from USA (Loew 1869) and has been reported from Canada (Laffoon 1957), Russia (Siberia and Far East, Zaitzev 2003, Karelia, Polevoi and Humala 2005), Czech republic, Hungary (Chandler 2004), Britain, France and northern Italy (Gibbs 2009), Norway (Kjaerandsen and Jordal 2007) and Sweden (Kjaerandsen 2012). Here reported formally as new for Finland.

#### Ecology

Larvae are associated with wood-decaying polyporous fungi, *Coriolus*, *Daedaliopsis* and *Fomitopsis* (Zaitzev 2003, Ševčík 2010). The Finnish collecting site is a hemiboreal herb-rich forest.

#### Conservation

Red-listed in Norway (DD, Anonymous 2010) and Finland (VU, Penttinen et al. 2010). This species has only been recorded in Britain since 1998 but is now widespread in southern England, suggesting that it is a recent arrival. Records elsewhere in western Europe suggest that it has recently spread and it is possible that its spread into Fennoscandia has also been recent (P.Chandler, pers. comm). An increase in records might therefore be expected, so red-list status may be premature.

### Mycetophila sinuosa

Plassmann & Schacht, 1999*

http://www.faunaeur.org/full_results.php?id=140490

#### Materials

- **Type status:** Other material. **Occurrence:** recordedBy: J.Penttinen; individualCount: 1; sex: male; **Location:** country: Finland; stateProvince: Regio aboënsis; municipality: Salo; locality: Märy; decimalLatitude: 60.223; decimalLongitude: 22.905; geodeticDatum: WGS84; **Identification:** identifiedBy: J.Penttinen; **Event:** samplingProtocol: Malaise trap; eventDate: 2009-8-15/9-15; habitat: old managed forest, herb-rich type, large oak trees; **Record Level:** institutionCode: JPJ

#### Distribution

European. The species was described from Germany (Plassmann and Schacht 1999) and has been later recorded from Czech republic (Ševčík 2001a), Switzerland (Chandler 2004) and southern Sweden (Kjaerandsen et al. 2007). New for Finland.

#### Ecology

The Finnish sampling site is a herb-rich forest characterized by old oak trees. Immature stages are unknown.

### Mycetophila triangularis

Lundström, 1912**

http://www.faunaeur.org/full_results.php?id=140513

#### Materials

- **Type status:** Other material. **Occurrence:** recordedBy: M. Tietäväinen et al.; individualCount: 2; sex: male; **Location:** country: Russia; stateProvince: Republic Karelia; verbatimLocality: 5 km N of Tolvojarvi; decimalLatitude: 62.318; decimalLongitude: 31.436; geodeticDatum: WGS84; **Identification:** identifiedBy: A. Polevoi; **Event:** samplingProtocol: Malaise trap; eventDate: 1999-6-11/22; **Record Level:** institutionCode: FRIP

#### Distribution

Palaearctic. Rare species which was known for a long time only from the type locality, Ukraine (Lundström 1912). It has since been recorded also from Czech Republic, Slovakia (Ševčík 2004) and Altai (Zaitzev 2003). New to the Republic of Karelia and Fennoscandia.

#### Ecology

Collected in *Vaccinium myrtillus* type coniferous forest in different succession stages. Immature stages are unknown.

### Mycetophila uliginosa

Chandler, 1988*

http://www.faunaeur.org/full_results.php?id=140523

#### Materials

- **Type status:** Other material. **Occurrence:** recordedBy: J.Jakovlev and J.Penttinen; individualCount: 1; sex: male; **Location:** country: Finland; stateProvince: Lapponia enontekiensis; municipality: Enontekiö; locality: Kilpisjärvi_Saana_South_1; decimalLatitude: 69.033; decimalLongitude: 20.838; geodeticDatum: WGS84; **Identification:** identifiedBy: J.Penttinen; **Event:** samplingProtocol: Malaise trap; eventDate: 2006-7-15/8-1; habitat: mountain birch forest
- **Type status:** Other material. **Occurrence:** recordedBy: Noora Vartija; individualCount: 1; sex: male; **Location:** country: Finland; stateProvince: Tavastia australis; municipality: Muurame; locality: Kuusimäki Forest Reserve; decimalLatitude: 62.215; decimalLongitude: 25.496; geodeticDatum: WGS84; **Identification:** identifiedBy: J.Penttinen; **Event:** samplingProtocol: Reared from wood; eventDate: 2008-7-8/8-4; habitat: old-growth forest, Myrtillus type; **Record Level:** institutionCode: JPJ
- **Type status:** Other material. **Occurrence:** recordedBy: Noora Vartija; individualCount: 1; sex: male; **Location:** country: Finland; stateProvince: Tavastia australis; municipality: Muurame; locality: Kuusimäki Forest Reserve; decimalLatitude: 62.215; decimalLongitude: 25.496; geodeticDatum: WGS84; **Identification:** identifiedBy: J.Penttinen; **Event:** samplingProtocol: Reared from wood; eventDate: 2008-6-9/7-7; habitat: old-growth forest, Myrtillus type; **Record Level:** institutionCode: JPJ
- **Type status:** Other material. **Occurrence:** recordedBy: J.Penttinen; individualCount: 1; sex: male; **Location:** country: Finland; stateProvince: Savonia australis; municipality: Rantasalmi; locality: Linnansaari; decimalLatitude: 62.116; decimalLongitude: 28.477; geodeticDatum: WGS84; **Identification:** identifiedBy: J.Penttinen; **Event:** samplingProtocol: Malaise trap; eventDate: 2008-7-25/9-4; habitat: old-growth forest, herb-rich type; **Record Level:** institutionCode: JPJ
- **Type status:** Other material. **Occurrence:** recordedBy: J.Penttinen; individualCount: 1; sex: male; **Location:** country: Finland; stateProvince: Tavastia borealis; municipality: Saarijärvi; locality: Pyhä-Häkki National Park; decimalLatitude: 62.842; decimalLongitude: 25.474; geodeticDatum: WGS84; **Identification:** identifiedBy: J.Penttinen; **Event:** samplingProtocol: Malaise trap; eventDate: 2008; habitat: old-growth forest, Myrtillus type; **Record Level:** institutionCode: JPJ
- **Type status:** Other material. **Occurrence:** recordedBy: J.Jakovlev and J.Penttinen; individualCount: 1; sex: male; **Location:** country: Finland; stateProvince: Lapponia enontekiensis; municipality: Enontekiö; locality: Kilpisjärvi_Saana_North_4; decimalLatitude: 69.045; decimalLongitude: 20.808; geodeticDatum: WGS84; **Identification:** identifiedBy: J.Jakovlev; **Event:** samplingProtocol: Malaise trap; eventDate: 2006-8-1/8-15; habitat: mountain birch forest
- **Type status:** Other material. **Occurrence:** catalogNumber: MYCE-NV-2013-0136; recordedBy: J. Salmela; individualCount: 1; sex: male; **Location:** country: Finland; stateProvince: Lapponia kemensis pars orientalis; verbatimLocality: Sodankylä, Ylä-Postojoki; decimalLatitude: 67.851; decimalLongitude: 26.481; geodeticDatum: WGS84; **Identification:** identifiedBy: N. Vartija; J. Salmela; **Event:** samplingProtocol: Malaise trap; eventDate: 2009-6-1/29; habitat: riparian forest; **Record Level:** institutionCode: JES

#### Distribution

European. The species was described from the British Isles (Chandler 1988) and has been since recorded from Spain, France (Chandler 2004), Norway (Kjaerandsen and Jordal 2007, Anonymous 2010) and Sweden (Kjaerandsen et al. 2007). New for Finland.

#### Ecology

Reared from decaying logs *in situ* (eclector traps). The Finnish collecting sites are a mountain birch forest, a riparian forest and old-growth boreal forests. Biology unknown. The larvae probably develop in lignicolous fungi (Falk and Chandler 2005).

#### Conservation

Red-listed in Norway (DD, Anonymous 2010).

### Mycetophila unguiculata

Lundström, 1913**

http://www.faunaeur.org/full_results.php?id=140524

#### Materials

- **Type status:** Other material. **Occurrence:** recordedBy: A. Polevoi; individualCount: 1; sex: male; **Location:** country: Russia; stateProvince: Leningrad province; verbatimLocality: Voznesenje, 1 km SE of Gimreka; decimalLatitude: 61.151; decimalLongitude: 35.642; geodeticDatum: WGS84; **Identification:** identifiedBy: A. Polevoi; **Event:** samplingProtocol: Malaise trap; eventDate: 2008-4-23/5-25; **Record Level:** institutionCode: FRIP
- **Type status:** Other material. **Occurrence:** recordedBy: A. Polevoi; individualCount: 1; sex: male; **Location:** country: Russia; stateProvince: Leningrad province; verbatimLocality: Voznesenje, 1 km SE of Gimreka; decimalLatitude: 61.151; decimalLongitude: 35.642; geodeticDatum: WGS84; **Identification:** identifiedBy: A. Polevoi; **Event:** samplingProtocol: Malaise trap; eventDate: 2008-8-27/10-1; **Record Level:** institutionCode: FRIP

#### Distribution

Palaearctic. Scattered records from Europe and West Siberia (Chandler 2004, Zaitzev 2003). In Fennoscandia known from Norway (Gammelmo and Søli 2006) and Sweden (Kjaerandsen et al. 2007). New to the Republic of Karelia.

#### Ecology

The Karelian specimens were collected in secondary *Vaccinium myrtillus* type pinedominated forest. Immature stages are unknown.

### Mycetophila uschaica

Subbotina & Maximova, 2011***

#### Materials

- **Type status:** Other material. **Occurrence:** recordedBy: A. Polevoi; individualCount: 1; sex: male; **Location:** country: Russia; stateProvince: Republic Karelia; verbatimLocality: Tipinitsy, 4 km S of Polya; decimalLatitude: 62.29; decimalLongitude: 35.309; geodeticDatum: WGS84; **Identification:** identifiedBy: A. Polevoi; **Event:** samplingProtocol: Sweep netting; eventDate: 2013-8-25; **Record Level:** institutionCode: FRIP

#### Distribution

Palaearctic. The species was known only from the type locality in West Siberia, Tomsk Province (Subbotina and Maximova 2011). New to the Republic of Karelia and Europe.

#### Ecology

Karelian specimens were collected in *Vaccinium myrtillus* type spruce dominated forest. Immature stages are unknown.

### Phronia gracilis

Hackman, 1970

http://www.faunaeur.org/full_results.php?id=140266

#### Materials

- **Type status:** Other material. **Occurrence:** catalogNumber: MYCE-JS-2013-0179; recordedBy: J. Salmela; individualCount: 1; sex: male; **Location:** country: Finland; stateProvince: Lapponia kemensis pars orientalis; verbatimLocality: Savukoski, Törmäoja; decimalLatitude: 67.835; decimalLongitude: 29.454; geodeticDatum: WGS84; **Identification:** identifiedBy: J. Salmela; **Event:** samplingProtocol: Malaise trap; eventDate: 2012-6-14/7-10; habitat: headwater stream, old-growth boreal forest; **Record Level:** institutionCode: JES

#### Distribution

European. The species was described from NE Finland, Kuusamo, Jäkälävuoma (Hackman 1970) and has been since recorded only from Germany (Chandler 2004). The species has a characteristic eastern distribution in Finland, so far known from the Kainuu and Kuusamo areas (Hackman 1970, Jakovlev 2011b Jakovlev 2011b) here reported for the first time from NE Lapland.

#### Ecology

Collected from old-growth boreal forests. Immature stages are unknown. Larvae of Trichonta and Phronia are usually surface feeders on encrusting fungi and slime moulds (Gagné 1975, Gagné 1981).

#### Conservation

Red-listed (NT) in Finland (Penttinen et al. 2010).

### Phronia humeralis

Winnertz, 1863**

http://www.faunaeur.org/full_results.php?id=140267

#### Materials

- **Type status:** Other material. **Occurrence:** recordedBy: A. Polevoi; individualCount: 2; sex: male; **Location:** country: Russia; stateProvince: Republic Karelia; verbatimLocality: Obzha, Mayachino; decimalLatitude: 60.777; decimalLongitude: 32.818; geodeticDatum: WGS84; **Identification:** identifiedBy: A. Polevoi; **Event:** samplingProtocol: Sweep netting; eventDate: 2012-6-25; **Record Level:** institutionCode: FRIP

#### Distribution

Palaearctic. Widely distributed in Europe (Chandler 2004, Zaitzev 2003), recorded from West Siberia (Ostroverkhova 1979). In Fennoscandia known from Finland (Hackman 1980), Norway (Gammelmo and Søli 2006) and Sweden (Kjaerandsen et al. 2007). New to the Republic of Karelia.

#### Ecology

Collected in herb-rich spruce dominated forest. Larvae are associated with dead wood and wood-growing fungi. Reared from *Corticium praetermissum* (Buxton 1960), *Chondrostereum purpureum*, and repeatedly obtained with eclector traps over decaying logs (Jakovlev 2011a).

### Phronia maculata

Dziedzicki, 1889**

http://www.faunaeur.org/full_results.php?id=140275

#### Materials

- **Type status:** Other material. **Occurrence:** recordedBy: A. Polevoi; individualCount: 1; sex: male; **Location:** country: Russia; stateProvince: Leningrad province; verbatimLocality: Voznesenje, 1 km E of Gimreka; decimalLatitude: 61.151; decimalLongitude: 35.64; geodeticDatum: WGS84; **Identification:** identifiedBy: A. Polevoi; **Event:** samplingProtocol: Malaise trap; eventDate: 2008-4-23/5-25; **Record Level:** institutionCode: FRIP
- **Type status:** Other material. **Occurrence:** recordedBy: A. Polevoi; individualCount: 1; sex: male; **Location:** country: Russia; stateProvince: Leningrad province; verbatimLocality: Voznesenje, 1 km E of Gimreka; decimalLatitude: 61.151; decimalLongitude: 35.64; geodeticDatum: WGS84; **Identification:** identifiedBy: A. Polevoi; **Event:** samplingProtocol: Malaise trap; eventDate: 2008-8-27/10-1; **Record Level:** institutionCode: FRIP
- **Type status:** Other material. **Occurrence:** recordedBy: A. Polevoi; individualCount: 1; sex: male; **Location:** country: Russia; stateProvince: Republic Karelia; verbatimLocality: Velikaya Guba, 2 km NW of Uzkaya Salma; decimalLatitude: 62.14; decimalLongitude: 34.941; geodeticDatum: WGS84; **Identification:** identifiedBy: A. Polevoi; **Event:** samplingProtocol: Sweep netting; eventDate: 2013-6-28; **Record Level:** institutionCode: FRIP
- **Type status:** Other material. **Occurrence:** recordedBy: A. Polevoi; individualCount: 1; sex: male; **Location:** country: Russia; stateProvince: Republic Karelia; verbatimLocality: Velikaya Guba, 5 km NE of Lipovitsy; decimalLatitude: 62.132; decimalLongitude: 35.093; geodeticDatum: WGS84; **Identification:** identifiedBy: A. Polevoi; **Event:** samplingProtocol: Sweep netting; eventDate: 2013-6-25; **Record Level:** institutionCode: FRIP
- **Type status:** Other material. **Occurrence:** recordedBy: A. Polevoi; individualCount: 3; sex: male; **Location:** country: Russia; stateProvince: Republic Karelia; verbatimLocality: 11 km W of Tipinitsy; decimalLatitude: 62.185; decimalLongitude: 35.311; geodeticDatum: WGS84; **Identification:** identifiedBy: A. Polevoi; **Event:** samplingProtocol: Sweep netting; eventDate: 2013-8-27; **Record Level:** institutionCode: FRIP
- **Type status:** Other material. **Occurrence:** catalogNumber: MYCE-JS-2013-0340; recordedBy: J. Ilmonen; individualCount: 1; sex: male; **Location:** country: Finland; stateProvince: Nylandia; municipality: Espoo; locality: Matalajärvi; decimalLatitude: 60.247; decimalLongitude: 24.687; geodeticDatum: WGS84; **Identification:** identifiedBy: J. Salmela; **Event:** samplingProtocol: Malaise trap; eventDate: 2012-7-21/8-23; habitat: swampy lake shore; **Record Level:** institutionCode: JES
- **Type status:** Other material. **Occurrence:** recordedBy: J.Penttinen; individualCount: 2; sex: male; **Location:** country: Finland; stateProvince: Savonia australis; municipality: Rantasalmi; locality: Linnansaari; decimalLatitude: 62.116; decimalLongitude: 28.477; geodeticDatum: WGS84; **Identification:** identifiedBy: J.Penttinen; **Event:** samplingProtocol: Malaise trap; eventDate: 2008-5-20/6-24; habitat: dry herb-rich forest; **Record Level:** institutionCode: JPJ

#### Distribution

Palaearctic. Widely disributed in Europe and East Russia (Chandler 2004, Zaitzev 2003). In Fennoscandia known from Finland (Hackman 1980) and Sweden (Kjaerandsen et al. 2007). New to the Republic of Karelia.

#### Ecology

The Karelian specimens were collected in *Vaccinium myrtillus* type coniferous forests in different succession stages. The Finnish specimen was collected from a swampy lake shore of an eutrophic lake. Immature stages are unknown.

#### Conservation

Red-listed in Finland, presumed to occur in herb-rich forests (VU, Penttinen et al. 2010).

### Phronia mutila

Lundström, 1911*

http://www.faunaeur.org/full_results.php?id=140281

#### Materials

- **Type status:** Other material. **Occurrence:** recordedBy: J.Jakovlev and J.Penttinen; individualCount: 3; sex: 1 male, 2 females; **Location:** country: Finland; stateProvince: Lapponia enontekiensis; municipality: Enontekiö; locality: Kilpisjärvi_Saana_South_1; decimalLatitude: 69.033; decimalLongitude: 20.837; geodeticDatum: WGS84; **Identification:** identifiedBy: J.Jakovlev; **Event:** samplingProtocol: Malaise trap; eventDate: 2006-6-19/7-14; habitat: subarctic mountain birch forest; **Record Level:** institutionCode: JPJ

#### Distribution

European. This very rare species was only known from the Austrian type material (Lundström 1911) and from one recent record from Russian Karelia, from the shore of the White Sea (Humala and Polevoi 2008). No former records from Finland. The species is possibly arctic-alpine.

#### Ecology

The Finnish sampling locality is a mountain birch forest in NW Lapland. Immature stages are unknown.

### Phronia signata

Winnertz, 1863**

http://www.faunaeur.org/full_results.php?id=140303

#### Materials

- **Type status:** Other material. **Occurrence:** recordedBy: A. Polevoi; individualCount: 1; sex: male; **Location:** country: Russia; stateProvince: Republic Karelia; verbatimLocality: 3 km NW of Sheltozero; decimalLatitude: 61.393; decimalLongitude: 35.308; geodeticDatum: WGS84; **Identification:** identifiedBy: A. Polevoi; **Event:** samplingProtocol: Sweep netting; eventDate: 2004-7-13; **Record Level:** institutionCode: FRIP
- **Type status:** Other material. **Occurrence:** recordedBy: A. Polevoi; individualCount: 1; sex: male; **Location:** country: Russia; stateProvince: Republic Karelia; verbatimLocality: Tambitsy, Kaskosel'ga; decimalLatitude: 62.242; decimalLongitude: 35.492; geodeticDatum: WGS84; **Identification:** identifiedBy: A. Polevoi; **Event:** samplingProtocol: Sweep netting; eventDate: 2013-8-28; **Record Level:** institutionCode: FRIP

#### Distribution

Palaearctic. Widely distributed in Europe and East Russia (Chandler 2004, Zaitzev 2003). In Fennoscandia known from Finland (Hackman 1980), Norway (Gammelmo and Søli 2006) and Sweden (Kjaerandsen et al. 2007). New to the Republic of Karelia.

#### Ecology

The Karelian specimens were collected in *Vaccinium myrtillus* type spruce dominated forests. Immature stages are unknown.

### Sceptonia flavipuncta

Edwards, 1925*

http://www.faunaeur.org/full_results.php?id=140188

#### Materials

- **Type status:** Other material. **Occurrence:** recordedBy: J.Penttinen; individualCount: 1; sex: male; **Location:** country: Finland; stateProvince: Karelia ladogensis; municipality: Parikkala; locality: Siikalahti; decimalLatitude: 61.556; decimalLongitude: 29.558; geodeticDatum: WGS84; **Identification:** identifiedBy: J.Penttinen; **Event:** samplingProtocol: Malaise trap; eventDate: 2008-7-22/9-1; habitat: old-growth forest, herb-rich type; **Record Level:** institutionCode: JPJ

#### Distribution

European. Described from Great Britain (Edwards 1925), later recorded from many countries in western and southern Europe (Chandler 2004). In Fennoscandia recorded from Russian Karelia (Polevoi et al. 2005), Sweden and Norway (Kjaerandsen et al. 2007, Kjaerandsen 2012). Here formally reported as new for Finland.

#### Ecology

The only Finnish sampling locality is a herb-rich forest. Reared from *Rhodocybe gemina* in Czech Republic (Ševčík 2010).

#### Conservation

Included in the Red List of Finnish species (NT, Penttinen et al. 2010).

### Trichonta generosa

Gagne, 1981*

http://www.faunaeur.org/full_results.php?id=140139

#### Materials

- **Type status:** Other material. **Occurrence:** recordedBy: M.Jaschhof and C.Jaschhof; individualCount: 1; sex: male; **Location:** country: Finland; stateProvince: Regio kuusamoënsis; municipality: Kuusamo; locality: Kuohusuo-Kalliovaara; decimalLatitude: 65.674; decimalLongitude: 28.888; geodeticDatum: WGS84; **Identification:** identifiedBy: J.Jakovlev; **Event:** samplingProtocol: Sweep netting; eventDate: 2004-7-30/7-30; habitat: old managed forest, Myrtillus type; **Record Level:** institutionCode: JJH
- **Type status:** Other material. **Occurrence:** recordedBy: J.Jakovlev and J.Penttinen; individualCount: 1; sex: male; **Location:** country: Finland; stateProvince: Lapponia enontekiensis; municipality: Enontekiö; locality: Kilpisjärvi_Saana_North_3; decimalLatitude: 69.044; decimalLongitude: 20.807; geodeticDatum: WGS84; **Identification:** identifiedBy: J.Jakovlev; **Event:** samplingProtocol: Malaise trap; eventDate: 2006-8-1/8-15; habitat: Subarctic
- **Type status:** Other material. **Occurrence:** recordedBy: J.Jakovlev and J.Penttinen; individualCount: 1; sex: male; **Location:** country: Finland; stateProvince: Lapponia enontekiensis; municipality: Enontekiö; locality: Kilpisjärvi_Saana_North_4; decimalLatitude: 69.045; decimalLongitude: 20.808; geodeticDatum: WGS84; **Identification:** identifiedBy: J.Jakovlev; **Event:** samplingProtocol: Malaise trap; eventDate: 2006-8-1/8-15; habitat: subarctic
- **Type status:** Other material. **Occurrence:** recordedBy: J.Jakovlev; individualCount: 1; sex: male; **Location:** country: Finland; stateProvince: Lapponia enontekiensis; municipality: Enontekiö; locality: Kilpisjärvi_Saana_North_5; decimalLatitude: 69.045; decimalLongitude: 20.809; geodeticDatum: WGS84; **Identification:** identifiedBy: J.Jakovlev; **Event:** samplingProtocol: Sweep netting; eventDate: 2006-6-21/6-21; habitat: Subarctic; **Record Level:** institutionCode: JJH
- **Type status:** Other material. **Occurrence:** recordedBy: J.Jakovlev and J.Penttinen; individualCount: 2; sex: 2 male; **Location:** country: Finland; stateProvince: Lapponia enontekiensis; municipality: Enontekiö; locality: Kilpisjärvi_Saana_South_1; decimalLatitude: 69.033; decimalLongitude: 20.837; geodeticDatum: WGS84; **Identification:** identifiedBy: J.Jakovlev; **Event:** samplingProtocol: Malaise trap; eventDate: 2006-8-1/8-15; habitat: subarctic
- **Type status:** Other material. **Occurrence:** recordedBy: J.Jakovlev and J.Penttinen; individualCount: 1; sex: male; **Location:** country: Finland; stateProvince: Lapponia enontekiensis; municipality: Enontekiö; locality: Kilpisjärvi_Saana_South_2; decimalLatitude: 69.035; decimalLongitude: 20.839; geodeticDatum: WGS84; **Identification:** identifiedBy: J.Jakovlev; **Event:** samplingProtocol: Malaise trap; eventDate: 2006-8-1/8-15; habitat: subarctic
- **Type status:** Other material. **Occurrence:** recordedBy: J.Jakovlev and J.Penttinen; individualCount: 1; sex: male; **Location:** country: Finland; stateProvince: Lapponia kemensis pars occidentalis; municipality: Muonio; locality: Pallas-Yllästunturi National Park_Pallas_2; decimalLatitude: 68.018; decimalLongitude: 24.153; geodeticDatum: WGS84; **Identification:** identifiedBy: A.Polevoi; **Event:** samplingProtocol: Malaise trap; eventDate: 2006-6-18/7-14; habitat: old-growth forest, Myrtillus type
- **Type status:** Other material. **Occurrence:** recordedBy: J.Salmela; individualCount: 1; sex: male; **Location:** country: Finland; stateProvince: Lapponia inarensis; municipality: Utsjoki; locality: Galddasjohka; decimalLatitude: 69.861; decimalLongitude: 27.803; geodeticDatum: WGS84; **Identification:** identifiedBy: J.Jakovlev; **Event:** samplingProtocol: Malaise trap; eventDate: 2007-6-15/7-19; habitat: subarctic; **Record Level:** institutionCode: JJH
- **Type status:** Other material. **Occurrence:** catalogNumber: MYCE-NV-2013-0151; recordedBy: J.Salmela; individualCount: 1; sex: male; **Location:** country: Finland; stateProvince: Lapponia kemensis pars orientalis; verbatimLocality: Sodankylä, Ylä-Postojoki; decimalLatitude: 67.851; decimalLongitude: 26.481; geodeticDatum: WGS84; **Identification:** identifiedBy: N. Vartija; J. Salmela; **Event:** samplingProtocol: Malaise trap; eventDate: 2009-6-1/29; habitat: headwater stream; **Record Level:** institutionCode: JES

#### Distribution

Holarctic. Described from North America (Gagné 1981), later found in the Altai Mountains, western Siberia (Zaitzev 2003). In Europe recorded so far only from Norway (Økland and Zaitzev 1997, Søli and Rindal 2012) and Murmansk region of NW Russia (Polevoi 2010). No former records from Finland; all findings reported here are from North Finland.

#### Ecology

Finnish collecting sites are old-growth boreal forests, mountain birch forests and a riparian forest. Immature stages are unknown.

#### Conservation

Red-listed in Norway (DD, Anonymous 2010, Gammelmo et al. 2010).

### Trichonta palustris

Maximova, 2002***

http://www.catalogueoflife.org/col/details/species/id/8760853

#### Materials

- **Type status:** Other material. **Occurrence:** catalogNumber: DIPT-JS-2014-0004; recordedBy: J. Salmela; T. Hietajärvi; individualCount: 1; sex: male; **Location:** country: Finland; stateProvince: Regio kuusamoensis; verbatimLocality: Salla, Kuntasjoki, Värriö Strict Nature Reserve; verbatimElevation: 320 m; decimalLatitude: 67.749; decimalLongitude: 29.617; geodeticDatum: WGS84; **Identification:** identifiedBy: J. Salmela; **Event:** samplingProtocol: Malaise trap; eventDate: 2013; verbatimEventDate: 2013-6-4/29; habitat: headwater stream, old-growth boreal forest; **Record Level:** institutionCode: JES

#### Distribution

Palaearctic. The species was described from West Siberia, Kutznetskyi Alatau Nature Reserve, based on a holotype male (Maximova 2002). Here reported for the first time from Europe. The Finnish sampling locality is in NE Lapland, north boreal zone.

#### Ecology

The holotype male was collected from a swamp (Maximova 2002). The Malaise trapping locality in Värriö is a headwater stream with wet margins, including seepages and rich riparian vegetation, surrounded by old-growth boreal forest. Immature stages are unknown.

### Trichonta tristis

(Strobl, 1898)**

http://www.faunaeur.org/full_results.php?id=140163

#### Materials

- **Type status:** Other material. **Occurrence:** recordedBy: A. Polevoi; individualCount: 1; sex: male; **Location:** country: Russia; stateProvince: Leningrad province; verbatimLocality: Voznesehje, 1 km E of Gimreka; decimalLatitude: 61.151; decimalLongitude: 35.64; geodeticDatum: WGS84; **Identification:** identifiedBy: A. Polevoi; **Event:** samplingProtocol: Malaise trap; eventDate: 2008-4-23/5-25; **Record Level:** institutionCode: FRIP
- **Type status:** Other material. **Occurrence:** recordedBy: A. Polevoi; individualCount: 1; sex: male; **Location:** country: Russia; stateProvince: Republic Karelia; verbatimLocality: Kivach Nature Reserve; decimalLatitude: 62.272; decimalLongitude: 33.986; geodeticDatum: WGS84; **Identification:** identifiedBy: A. Polevoi; **Event:** samplingProtocol: Malaise trap; eventDate: 1989-8-24/9-25; **Record Level:** institutionCode: FRIP

#### Distribution

Palaearctic. Recorded from Austria and Switzerland (Chandler 2004), southern Finland (Jakovlev and Penttinen 2007), Murmansk Province (Polevoi 2010) and East Russia (Zaitzev 2003). New to the Republic of Karelia.

#### Ecology

The Karelian specimens were collected in mixed and aspen dominated deciduous forests. In Finland the species was reared from a decaying spruce log bearing polypore fungus *Antrodia xantha* (Jakovlev and Penttinen 2007).

#### Notes

This species is closely related to widely distributed *Trichonta vulcani* Dziedzicki and might be overlooked in other countries (Jakovlev and Penttinen 2007).

## Figures and Tables

**Figure 3a.**
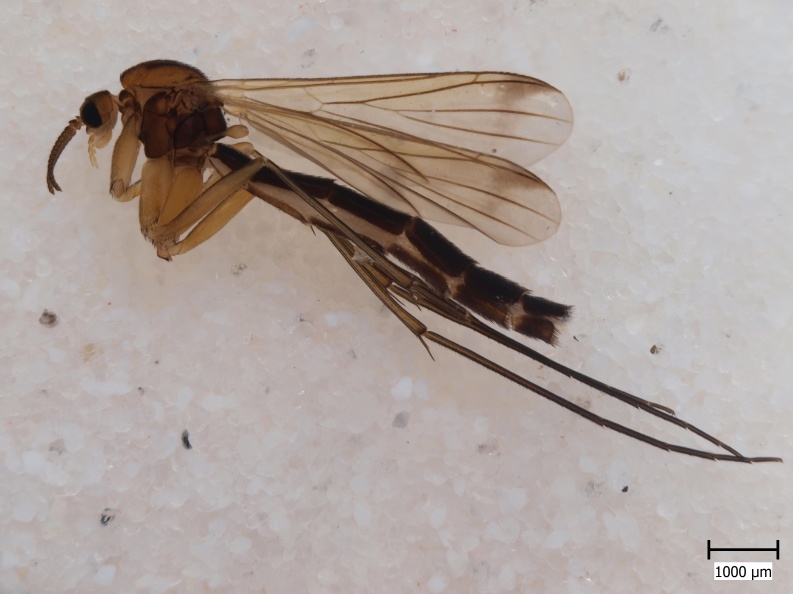
Habitus, lateral view.

**Figure 3b.**
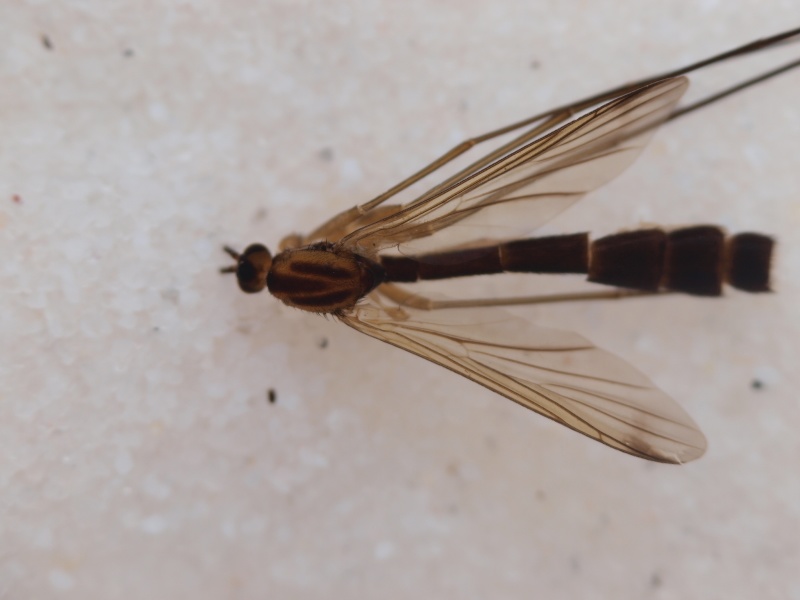
Habitus, dorsal view.

**Figure 3c.**
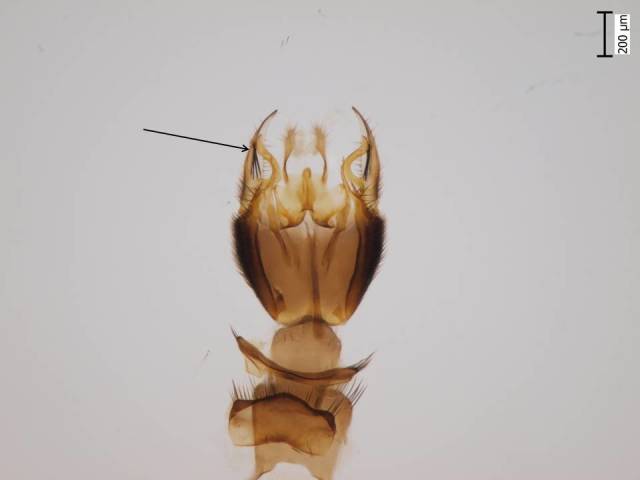
Male hypopgium, tergal view. Arrow points to the apex of sinuous style bearing a dense cluster of hairs.

**Figure 3d.**
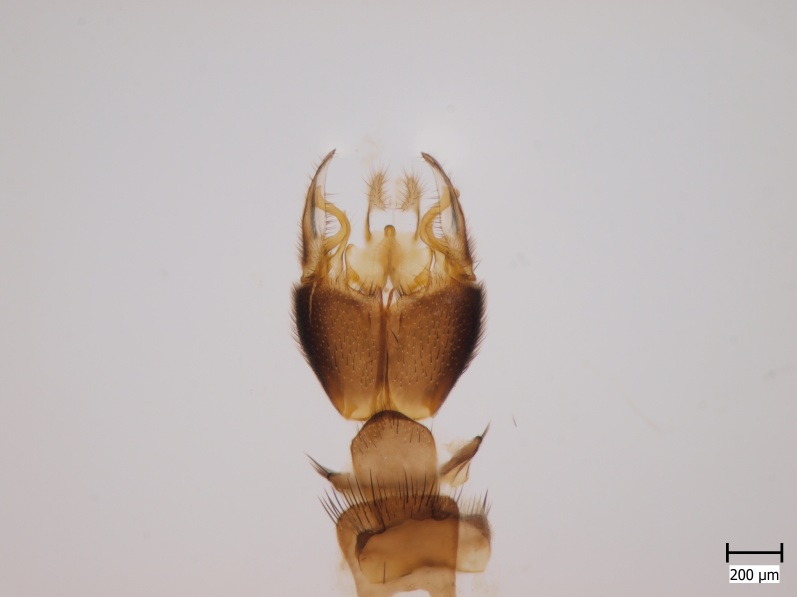
Male hypopygium, sternal view.

**Figure 5a.**
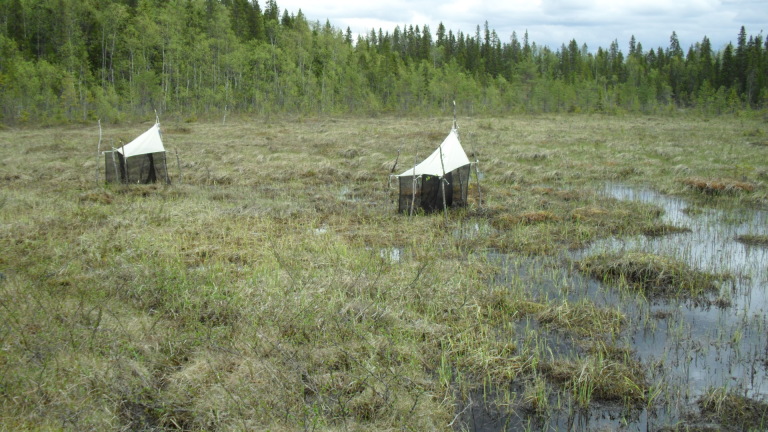
Tornio, Rakanjänkkä, rich spring fen, dominated by brown mosses such as *Scorpidium revolverns*, *Scorpidium cossoni*, *Paludella squarrosa* and *Sphagnum warnstorfii*. Vascular plant flora includes *Carex* spp., *Dactylorhiza incarnata* and *Parnassia palustris*. J. Salmela 6/2012.

**Figure 5b.**
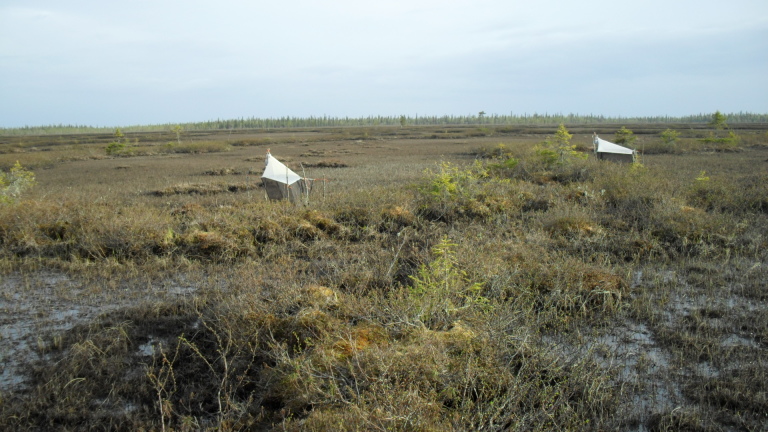
Sodankylä, Pomokaira, Kaita-aapa, aapamire, intermediate rich flark fen. Moss flora characterized by *Sphagnum* spp. and *Warnstorfia procera*. Flarks are wet, moss covered areas surrounded by the narrow, hummock level strings. Aapamires are widespread mire types in the northern and middle boreal vegetation zones of West European and East Siberian plains, and in parts of boreal Canada. Aapamires are minerogenous in contrast to bogs that only receive nutrients from rain water. J. Salmela 6/2012.

**Figure 5c.**
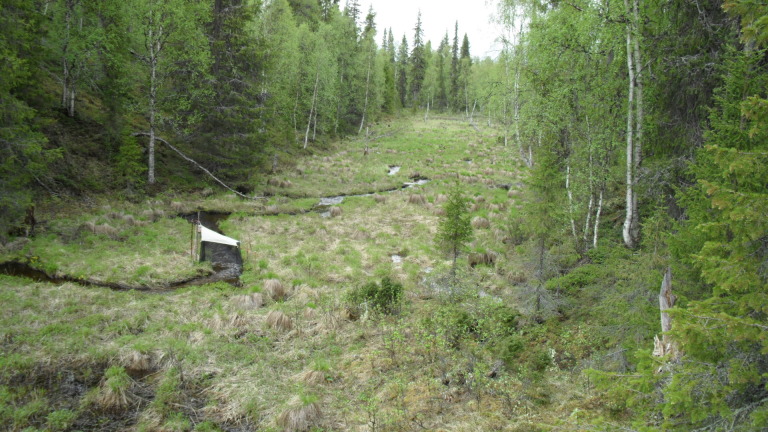
Savukoski, Joutenoja, headwater stream surrounded by boreal mixed forest. Meadow is dominated by *Filipendula ulmaria*, *Angelica archangelica*, *Viola* spp. and Urtica dioica ssp. sondenii. In the forest conifers *Pinus sylvestris* and *Picea abies* are common, with mixed *Betula* spp. J. Salmela 6/2012.

**Figure 5d.**
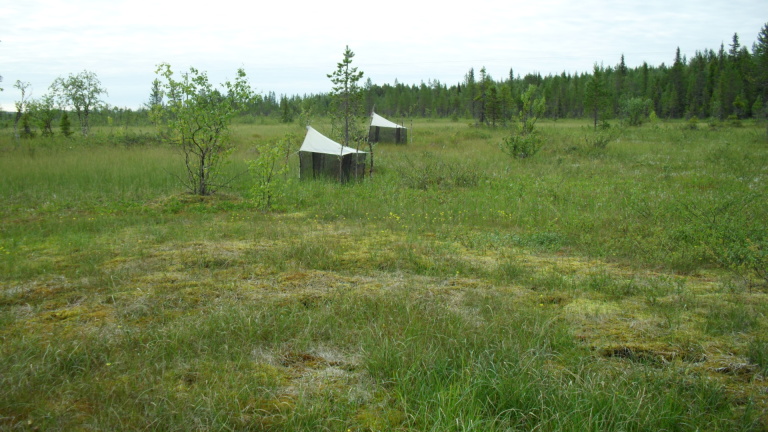
Sodankylä, Heinäaapa, rich spring fen, dominated by mosses *Palustriella falcata*, *Philonotis* sp., and *Scorpidium cossoni*. Vascular plants such as *Saxifraga hirculus*, *Saussurea alpina* and *Bartsia alpina* are conspicuous. 8/2012.

**Figure 5e.**
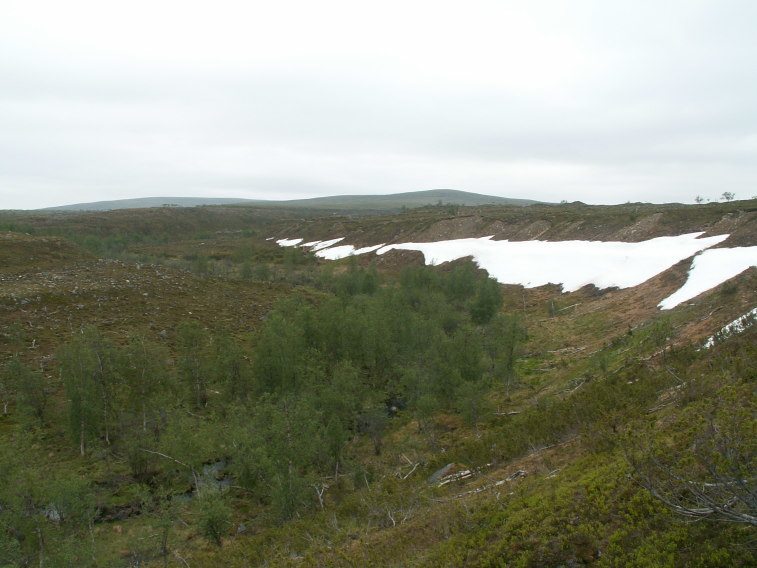
Utsjoki, Kaldoaivi, Galddasjohka. A diverse headwater stream with luxuriant riparian vegetation. The surroundigns are open fell areas, but a narrow strip of mountain birch (Betula pubescens ssp. czerepanovii) is present in the valley. J. Salmela 7/2007.

**Figure 5f.**
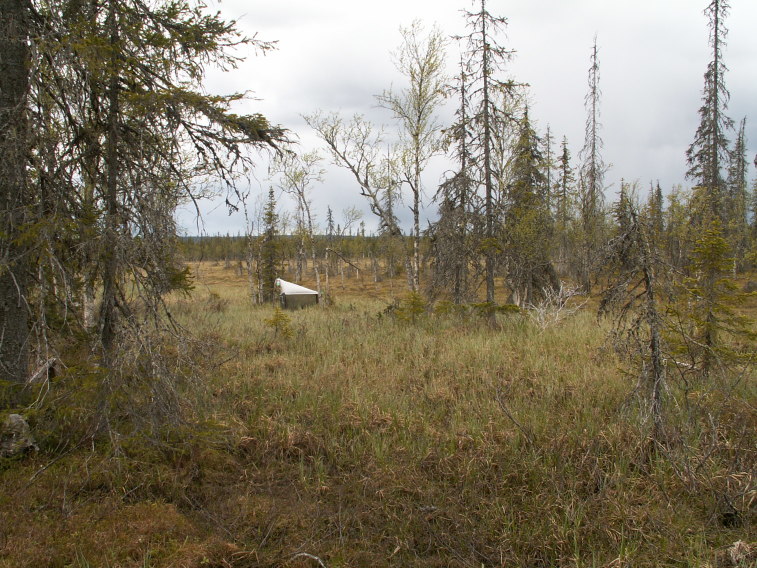
Sodankylä, Pomokaira, Paistipuolet NW, poor sloping fen surrounded by spruce mire. Characterized by large sedges (*Carex* spp.) and *Sphagnum riparium*. J. Salmela 6/2009.

**Figure 7a.**
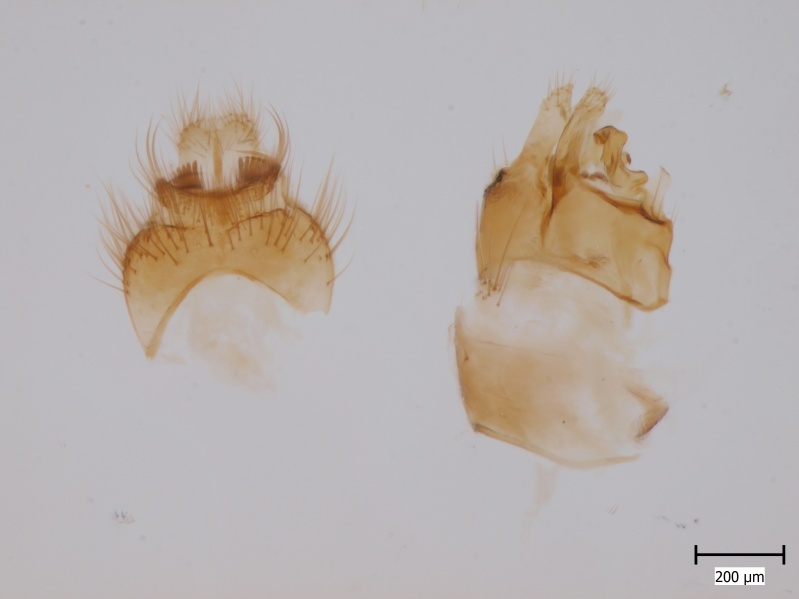
Male hypopygium, 9th tergite (left, dorsal view) and sternal synsclerite (right, ventro-lateral view).

**Figure 7b.**
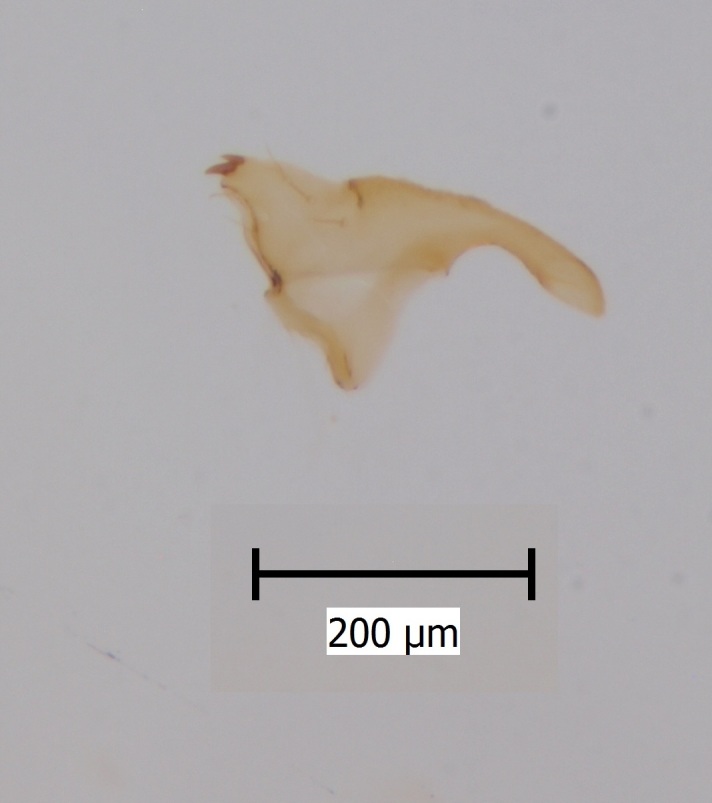
Male hypopygium, gonostylus.

**Figure 8a.**
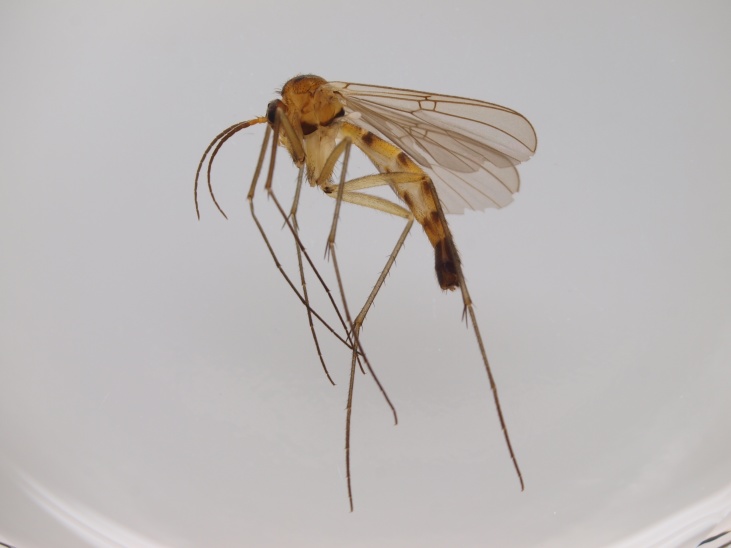
Habitus, lateral view.

**Figure 8b.**
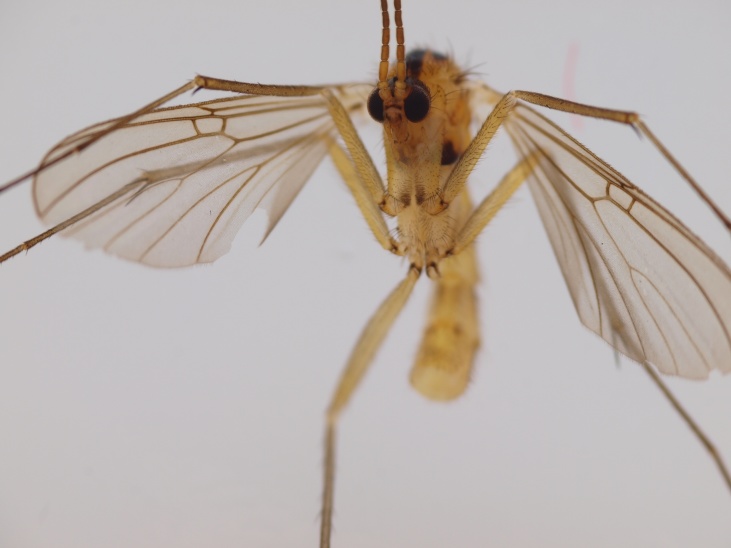
Habitus, frontal view.

**Figure 8c.**
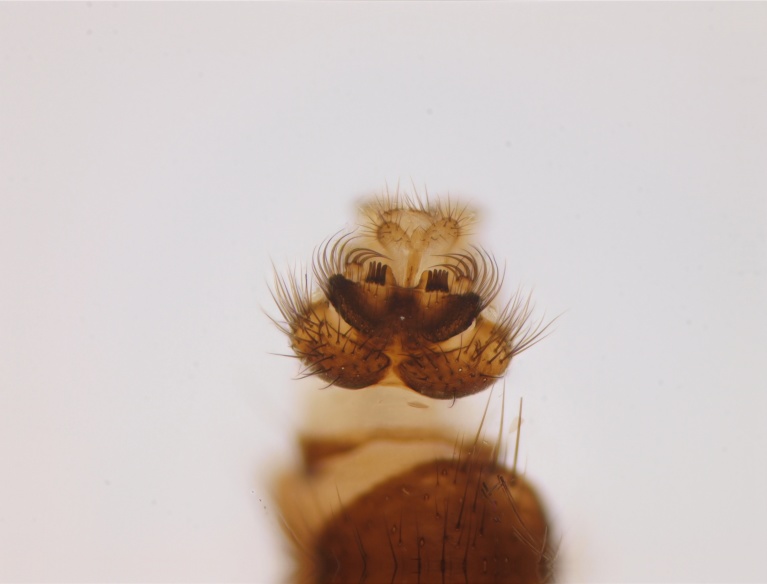
Male hypopygium, dorsal view.

**Figure 8d.**
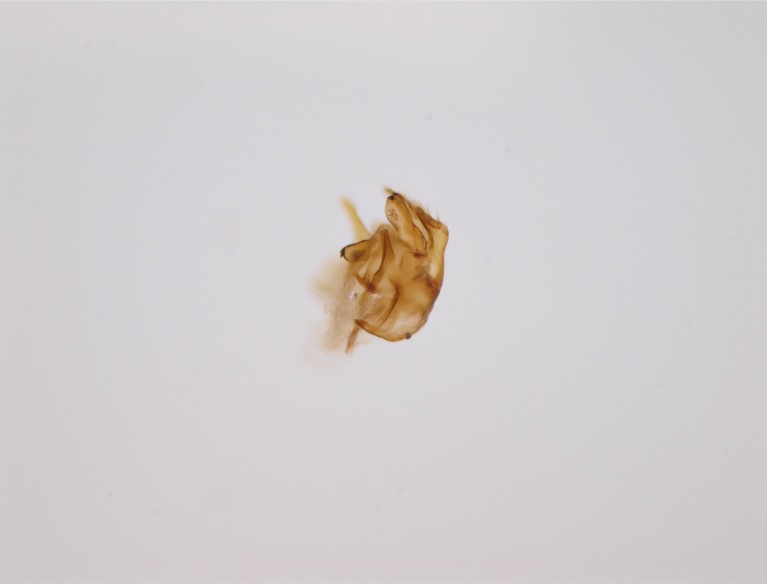
Male hypopygium, lateral view (9th tergite removed).

**Figure 8e.**
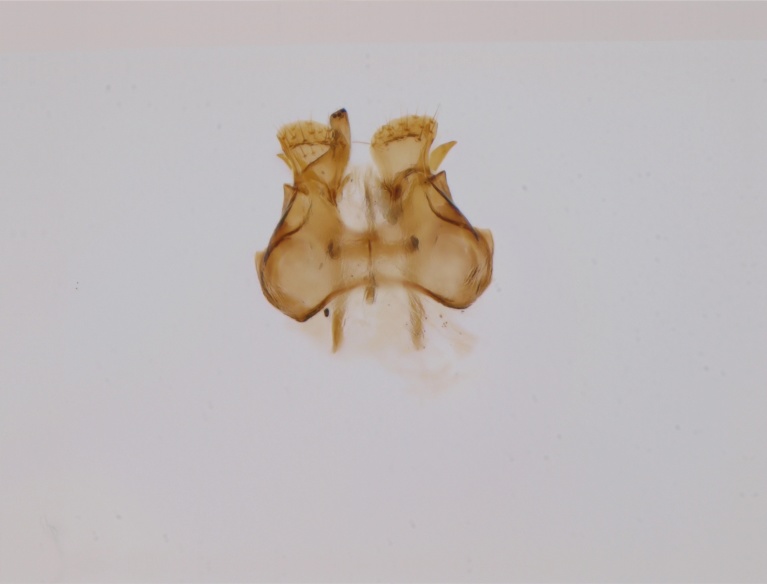
Sternal synsclerite, ventral view.

**Figure 8f.**
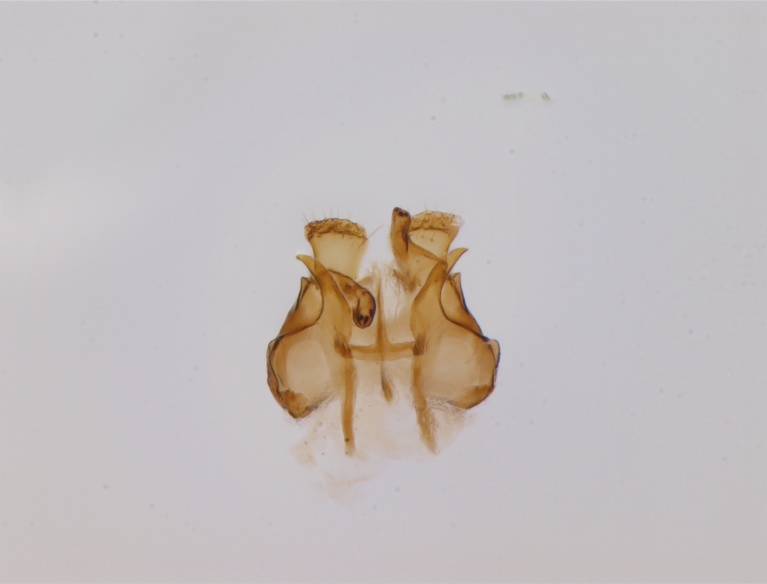
Sternal synsclerite and parameres, dorsal view.

**Figure 9a.**
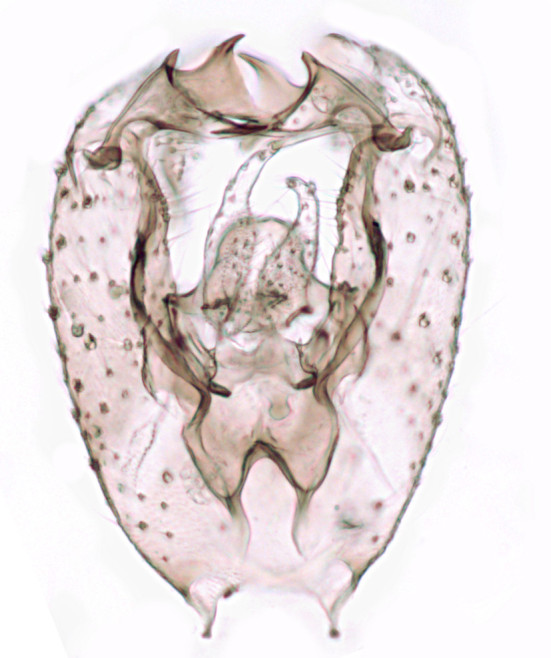
Male hypopygium, dorsal view.

**Figure 9b.**
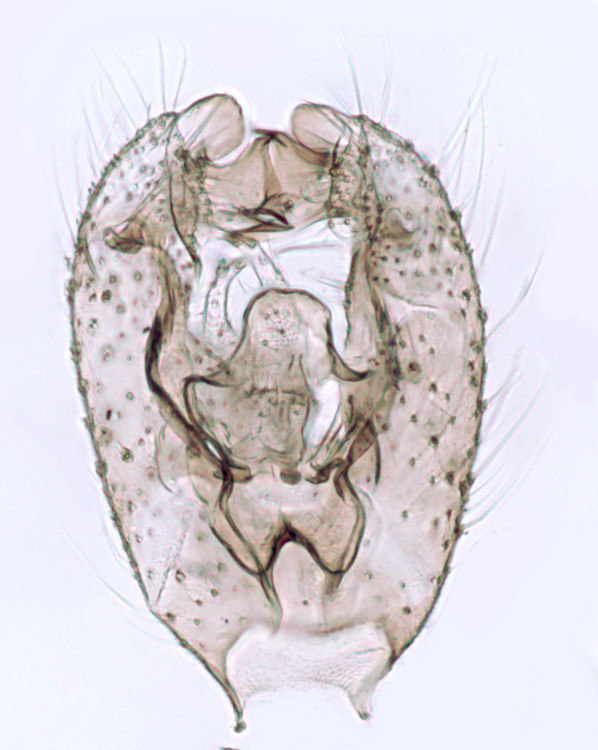
Male hypopygium, ventral view.

**Figure 11a.**
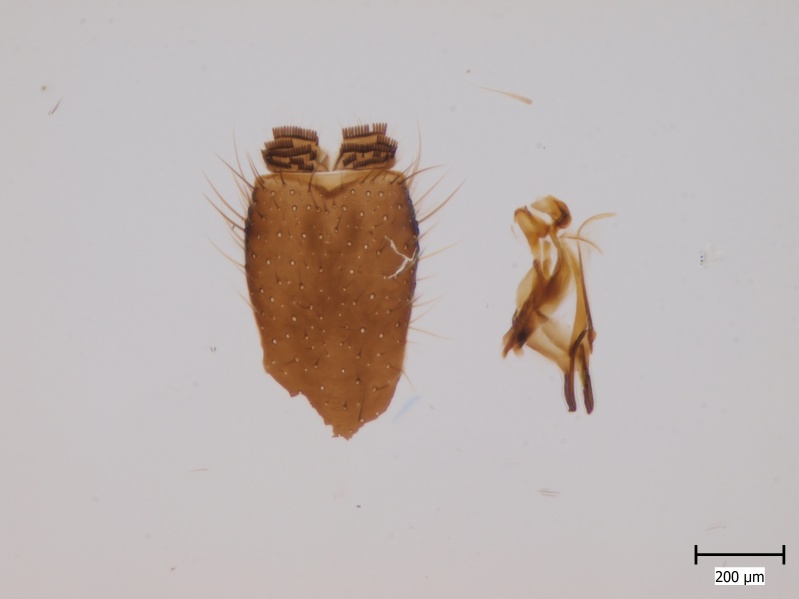
*Boletina borealis*, 9th tergite (left, dorsal viewl) and aedeagus and parameres (right, lateral view). Note the curved, long and thin apices of the parameres.

**Figure 11b.**
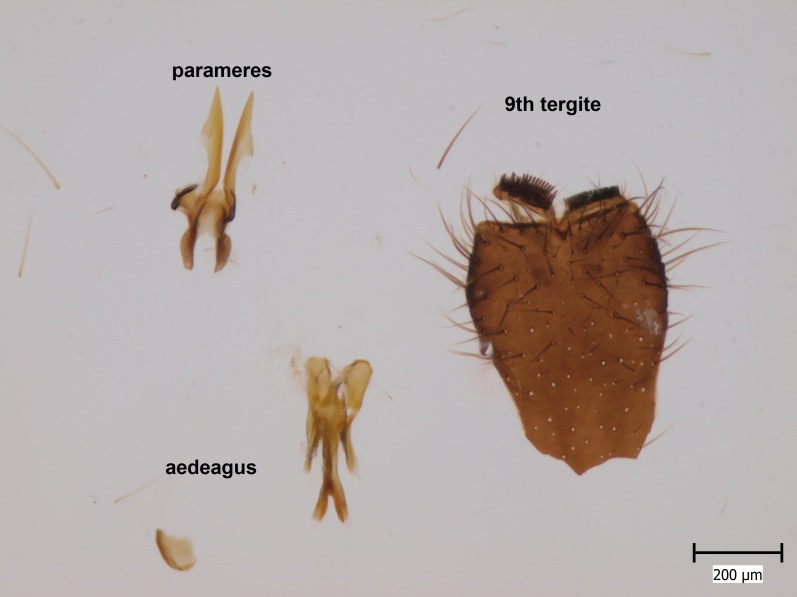
*Boletina intermedia*, parameres, aedeagus and 9th tergite (all in dorsal view). Note the spear-shaped, stout parameres.

**Figure 14a.**
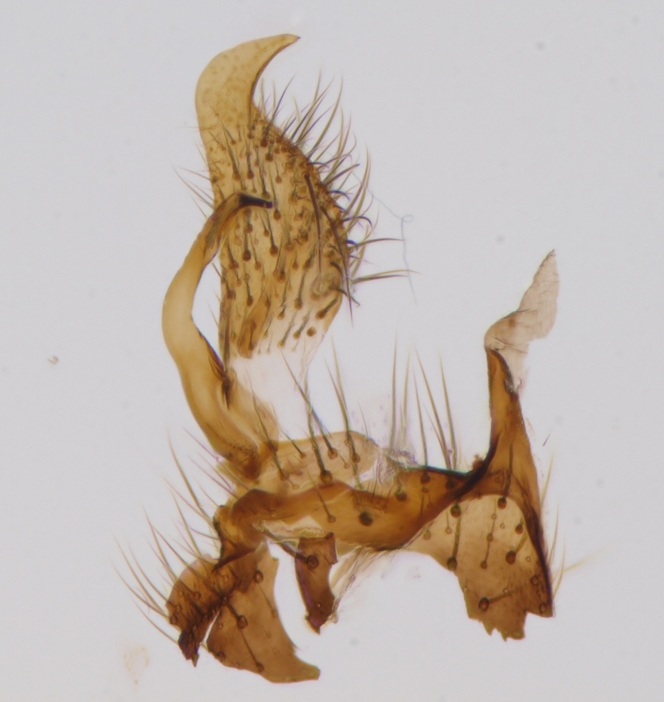
Gonostylus, lateral view.

**Figure 14b.**
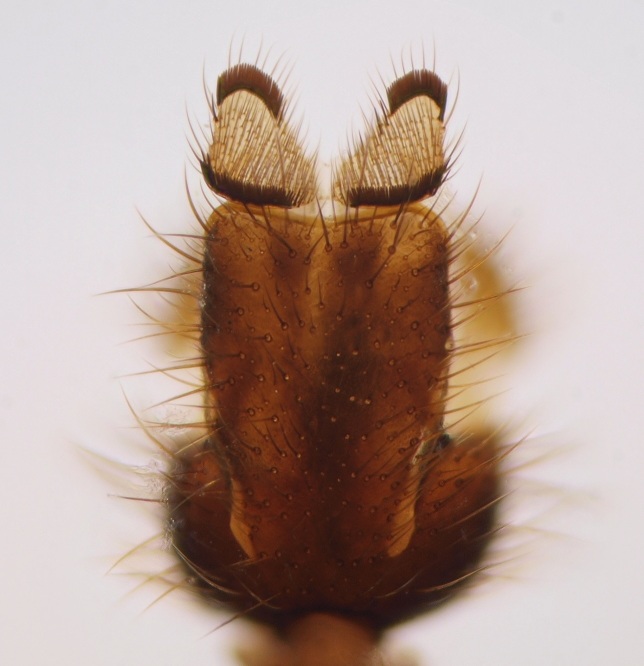
9th tergite, dorsal view.

**Figure 18a.**
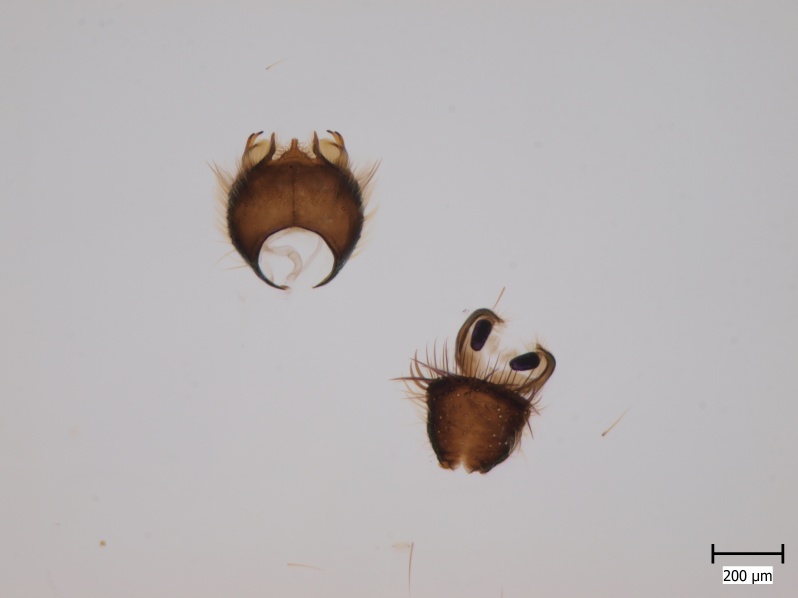
Male hypopygium, 9th tergite (top, dorsal view) and cerci (ventral view).

**Figure 18b.**
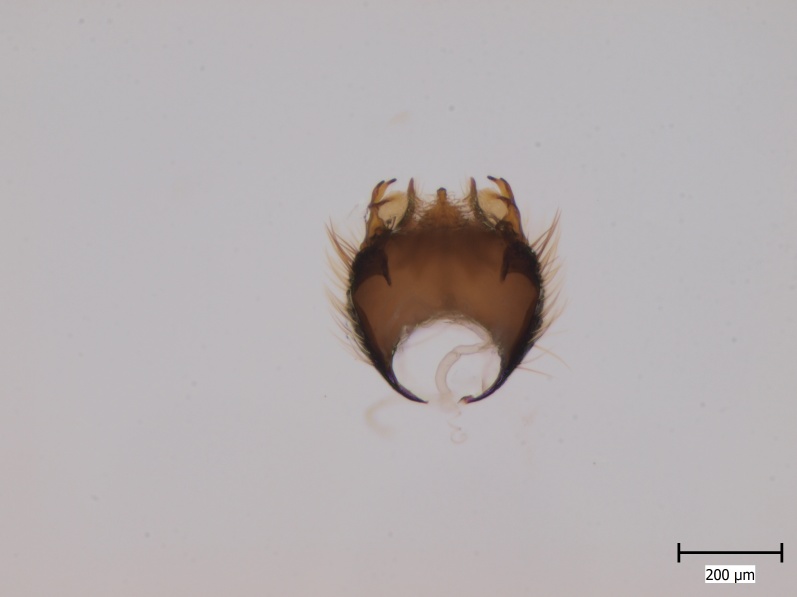
Male hypopygium, 9th tergite (ventral view).
